# Supplementary material for: PEBP balances apoptosis and autophagy in whitefly upon arbovirus infection
Source: Nat Commun. 2022 Feb 11;13:846. doi: 10.1038/s41467-022-28500-8 (PMC8837789; doi:10.1038/s41467-022-28500-8)
Supplement: Supplementary file 1 — Supplementary Information [file 41467_2022_28500_MOESM1_ESM.pdf]

# Supplementary Information

PEBP balances apoptosis and autophagy in whitefly upon arbovirus infection

Wang *et al*.

Supplementary Figure 1. Homology analysis of the candidate whitefly PEBP.

Supplementary Figure 2. Whitefly PEBP4 is primarily located in cytoplasmic membrane.

Supplementary Figure 3. PEBP4 is continuously consumed during TYLCV infection.

Supplementary Figure 4. PEBP4 mediates the MAPK pathway by interacting with Raf1.

Supplementary Figure 5. PEBP4 interacts with ATG8.

Supplementary Figure 6. Comprehensive interaction of apoptosis and autophagy verified by RNA interference.

Supplementary Table 1. Putative MED *Bemisia tabaci* proteins that interacted with coat protein of tomato yellow leaf curl virus in yeast two-hybrid screening.

Supplementary Table 2. Primer List.

A

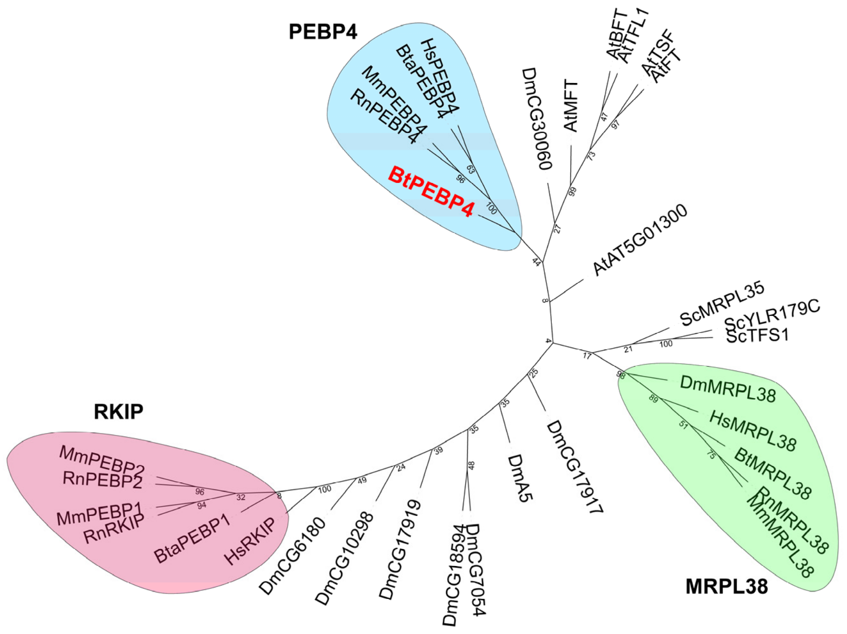


B

```
BtPEBP4    MASVSVMLSMVLQTYSVSVVSMAHSLHLLGFPPRKVRSRKNKENMERTDSPESMEMESTKGTESTKKTRRTKSTKTKSTK   80
HsPEBP4    ................................................................................    0
MmPEBP4    ................................................................................    0
BtaPEBP4   ................................................................................    0
RnPEBP4    ................................................................................    0

BtPEBP4    SKKPKSTKSTKTKSTKARSTKITKTKKTPTPKIKKSTRNTRRPRISKNTESTKISKTTEVIGSTEILTTTRHGKTRSRPK  160
HsPEBP4    ......................................MGWTMRLVTAALLLGLMMVVTGDEDE...............N   27
MmPEBP4    ...............................MTMKLVAAALCLSLLAAGLWVGLSLTAESIEEGKPGGEKPGGGKPGGSG   49
BtaPEBP4   ......................................MGWTMRLVTAALLLGLAVAVTGEEED...............A   27
RnPEBP4    .................................MKLVAAAFGLSLLVAGLWVGLSLTSNSDGGGKPGG...GGG..GGGG   42

BtPEBP4    NISPFDAIFRENEIVPQIVNNTPIYKCEVEYGKEKIHFGNNMTADQIAEKPTLVKWPTQSG.AFYTLLMFGPDVPTREKS  239
HsPEBP4    SPCAHEALLDED..TLFCQGLEVFYPELGNIGCKVVPDCNNYRQKITSWMEPIVKFPGAVDGATYILVMVDPDAPSRAEP  105
MmPEBP4    RGCFLPPLPKED..VSLCRNLEVFYMEMGNISCKIVPKCNLYRQKITAWQAPIVKFHTALDGALYLLVMVDPDAPSRSNP  127
BtaPEBP4   DLCVYEALPDND..AVLCKGLKVFYPELGNIGCMIVPECNNYRQKITTWPEPIVKFPQALDDAAYILVMVDPDAPSRSSP  105
RnPEBP4    RGCFLPPLPKED..VSLCRNLEVFYMEMGNISCKVVPKCNQYRRKIMTWQSPIVKFHGALD...................  101

BtPEBP4    CEMMWRHWAVGNIPKND....IHKGDTLVEYENIEPFYRAGFHRILFFIYKQPGRRRLNFTSSKGVIKKGAKWEVRGVWY  315
HsPEBP4    RQRFWRHWLVTDIKGADLKKGKIQGQELSAYQAPSPPAHSGFHRYQFFVYLQEGKV.ISLLPKENKTRG........SWK  176
MmPEBP4    VMKYWRHWLVSNITGADMKSGSIRGNVLSDYSPPTPPPETGLHRYQFFVYLQGDRD.ISLSVEEKADLG........GWN  198
BtaPEBP4   KARFWRHWLVSDIKGSDMKIGKIQGQELSPYHPPSPPAHSGLHRYQFFVYLQEGRT.ISLSPKENKTRG........SWK  176
RnPEBP4    .........VSELC.......WLKENVGPQLAFSSPGP..........VFLLTN...IKWPAFENVSL............  140

BtPEBP4    IERFLAKFNLTEPIAANFFFLYDLNPHPEIPQVTVDPLSLIR.........                              357
HsPEBP4    MDRFLNRFHLGEPEASTQFMTQNYQDSPTLQAPRERASEPKHKNQAEIAAC                              227
MmPEBP4    LDKFLQQYGLRDPDTSTQFMTQFDEELSSEFGRINDDQEQFNQK.......                              242
BtaPEBP4   MDKFLSHFHLTEPEASTQFMTQYYLDGLSRQNPGVVSNETTDKPEPK....                              223
RnPEBP4    ...FSSHRKLKSPKERLD.................................                              155
```

**Supplementary Figure 1. Homology analysis of the candidate whitefly PEBP.** (A) The PEBP protein sequences that identified from 7 species and the candidate PEBP in whitefly were taken as input sequences, and the phylogenetic tree was constructed through the neighbor-joining (N-J) method with 1000 bootstrap replicates. Three major clusters were named according to the dominate proteins. (B) The candidate PEBP in whitefly was named as BtPEBP4, and the amino acid sequence was aligented with other PEBP4s in mammals.

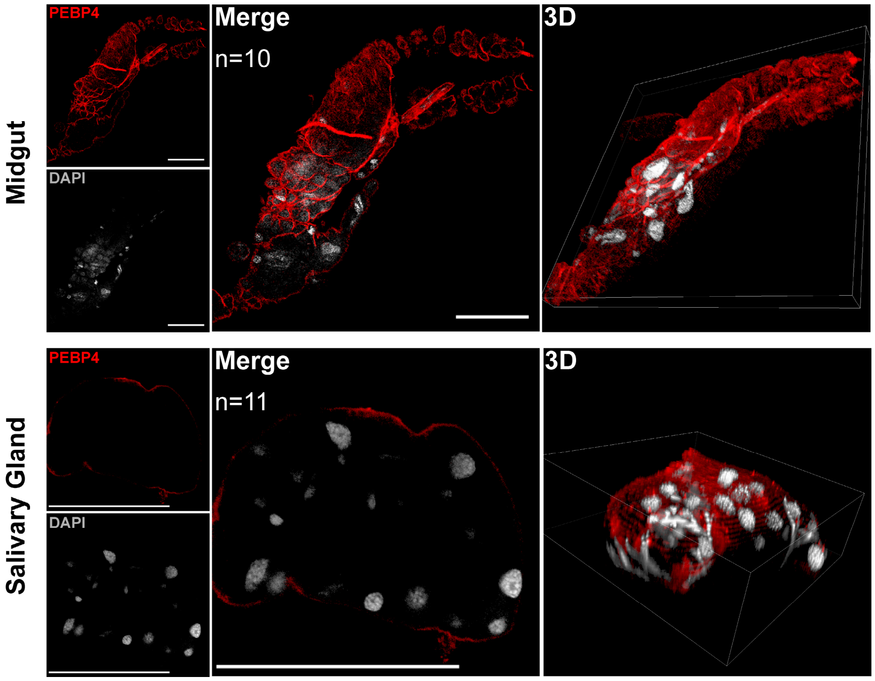


**Supplementary Figure 2. Whitefly PEBP4 is primarily located in cytoplasmic membrane.** PEBP4 (red), and nuclei (white) were labeled with immunofluorescence. Sample sizes (n) for statistical tests indicated in the panels refer to biologically independent whitefly. Midgut and salivary gland were dissected from untreated whitefly. 3D images were simulated based on Z-Stack scanning. Scale bar represents 50 μm. Sample sizes (n) for statistical tests indicated in the panels refer to biologically independent whitefly.

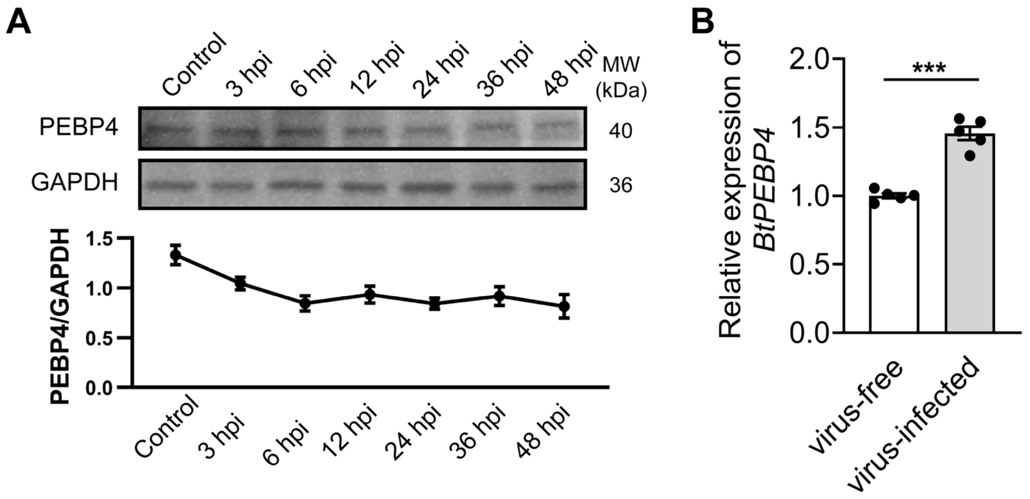


**Supplementary Figure 3. PEBP4 is continuously consumed during TYLCV infection.** (A) The dynamic of the PEBP4 protein abundance in whitefly after virus acquisition was analyzed by immunoblotting, and the grey value of PEBP4 was quantified by ImageJ software compared to GAPDH, six independent samples were used for each treatment. (B) The transcriptional expression of *BtPEBP4* was analyzed by qPCR after 48 hours TYLCV acquisition, five independent samples were used for each treatment. Values in bar or line plots represent mean ± SEM. All data were checked for normality by the Wilk-Shapiro test. Two-sided paired t-test was used to separate the means of normally distributed data.

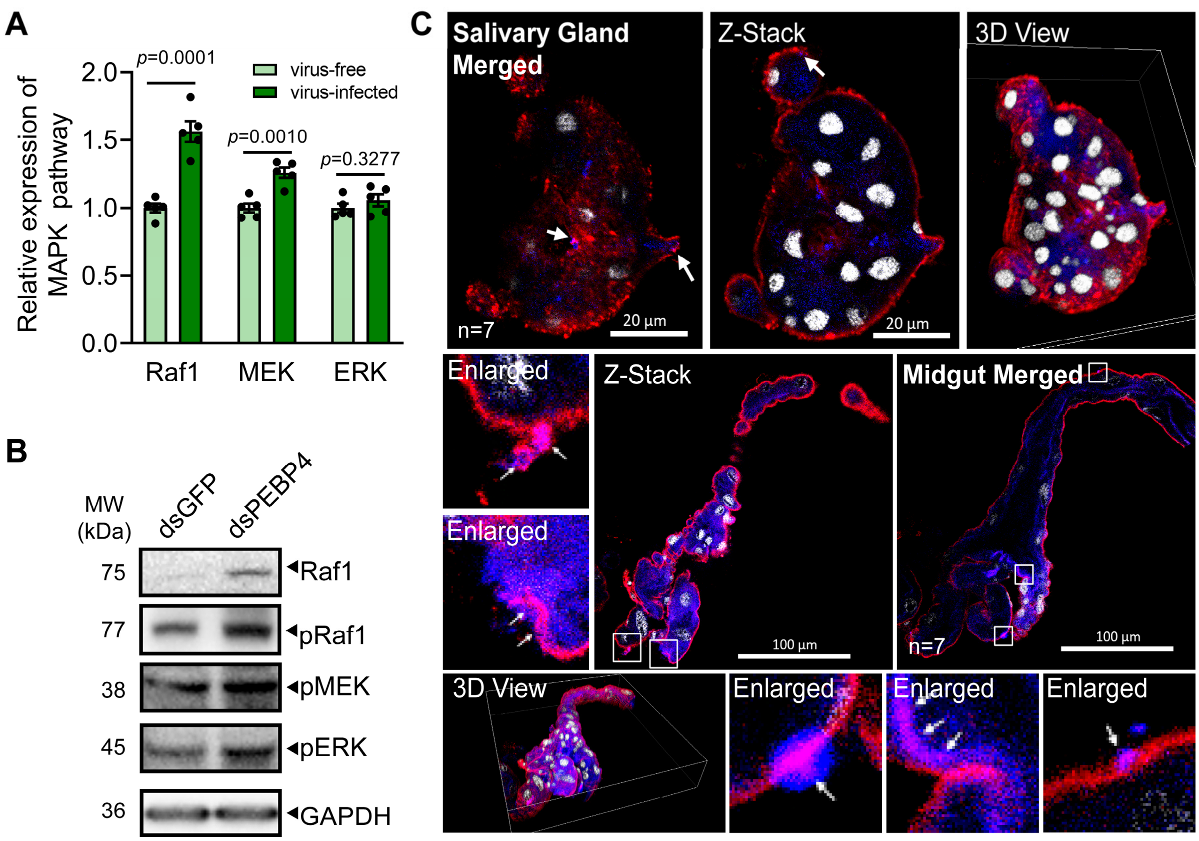


**Supplementary Figure 4. PEBP4 mediates the MAPK pathway by interacting with Raf1.** (A) Transcriptional expression of *Raf1*, *MEK*, and *ERK* after 48 hours TYLCV acquisition, five independent samples were used for each treatment. Values in bar plot represent mean ± SEM. All data were checked for normality by the Wilk-Shapiro test. Two-sided paired t-test was used to separate the means of normally distributed data. (B) The phosphorylation of MAPK pathway after PEPBP4 knockdown was analyzed by immunoblotting. (C) Raf1 (blue) and PEBP4 (red) of nonviruliferous whitefly were labeled and localized by immunofluorescence. The nuclei were stained by DAPI (white). Z-Stack image showed another layer of the same sample. 3D images were simulated from all scanned Z-Stack layers. Sample sizes (n) for statistical tests indicated in the panels refer to biologically independent whitefly.

A

Query: >PEBP4

| Motif | Start | End | Pattern | PSSM Score | LIR in Anchor |
|---|---|---|---|---|---|
| WxxL | 163 | 168 | SPFDAI | 9 | Yes |
| WxxL | 221 | 226 | GAFYTL | 5 | No |
| WxxL | 264 | 269 | VEYENI | 9 | No |
| WxxL | 275 | 280 | AGFHRI | 6 | No |
| WxxL | 331 | 336 | ANFFFL | 7 | No |

**ANCHOR Positions - Start and End**

| Anchor | Start | End |
|---|---|---|
| 1 | 1 | 38 |
| 2 | 96 | 103 |
| 3 | 137 | 148 |
| 4 | 162 | 175 |
| 5 | 179 | 189 |

iLIR Autophagy Database

B

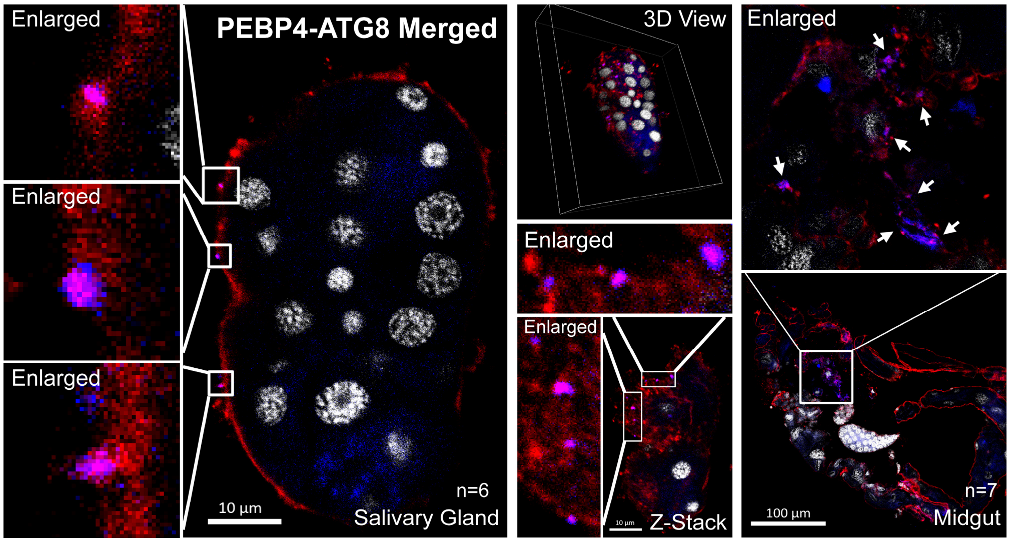


**Supplementary Figure 5. PEBP4 interacts with ATG8.** (A) LIR motifs are necessary for direct interaction with the autophagy-related ATG8 family. The amino acid sequence of whitefly PEBP4 was analyzed and predicted by the online tool of LIR motif searching in iLIR Autophagy Database (https://ilir.warwick.ac.uk/index.php). (B) ATG8 (blue) and PEBP4 (red) of nonviruliferous whitefly were labeled and localized by immunofluorescence. The nuclei were stained by DAPI (white). Z-Stack image showed another layer of the same sample. 3D images were simulated from all scanned Z-Stack layers. Sample sizes (n) for statistical tests indicated in the panels refer to biologically independent whitefly.

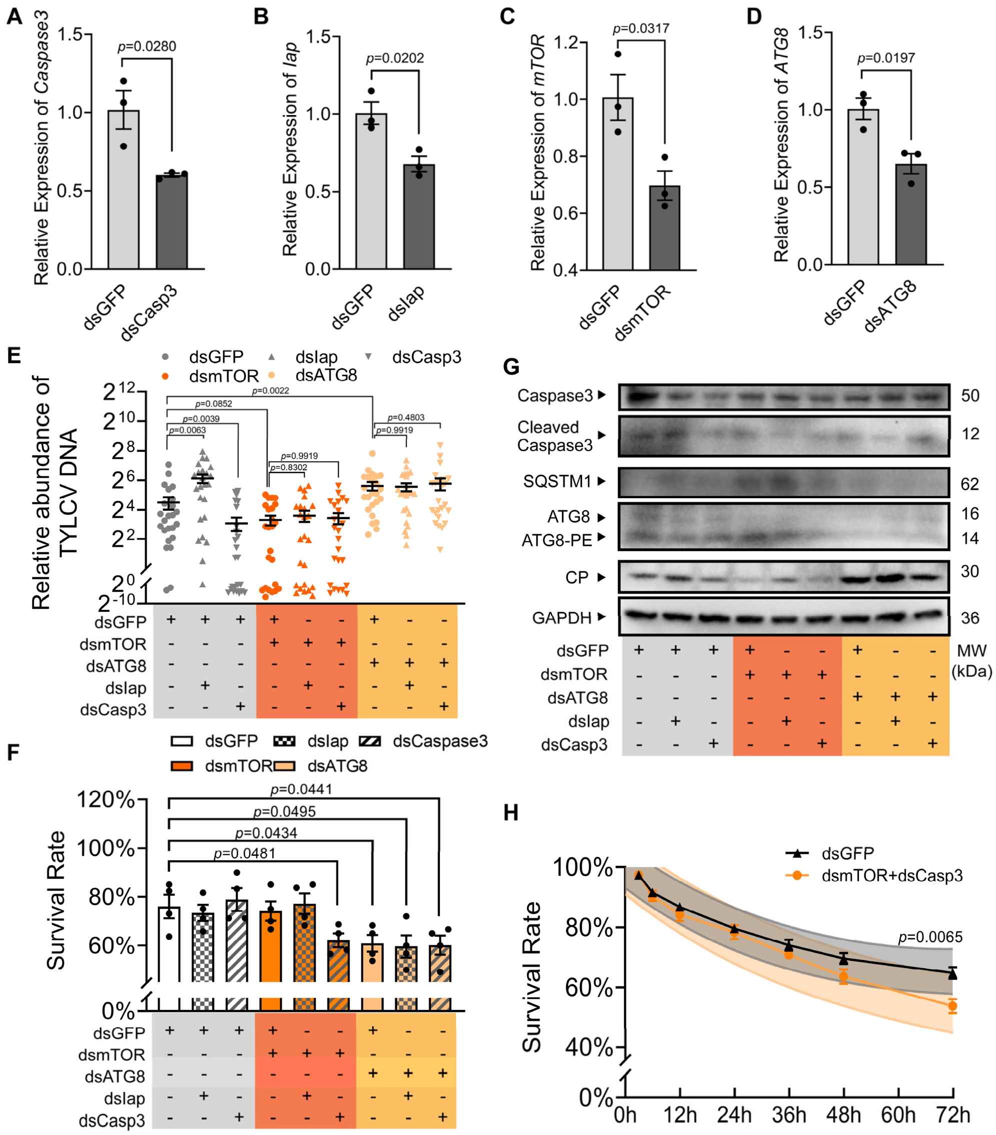

**Supplementary Figure 6. Comprehensive interaction of apoptosis and autophagy verified by RNA interference.** (A-D) RNAi efficiency of dsIap, dsCaspase3, dsmTOR and dsATG8 feeding (dsGFP as control), three independent samples were used for each treatment. (E-G) After feeding on combinations of two different dsRNAs as indicated, whiteflies were collected and transferred to infected plants for 24 hours TYLCV acquisition. (E) Relative virus DNA content per whitefly was determined by qPCR, 24 independent whiteflies were sampled for each treatment. (F) Whitefly survival rate was recorded after dsRNA feeding and virus acquisition, four independent samples were used for each treatment. (G) 100 whiteflies per replicate from the same treatment as shown above was sampled for monitoring dsRNA combination effects on apoptosis, autophagy and CP abundance. (H) Nonviruliferous whiteflies fed with both dsmTOR (autophagy activation) and dsCaspase3 (relieve autophagy limitation of apoptosis) were sampled for survival rate measurement, dsRNA-contained diet was replaced every 24 h, prediction bands (95% confidential interval) of whitefly survival rate were shown based on linear quadratic survival model, five independent samples were used for each treatment. Values in all bars or dot plots represent mean ± SEM. All data were checked for normality by the Wilk-Shapiro test. Two-sided paired t-test was used to separate the means of normally distributed data, while

Mann-Whitney test was used to analyze nonparametric distributed data, no multiple comparisons were performed in each test.

**Supplementary Table 1**. Putative MED *Bemisia tabaci* proteins that interacted with coat protein of tomato yellow leaf curl virus in yeast two-hybrid screening.

| Query No. | Score[a] | E-value[a] | Identity(%)[a] | Genome ID | Score[b] | E-value[b] | Identity(%)[b] | Acession No. | Description[c] |
|---|---|---|---|---|---|---|---|---|---|
| 1 | 25.0 | 5.4 | 43.3 | BTA008214.1 | 180 | 5.00E-47 | 27.72% | XP_018897595.1 | PREDICTED: uncharacterized protein LOC109030865 isoform X1 [Bemisia tabaci] |
| | | | | | 120 | 3.00E-27 | 24.76% | XP_018915628.1 | PREDICTED: glutamate receptor U1-like [Bemisia tabaci] |
| 2 | 94.4 | 4.80E-25 | 93.9 | BTA012003.1 | 263 | 9.00E-91 | 98.43% | XP_018901274.1 | PREDICTED: NADH dehydrogenase [ubiquinone] 1 beta subcomplex subunit 5, mitochondrial [Bemisia tabaci] |
| 3 | 149.0 | 1.82E-42 | 97.3 | BTA000233.1 | 516 | 0 | 96.73% | XP_018900154.1 | PREDICTED: uncharacterized protein LOC109032456 isoform X2 [Bemisia tabaci] |
| | | | | | 33.5 | 1.7 | 25.76% | RZF41284.1 | obscurin isoform X3 [Sipha flava] |
| 4 | 523.0 | 0 | 99.2 | BTA010352.1 | 1000 | 0 | 99.59% | XP_018911064.1 | PREDICTED: esterase E4-like [Bemisia tabaci] |
| 5 | 33.1 | 0.017 | 31.5 | BTA017005.1 | 564 | 0 | 96.50% | XP_018915181.1 | PREDICTED: probable dimethyladenosine transferase [Bemisia tabaci] |
| 6 | 26.2 | 6.5 | 71.4 | BTA006720.1 | 564 | 0 | 96.50% | XP_018915181.1 | PREDICTED: rho GTPase-activating protein 21 isoform X8 [Bemisia tabaci] |
| 7 | 265.0 | 2.89E-85 | 100.0 | BTA007379.1 | 567 | 0 | 99.62% | XP_018901717.1 | PREDICTED: alpha-N-acetylgalactosaminidase-like [Bemisia tabaci] |
| 8 | 227.0 | 4.41E-77 | 93.0 | BTA023875.1 | 267 | 4.00E-93 | 100.00% | XP_018910818.1 | PREDICTED: 40S ribosomal protein S15Aa-like [Bemisia tabaci] |
| 9 | 38.5 | 0.006 | 50.0 | BTA015065.1 | 286 | 4.00E-87 | 34.67% | XP_025417167.1 | ATP-binding cassette sub-family B member 10, mitochondrial [Sipha flava] |
| 10 | 157.0 | 2.10E-46 | 85.4 | BTA024002.1 | 293 | 2.00E-102 | 93.79% | XP_018899372.1 | PREDICTED: WAS/WASL-interacting protein family member 1-like [Bemisia tabaci] |
| 11 | 311.0 | 2.15E-97 | 99.3 | BTA011564.1 | 1976 | 0 | 99.36% | XP_018904225.1 | PREDICTED: C-1-tetrahydrofolate synthase, cytoplasmic [Bemisia tabaci] |
| 12 | 223.0 | 6.24E-69 | 94.1 | BTA012527.1 | 913 | 0 | 84.84% | XP_018917484.1 | PREDICTED: uncharacterized protein LOC109044293 [Bemisia tabaci] |
| | | | | | 34.7 | 1.9 | 27.63% | XP_027848922.1 | ethylmalonyl-CoA decarboxylase-like isoform X1 [Aphis gossypii] |
| 13 | 28.9 | 3.5 | 44.4 | BTA029690.1 | 2754 | 0 | 99.85% | XP_018903654.1 | PREDICTED: uncharacterized protein LOC109034784 [Bemisia tabaci] |
| | | | | | 1675 | 0 | 98.90% | XP_018903657.1 | PREDICTED: PAX3- and PAX7-binding protein 1 [Bemisia tabaci] |
| 14 | 106.0 | 2.57E-27 | 99.0 | BTA014874.1 | 1469 | 0 | 98.28% | XP_018901035.1 | PREDICTED: proteoglycan 4-like [Bemisia tabaci] |
| 15 | 145.0 | 1.14E-43 | 97.5 | BTA017133.1 | 518 | 0 | 99.21% | XP_018902549.1 | PREDICTED: probable 39S ribosomal protein L24, mitochondrial [Bemisia tabaci] |
| **16** | **306.0** | **1.08E-104** | **99.3** | **BTA013106.1** | **722** | **0** | **97.51%** | **XP_018910109.1** | **PREDICTED: uncharacterized protein LOC109039182 isoform X1 [Bemisia tabaci]** |
| | | | | | **336** | **3.00E-116** | **98.08%** | **XP_018910114.1** | **PREDICTED: phosphatidylethanolamine-binding protein homolog F40A3.3-like isoform X4 [Bemisia tabaci]** |
| 17 | 26.9 | 0.43 | 44.4 | BTA020855.1 | 459 | 9.00E-166 | 99.55% | XP_018897453.1 | PREDICTED: uncharacterized protein LOC109030777 [Bemisia tabaci] |
| | | | | | 126 | 1.00E-34 | 39.41% | VVC29685.1 | Immunoglobulin subtype,Immunoglobulin-like domain,Immunoglobulin-like fold [Cinara cedri] |
| 18 | 62.4 | 2.41E-10 | 34.4 | BTA027805.1 | 347 | 3.00E-108 | 41.13% | QEP09172.1 | cytochrome c oxidase subunit I [Aphaena discolor nigrotibiata] |
| 19 | 27.3 | 0.53 | 39.3 | BTA004184.1 | 4908 | 0 | 98.87% | XP_018899018.1 | PREDICTED: uncharacterized protein LOC109031748 [Bemisia tabaci] |
| | | | | | 957 | 3.00E-173 | 65.36% | XP_025414375.1 | protein FAM135A [Sipha flava] |
| 20 | 26.2 | 1.7 | 41.9 | BTA000356.1 | 1333 | 0 | 98.76% | XP_018901482.1 | PREDICTED: glucose dehydrogenase [FAD, quinone]-like [Bemisia tabaci] |
| 21 | 25.4 | 1.8 | 39.1 | BTA026158.1 | 623 | 0 | 99.35% | XP_018915405.1 | PREDICTED: facilitated trehalose transporter Tret1-like isoform X1 [Bemisia tabaci] |
| 22 | 29.6 | 4 | 34.5 | BTA021511.2 | 1143 | 0 | 96.05% | XP_018915012.1 | PREDICTED: cytochrome P450 4g15-like isoform X1 [Bemisia tabaci] |
| 23 | 27.3 | 0.41 | 41.9 | BTA019316.1 | 1060 | 0 | 100.00% | XP_018913374.1 | PREDICTED: kelch-like protein 30 [Bemisia tabaci] |

a Query sequences were used in local Blastx to identify full amino acid sequences in MED whitefly genome

data.

b Selected proteins were used Blastp and compared with the database of *Hemiptera* to predict putative function.

c If the subject protein was predicted as “uncharacterized protein”, a further PSI-Blast result with specific description would be supplied below the previous result.

**Supplementary Table 2. Primer List.**

| Name | Primer Sequence (5'-3') | Accession No. |
|---|---|---|
| *Actin* | TCTTCCAGCCATCCTTCTTG | XM_019042718.1 |
| | CGGTGATTTCCTTCTGCATT | |
| *Caspase1* | TGTTGGAGACGGTATGGA | BTA009205.1 |
| | ATGAAGACAGTGCTTAATGC | |
| *Caspase3* | CATCACGATCAACGGGACCA | BTA015946.1 |
| | TGTCGATGTGCTGCTCGAAT | |
| *TYLCV* | GAAGCGACCAGGCGATATAA | AM282874.1 |
| | GGAACATCAGGGCTTCGATA | |
| *PEBP4* | CCAGGAAGAAGGAGATTGAA | BTA013106.1 (Sequenced PEBP4 CDs region is appended below) |
| | GCTATTGGTTCGGTCAGAT | |
| *Iap* | ATGTCTCGGAATCATCTCAG | BTA000132.1 |
| | ACCACAAGGAAGGAATGC | |
| *Bcl-2* | GCTAATGACACAGACTGGAT | BTA025366.1 |
| | GAGATGAAGTTCGTGAGGAA | |
| *Raf1* | AAGTGCTGATGATAGTGCTA | BTA014695.1 |
| | CATCGGTAGACAGTTCCAA | |
| *MEK* | TTGAGTTGCTGGACTACATAG | BTA026985.1 |
| | TTATCCGCCTCGCACTTA | |
| *ERK* | CAATGATAAGGCACGCAAT | BTA017617.1 |
| | CATAATACTGTTCCAGGTAAGG | |
| *ATG3* | CGTTTAAGGGAACAGCACTTG | BTA008054.1 |
| | CCAGATTGTCTCCAGCAGCA | |
| *ATG9* | TTGCCATCATTAACTTTCTGCT | BTA017980.1 |
| | AGGGTTCCTGGTTCACGC | |
| *ATG8* | TACACTTGAGACCAGAGGA | BTA002927.2 |
| | CTTCTTCGTGATGTTCTTGA | |
| *ATG12* | TCAAAGCCACTGGTAACGC | BTA009484.1 |
| | TCTGGTCCGGAGCAGGAGC | |
| dsGFP | CTCGTGACCACCCTGACCTAC | |
| | GTTCACCTTGATGCCGTTCTT | |
| | T7-CTCGTGACCACCCTGACCTAC | |
| | T7-GTTCACCTTGATGCCGTTCTT | |
| dsPEBP4 | CAACCACAAGGCACGAA | |
| | TCCTTCTTCCTGGCTGTT | |
| | T7-CAACCACAAGGCACGAA | |
| | T7-TCCTTCTTCCTGGCTGTT | |

| | | |
|---|---|---|
| dsCasp3 | GATGGAGCGACAGATGAC | |
| | GGCGAGCAGAAGTTGAA | |
| | T7-GATGGAGCGACAGATGAC | |
| | T7-GGCGAGCAGAAGTTGAA | |
| dsIap | GCATACCGTGAGCATACC | |
| | TCGTTGGAAGTTGATGATGA | |
| | T7-GCATACCGTGAGCATACC | |
| | T7-TCGTTGGAAGTTGATGATGA | |
| dsmTOR | AGGCTGTGGTTGGTCTT | |
| | CGGATTGTGCTCTGTCTT | |
| | T7-AGGCTGTGGTTGGTCTT | |
| | T7-CGGATTGTGCTCTGTCTT | |
| dsATG8 | GCTGAGCATGTGTTTGAAA | |
| | AGTCTTCTTCGTGATGTTCT | |
| | T7-GCTGAGCATGTGTTTGAAA | |
| | T7-AGTCTTCTTCGTGATGTTCT | |
| PEBP4 | CGCGAATTCATGGCTTCCGTCTCCGTAAT | EcoRI/XhoI |
| (BL21) | CGCCTCGAGTCATCGAATTAGTGACAGTGG | (pGEX-4T-1) |
| PEBP4 pep1 | CGCGAATTCATGGCTTCCGTCTCCGTAAT | EcoRI/XhoI |
| (BL21) | CGCTCGAGTCAAGGCCACTTGACTAACGTTG | (pGEX-4T-1) |
| PEBP4 pep2 | GCGAATTCACGCAGAGTGGTGCTTTTTA | EcoRI/XhoI |
| (BL21) | CGCCTCGAGTCATCGAATTAGTGACAGTGG | (pGEX-4T-1) |
| Raf1 | CGCGGAATTCATGTCTGTCGAATATGACGA | EcoRI/XhoI |
| (BL21(DE3)) | CGCCTCGAGTTAGATTATTCCACCCATTG | (pET28a) |
| ATG8 | CGCGCGGAATTCATGAATTTCCAATACAAAGC | EcoRI/XhoI |
| (BL21(DE3)) | CGCTCGAGTTAAGCACAGATCTGGC | (pET28a) |

T7=5’-TAATACGACTCACTATAGG-3’

>BtPEBP4

ATGGCTTCCGTCTCCGTAATGCTATCCATGGTTTTGCAGACGTATTCGGTGTCCGTGGTATCTATGGCACAT
TCGTTGCATTTGCTCGGTTTTCCCCCGAGGAAAGTGAGAAGCAGGAAAAACAAGGAAAACATGGAAAGAA
CGGATAGCCCGGAAAGTATGGAGATGGAAAGCACGAAAGGCACAGAAAGCACAAAGAAAACAAGACGCA
CGAAAAGCACAAAAACTAAAAGCACGAAAAGCAAAAAACCCAAAAGCACGAAAAGCACAAAAACCAAAAG
CACAAAAGCCAGAAGCACGAAAATCACAAAAACAAAAAAAACACCAACACCAAAAATCAAGAAAAGCACA
AGGAACACGAGGCGTCCGCGAATAAGTAAAAACACGGAAAGCACCAAAATTTCAAAAACCACTGAAGTTAT
TGGAAGCACAGAAATTTTAACAACCACAAGGCACGGAAAGACGAGGTCAAGACCTAAAAATATATCACCTT
TTGATGCAATTTTTAGAGAAAATGAAATAGTGCCACAGATCGTTAATAACACACCAATATACAAATGTGAGGT
TGAATATGGCAAGGAGAAAATACATTTTGGGAACAACATGACGGCAGATCAAATCGCAGAAAAACCAACGT
TAGTCAAGTGGCCTACGCAGAGTGGTGCTTTTTATACTCTTCTCATGTTTGGACCTGATGTCCCAACGCGG
GAGAAATCTTGTGAGATGATGTGGCGACATTGGGCAGTTGGGAATATCCCAAAGAATGACATCCATAAAGG
AGACACCTTAGTCGAATACGAAAATATCGAGCCTTTCTACAGGGCAGGTTTTCACAGGATATTGTTTTTTATT
TACAAACAGCCAGGAAGAAGGAGATTGAACTTCACATCAAGTAAAGGGGTTATTAAGAAAGGAGCGAAATG

GGAAGTGCGAGGAGTCTGGTATATTGAGCGTTTTCTCGCTAAATTCAATCTGACCGAACCAATAGCAGCGA
ACTTTTTCTTTCTTTATGATTTGAATCCTCATCCCGAAATTCCACAAGTTACTGTAGATCCACTGTCACTAATT
CGATGA
