# Supplementary material for: PEBP balances apoptosis and autophagy in whitefly upon arbovirus infection
Source: Nat Commun. 2022 Feb 11;13:846. doi: 10.1038/s41467-022-28500-8 (PMC8837789; doi:10.1038/s41467-022-28500-8)
Supplement: Supplementary file 3 — Reporting Summary [file 41467_2022_28500_MOESM3_ESM.pdf]

nature research

Corresponding author(s): Yucheng Sun

Last updated by author(s): Dec 4, 2021

Reporting Summary

Nature Research wishes to improve the reproducibility of the work that we publish. This form provides structure for consistency and transparency in reporting. For further information on Nature Research policies, see our Editorial Policies and the Editorial Policy Checklist.

Statistics

For all statistical analyses, confirm that the following items are present in the figure legend, table legend, main text, or Methods section.

n/a	Confirmed	
☐	☒	The exact sample size (*n*) for each experimental group/condition, given as a discrete number and unit of measurement
☐	☒	A statement on whether measurements were taken from distinct samples or whether the same sample was measured repeatedly
☐	☒	The statistical test(s) used AND whether they are one- or two-sided<br>*Only common tests should be described solely by name; describe more complex techniques in the Methods section.*
☒	☐	A description of all covariates tested
☐	☒	A description of any assumptions or corrections, such as tests of normality and adjustment for multiple comparisons
☐	☒	A full description of the statistical parameters including central tendency (e.g. means) or other basic estimates (e.g. regression coefficient) AND variation (e.g. standard deviation) or associated estimates of uncertainty (e.g. confidence intervals)
☐	☒	For null hypothesis testing, the test statistic (e.g. *F*, *t*, *r*) with confidence intervals, effect sizes, degrees of freedom and *P* value noted<br>*Give P values as exact values whenever suitable.*
☒	☐	For Bayesian analysis, information on the choice of priors and Markov chain Monte Carlo settings
☒	☐	For hierarchical and complex designs, identification of the appropriate level for tests and full reporting of outcomes
☒	☐	Estimates of effect sizes (e.g. Cohen's *d*, Pearson's *r*), indicating how they were calculated

Our web collection on statistics for biologists contains articles on many of the points above.

Software and code

Policy information about availability of computer code

Data collection	Image data were collected using ZEN software for Zeiss LSM710 microscopes. RT-qPCR data were collected using ABI QuantStudio 12K Flex Real-Time PCR System and QuantStudio 12K Flex Software.
Data analysis	Data were collated using Microsoft Excel and then was analyzed using SPSS 21 to derive p values and perform indicated statistical tests. Data was entered into GraphPad Prism 8 to derive standard deviations and generate the graphs displayed in all figures. Confocal images were analyzed by Image J.

For manuscripts utilizing custom algorithms or software that are central to the research but not yet described in published literature, software must be made available to editors and reviewers. We strongly encourage code deposition in a community repository (e.g. GitHub). See the Nature Research guidelines for submitting code & software for further information.

Data

Policy information about availability of data

All manuscripts must include a data availability statement. This statement should provide the following information, where applicable:

- Accession codes, unique identifiers, or web links for publicly available datasets
- A list of figures that have associated raw data
- A description of any restrictions on data availability

All data are available within the article, supplementary information or source data file. All protocols have been described in Methods or in the references therein. Sequence information was acquired from NCBI and GIGA database. LIR motif was analyzed in iLIR Autophagy Database. Plasmids used in the study are freely available upon request. No custom code or mathematical algorithm was used in this work. Plant cultivars, virus, and insect may be available upon request after publication and respective material transfer agreements are completed.

Field-specific reporting

Please select the one below that is the best fit for your research. If you are not sure, read the appropriate sections before making your selection.

☒ Life sciences ☐ Behavioural & social sciences ☐ Ecological, evolutionary & environmental sciences

For a reference copy of the document with all sections, see nature.com/documents/nr-reporting-summary-flat.pdf

Life sciences study design

All studies must disclose on these points even when the disclosure is negative.

Sample size	Sample size was determined based on practical considerations and was sufficient based on power calculations and differences observed between groups giving statistically significant analysis, and based on reproducibility between experiments.
Data exclusions	No data were excluded
Replication	All experiments were replicated three or more times. Each bio-replicate/treatment at least contained 30 insects in most cases. For measuring virus loading, each insect was collected and analyzed as an individual replicate.
Randomization	New emerged adult insect were randomly allocated to the experiments.
Blinding	Blinding was not relevant to any feeding treatment as each sample was known in order to set up relevant treatment. However, for immunofluorescence observation, investigators were blinded to group allocation during data collection.

Reporting for specific materials, systems and methods

We require information from authors about some types of materials, experimental systems and methods used in many studies. Here, indicate whether each material, system or method listed is relevant to your study. If you are not sure if a list item applies to your research, read the appropriate section before selecting a response.

Materials & experimental systems

n/a	Involved in the study	
☐	☒	Antibodies
☒	☐	Eukaryotic cell lines
☒	☐	Palaeontology and archaeology
☐	☒	Animals and other organisms
☒	☐	Human research participants
☒	☐	Clinical data
☒	☐	Dual use research of concern

Methods

n/a	Involved in the study	
☒	☐	ChIP-seq
☒	☐	Flow cytometry
☒	☐	MRI-based neuroimaging

Antibodies

Antibodies used	Primary antibodies: Rabbit anti-Raf1 (BBI, D220484, 1:1000), Rabbit anti-pRaf1 (BBI, D151522, 1:1000), Rabbit anti-pMEK (BBI, D155070, 1:1000), Rabbit anti-pERK (BBI, D151580, 1:1000), Rabbit anti-SQSTM1 (CST, 8025S, 1:1000), Rabbit anti-ATG8/GABARAP (CST, 13733S, 1:1000), Rabbit anti-GABARAP (Proteintech, 18723-1-AP, 1:10000), and Mouse anti-GAPDH (Proteintech, 60004-1-Ig, 1:10000), Mouse IgG anti-TYLCV CP (1:2500, Provided by Professor Xiaowei Wang and Jianxiang Wu, Zhejiang University), Rabbit anti-BtCaspase3b p10 (saved in our lab, 1:2000), Rabbit anti-BtPEBP4 pep1/pep2 (1:1000, prepared by BGI against synthetic peptides CPRKVRSRKNKENMES and TRHETTRSRPKNISPC), HRP-conjugated mouse IgG anti-GST (Proteintech, HRP-66001, 1:10000), HRP-conjugated mouse IgG anti-His (Proteintech, HRP-66005, 1:10000).<br>Secondary antibodies: Goat anti-mouse IgG (Abcam, ab6789, 1:10000), Goat anti-rabbit IgG (Abcam, ab6721, 1:10000), Goat anti-mouse IgG conjugate Alexa 488 (Abcam, ab150113, 1:1000), Goat anti-rabbit IgG conjugate Alexa 555 (Abcam, ab150078, 1:1000), Goat anti-rabbit IgG conjugate Alexa 647 (Abcam, ab150079, 1:1000), Goat anti-rabbit IgG F(ab) fragment conjugate Texas Red (Abcam, ab7052, 1:1000).
Validation	Commercial primary antibodies were validated by manufacturers. Mouse IgG anti-TYLCV CP antibody was validated by our previous study (DOI: https://doi.org/10.7554/eLife.56168). The new prepared Rabbit anti-BtPEBP4 pep1/pep2 antibodies were validated by immunoblotting and immunofluorescence with whitefly lysate in this study.

Animals and other organisms

Policy information about studies involving animals; ARRIVE guidelines recommended for reporting animal research

Laboratory animals	Beimisa tabaci (Mediterranean group) was maintained by our laboratory, new emerged adult insects (7 days) with mixed sex were used for all experiments.
Wild animals	The study did not involve wild animals.
Field-collected samples	This study did not use field-collected samples.
Ethics oversight	These studies were performed at the Institute of Zoology, Chinese Academy of Sciences (Beijing, China), and no ethical guidance was required for our work with Bemisa tabaci according to the law of China.

Note that full information on the approval of the study protocol must also be provided in the manuscript.
