# Supplementary figures and images for: PEBP balances apoptosis and autophagy in whitefly upon arbovirus infection (part 2 of 2)
Source: Nat Commun. 2022 Feb 11;13:846. doi: 10.1038/s41467-022-28500-8 (PMC8837789; doi:10.1038/s41467-022-28500-8)

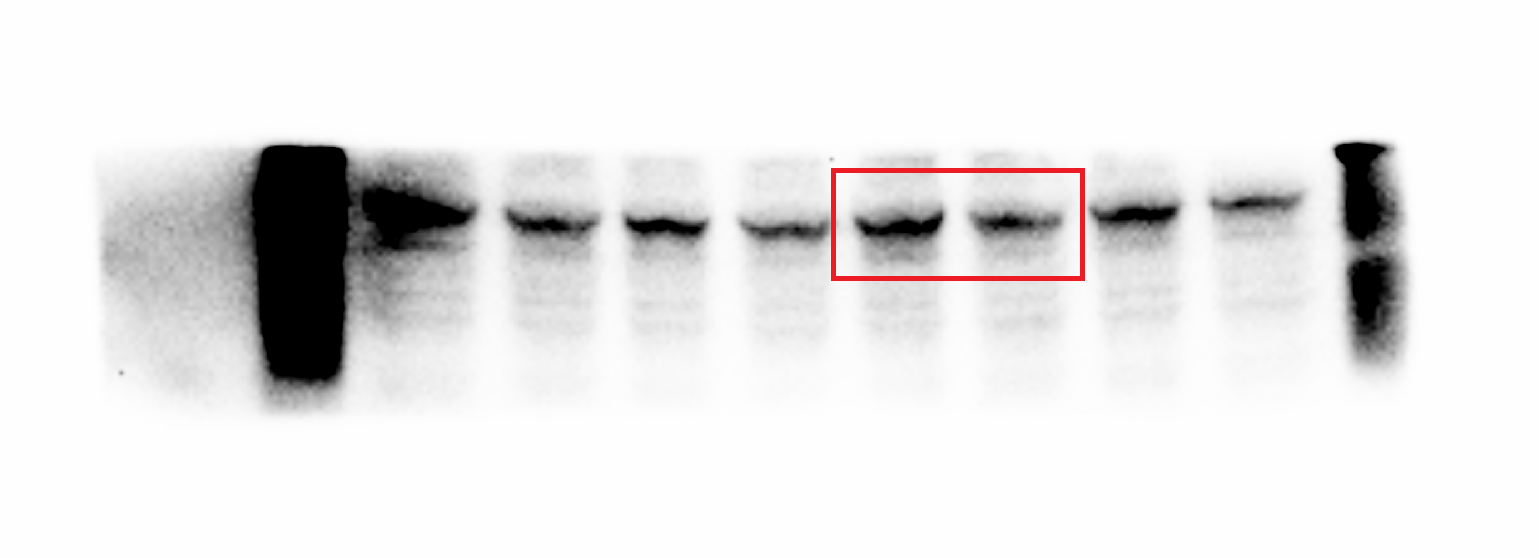

Supplement: Supplementary file 4 — Source Data [file 41467_2022_28500_MOESM4_ESM.zip › Source data/Fig3 F/CASP3.2.tif]

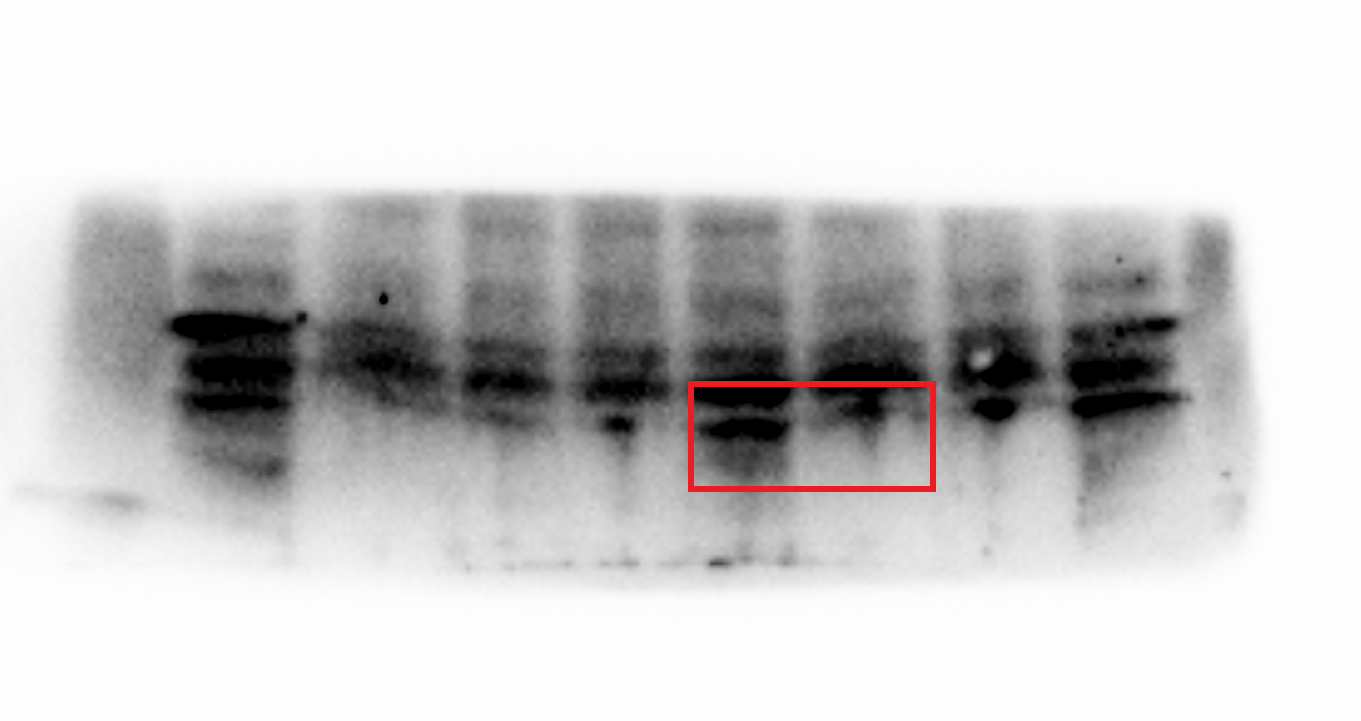

Supplement: Supplementary file 4 — Source Data [file 41467_2022_28500_MOESM4_ESM.zip › Source data/Fig3 F/cCasp3.tif]

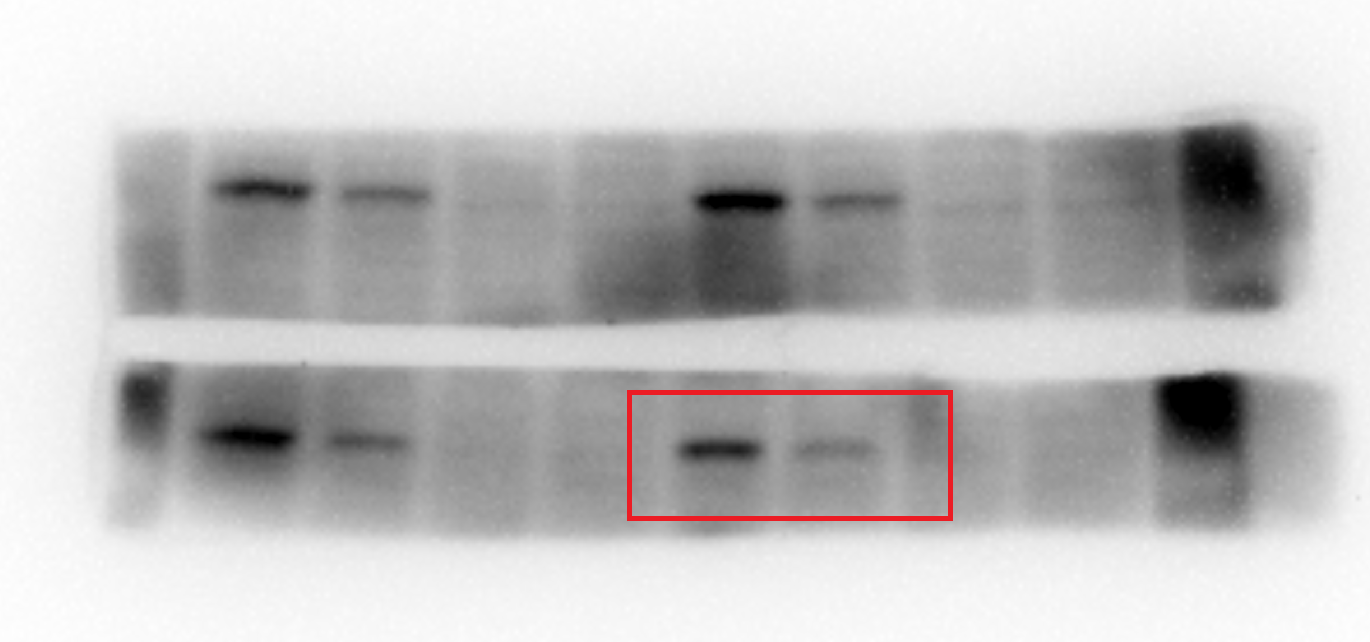

Supplement: Supplementary file 4 — Source Data [file 41467_2022_28500_MOESM4_ESM.zip › Source data/Fig3 F/CP.tif]

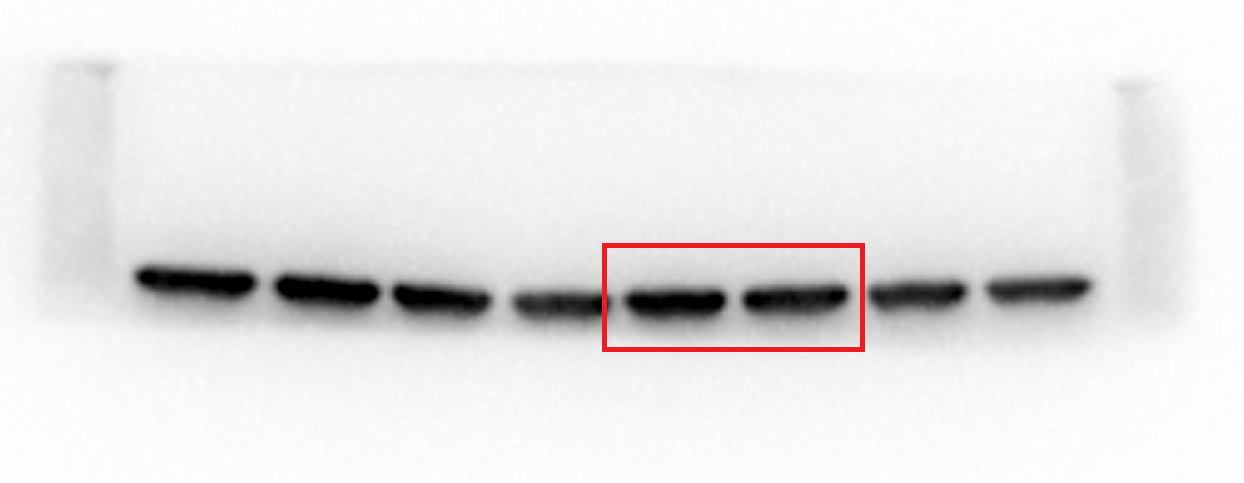

Supplement: Supplementary file 4 — Source Data [file 41467_2022_28500_MOESM4_ESM.zip › Source data/Fig3 F/GAPDH.tif]

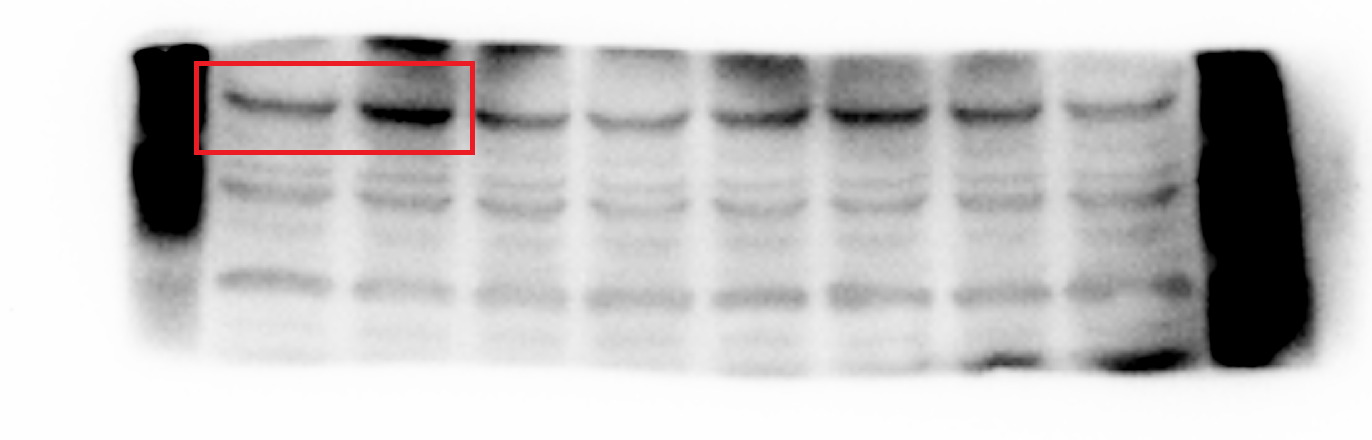

Supplement: Supplementary file 4 — Source Data [file 41467_2022_28500_MOESM4_ESM.zip › Source data/Fig3 F/PEBP4.tif]

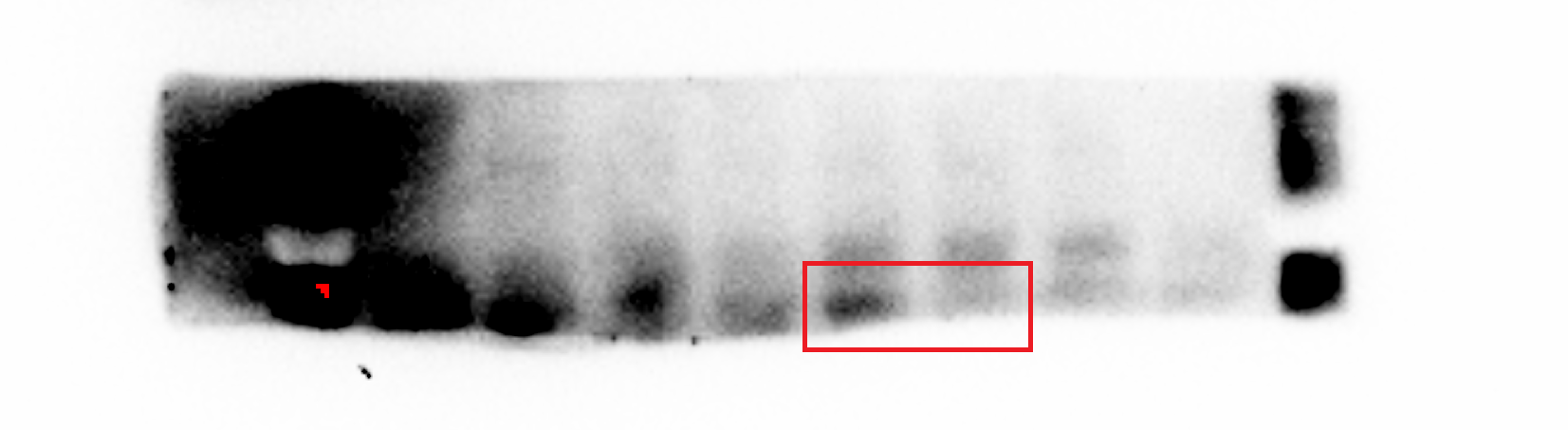

Supplement: Supplementary file 4 — Source Data [file 41467_2022_28500_MOESM4_ESM.zip › Source data/Fig3 F/SQSTM1.tif]

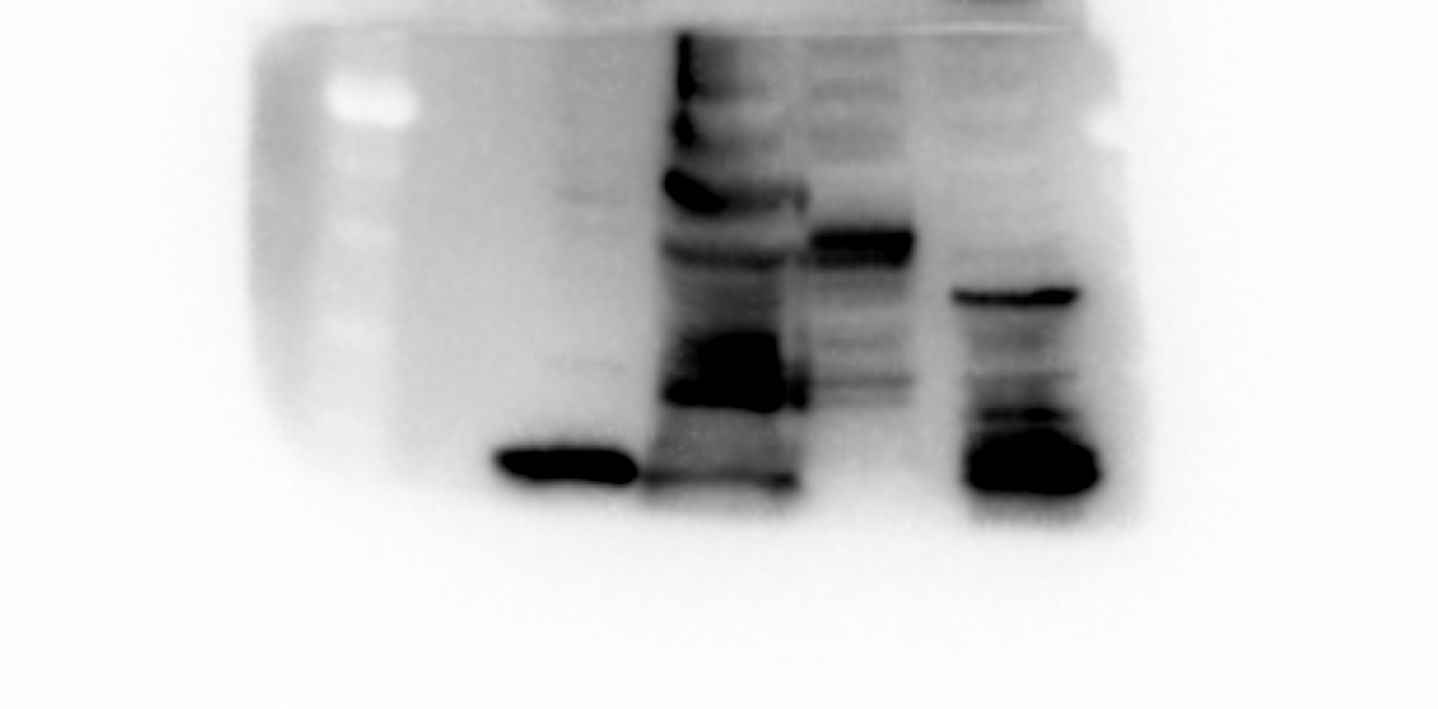

Supplement: Supplementary file 4 — Source Data [file 41467_2022_28500_MOESM4_ESM.zip › Source data/Fig4 A/input-GST.tif]

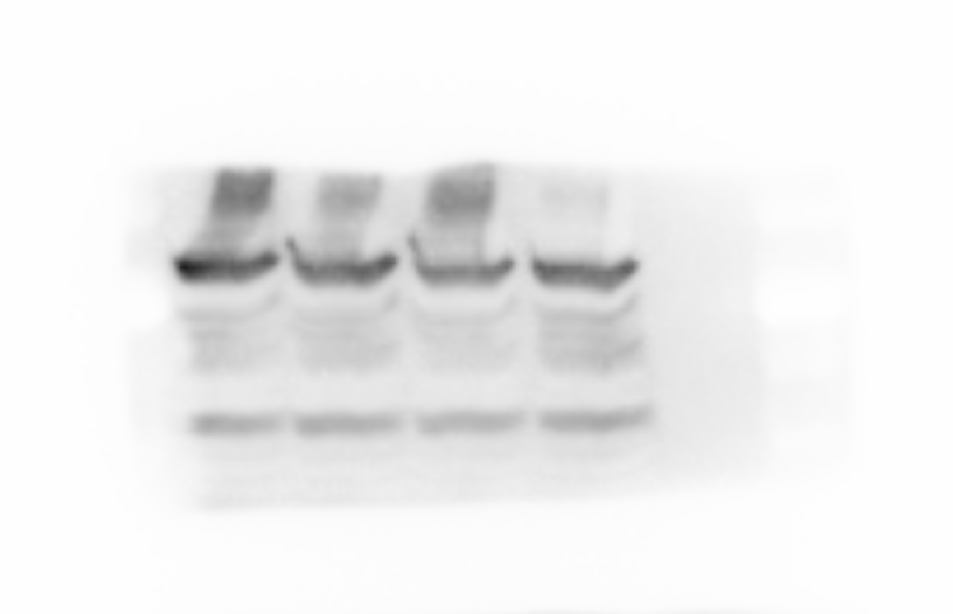

Supplement: Supplementary file 4 — Source Data [file 41467_2022_28500_MOESM4_ESM.zip › Source data/Fig4 A/input-His.tif]

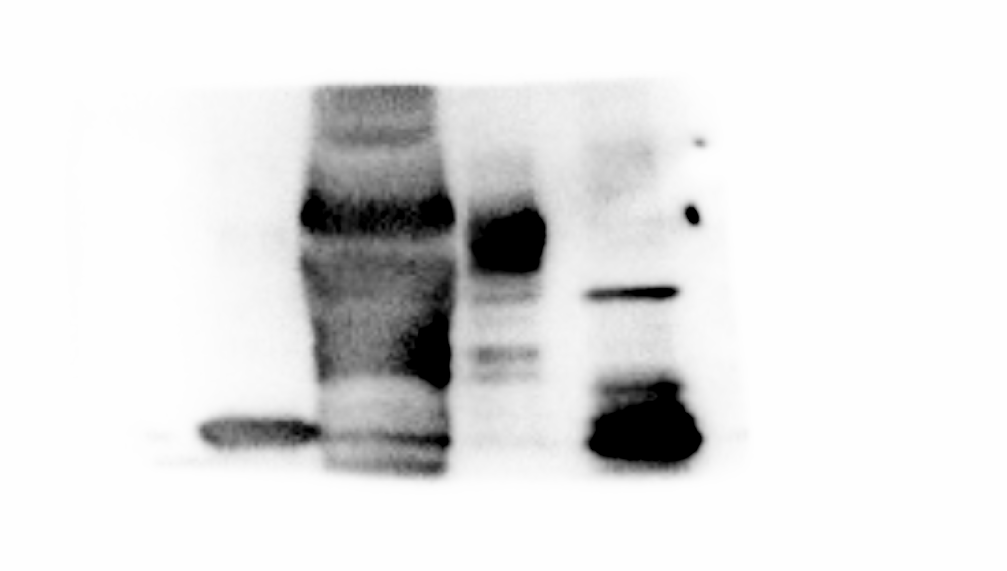

Supplement: Supplementary file 4 — Source Data [file 41467_2022_28500_MOESM4_ESM.zip › Source data/Fig4 A/pd-GST.tif]

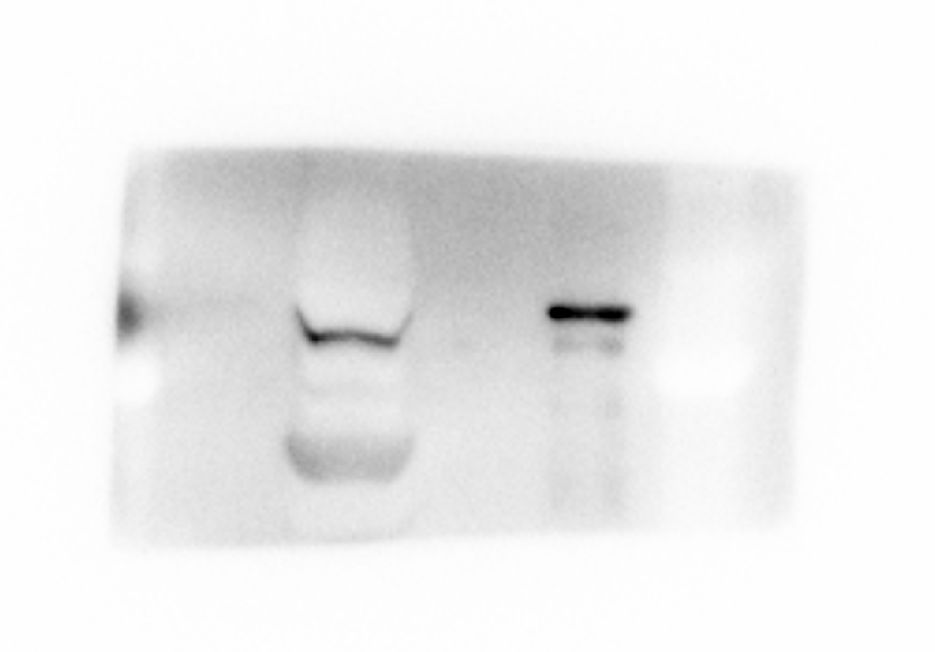

Supplement: Supplementary file 4 — Source Data [file 41467_2022_28500_MOESM4_ESM.zip › Source data/Fig4 A/pd-His.tif]

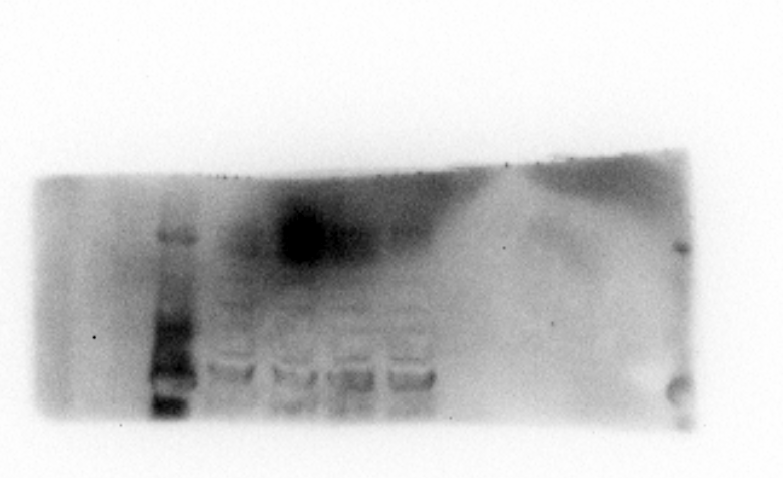

Supplement: Supplementary file 4 — Source Data [file 41467_2022_28500_MOESM4_ESM.zip › Source data/Fig4 B/input-Raf1.tif]

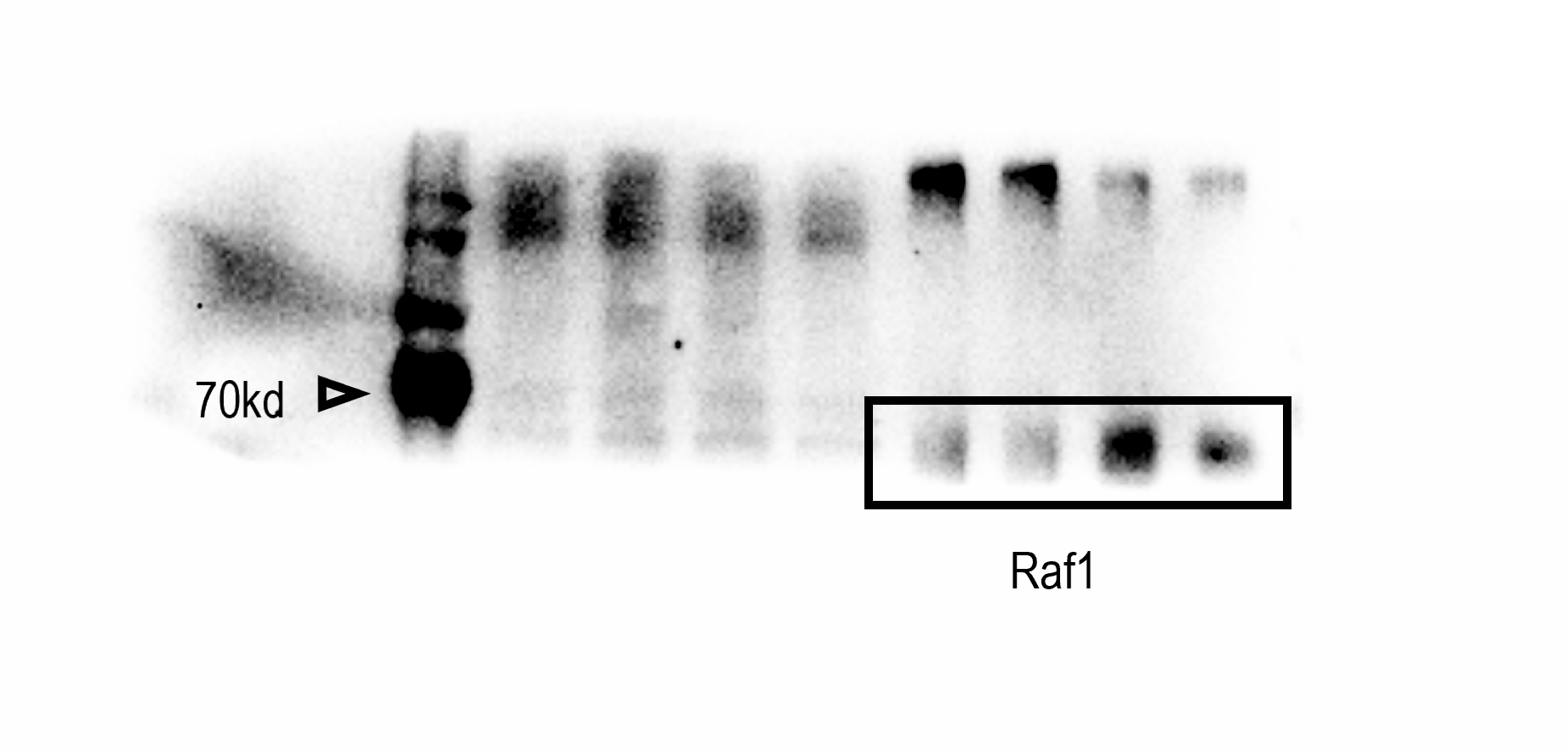

Supplement: Supplementary file 4 — Source Data [file 41467_2022_28500_MOESM4_ESM.zip › Source data/Fig4 B/pd-Raf1.tif]

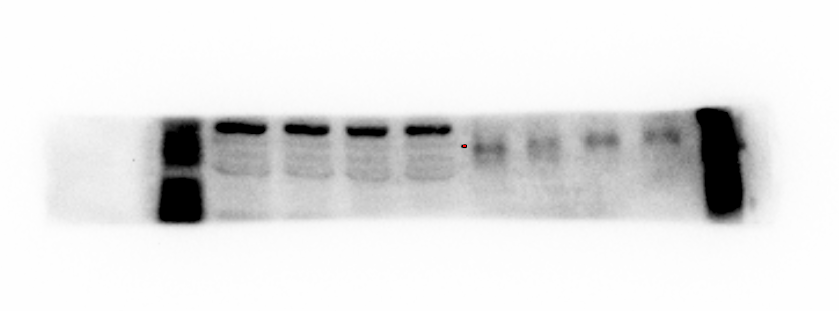

Supplement: Supplementary file 4 — Source Data [file 41467_2022_28500_MOESM4_ESM.zip › Source data/Fig4 B/PEBP.tif]

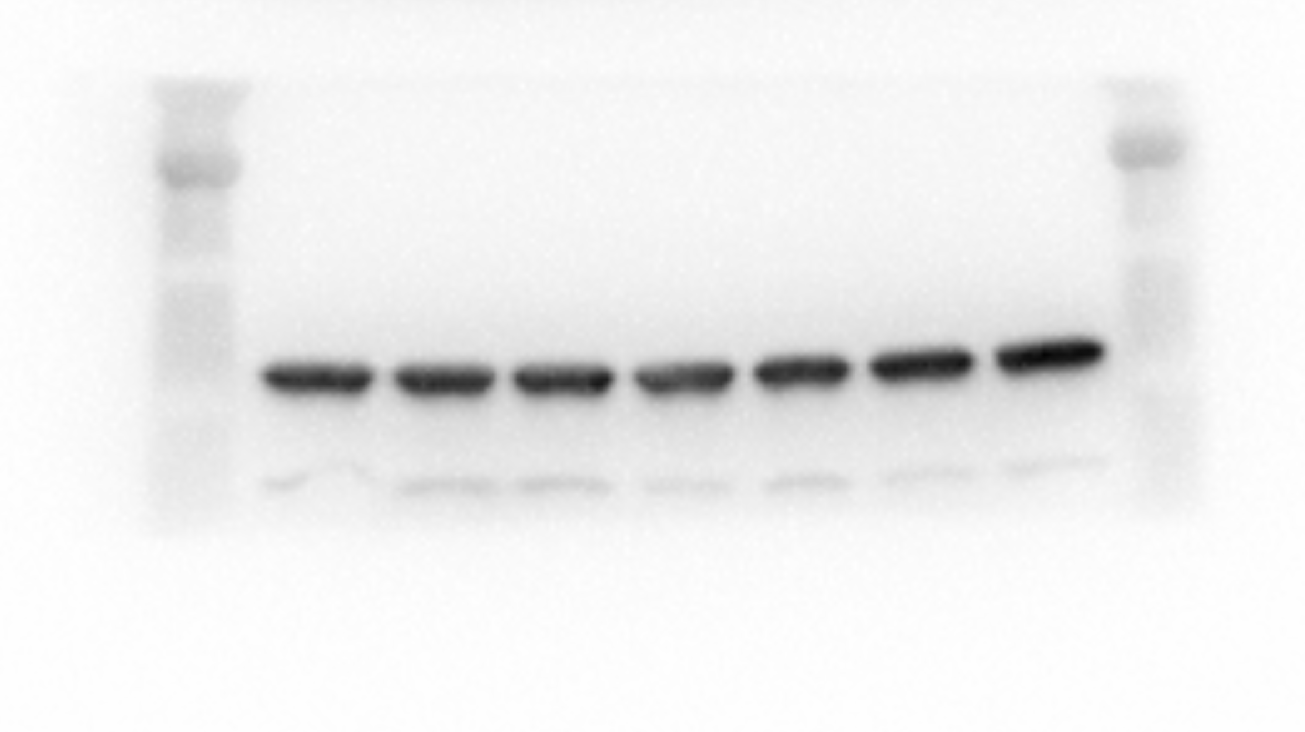

Supplement: Supplementary file 4 — Source Data [file 41467_2022_28500_MOESM4_ESM.zip › Source data/Fig4 C/GAPDH.tif]

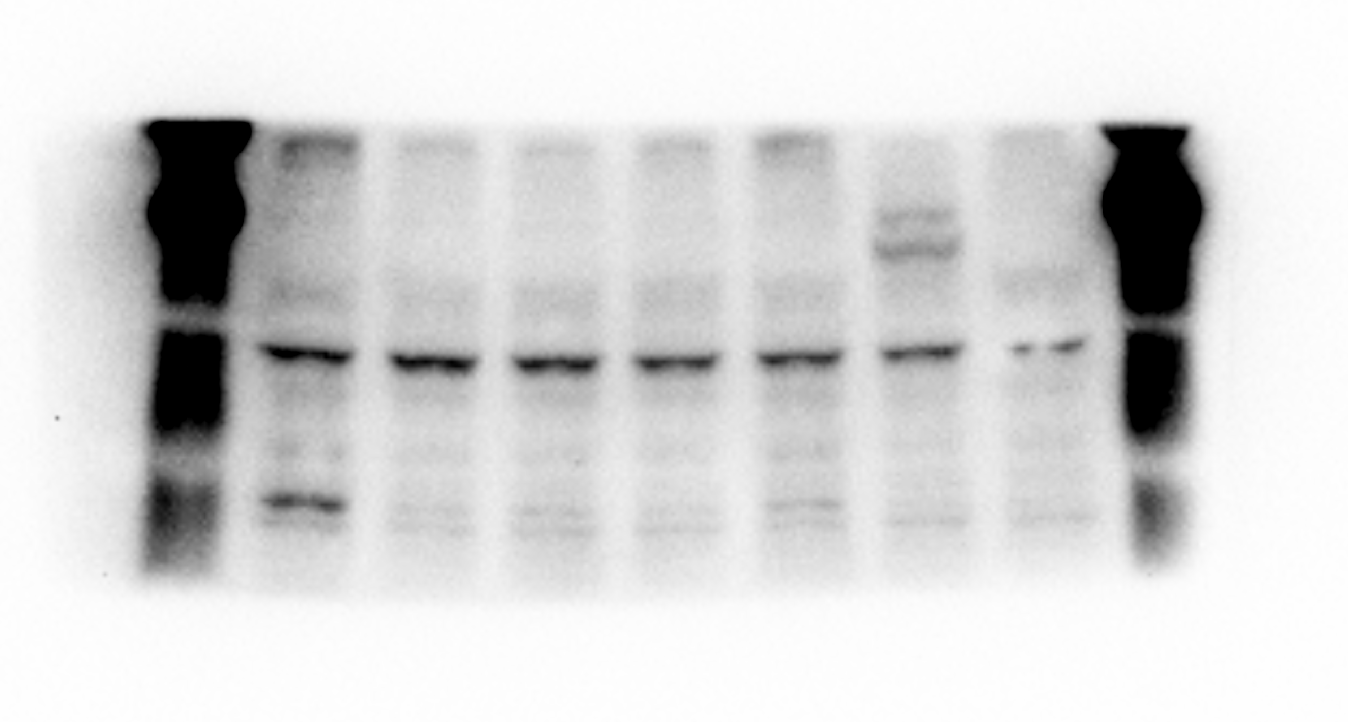

Supplement: Supplementary file 4 — Source Data [file 41467_2022_28500_MOESM4_ESM.zip › Source data/Fig4 C/pERK.tif]

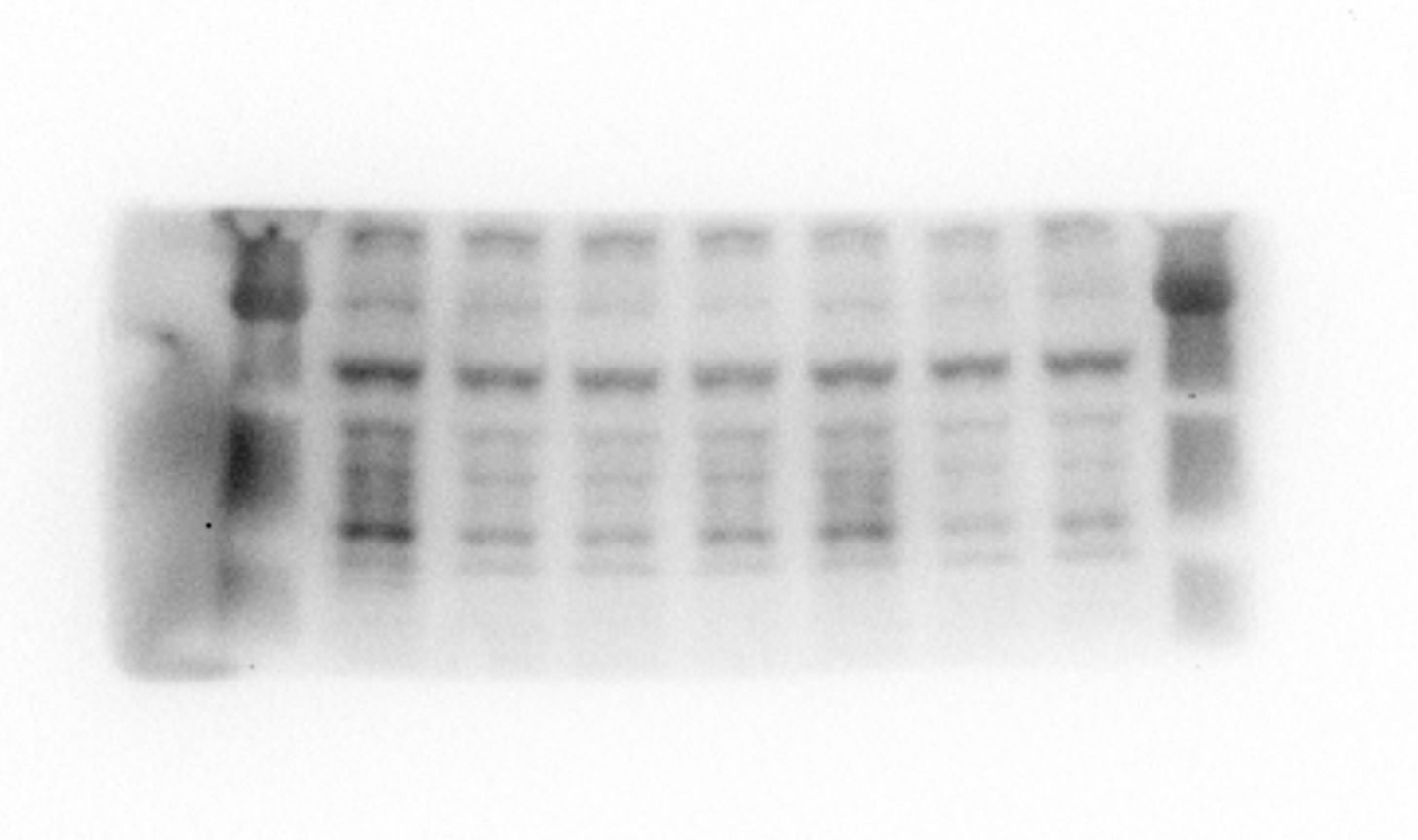

Supplement: Supplementary file 4 — Source Data [file 41467_2022_28500_MOESM4_ESM.zip › Source data/Fig4 C/pMEK.tif]

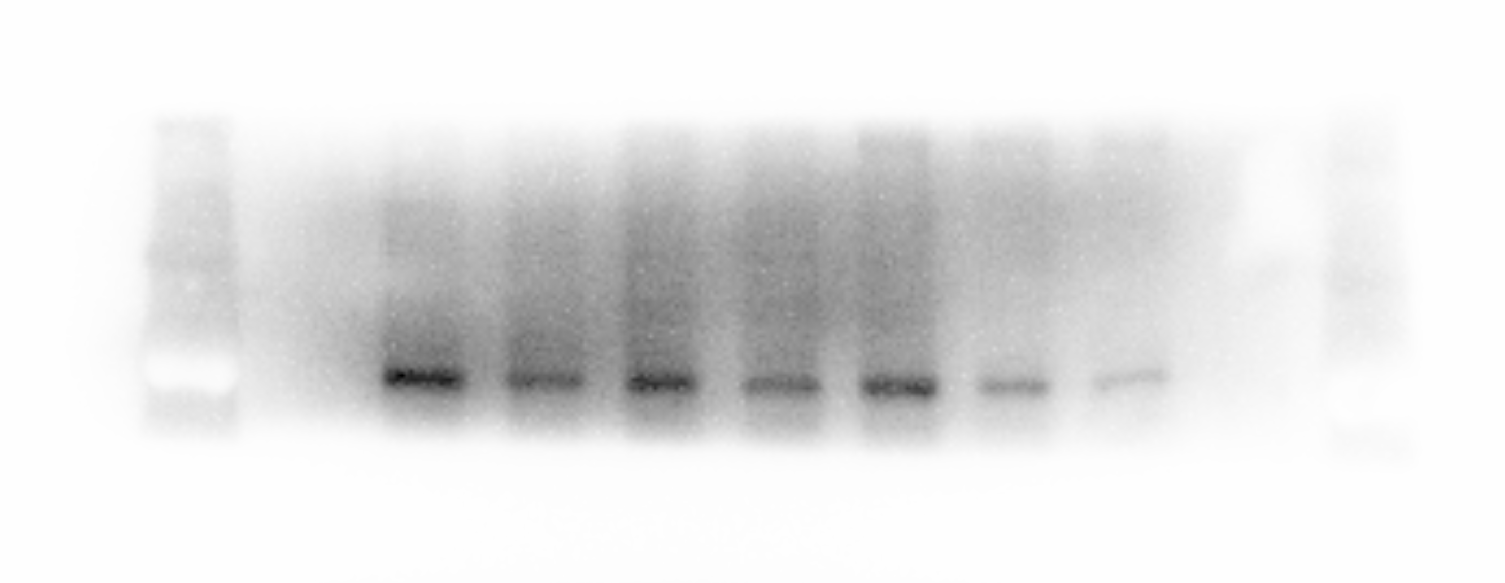

Supplement: Supplementary file 4 — Source Data [file 41467_2022_28500_MOESM4_ESM.zip › Source data/Fig4 C/pRaf1.tif]

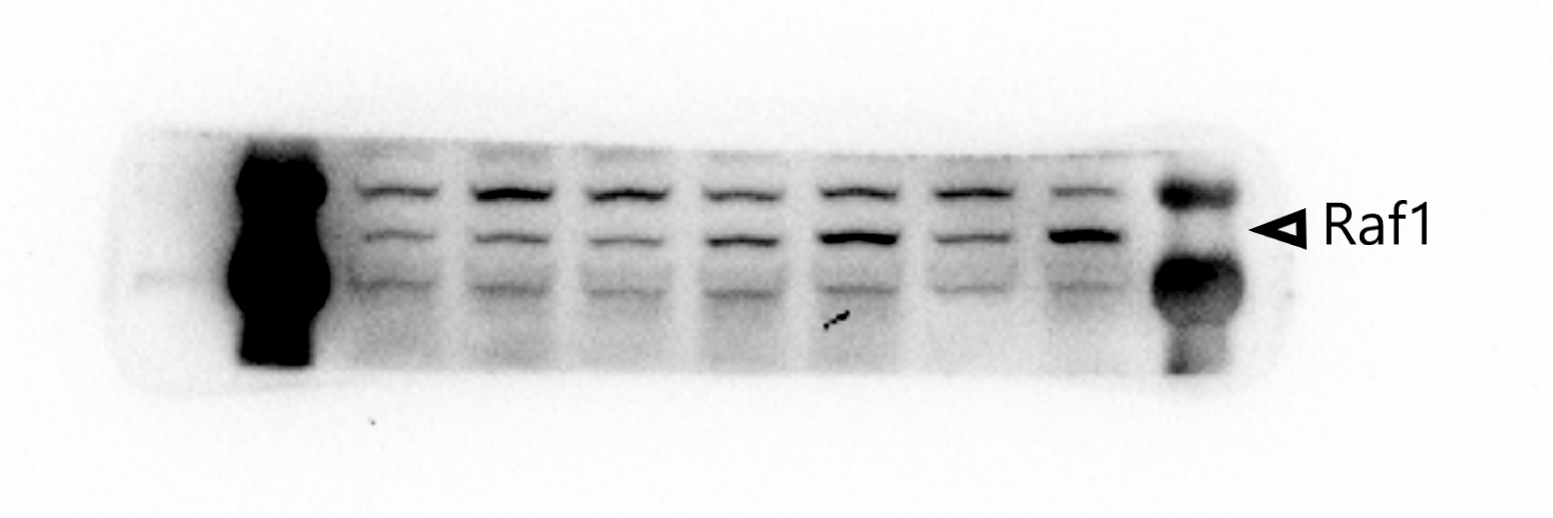

Supplement: Supplementary file 4 — Source Data [file 41467_2022_28500_MOESM4_ESM.zip › Source data/Fig4 C/Raf1.tif]

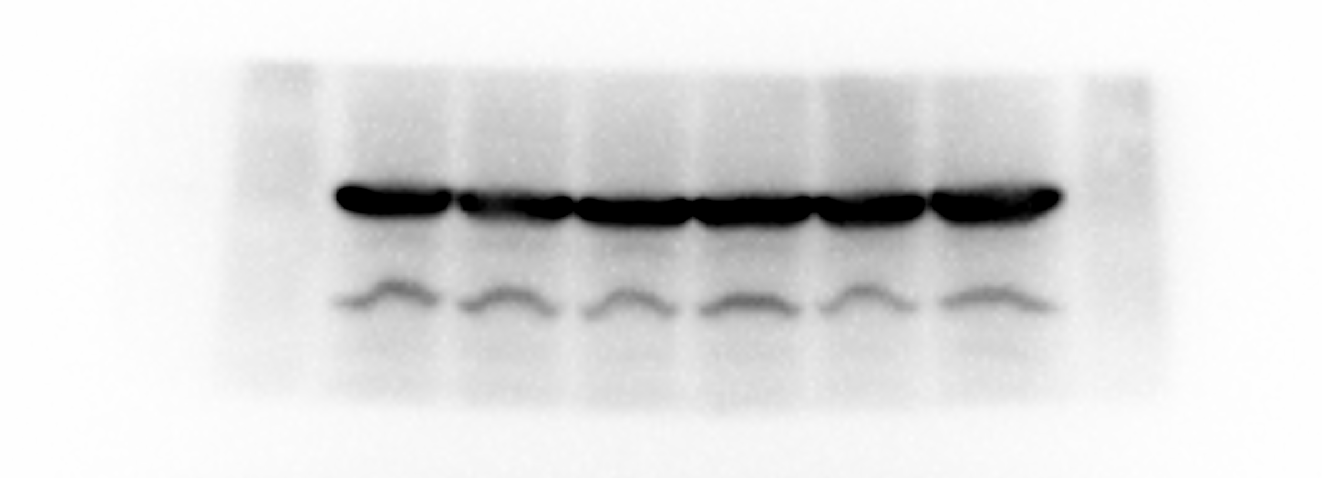

Supplement: Supplementary file 4 — Source Data [file 41467_2022_28500_MOESM4_ESM.zip › Source data/Fig4 D/GAPDH.tif]

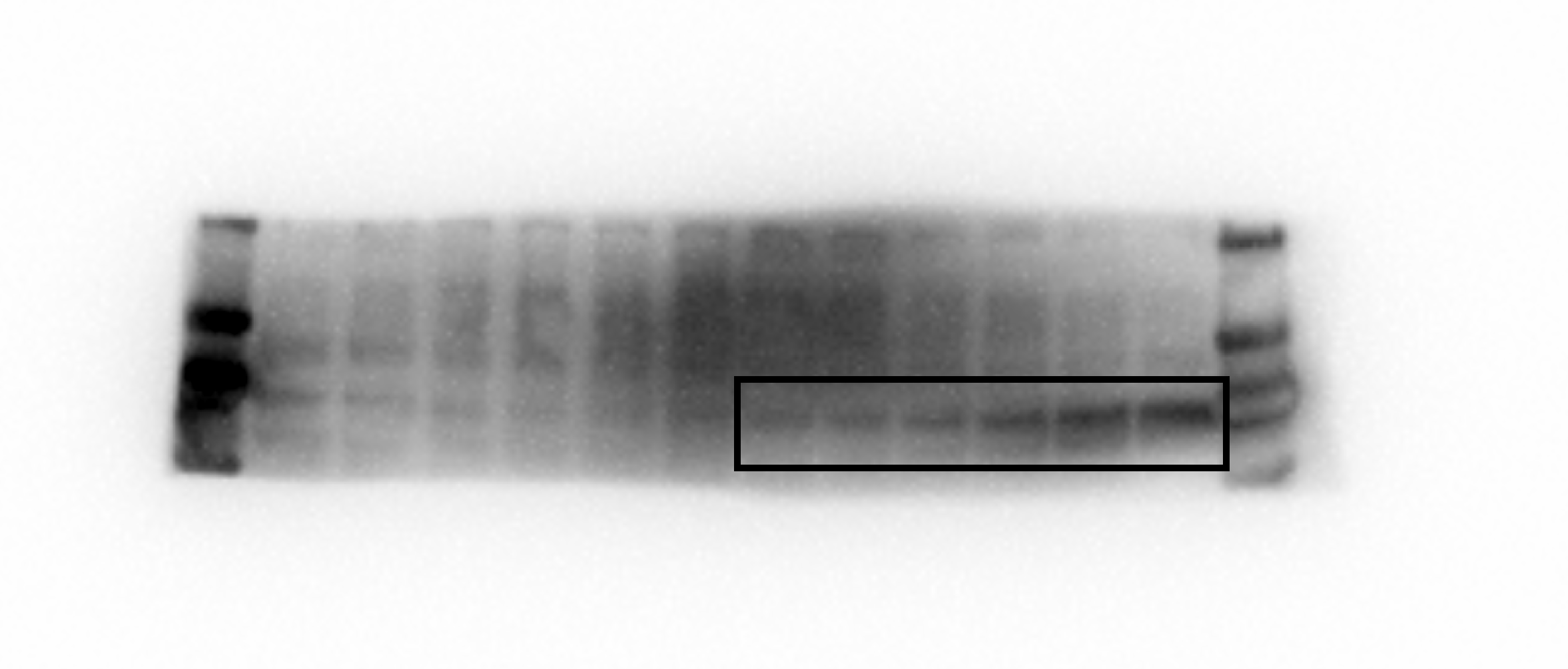

Supplement: Supplementary file 4 — Source Data [file 41467_2022_28500_MOESM4_ESM.zip › Source data/Fig4 D/GST-PEBP4.tif]

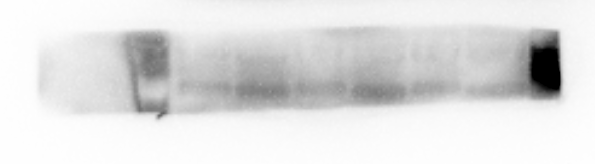

Supplement: Supplementary file 4 — Source Data [file 41467_2022_28500_MOESM4_ESM.zip › Source data/Fig4 D/PEBP.tif]

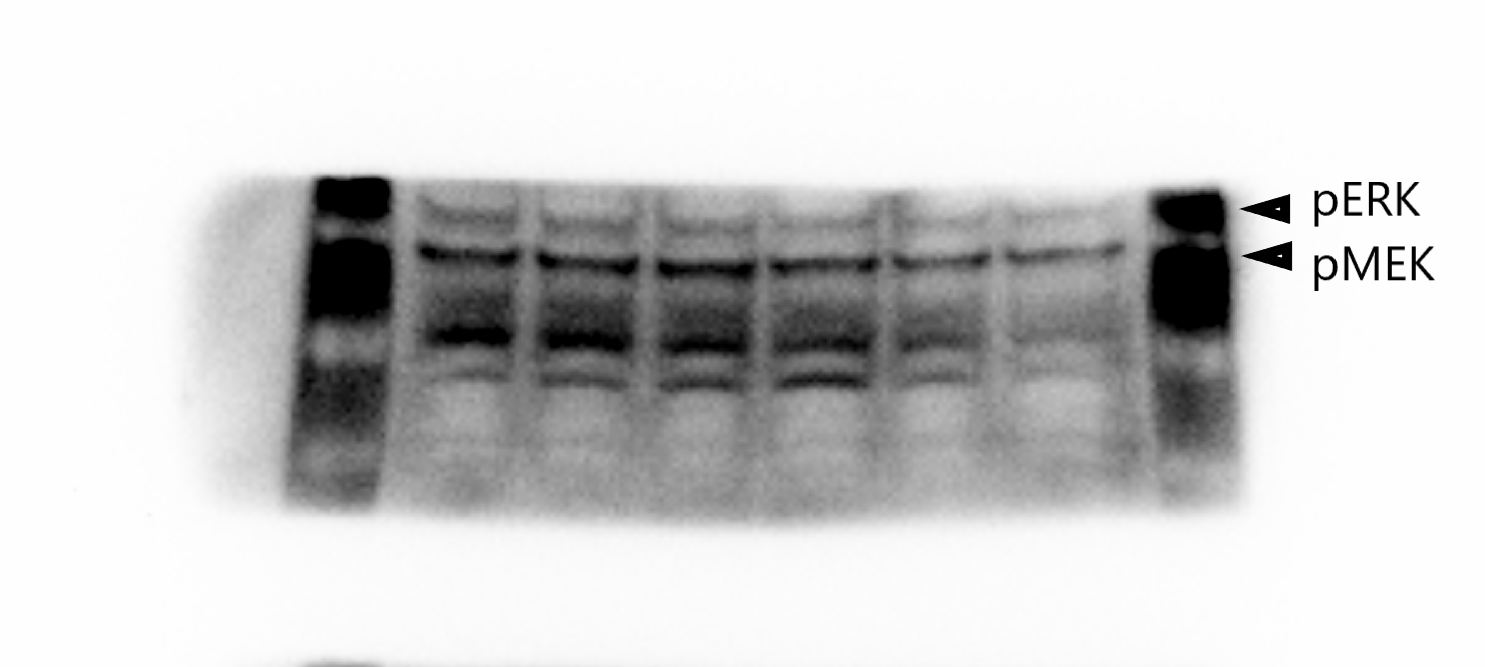

Supplement: Supplementary file 4 — Source Data [file 41467_2022_28500_MOESM4_ESM.zip › Source data/Fig4 D/pMEK-pERK.tif]

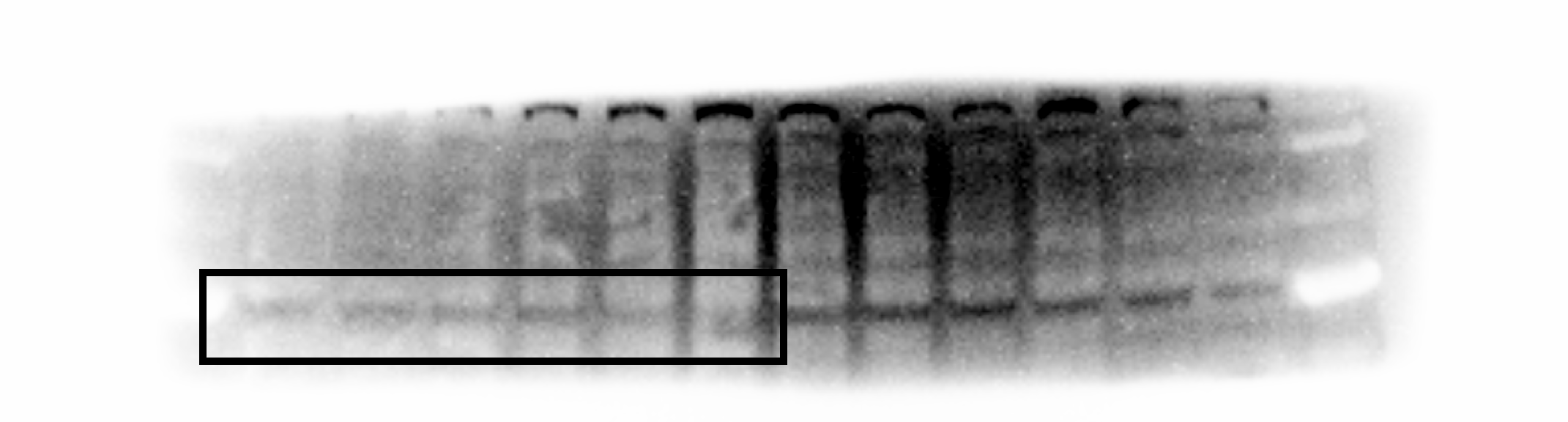

Supplement: Supplementary file 4 — Source Data [file 41467_2022_28500_MOESM4_ESM.zip › Source data/Fig4 D/pRaf1.tif]

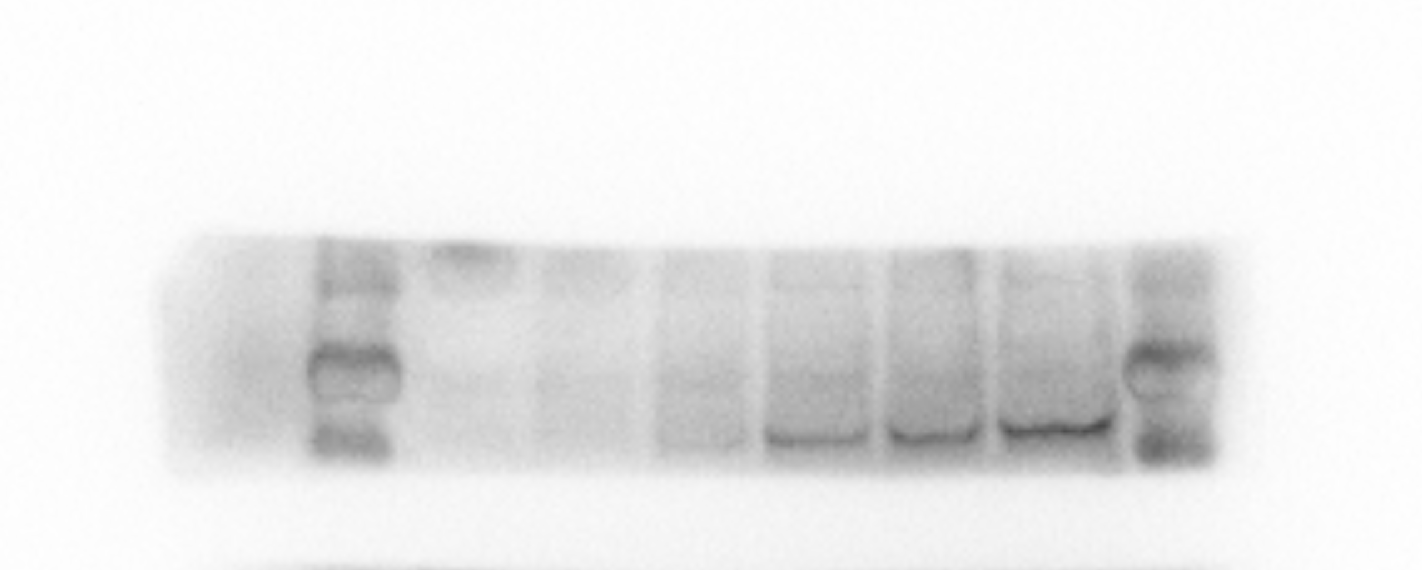

Supplement: Supplementary file 4 — Source Data [file 41467_2022_28500_MOESM4_ESM.zip › Source data/Fig4 D/Raf1.tif]

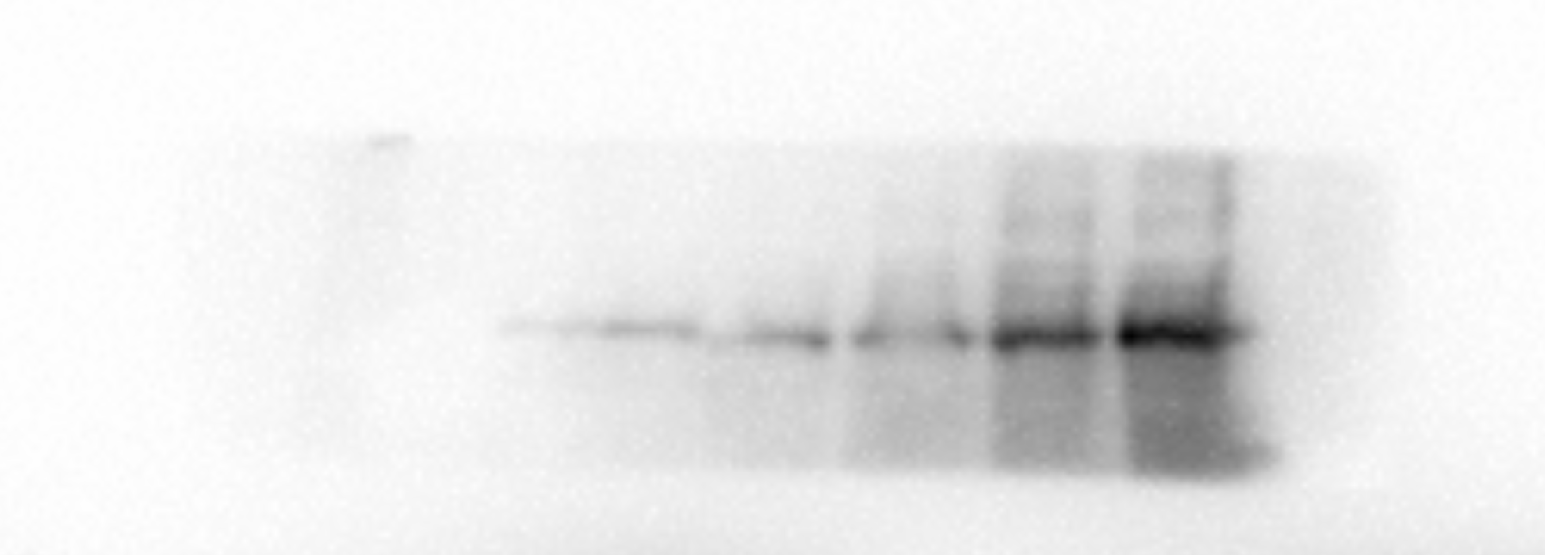

Supplement: Supplementary file 4 — Source Data [file 41467_2022_28500_MOESM4_ESM.zip › Source data/Fig4 E/CP.tif]

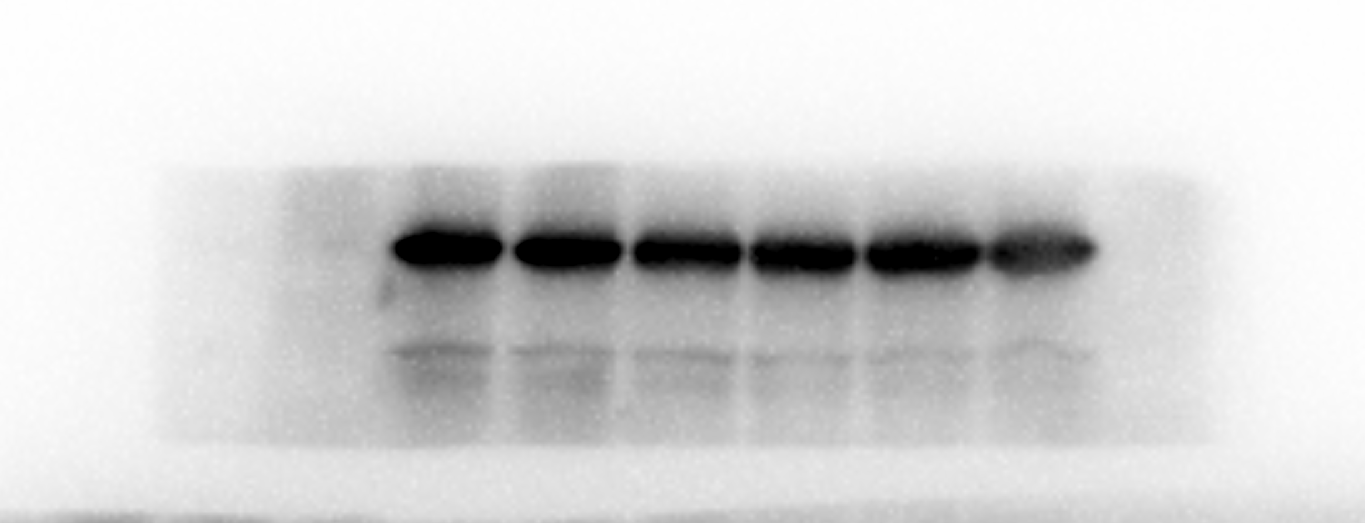

Supplement: Supplementary file 4 — Source Data [file 41467_2022_28500_MOESM4_ESM.zip › Source data/Fig4 E/GAPDH.tif]

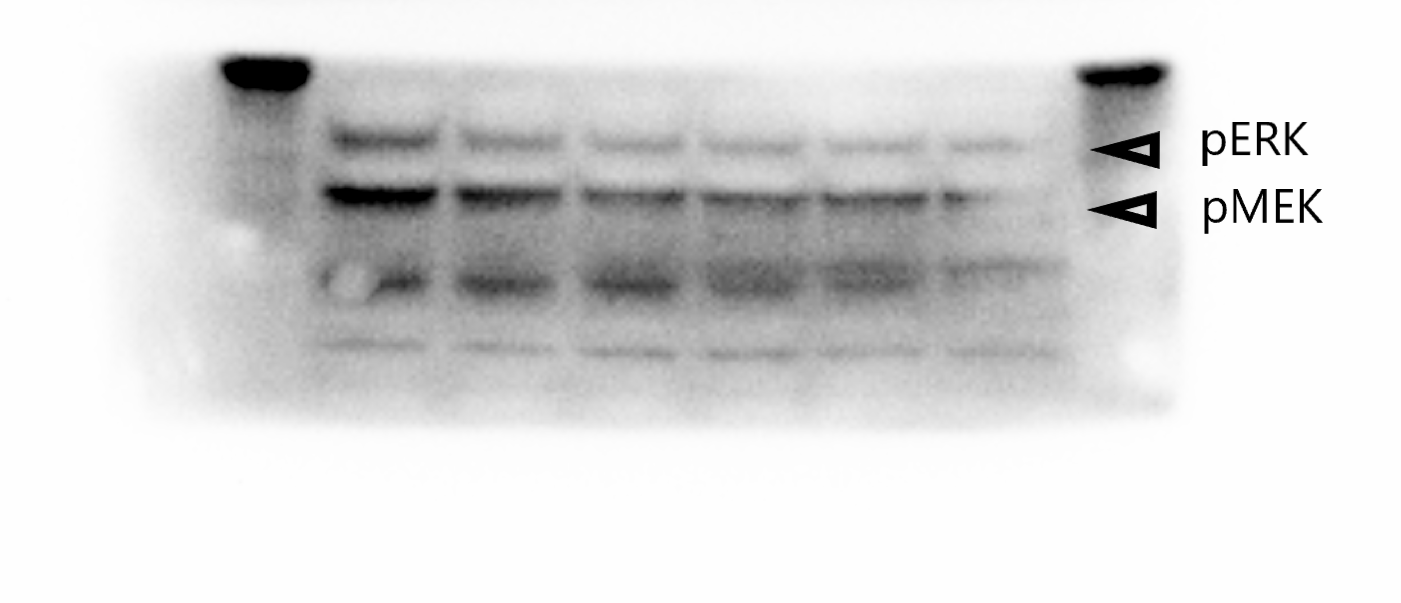

Supplement: Supplementary file 4 — Source Data [file 41467_2022_28500_MOESM4_ESM.zip › Source data/Fig4 E/pMEK&pERKE.tif]

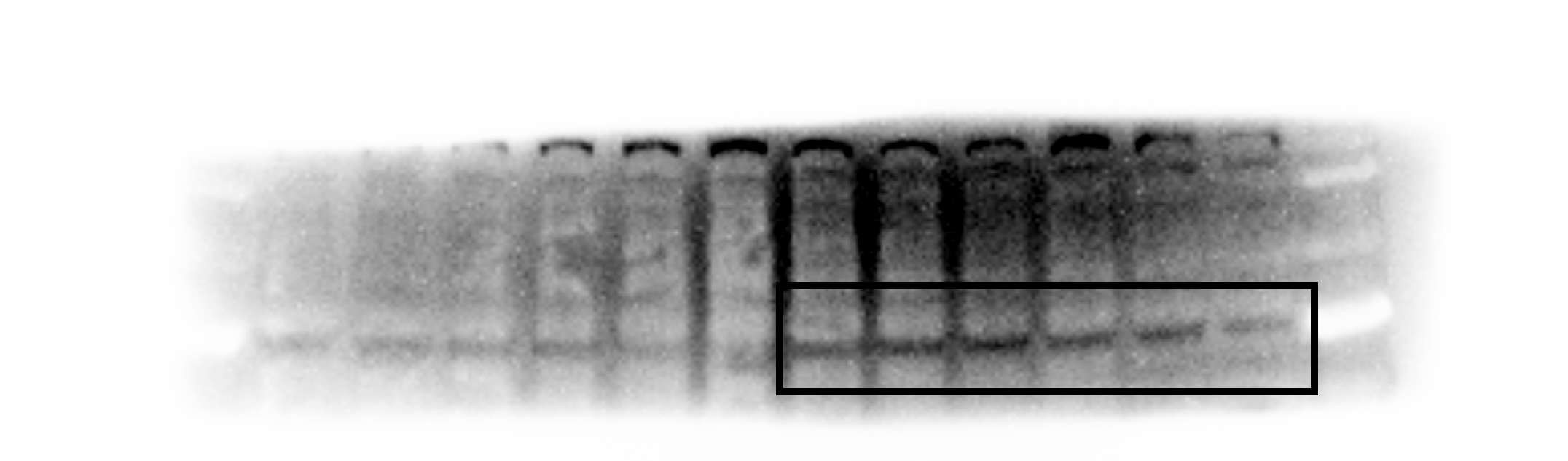

Supplement: Supplementary file 4 — Source Data [file 41467_2022_28500_MOESM4_ESM.zip › Source data/Fig4 E/pRaf1.tif]

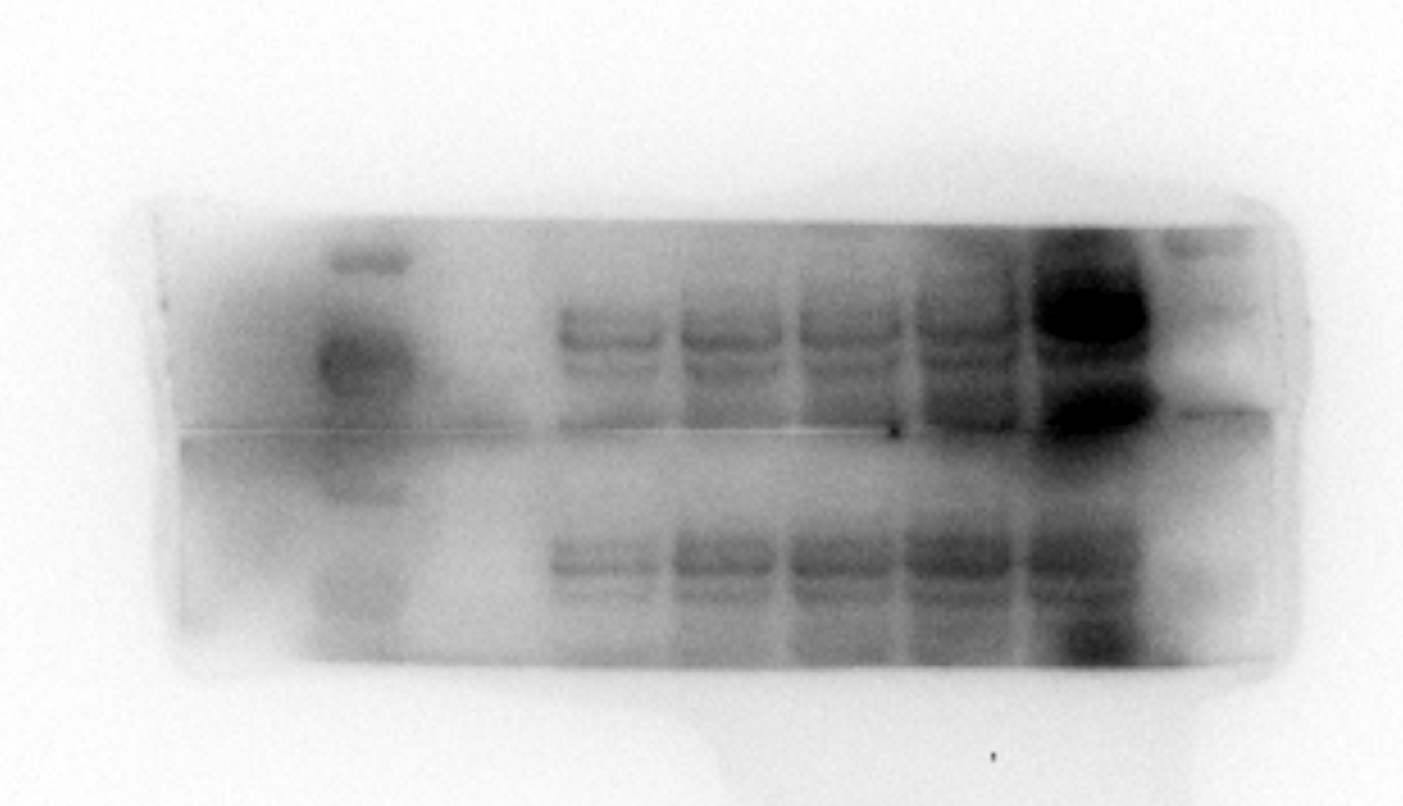

Supplement: Supplementary file 4 — Source Data [file 41467_2022_28500_MOESM4_ESM.zip › Source data/Fig4 E/Raf1.tif]

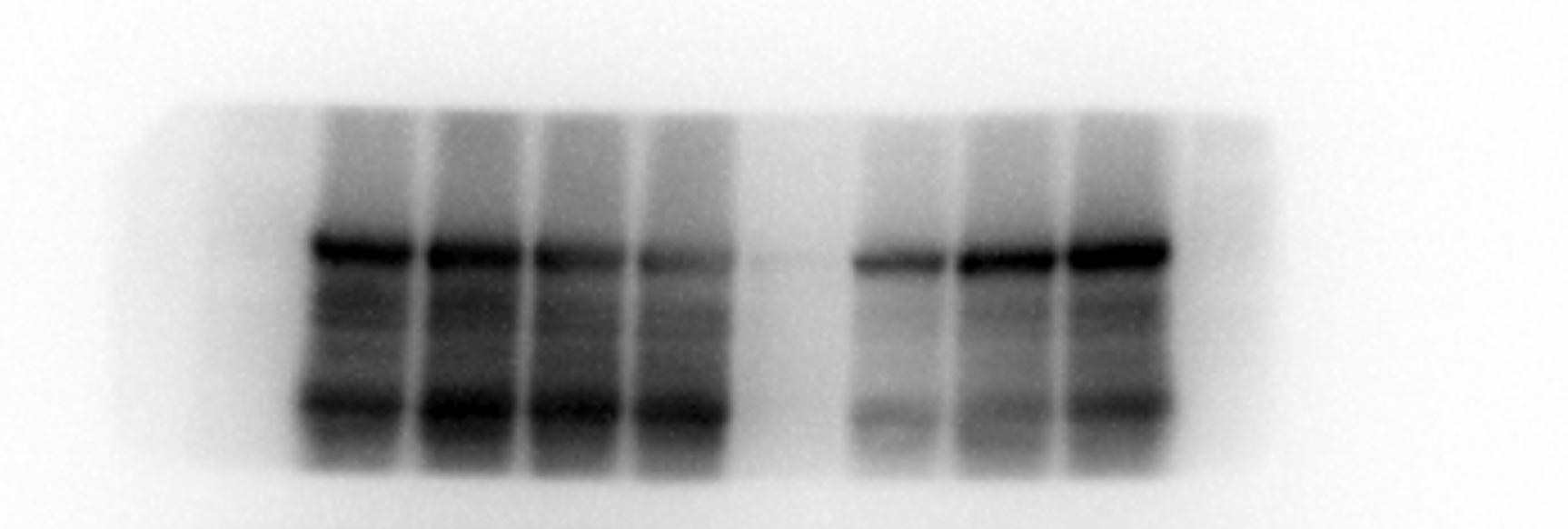

Supplement: Supplementary file 4 — Source Data [file 41467_2022_28500_MOESM4_ESM.zip › Source data/Fig4 F/input-CP.tif]

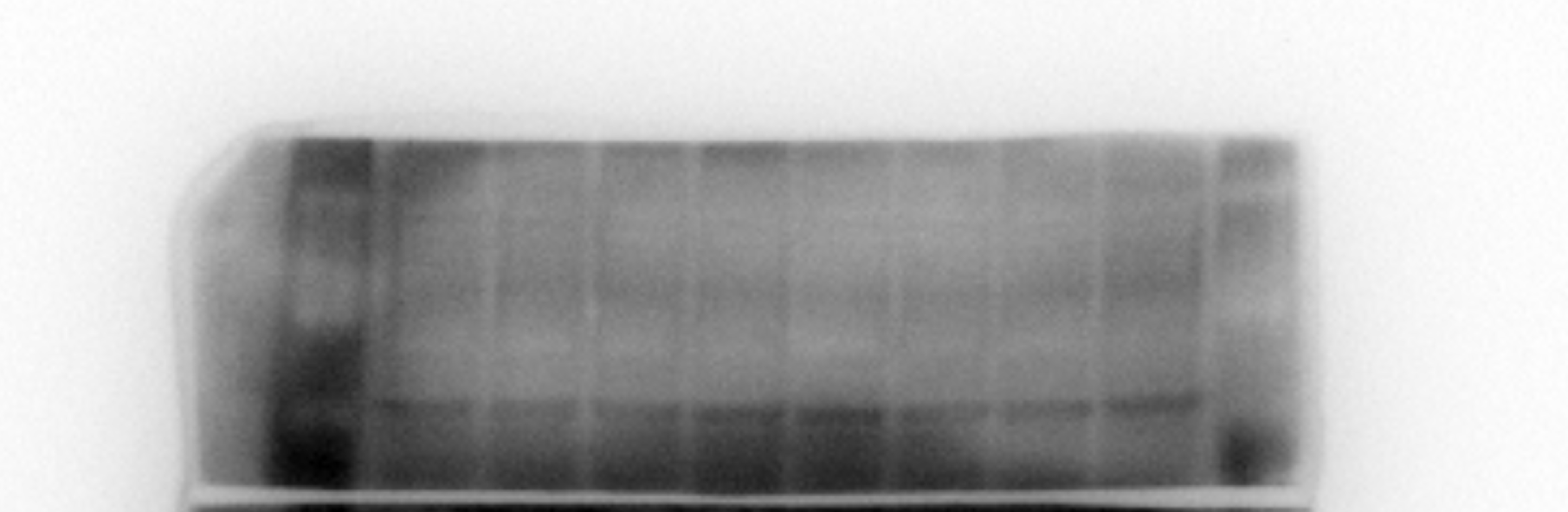

Supplement: Supplementary file 4 — Source Data [file 41467_2022_28500_MOESM4_ESM.zip › Source data/Fig4 F/input-PEBP4.tif]

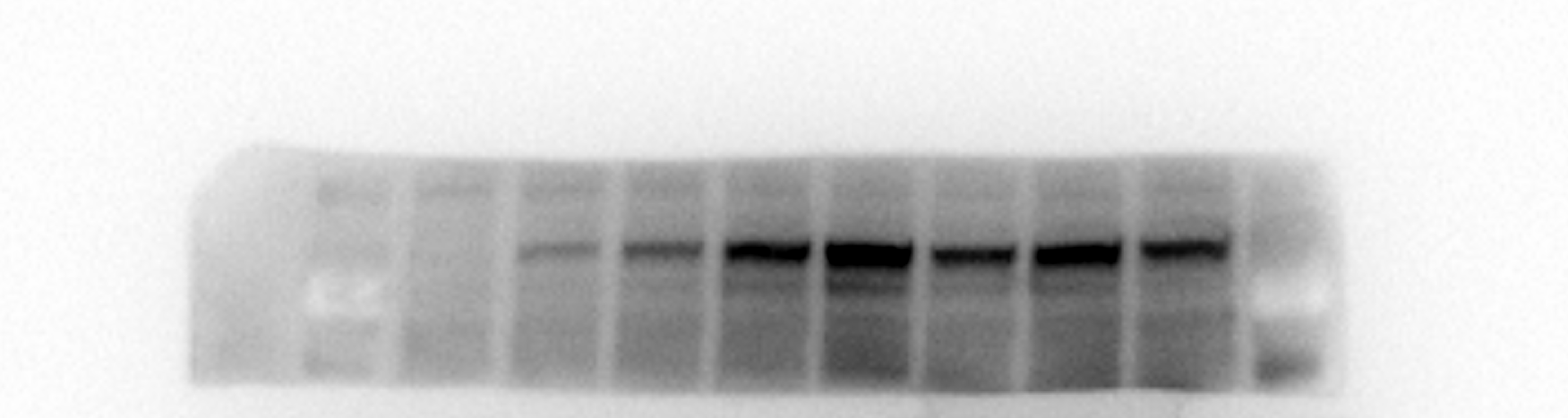

Supplement: Supplementary file 4 — Source Data [file 41467_2022_28500_MOESM4_ESM.zip › Source data/Fig4 F/input-Raf.tif]

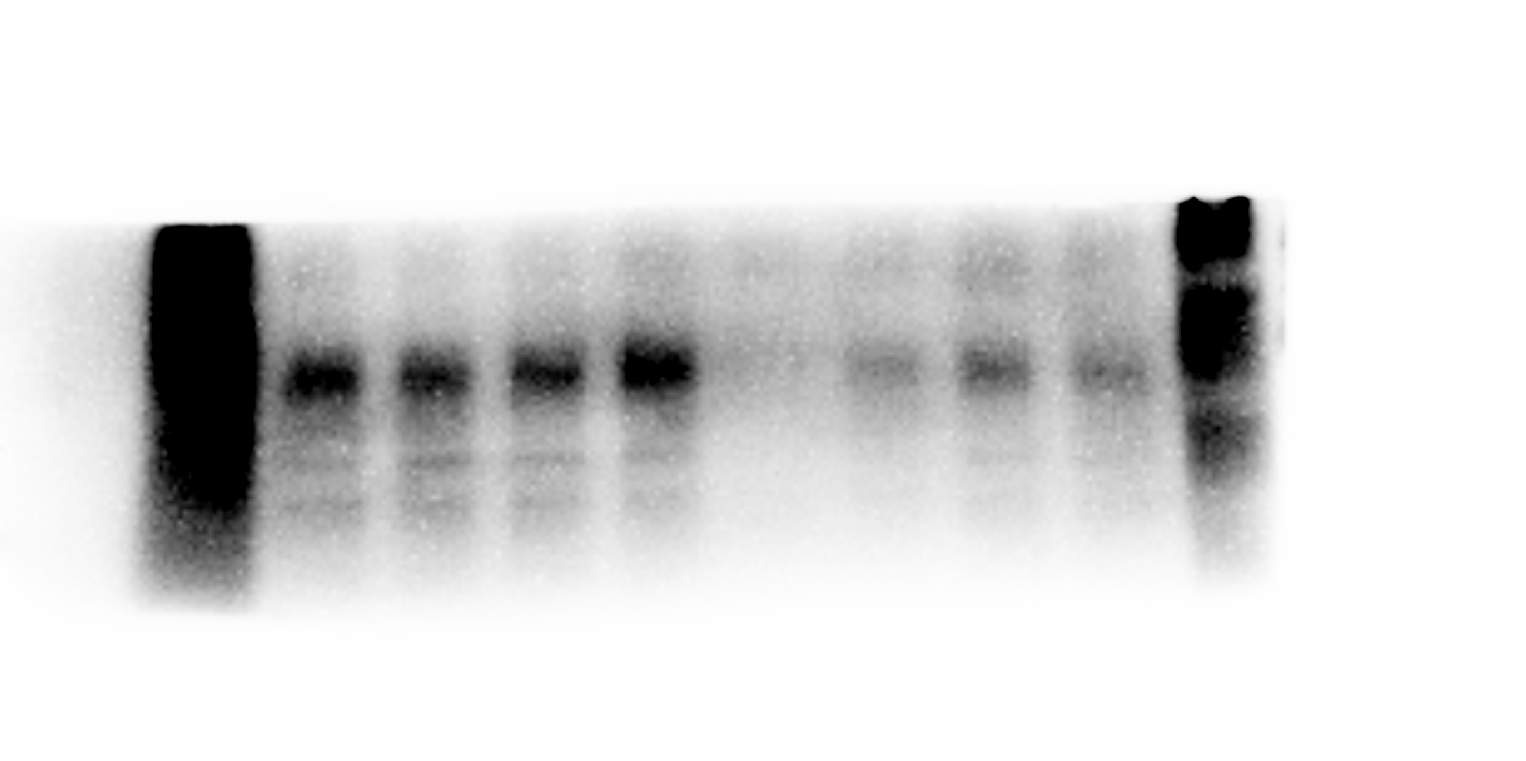

Supplement: Supplementary file 4 — Source Data [file 41467_2022_28500_MOESM4_ESM.zip › Source data/Fig4 F/PD-CP.tif]

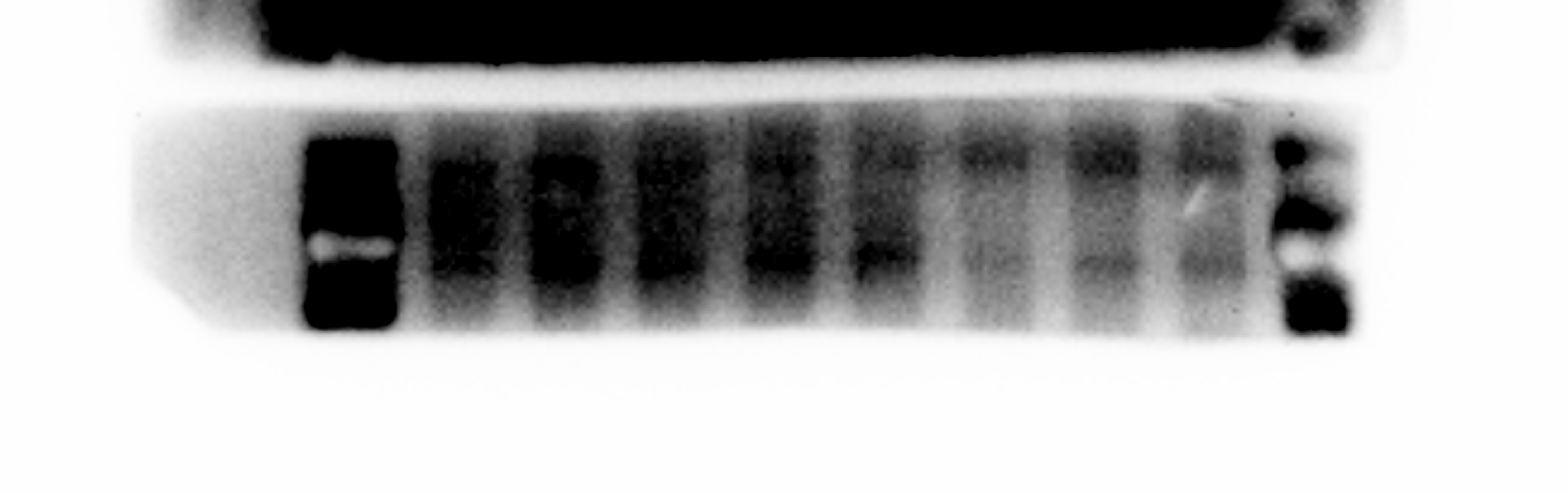

Supplement: Supplementary file 4 — Source Data [file 41467_2022_28500_MOESM4_ESM.zip › Source data/Fig4 F/pd-Raf1.tif]

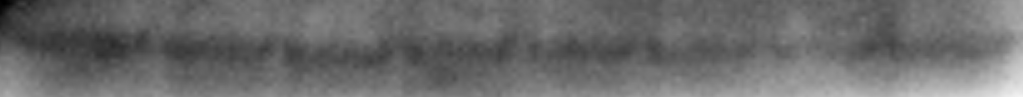

Supplement: Supplementary file 4 — Source Data [file 41467_2022_28500_MOESM4_ESM.zip › Source data/Fig4 F/PEBP4.tif]

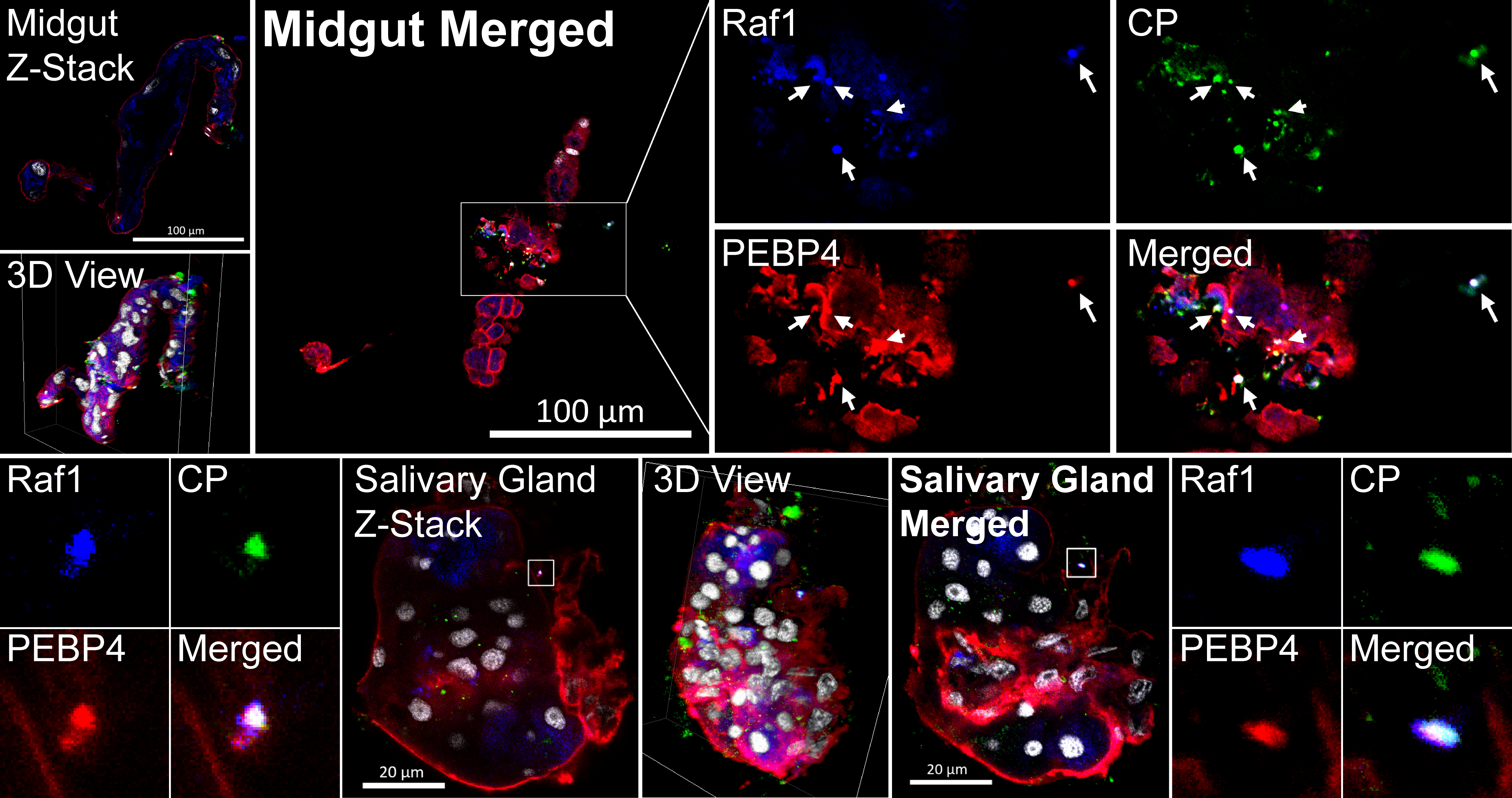

Supplement: Supplementary file 4 — Source Data [file 41467_2022_28500_MOESM4_ESM.zip › Source data/Fig4 G/Fig 4G.tif]

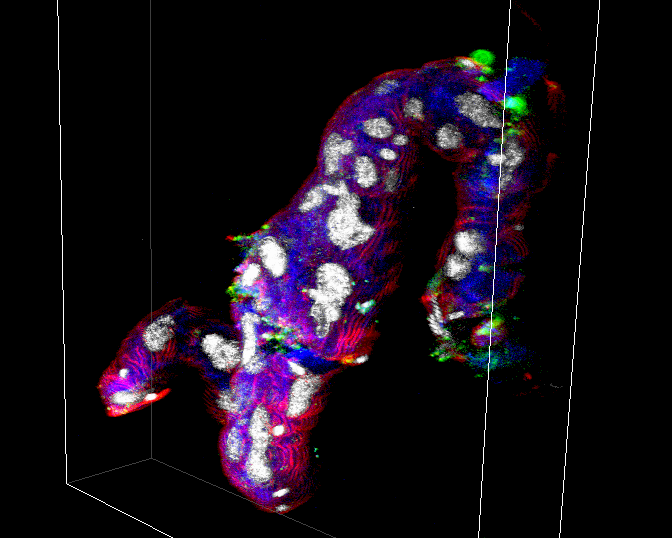

Supplement: Supplementary file 4 — Source Data [file 41467_2022_28500_MOESM4_ESM.zip › Source data/Fig4 G/MG (Snapshot).tif]

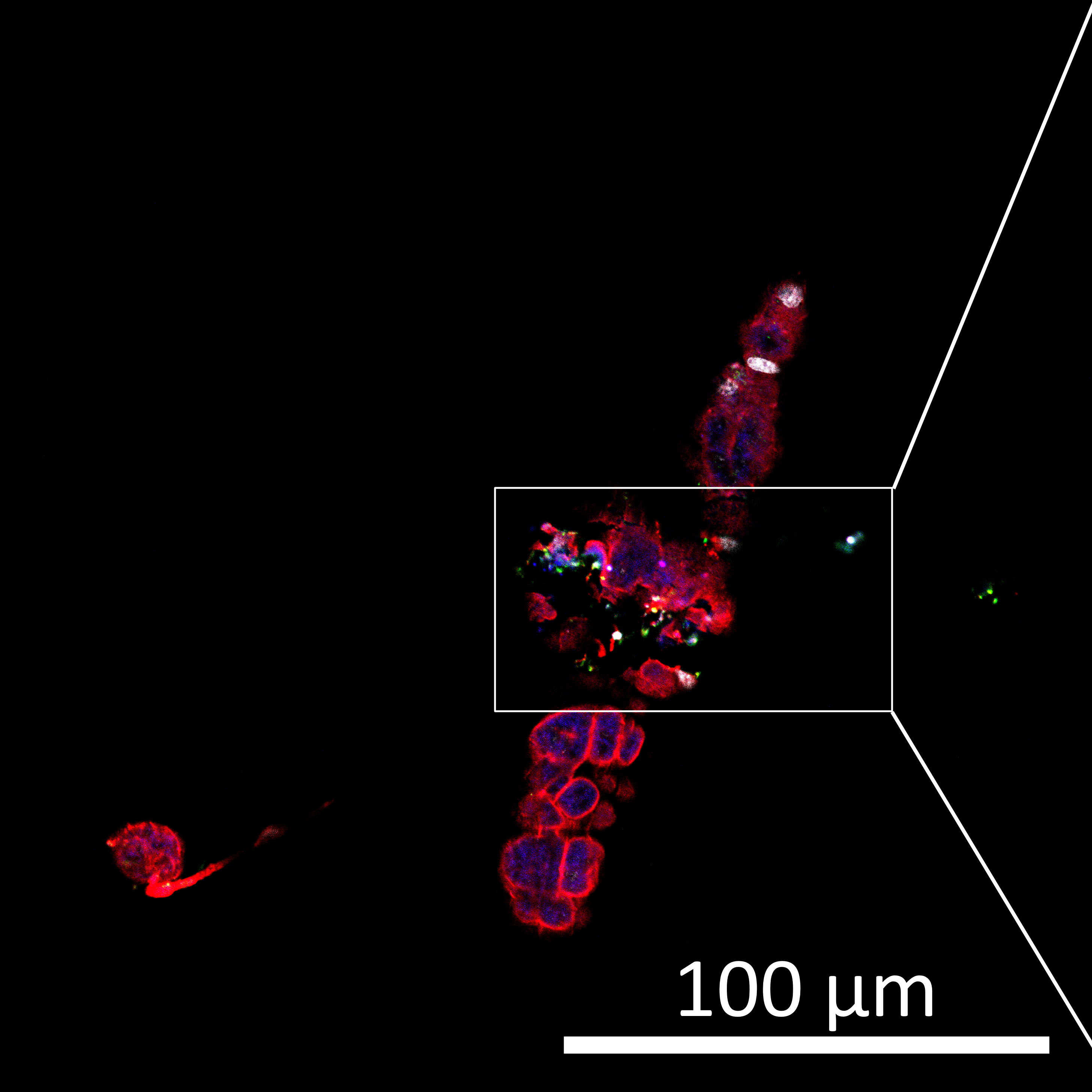

Supplement: Supplementary file 4 — Source Data [file 41467_2022_28500_MOESM4_ESM.zip › Source data/Fig4 G/MG-1_c1+2+3+4.tif]

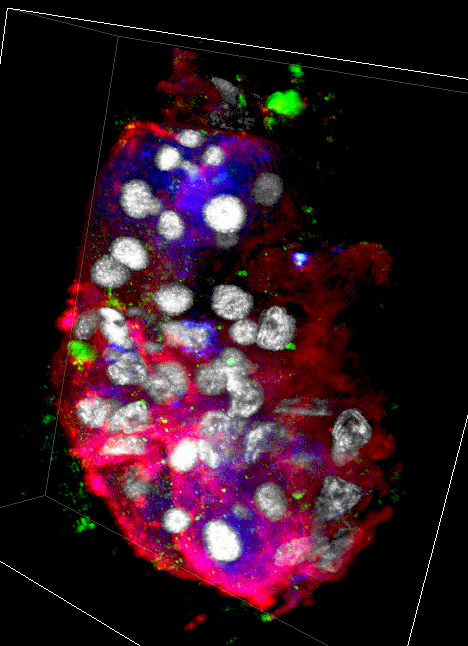

Supplement: Supplementary file 4 — Source Data [file 41467_2022_28500_MOESM4_ESM.zip › Source data/Fig4 G/SG (Snapshot).tif]

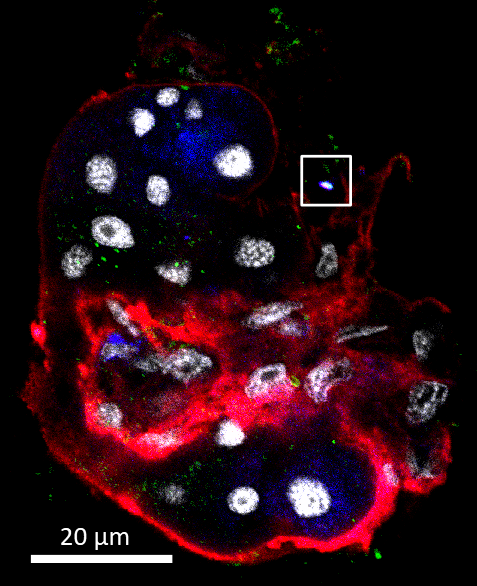

Supplement: Supplementary file 4 — Source Data [file 41467_2022_28500_MOESM4_ESM.zip › Source data/Fig4 G/Untitled28.tif]

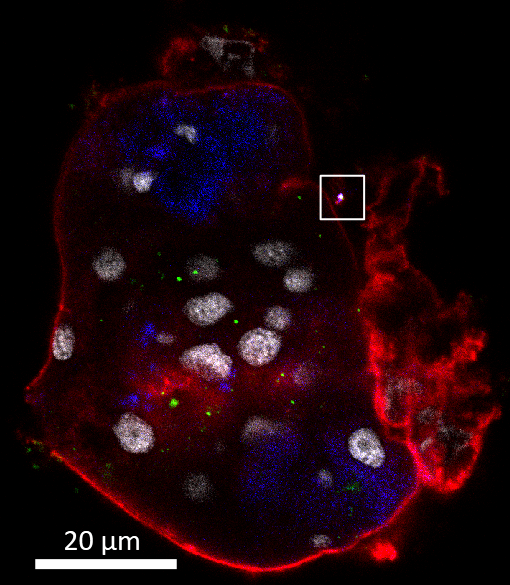

Supplement: Supplementary file 4 — Source Data [file 41467_2022_28500_MOESM4_ESM.zip › Source data/Fig4 G/Untitled37.tif]

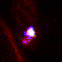

Supplement: Supplementary file 4 — Source Data [file 41467_2022_28500_MOESM4_ESM.zip › Source data/Fig4 G/Untitled40.tif]

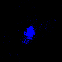

Supplement: Supplementary file 4 — Source Data [file 41467_2022_28500_MOESM4_ESM.zip › Source data/Fig4 G/Untitled41.tif]

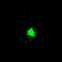

Supplement: Supplementary file 4 — Source Data [file 41467_2022_28500_MOESM4_ESM.zip › Source data/Fig4 G/Untitled42.tif]

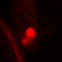

Supplement: Supplementary file 4 — Source Data [file 41467_2022_28500_MOESM4_ESM.zip › Source data/Fig4 G/Untitled43.tif]

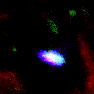

Supplement: Supplementary file 4 — Source Data [file 41467_2022_28500_MOESM4_ESM.zip › Source data/Fig4 G/Untitled44_c2+3+4.tif]

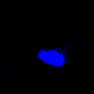

Supplement: Supplementary file 4 — Source Data [file 41467_2022_28500_MOESM4_ESM.zip › Source data/Fig4 G/Untitled44_c2.tif]

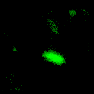

Supplement: Supplementary file 4 — Source Data [file 41467_2022_28500_MOESM4_ESM.zip › Source data/Fig4 G/Untitled44_c3.tif]

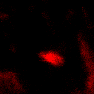

Supplement: Supplementary file 4 — Source Data [file 41467_2022_28500_MOESM4_ESM.zip › Source data/Fig4 G/Untitled44_c4.tif]

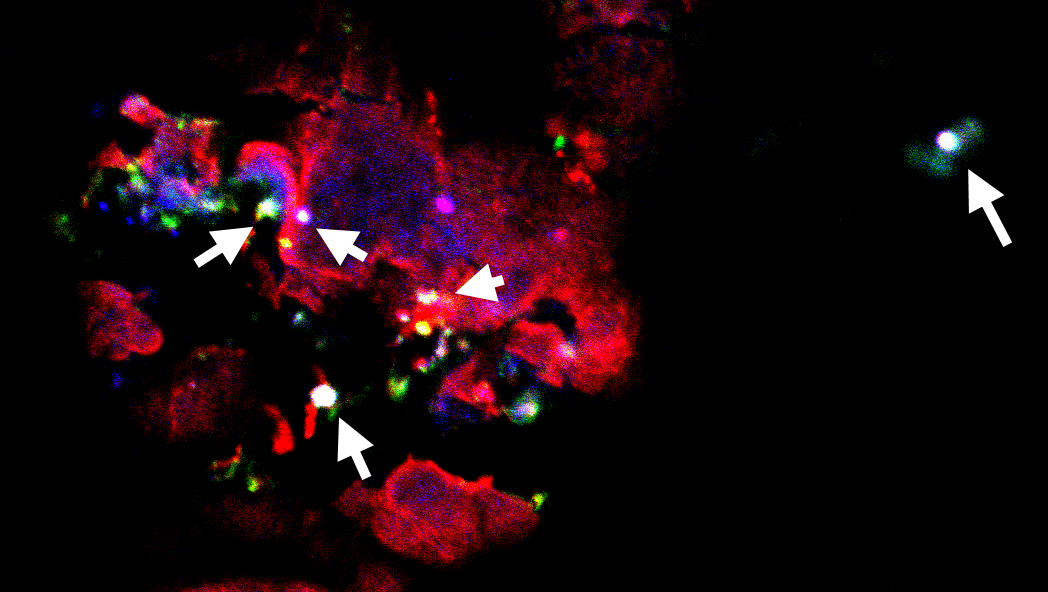

Supplement: Supplementary file 4 — Source Data [file 41467_2022_28500_MOESM4_ESM.zip › Source data/Fig4 G/Untitled45_c2+3+4.tif]

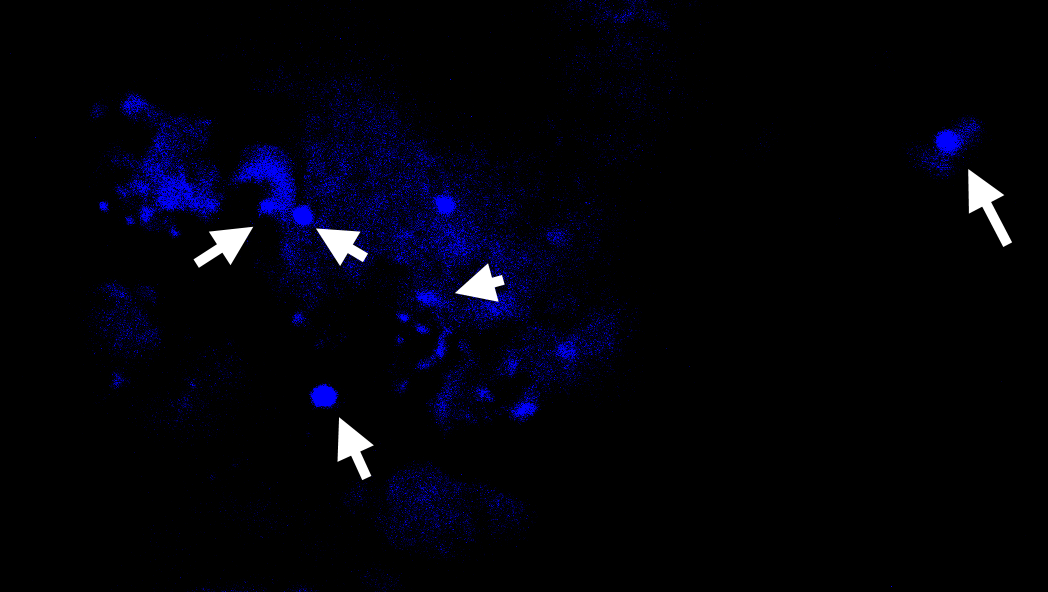

Supplement: Supplementary file 4 — Source Data [file 41467_2022_28500_MOESM4_ESM.zip › Source data/Fig4 G/Untitled45_c2.tif]

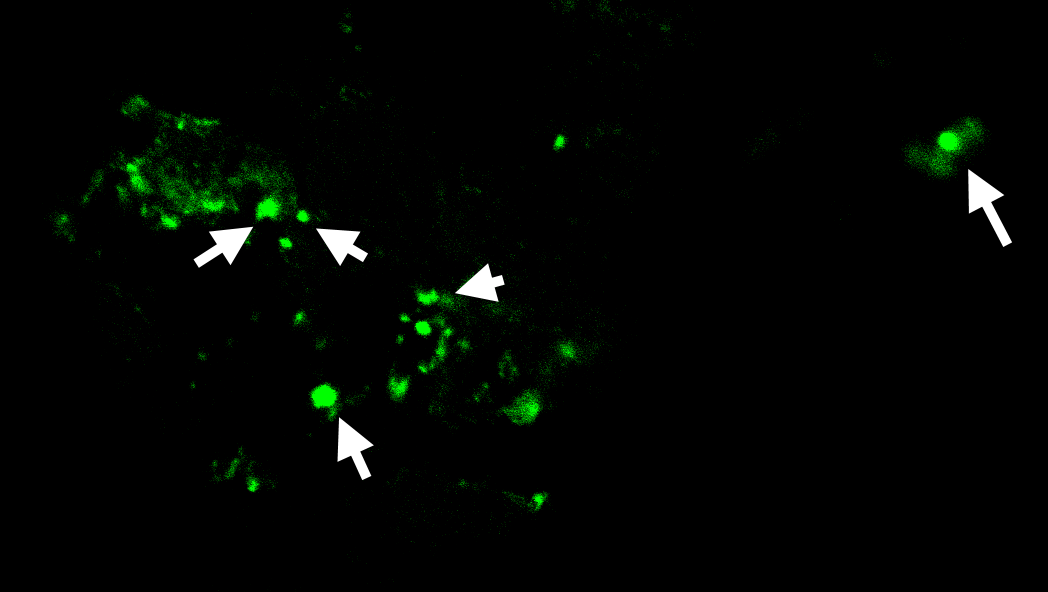

Supplement: Supplementary file 4 — Source Data [file 41467_2022_28500_MOESM4_ESM.zip › Source data/Fig4 G/Untitled45_c3.tif]

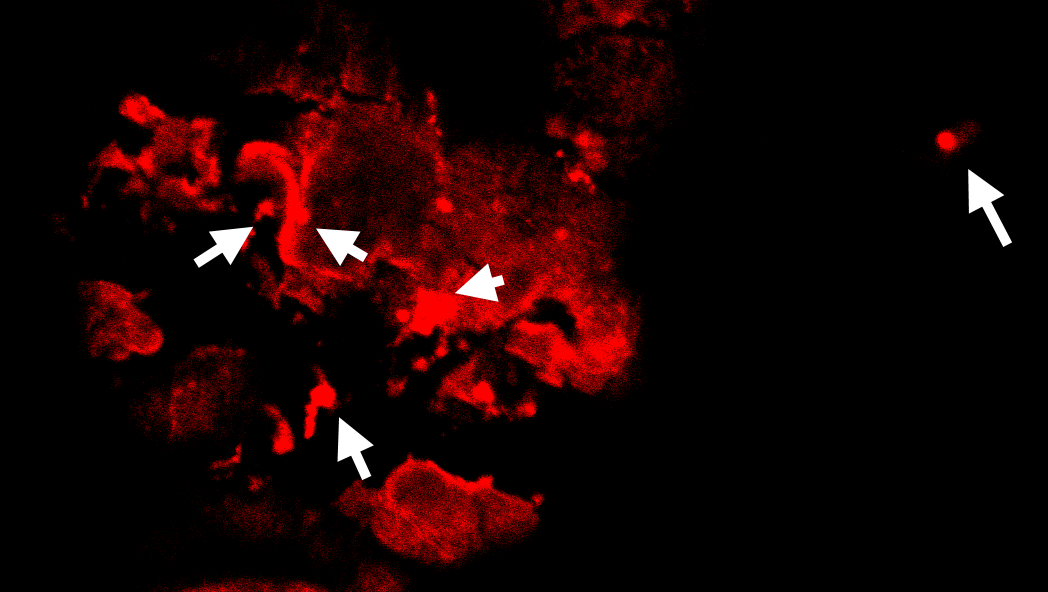

Supplement: Supplementary file 4 — Source Data [file 41467_2022_28500_MOESM4_ESM.zip › Source data/Fig4 G/Untitled45_c4.tif]

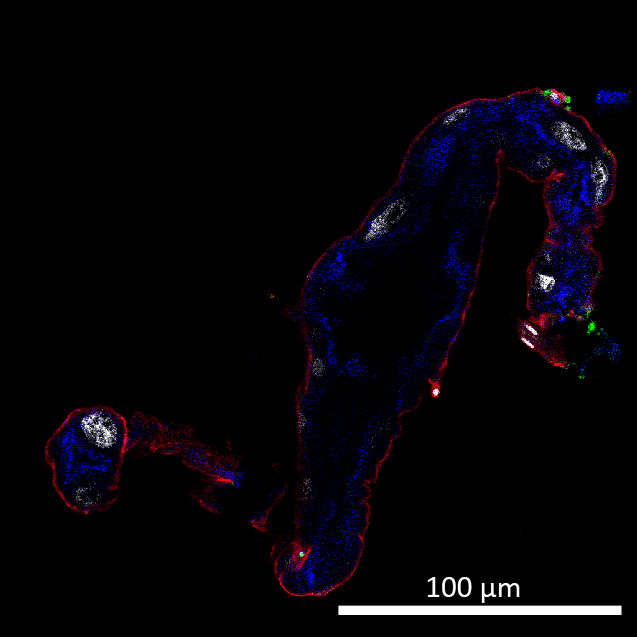

Supplement: Supplementary file 4 — Source Data [file 41467_2022_28500_MOESM4_ESM.zip › Source data/Fig4 G/Untitled47.tif]

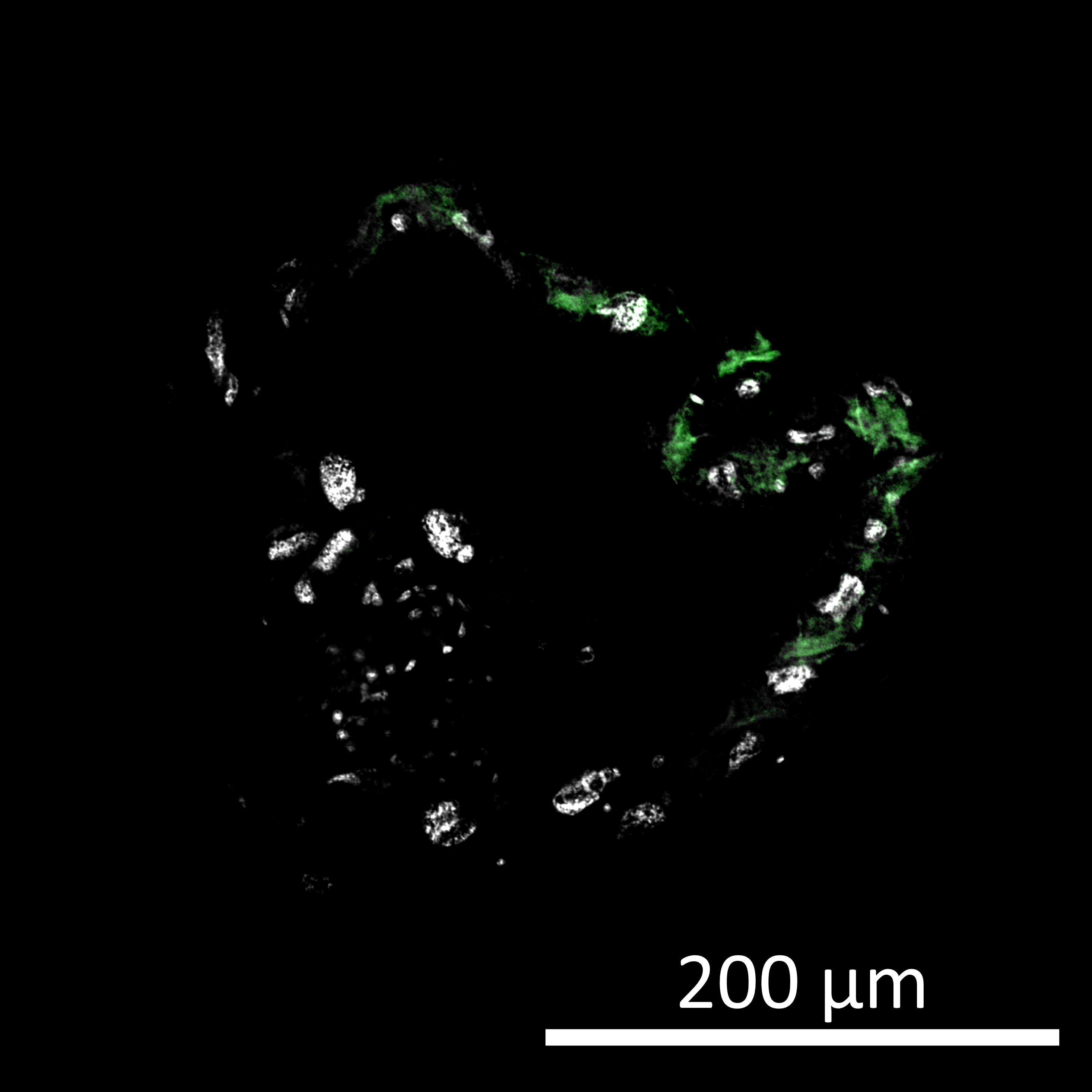

Supplement: Supplementary file 4 — Source Data [file 41467_2022_28500_MOESM4_ESM.zip › Source data/Fig4 H/DMSO-MG_c1+2.jpg]

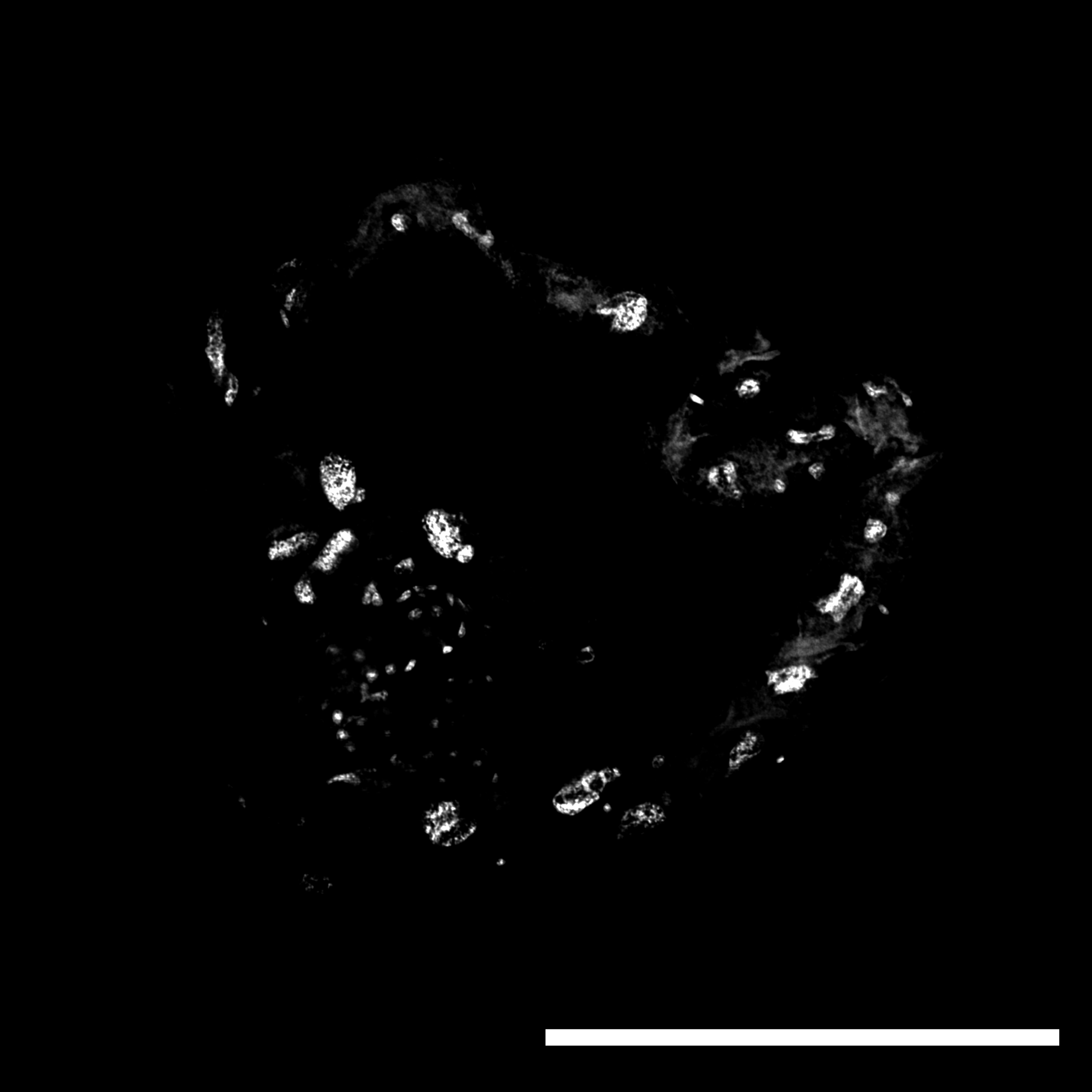

Supplement: Supplementary file 4 — Source Data [file 41467_2022_28500_MOESM4_ESM.zip › Source data/Fig4 H/DMSO-MG_c1.jpg]

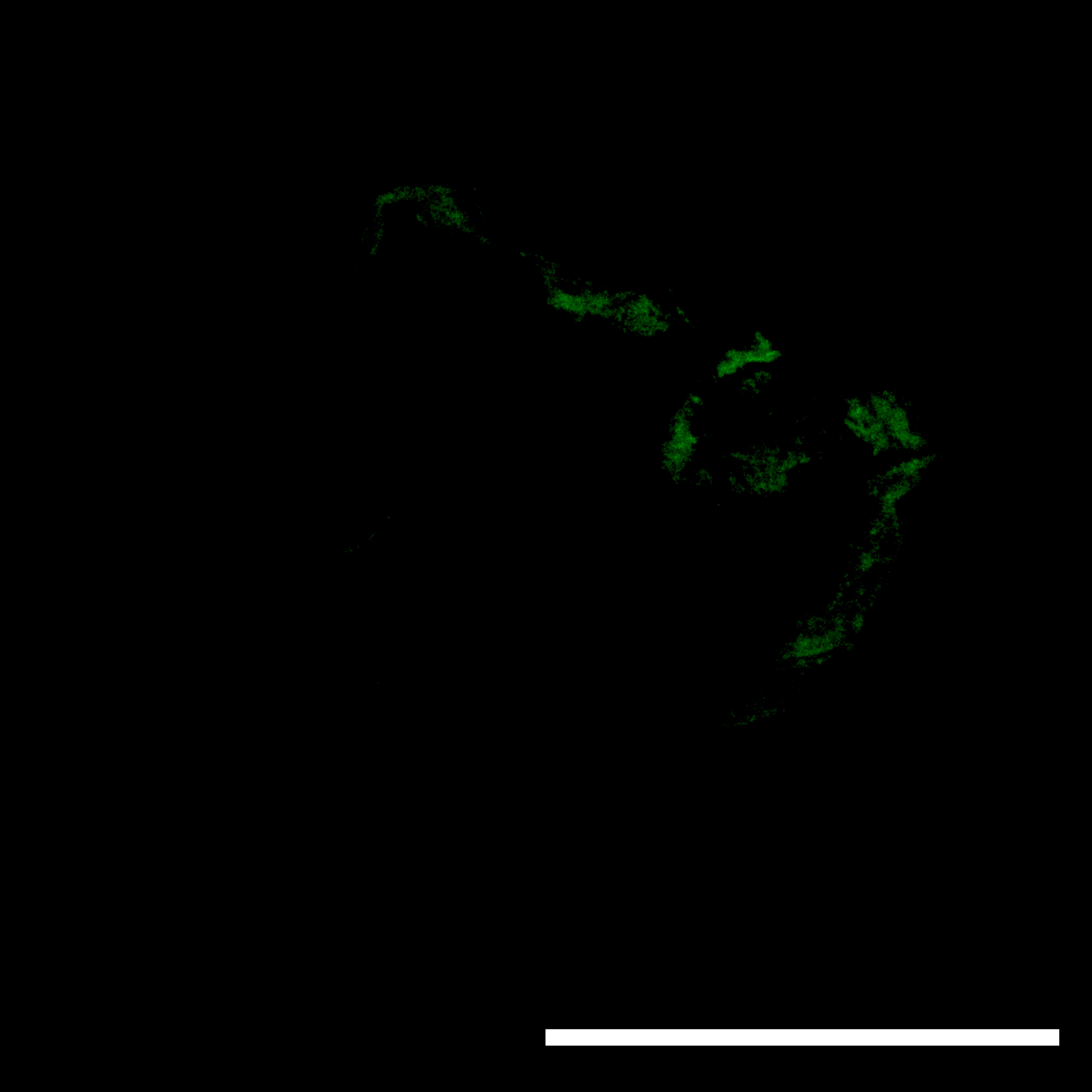

Supplement: Supplementary file 4 — Source Data [file 41467_2022_28500_MOESM4_ESM.zip › Source data/Fig4 H/DMSO-MG_c2.jpg]

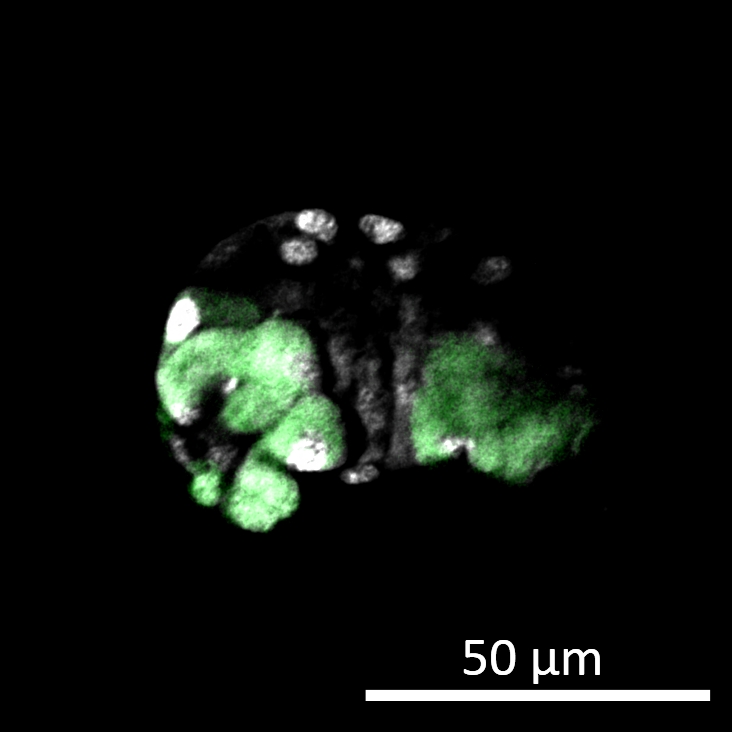

Supplement: Supplementary file 4 — Source Data [file 41467_2022_28500_MOESM4_ESM.zip › Source data/Fig4 H/DMSO-SG_c1+2.jpg]

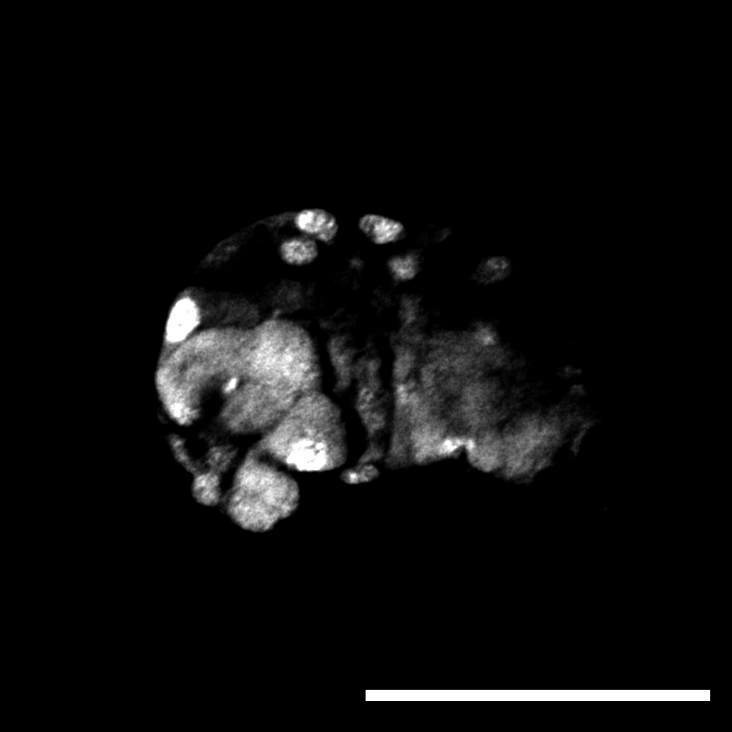

Supplement: Supplementary file 4 — Source Data [file 41467_2022_28500_MOESM4_ESM.zip › Source data/Fig4 H/DMSO-SG_c1.jpg]

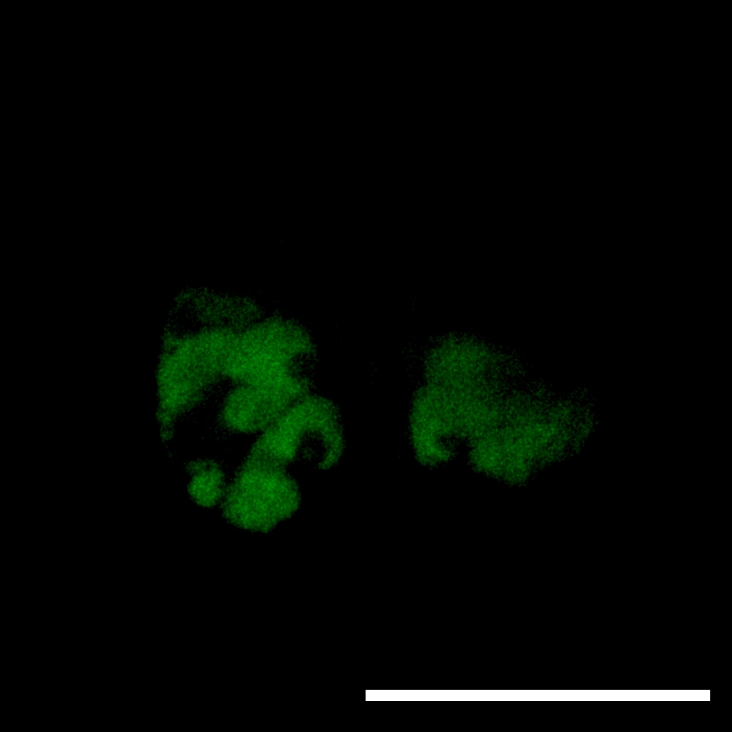

Supplement: Supplementary file 4 — Source Data [file 41467_2022_28500_MOESM4_ESM.zip › Source data/Fig4 H/DMSO-SG_c2.jpg]

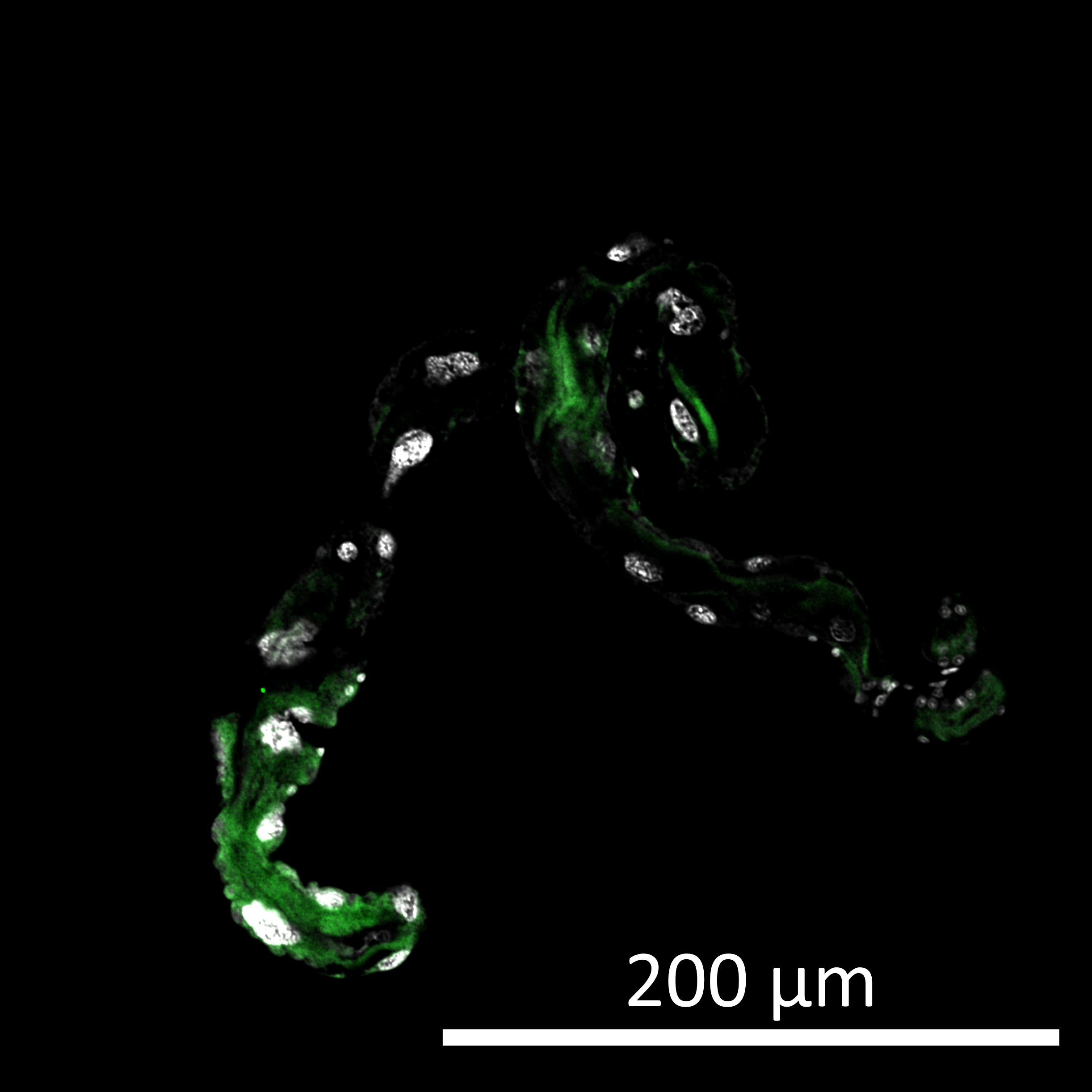

Supplement: Supplementary file 4 — Source Data [file 41467_2022_28500_MOESM4_ESM.zip › Source data/Fig4 H/Mird-MG_c1+2.jpg]

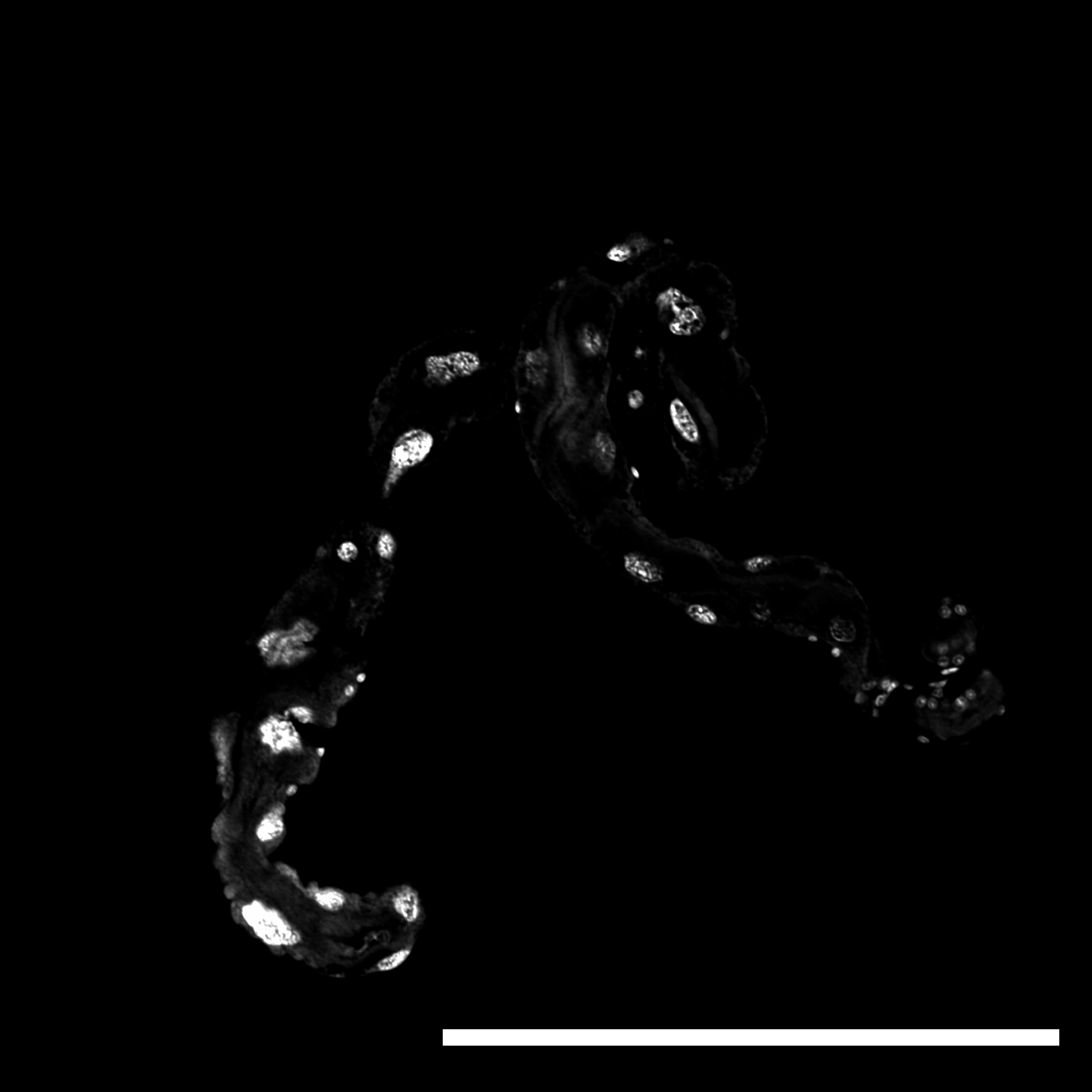

Supplement: Supplementary file 4 — Source Data [file 41467_2022_28500_MOESM4_ESM.zip › Source data/Fig4 H/Mird-MG_c1.jpg]

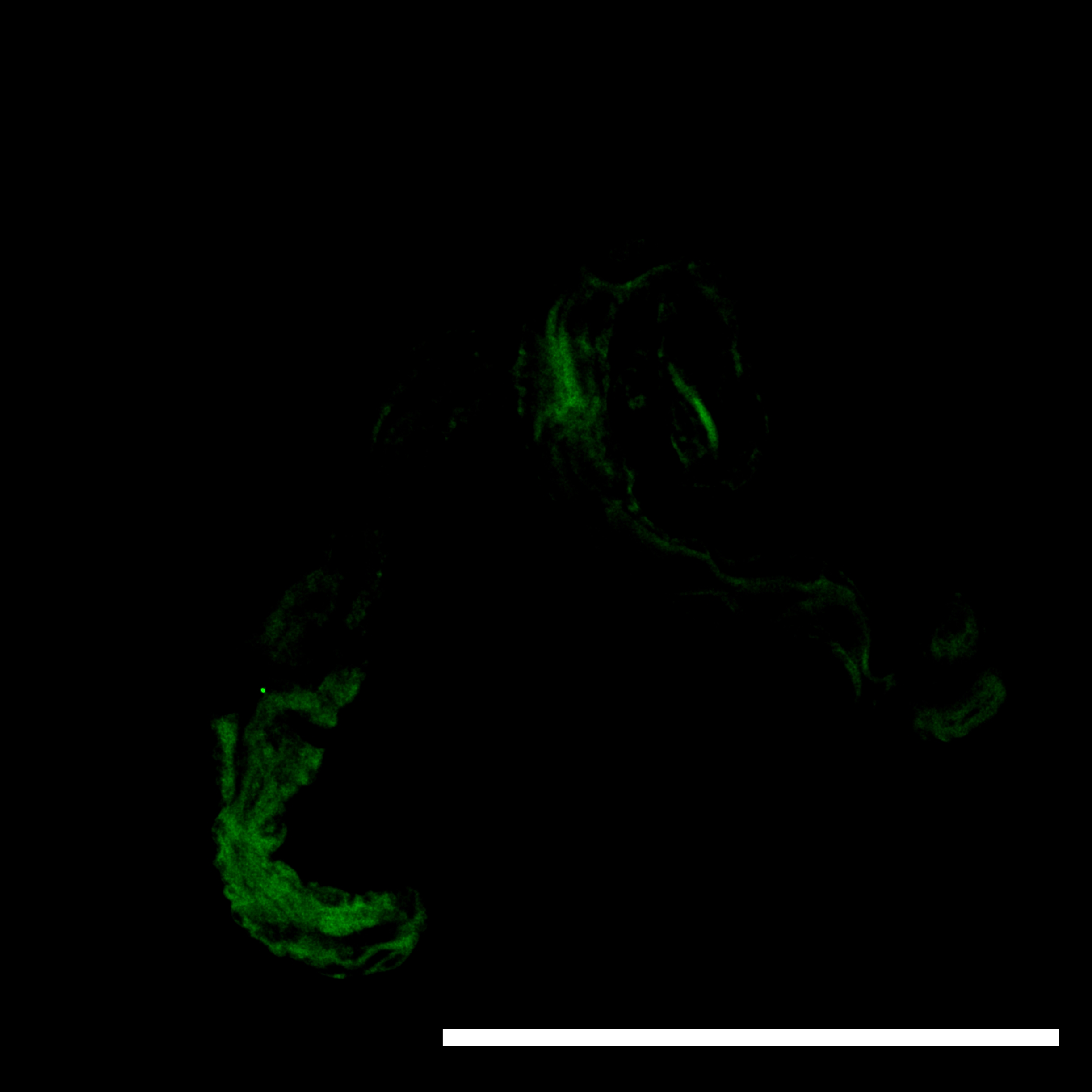

Supplement: Supplementary file 4 — Source Data [file 41467_2022_28500_MOESM4_ESM.zip › Source data/Fig4 H/Mird-MG_c2.jpg]

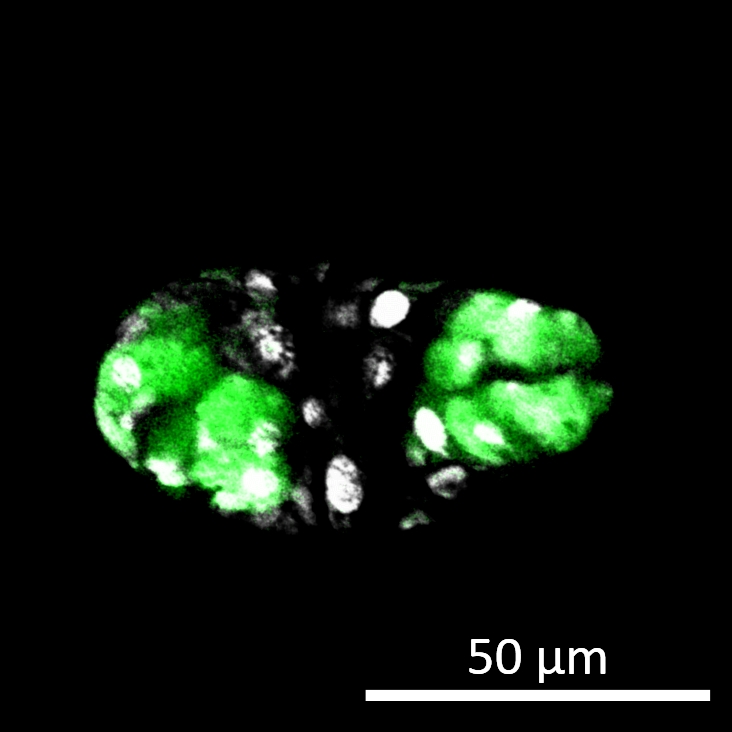

Supplement: Supplementary file 4 — Source Data [file 41467_2022_28500_MOESM4_ESM.zip › Source data/Fig4 H/Mird-SG_c1+2.jpg]

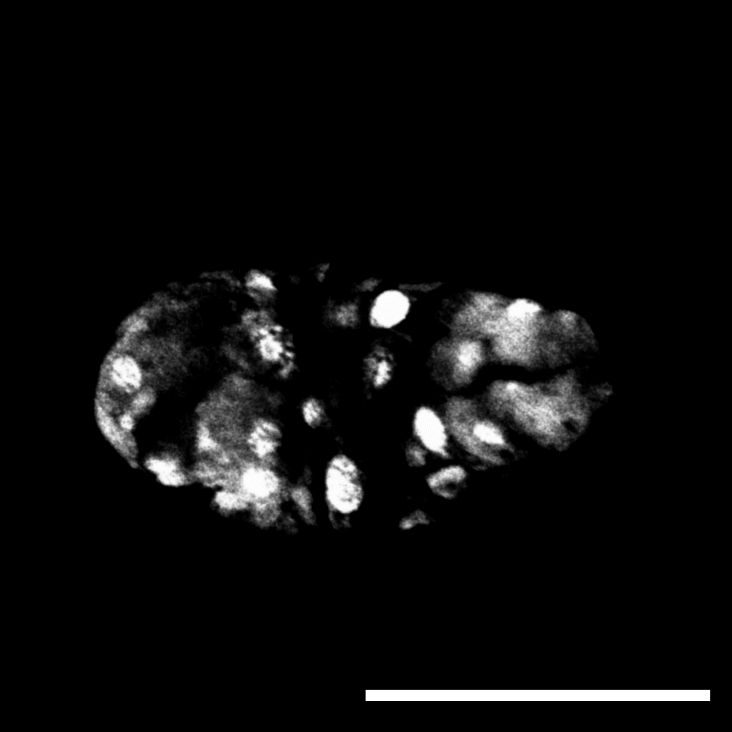

Supplement: Supplementary file 4 — Source Data [file 41467_2022_28500_MOESM4_ESM.zip › Source data/Fig4 H/Mird-SG_c1.jpg]

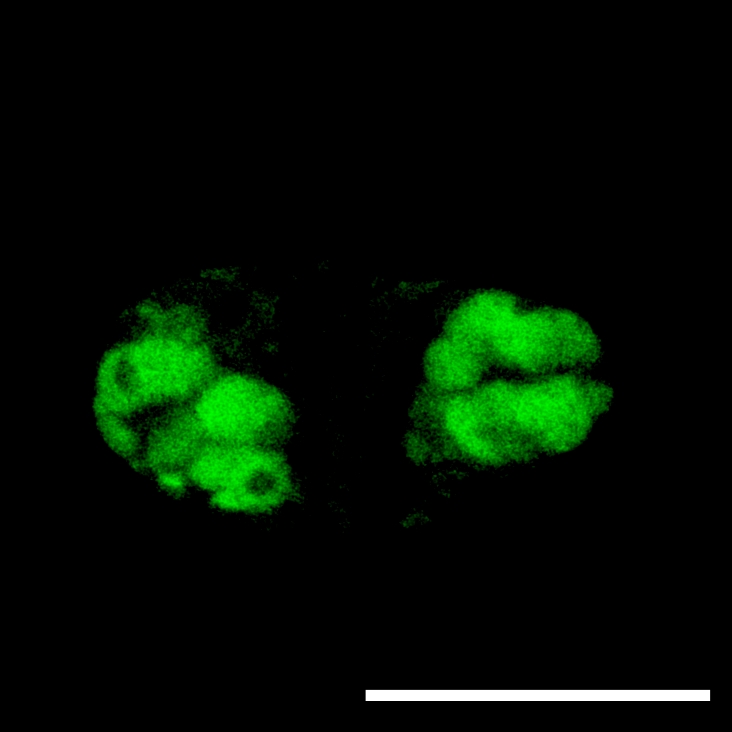

Supplement: Supplementary file 4 — Source Data [file 41467_2022_28500_MOESM4_ESM.zip › Source data/Fig4 H/Mird-SG_c2.jpg]

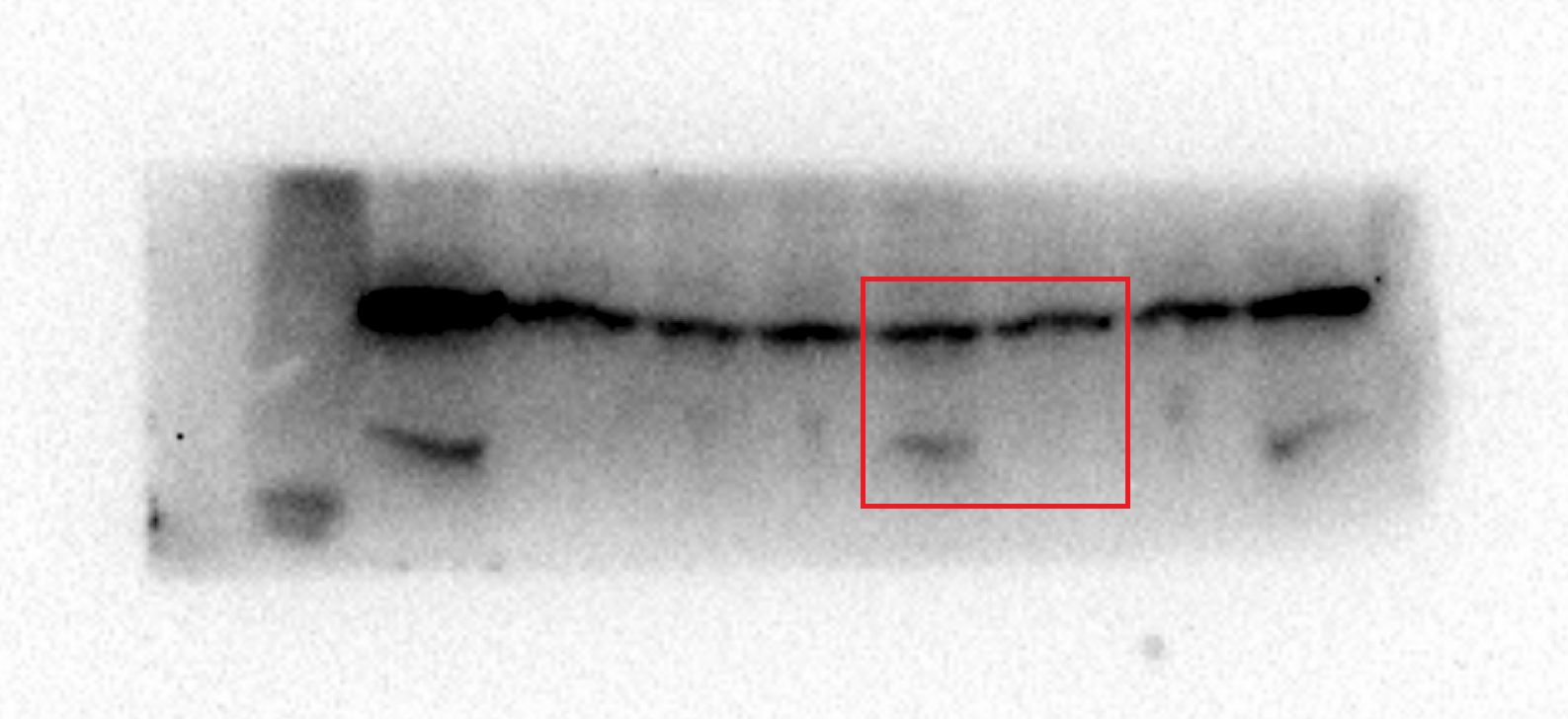

Supplement: Supplementary file 4 — Source Data [file 41467_2022_28500_MOESM4_ESM.zip › Source data/Fig4 I/ATG8.tif]

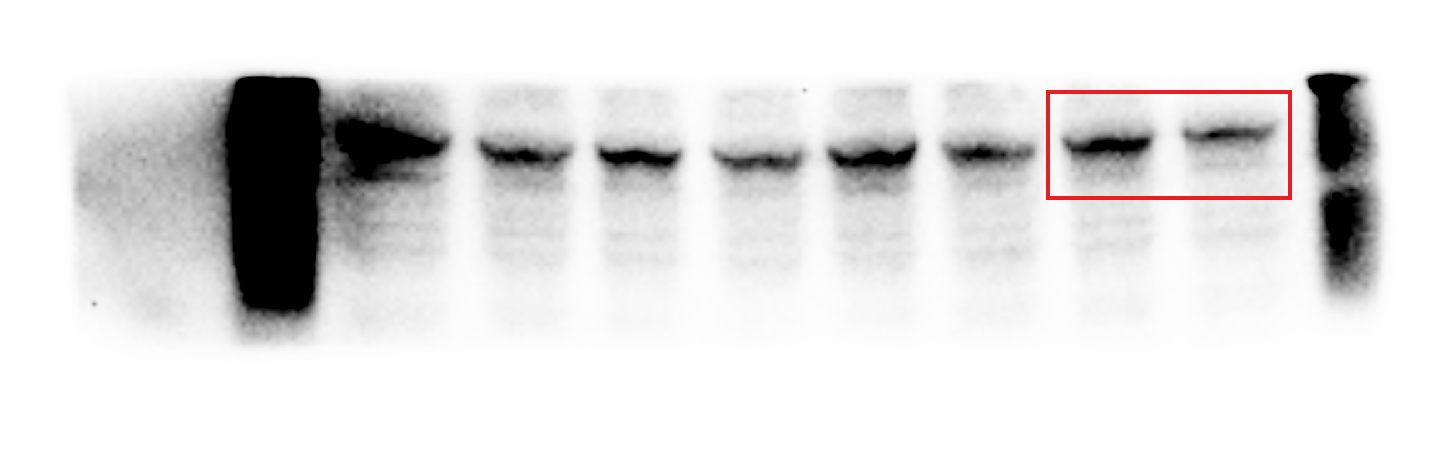

Supplement: Supplementary file 4 — Source Data [file 41467_2022_28500_MOESM4_ESM.zip › Source data/Fig4 I/Casp3.tif]

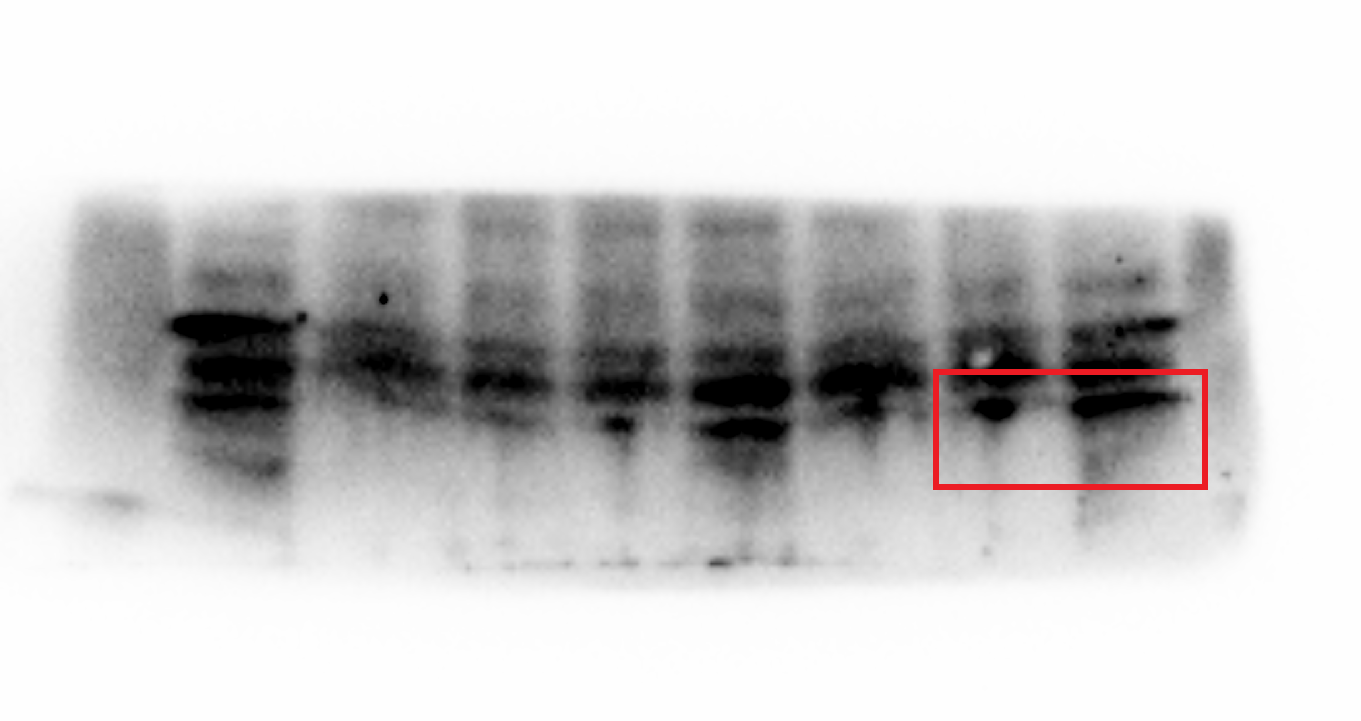

Supplement: Supplementary file 4 — Source Data [file 41467_2022_28500_MOESM4_ESM.zip › Source data/Fig4 I/cCasp3.tif]

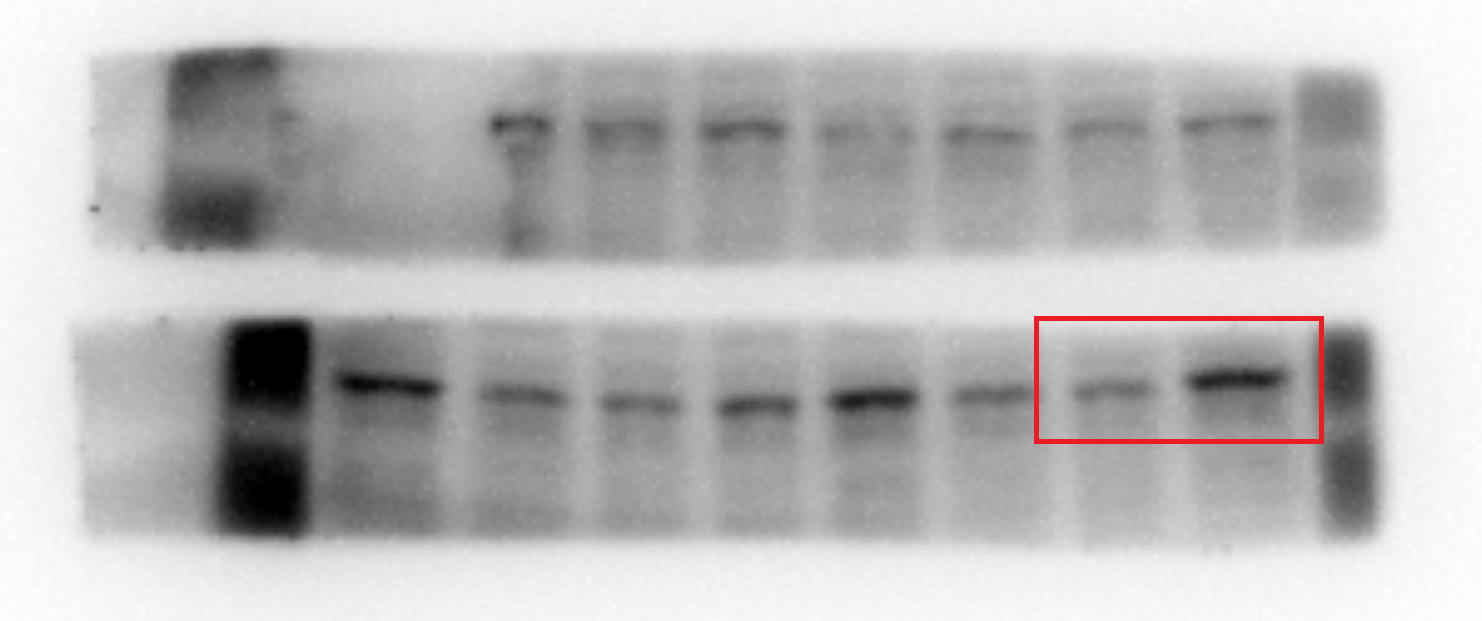

Supplement: Supplementary file 4 — Source Data [file 41467_2022_28500_MOESM4_ESM.zip › Source data/Fig4 I/CP.tif]

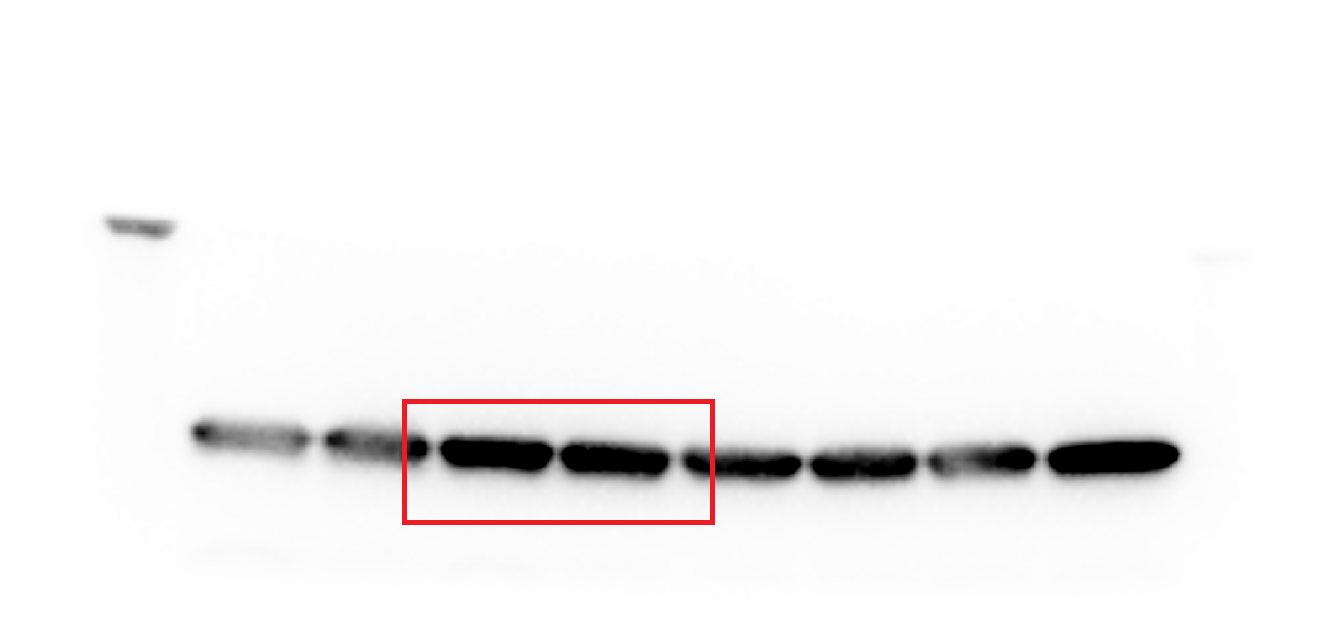

Supplement: Supplementary file 4 — Source Data [file 41467_2022_28500_MOESM4_ESM.zip › Source data/Fig4 I/GAPDH.tif]

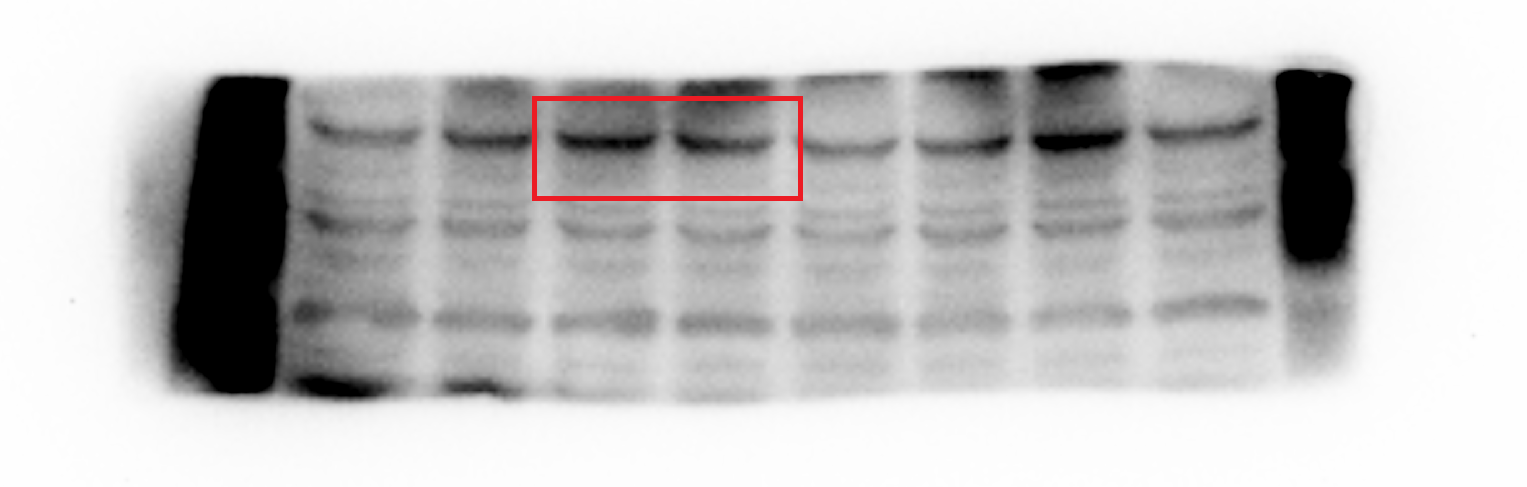

Supplement: Supplementary file 4 — Source Data [file 41467_2022_28500_MOESM4_ESM.zip › Source data/Fig4 I/PEBP.tif]

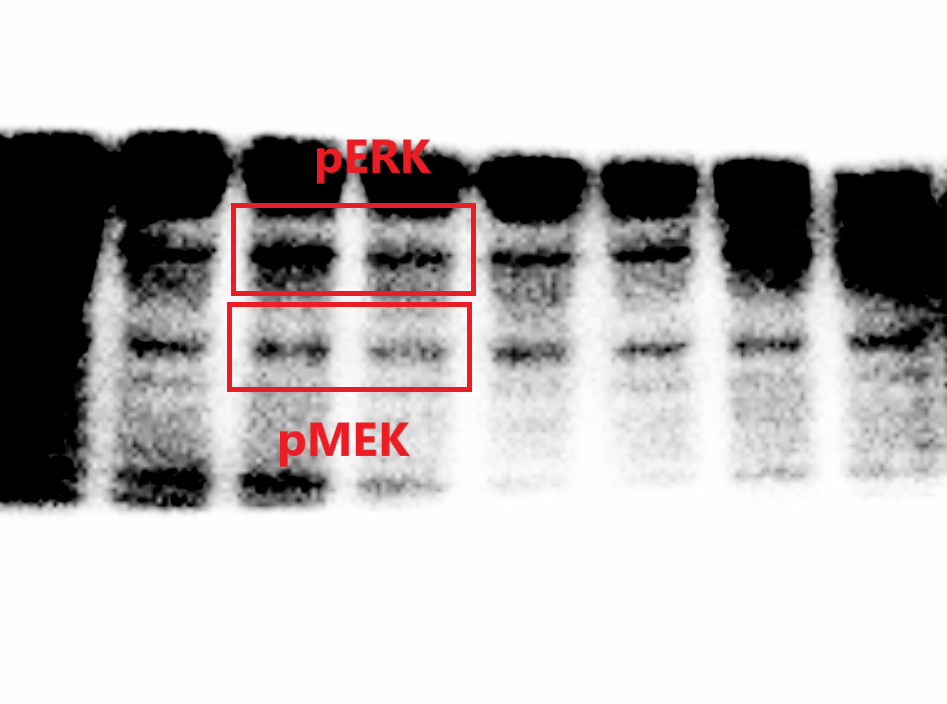

Supplement: Supplementary file 4 — Source Data [file 41467_2022_28500_MOESM4_ESM.zip › Source data/Fig4 I/pMEK&pERK.tif]

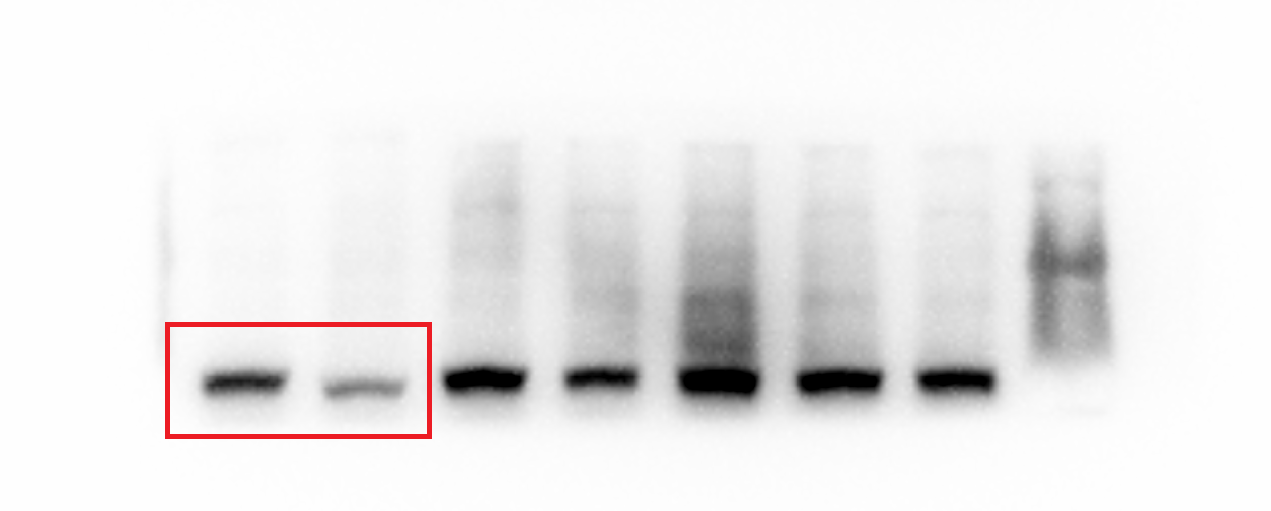

Supplement: Supplementary file 4 — Source Data [file 41467_2022_28500_MOESM4_ESM.zip › Source data/Fig4 I/pRaf1.tif]

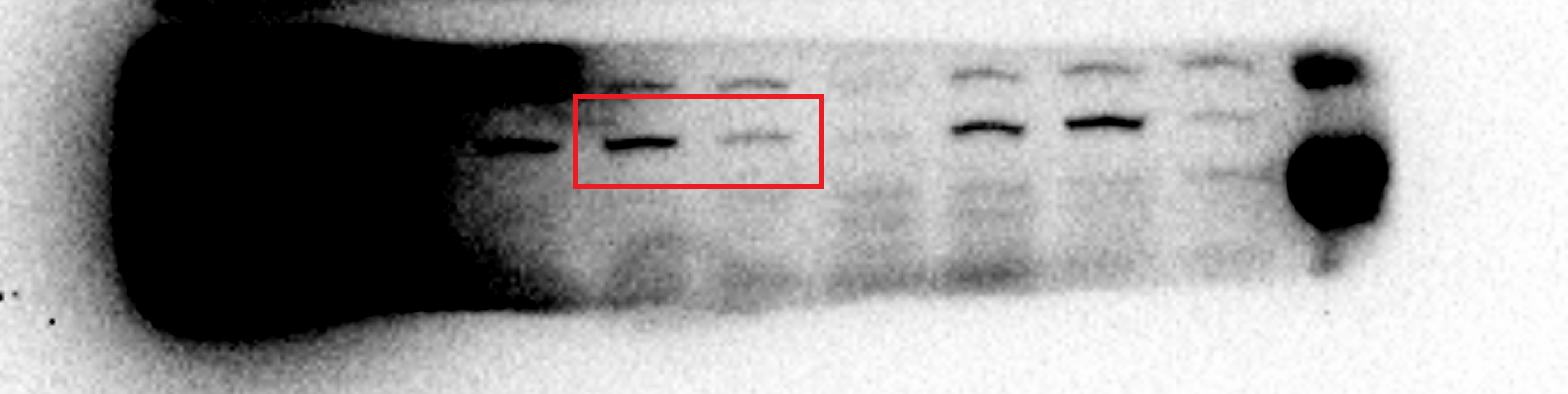

Supplement: Supplementary file 4 — Source Data [file 41467_2022_28500_MOESM4_ESM.zip › Source data/Fig4 I/Raf1.tif]

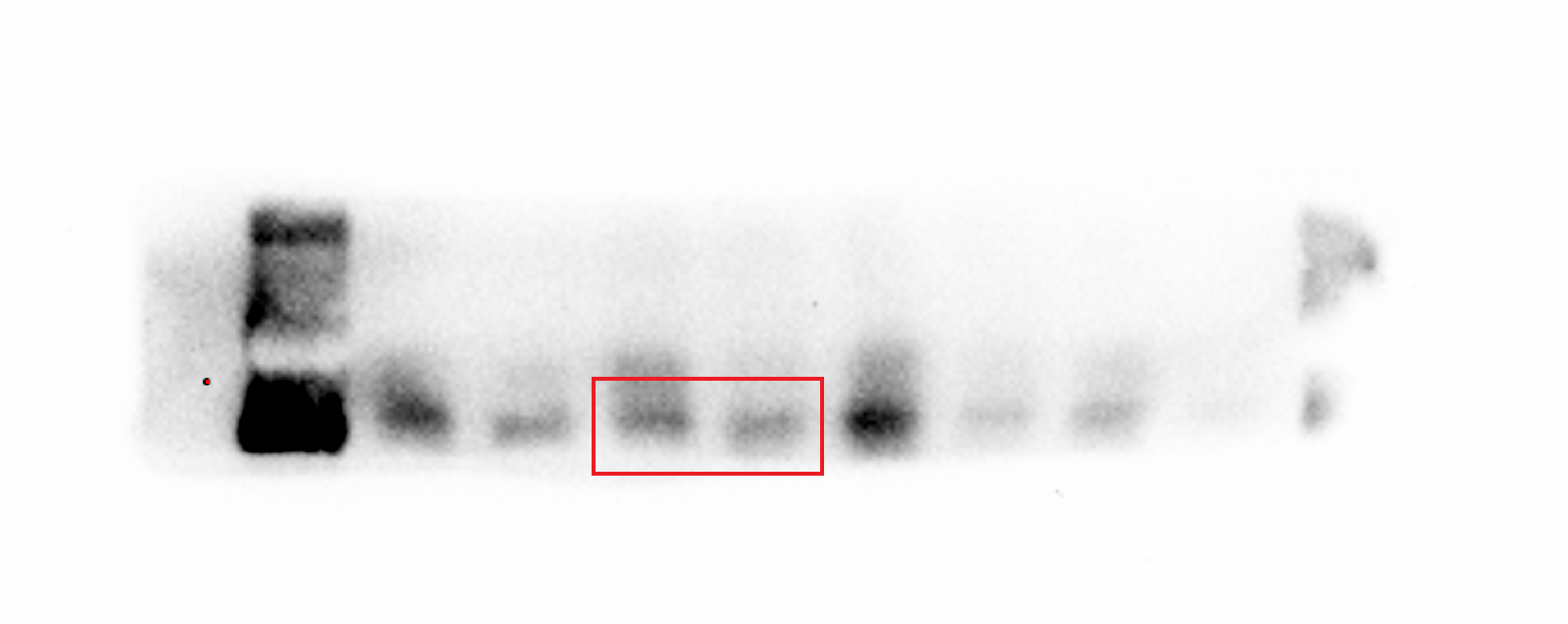

Supplement: Supplementary file 4 — Source Data [file 41467_2022_28500_MOESM4_ESM.zip › Source data/Fig4 I/SQSTM1.tif]

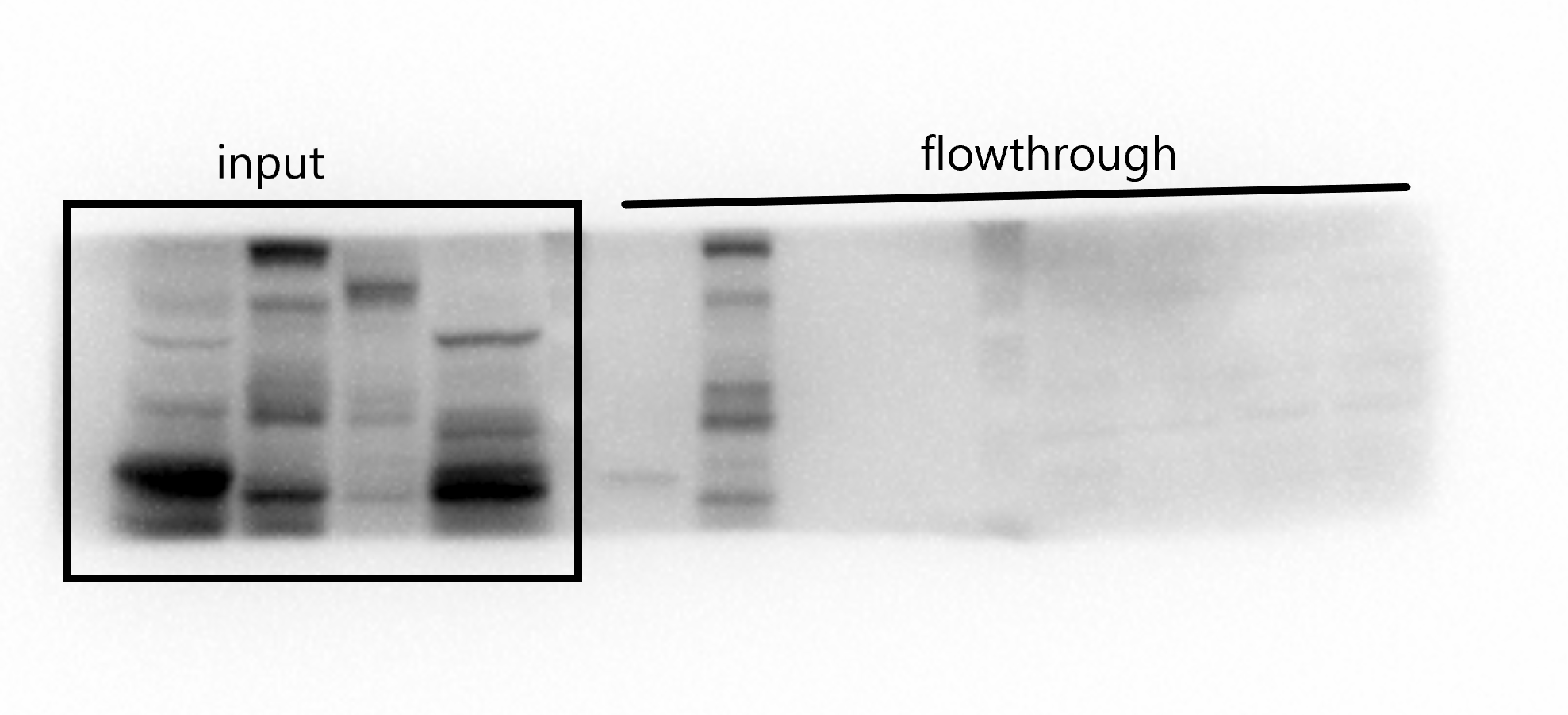

Supplement: Supplementary file 4 — Source Data [file 41467_2022_28500_MOESM4_ESM.zip › Source data/Fig5 A/input-GST.tif]

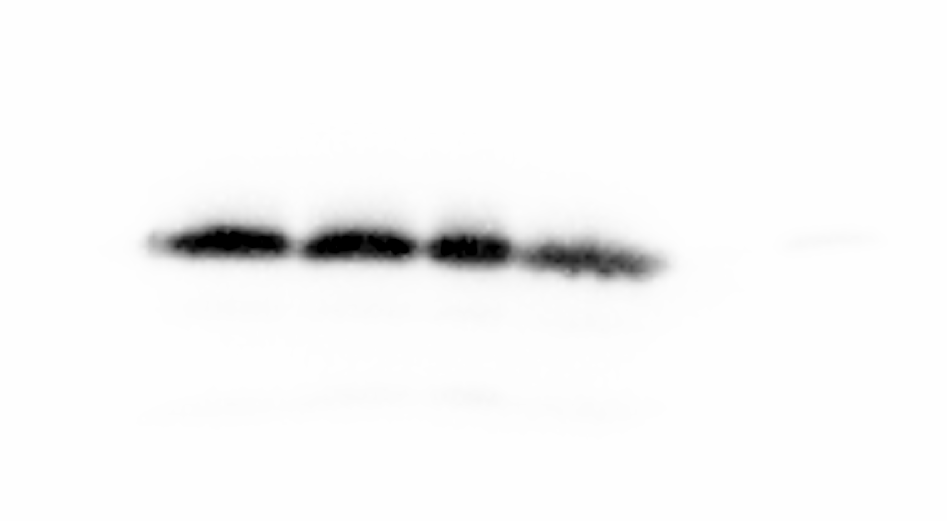

Supplement: Supplementary file 4 — Source Data [file 41467_2022_28500_MOESM4_ESM.zip › Source data/Fig5 A/input-HisATG8.tif]

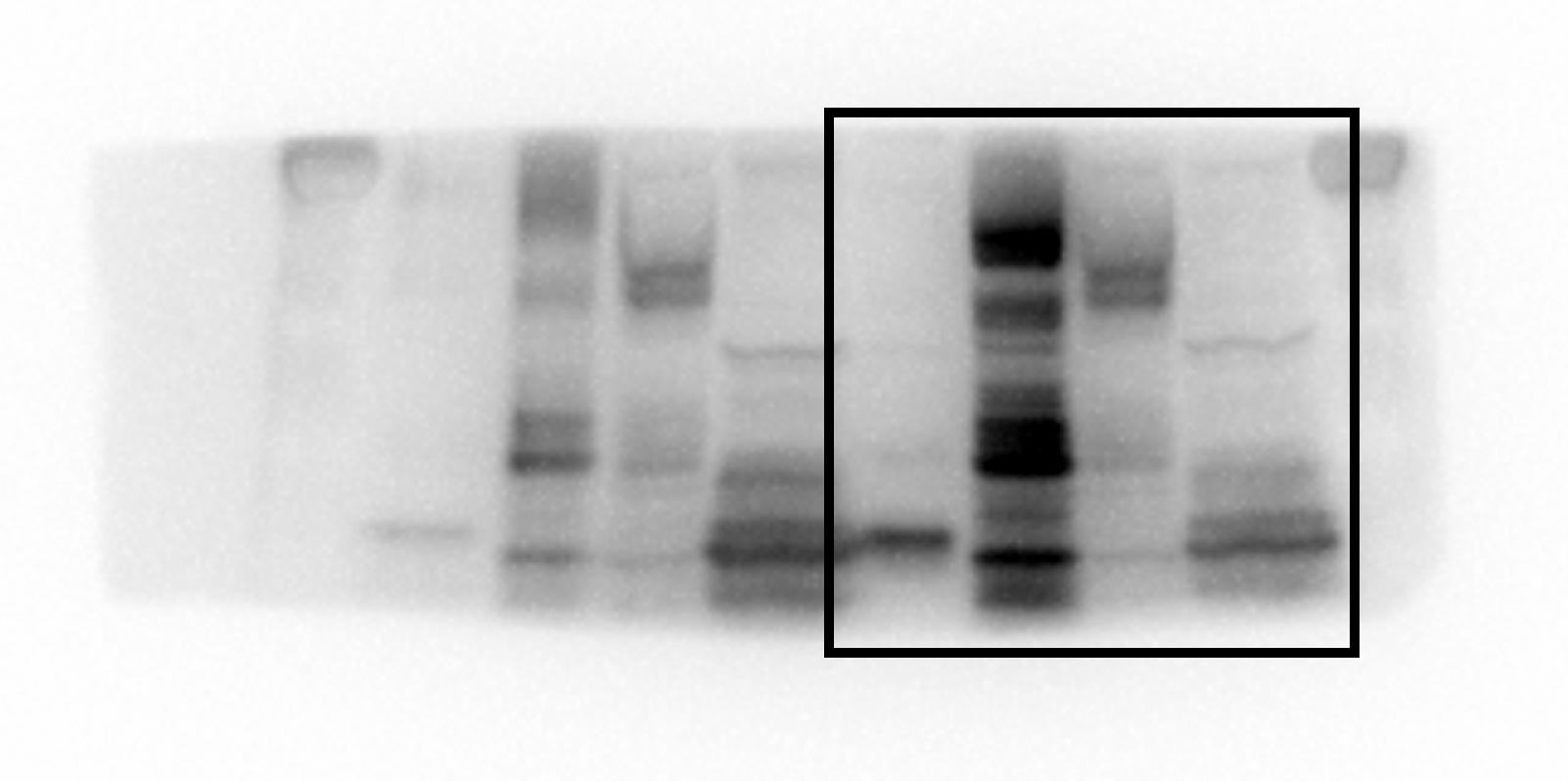

Supplement: Supplementary file 4 — Source Data [file 41467_2022_28500_MOESM4_ESM.zip › Source data/Fig5 A/PD-GST.tif]

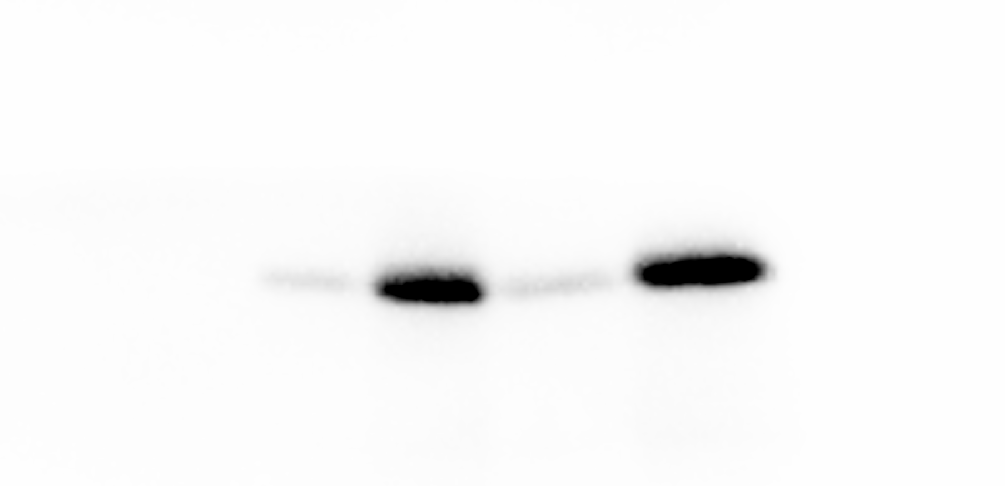

Supplement: Supplementary file 4 — Source Data [file 41467_2022_28500_MOESM4_ESM.zip › Source data/Fig5 A/PD-HisATG8.tif]

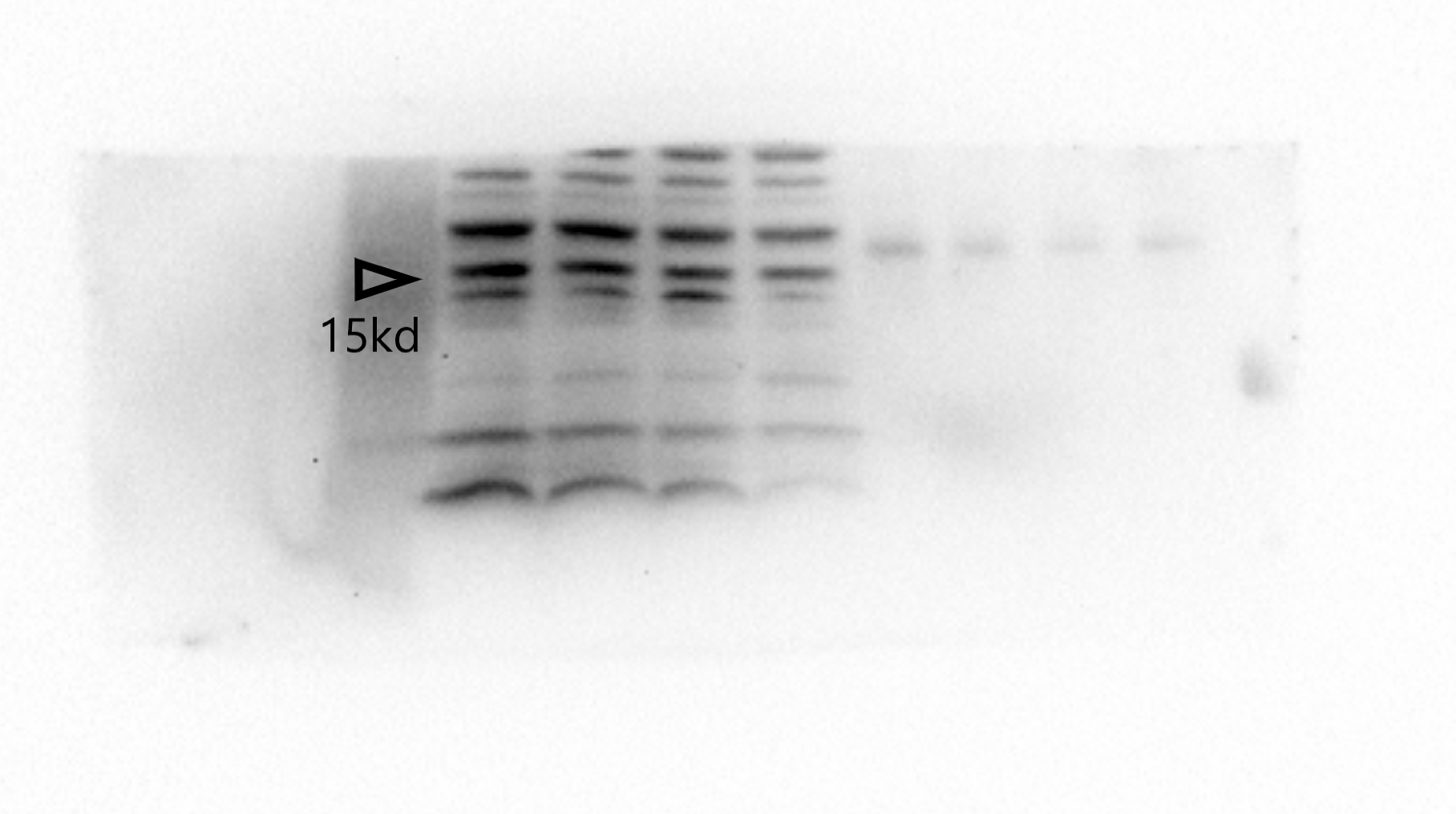

Supplement: Supplementary file 4 — Source Data [file 41467_2022_28500_MOESM4_ESM.zip › Source data/Fig5 B/ATG8 (input+pd).tif]

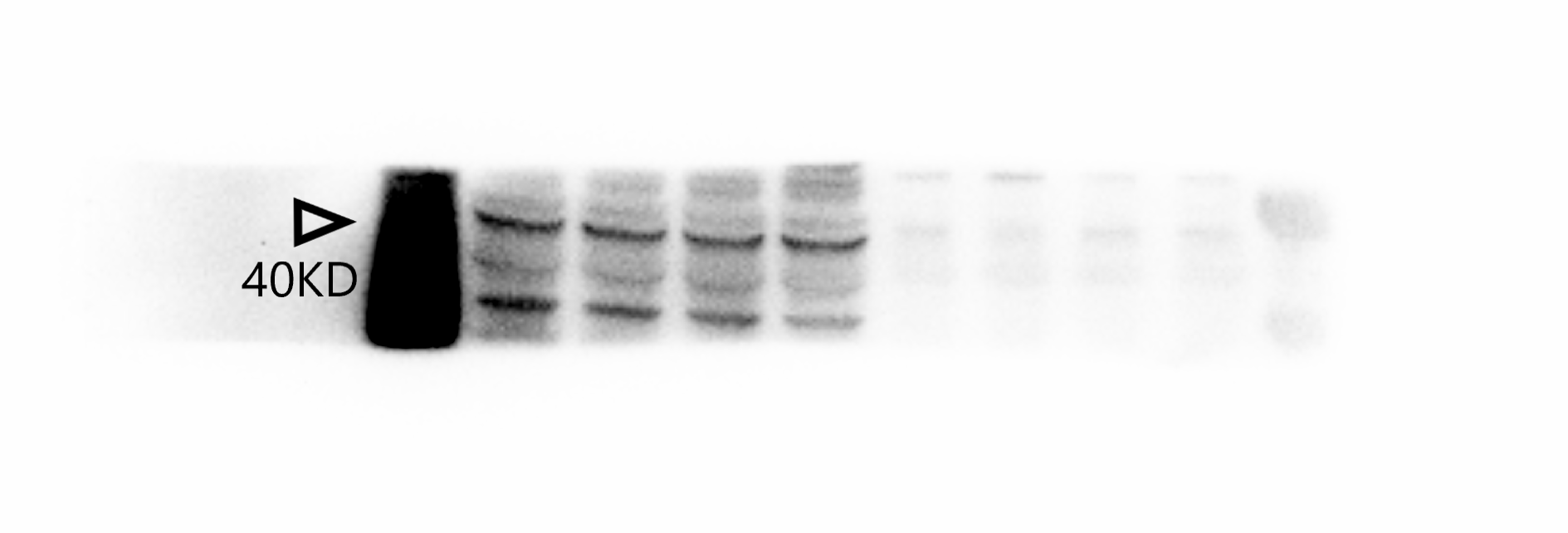

Supplement: Supplementary file 4 — Source Data [file 41467_2022_28500_MOESM4_ESM.zip › Source data/Fig5 B/PEBP4 (input+pd) (short exposure).tif]

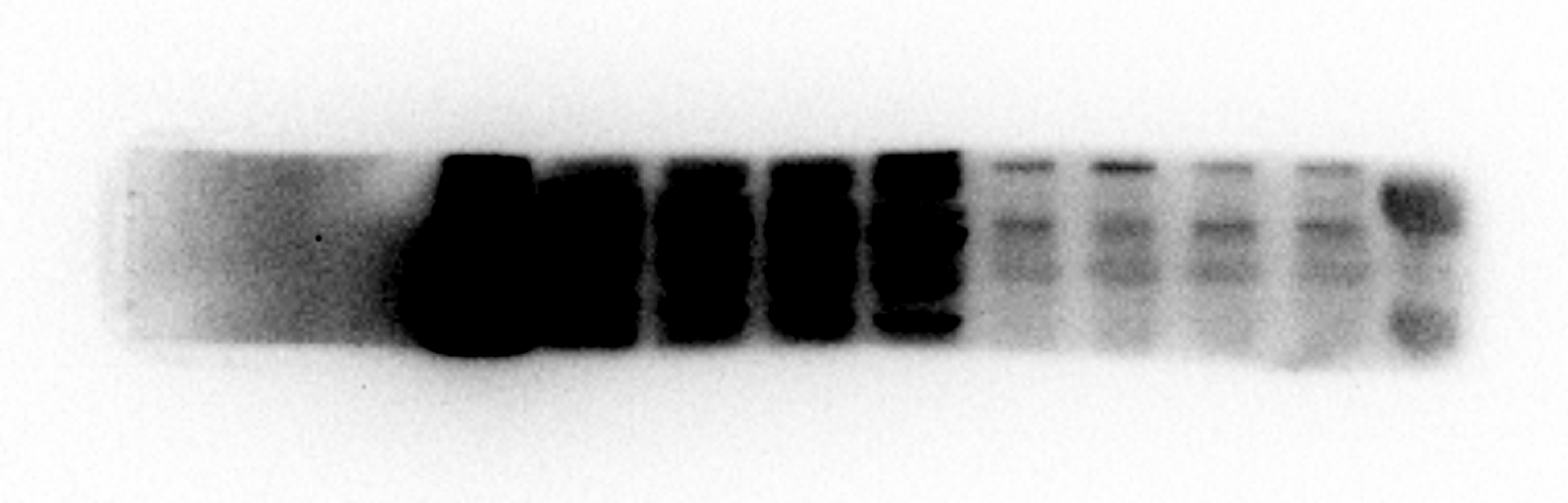

Supplement: Supplementary file 4 — Source Data [file 41467_2022_28500_MOESM4_ESM.zip › Source data/Fig5 B/PEBP4 (input+pd).tif]

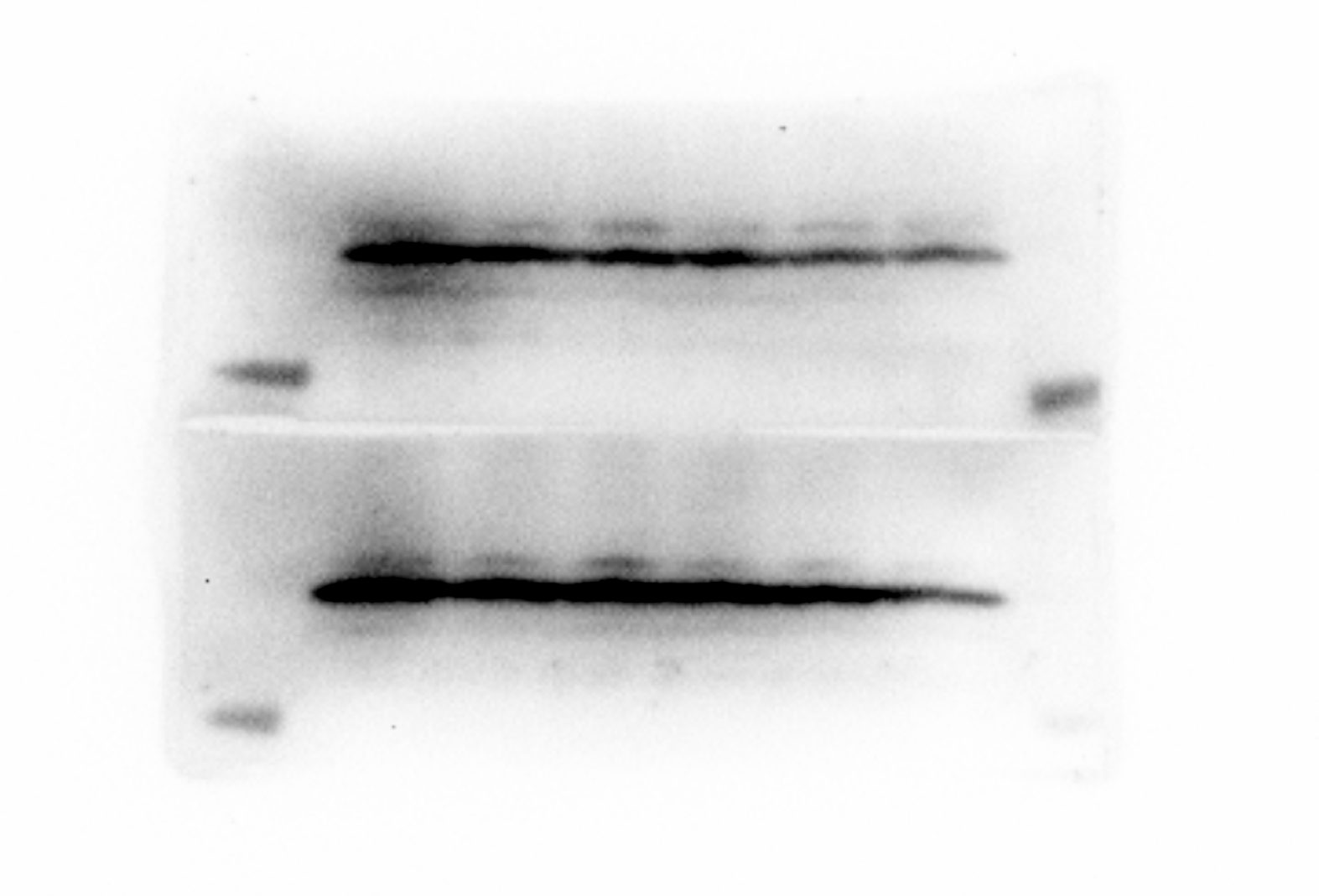

Supplement: Supplementary file 4 — Source Data [file 41467_2022_28500_MOESM4_ESM.zip › Source data/Fig5 C/ATG8.tif]

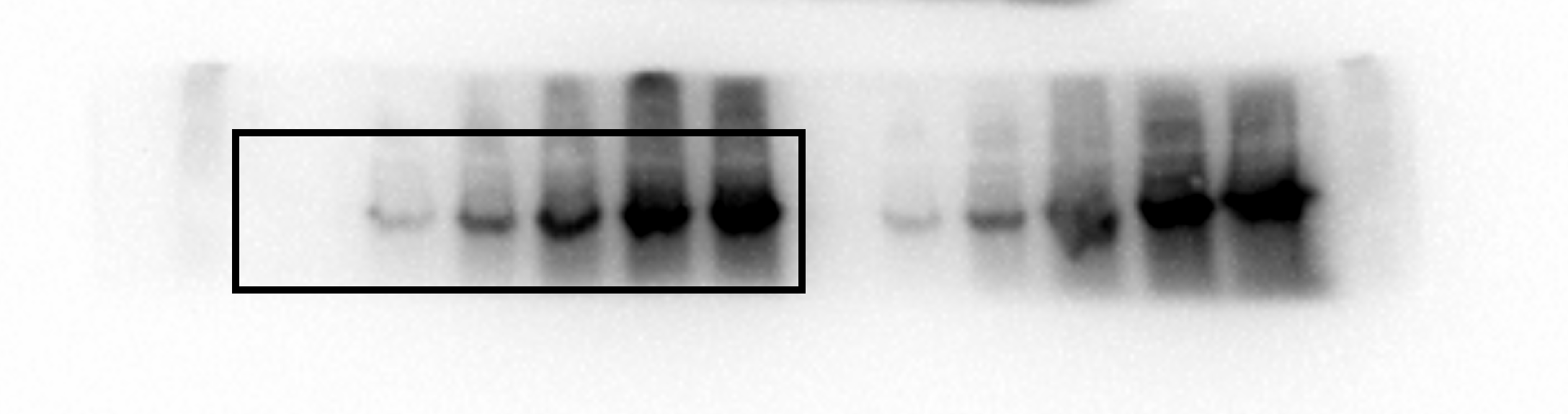

Supplement: Supplementary file 4 — Source Data [file 41467_2022_28500_MOESM4_ESM.zip › Source data/Fig5 C/CP.tif]

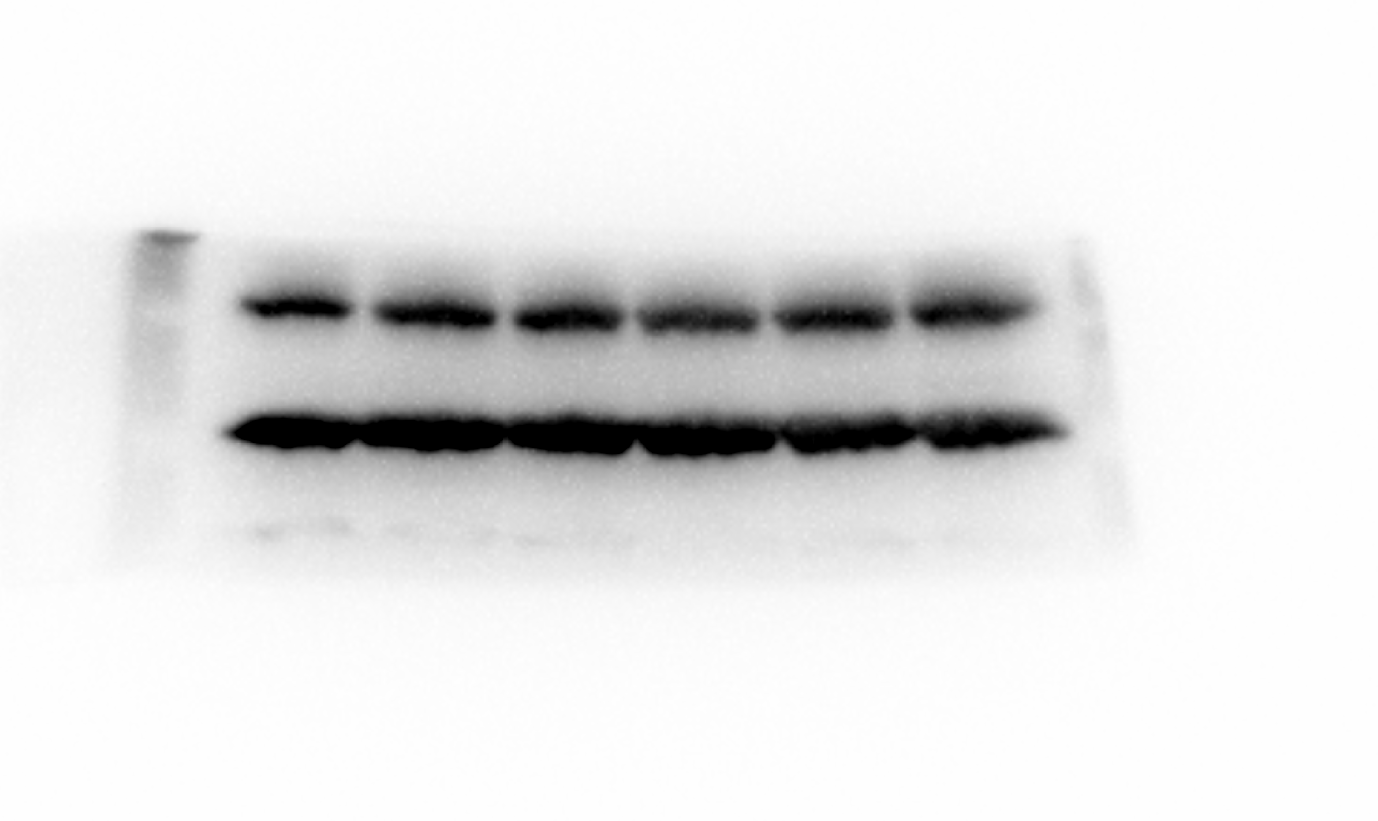

Supplement: Supplementary file 4 — Source Data [file 41467_2022_28500_MOESM4_ESM.zip › Source data/Fig5 C/GAPDH.tif]

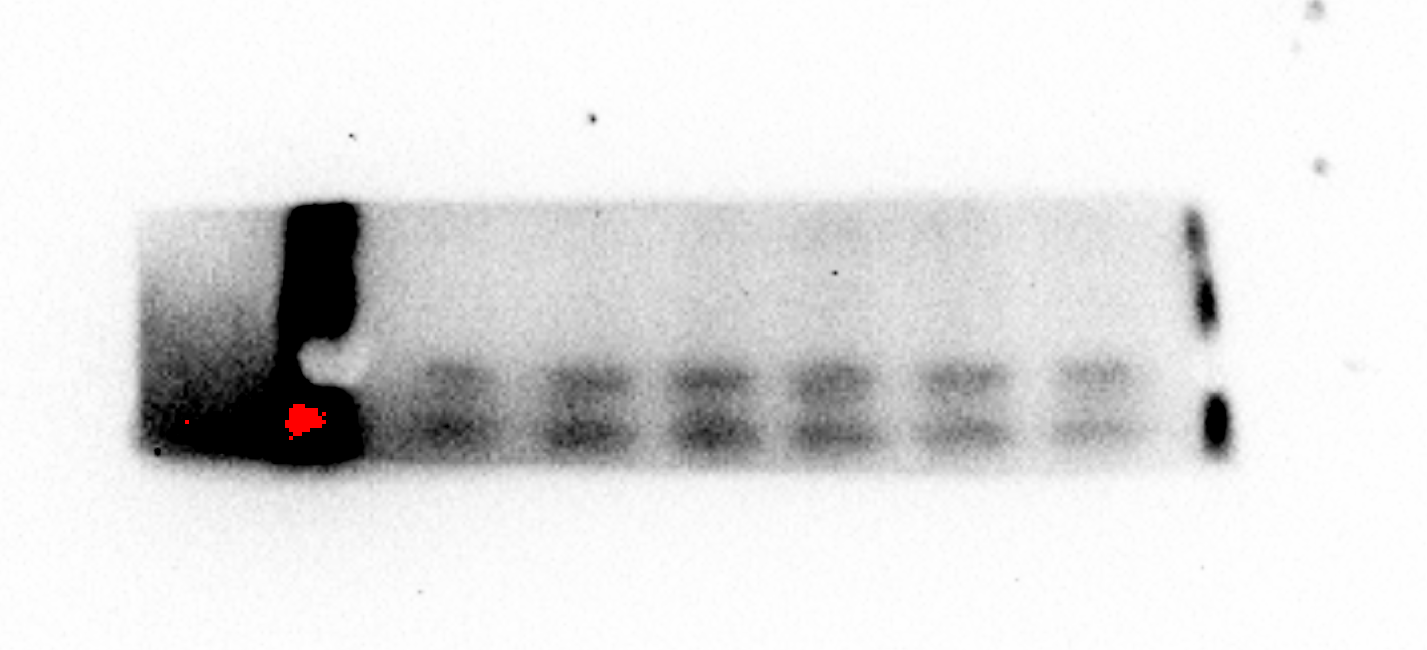

Supplement: Supplementary file 4 — Source Data [file 41467_2022_28500_MOESM4_ESM.zip › Source data/Fig5 C/SQSTM1.tif]

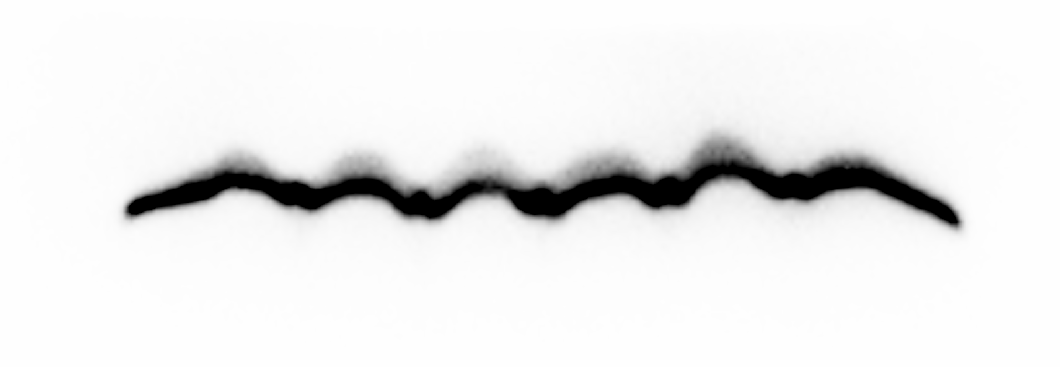

Supplement: Supplementary file 4 — Source Data [file 41467_2022_28500_MOESM4_ESM.zip › Source data/Fig5 D/ATG8.tif]

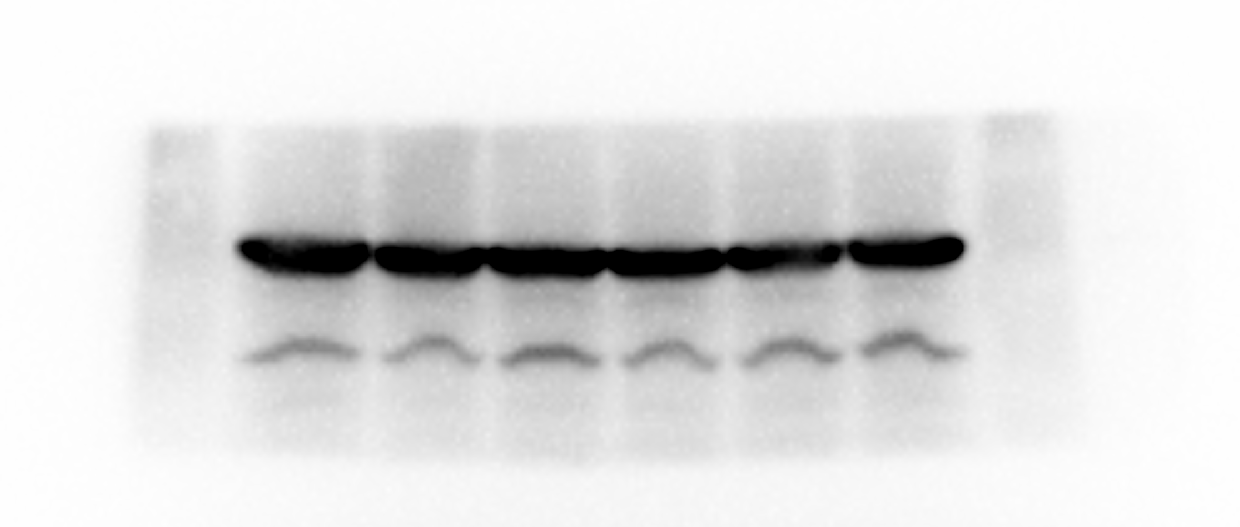

Supplement: Supplementary file 4 — Source Data [file 41467_2022_28500_MOESM4_ESM.zip › Source data/Fig5 D/GAPDH.tif]

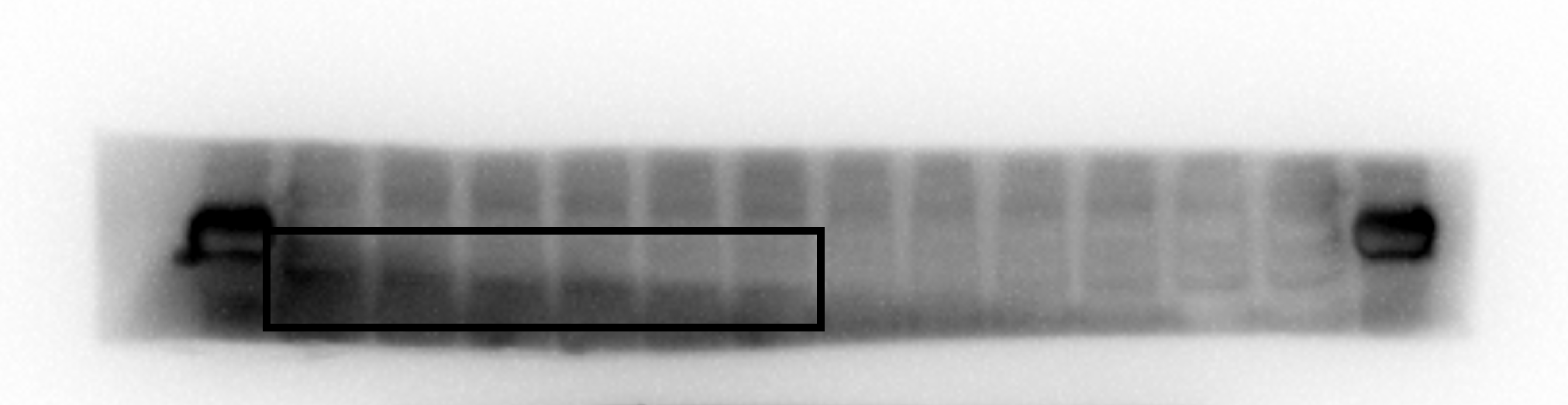

Supplement: Supplementary file 4 — Source Data [file 41467_2022_28500_MOESM4_ESM.zip › Source data/Fig5 D/PEBP4.tif]

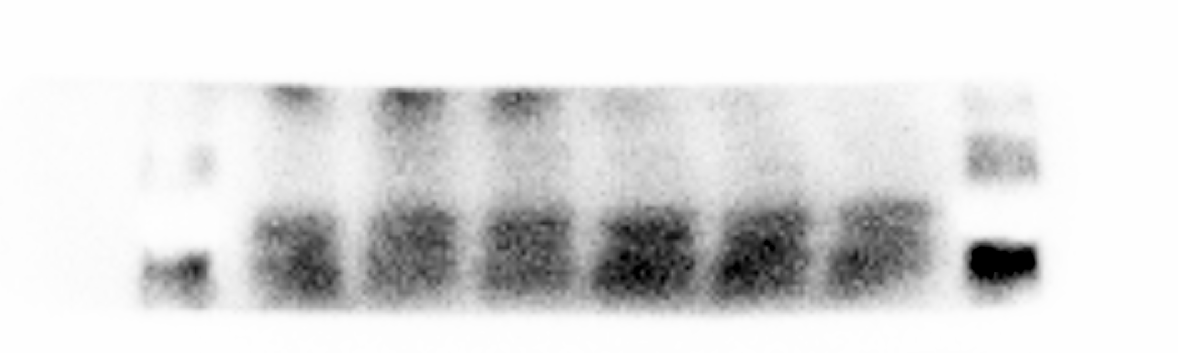

Supplement: Supplementary file 4 — Source Data [file 41467_2022_28500_MOESM4_ESM.zip › Source data/Fig5 D/SQSTM1.tif]

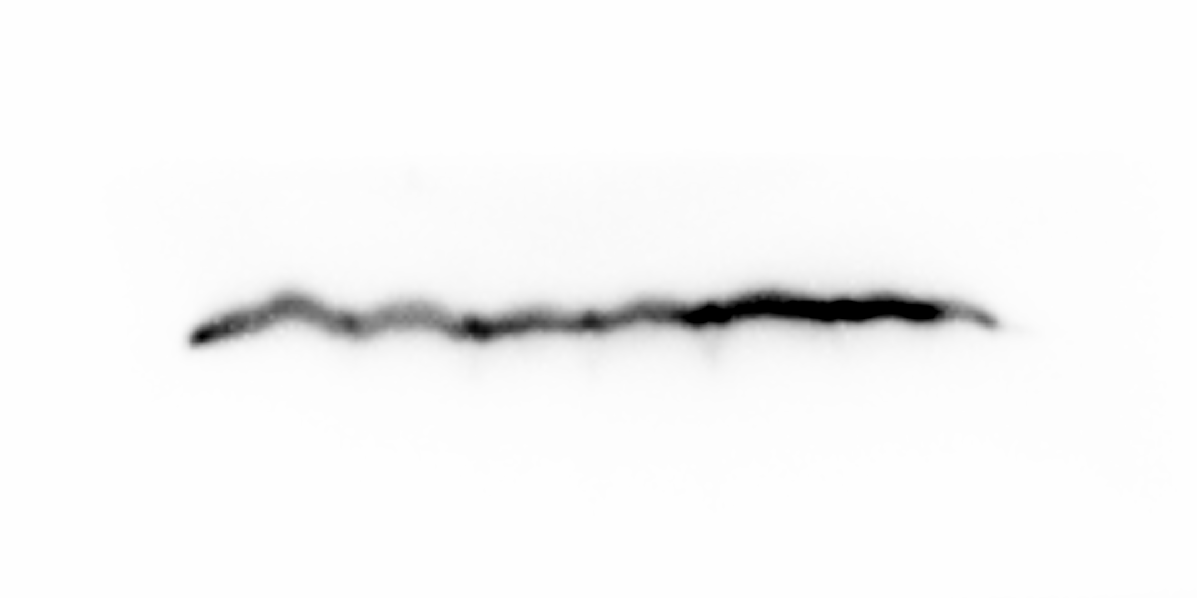

Supplement: Supplementary file 4 — Source Data [file 41467_2022_28500_MOESM4_ESM.zip › Source data/Fig5 E/ATG8.tif]

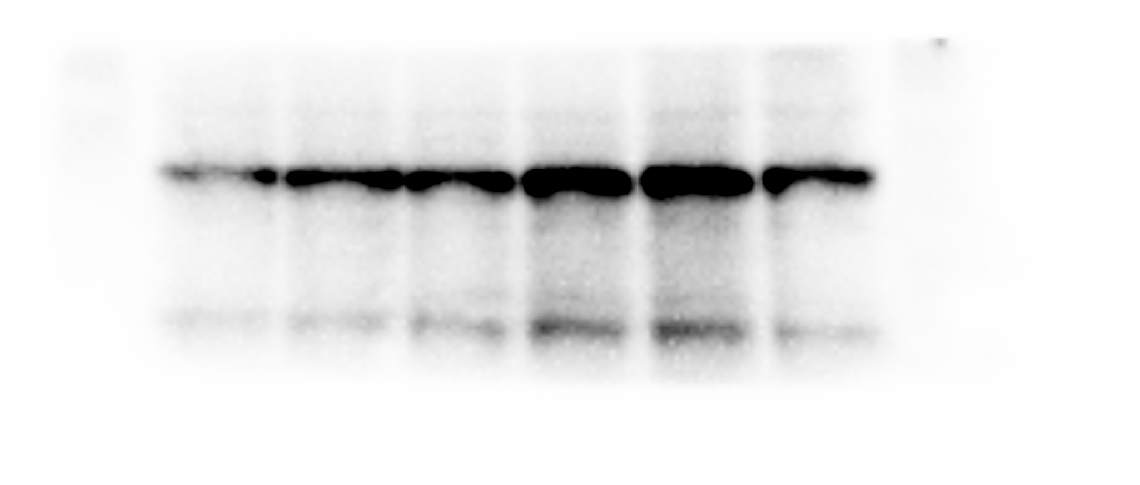

Supplement: Supplementary file 4 — Source Data [file 41467_2022_28500_MOESM4_ESM.zip › Source data/Fig5 E/CP.tif]

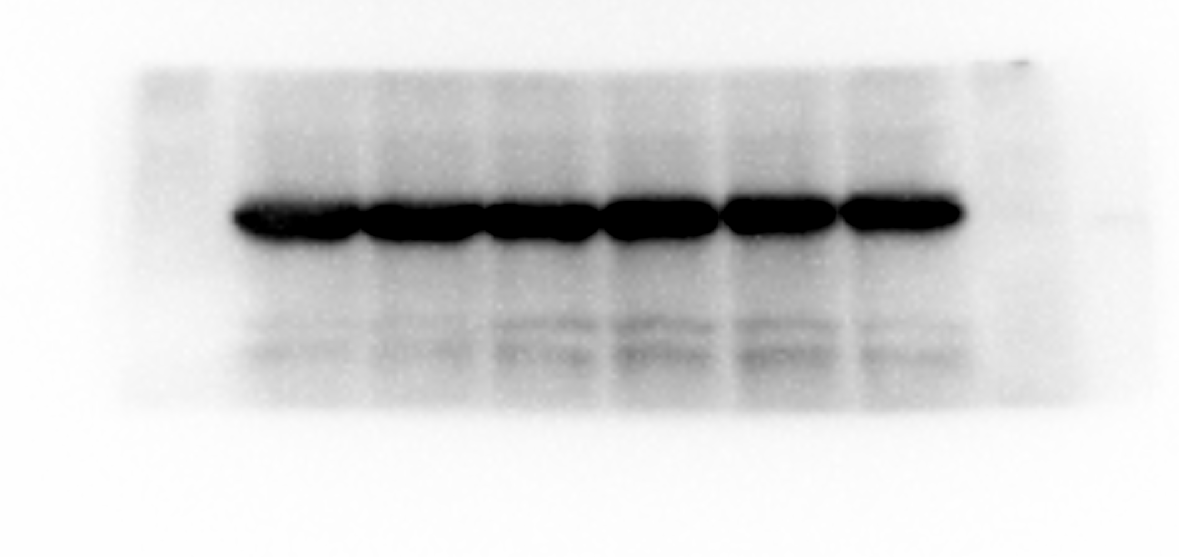

Supplement: Supplementary file 4 — Source Data [file 41467_2022_28500_MOESM4_ESM.zip › Source data/Fig5 E/GAPDH.tif]

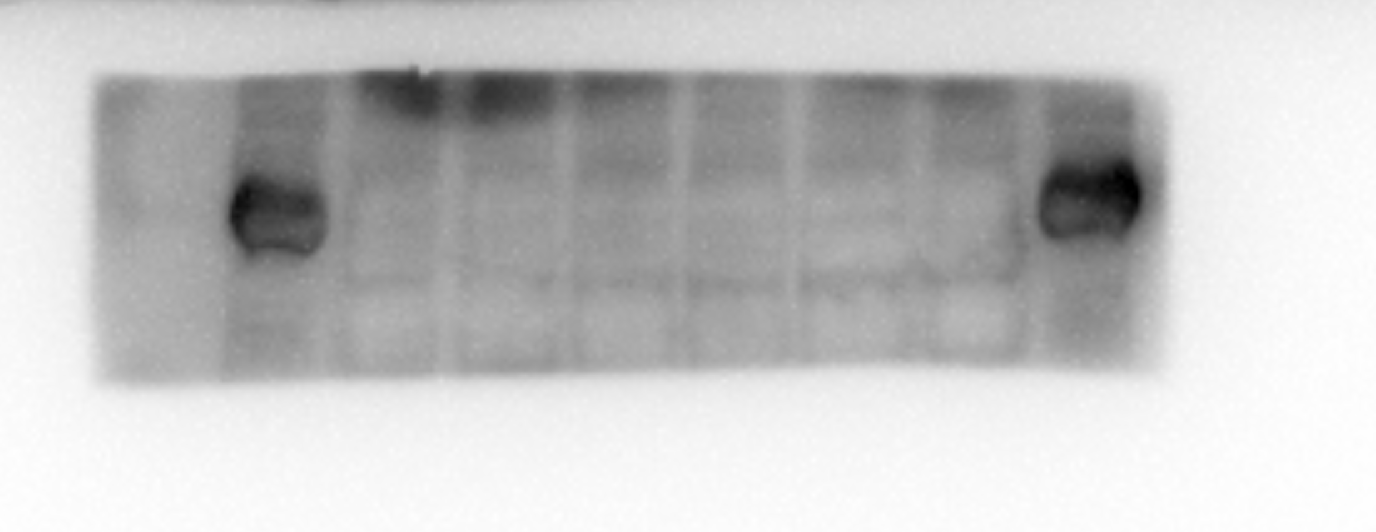

Supplement: Supplementary file 4 — Source Data [file 41467_2022_28500_MOESM4_ESM.zip › Source data/Fig5 E/GST-PEBP4.tif]

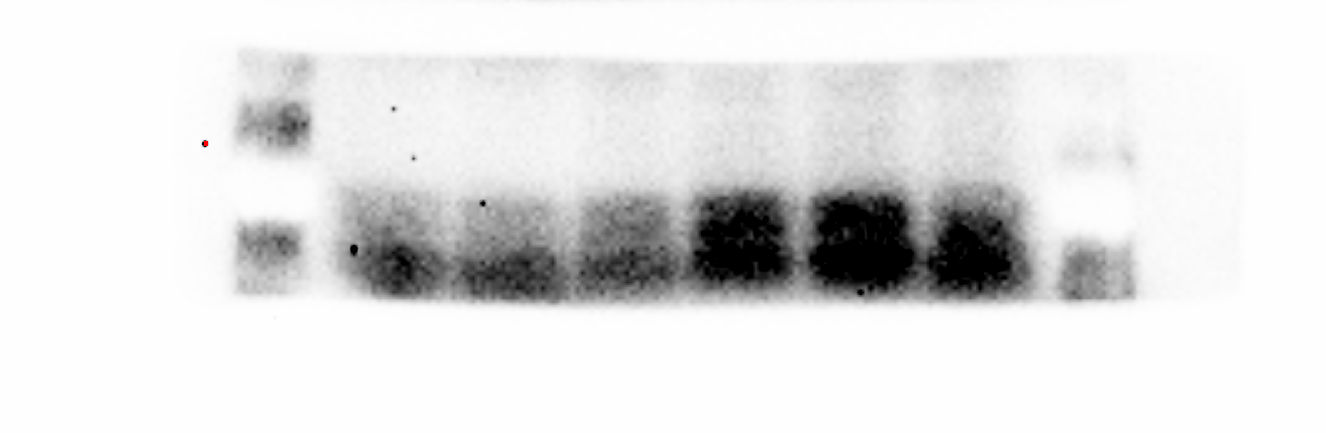

Supplement: Supplementary file 4 — Source Data [file 41467_2022_28500_MOESM4_ESM.zip › Source data/Fig5 E/SQSTM1.tif]

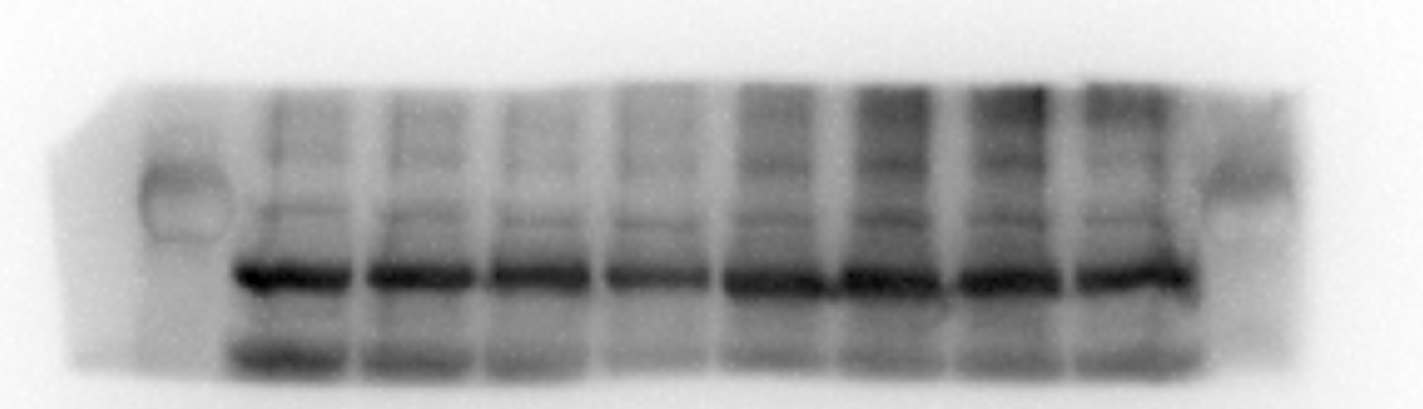

Supplement: Supplementary file 4 — Source Data [file 41467_2022_28500_MOESM4_ESM.zip › Source data/Fig5 F/Input-GSTPEBP4.tif]
