# Supplementary figures and images for: PEBP balances apoptosis and autophagy in whitefly upon arbovirus infection (part 1 of 2)
Source: Nat Commun. 2022 Feb 11;13:846. doi: 10.1038/s41467-022-28500-8 (PMC8837789; doi:10.1038/s41467-022-28500-8)

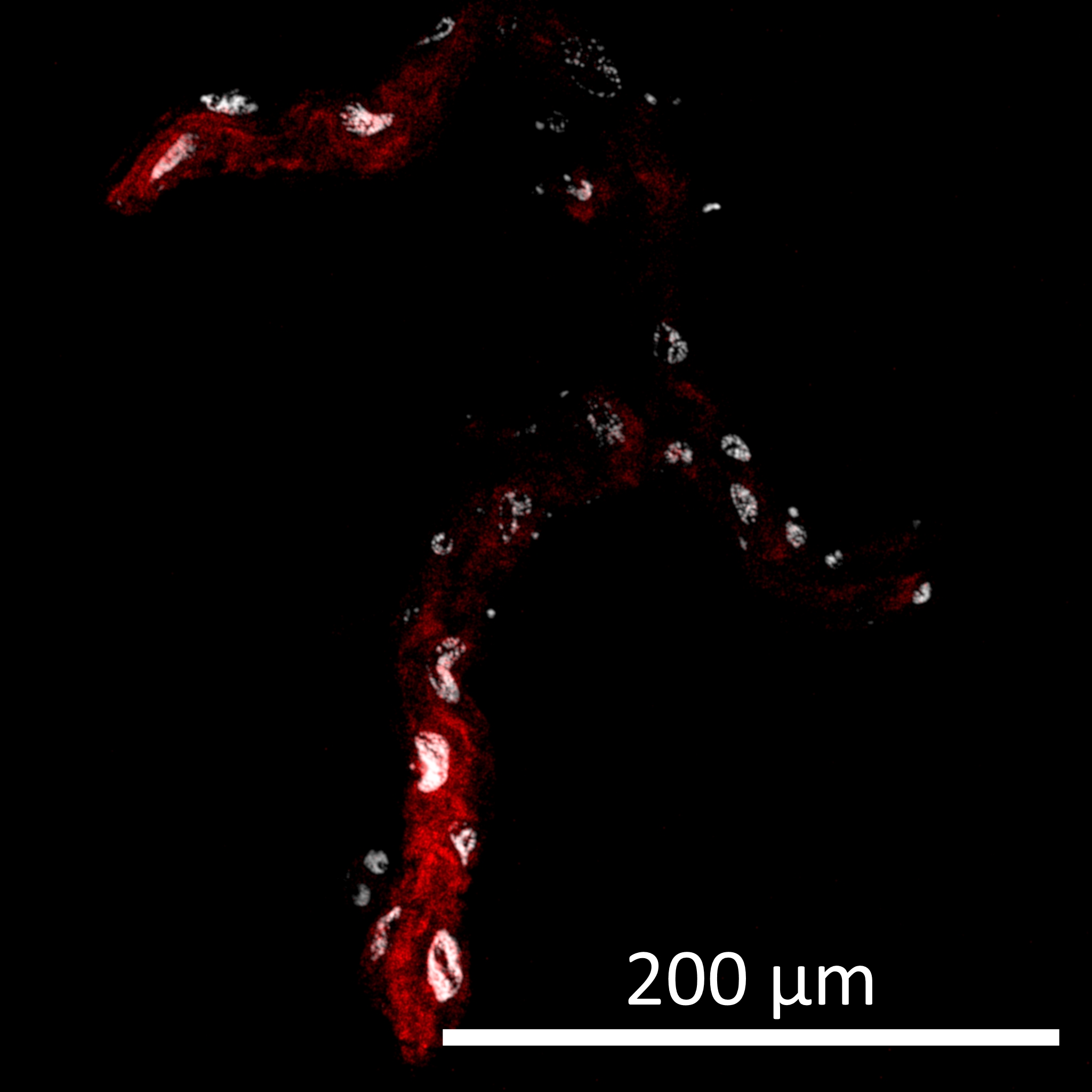

Supplement: Supplementary file 4 — Source Data [file 41467_2022_28500_MOESM4_ESM.zip › Source data/Fig1 A,B/ATG8(CP)-MG_c1+3.jpg]

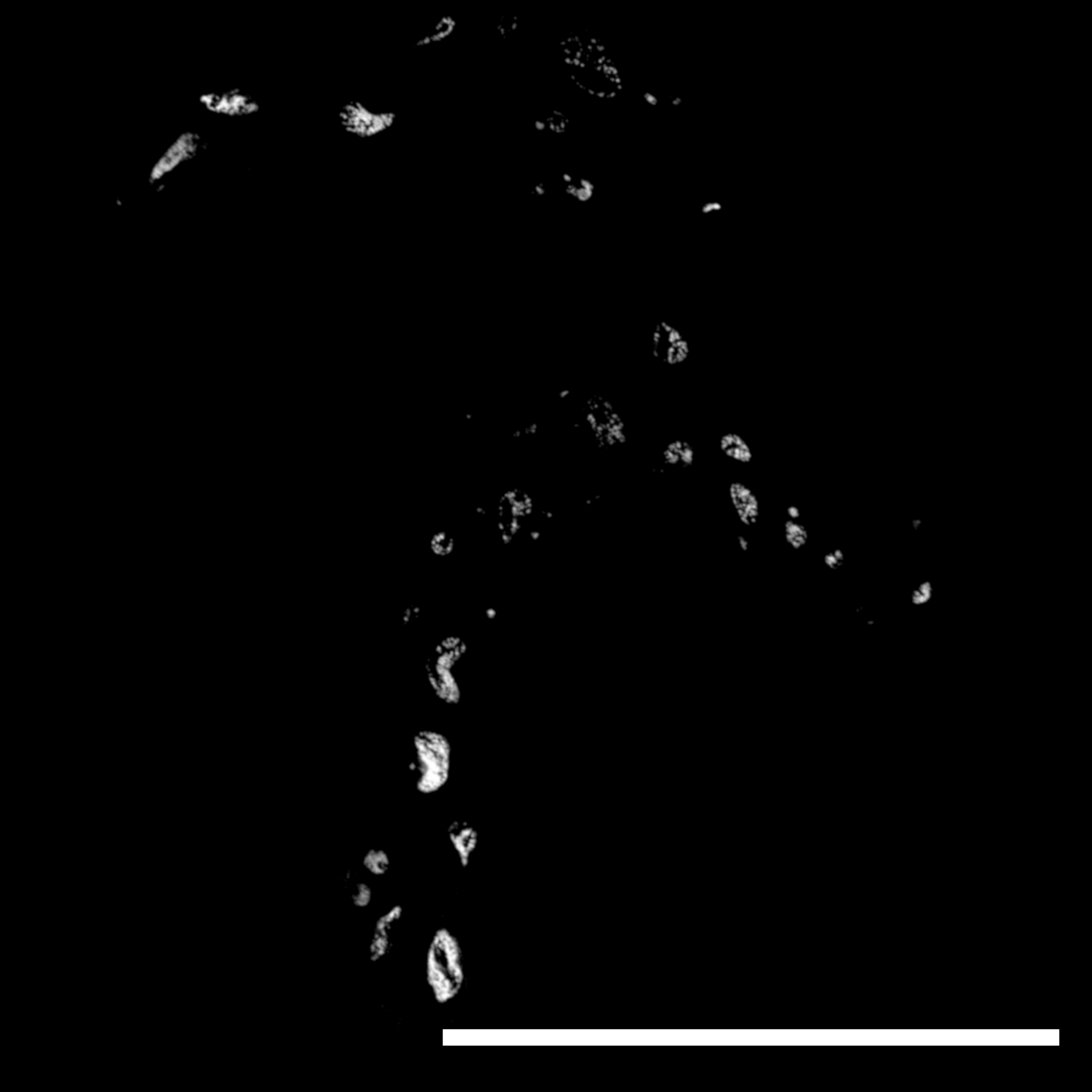

Supplement: Supplementary file 4 — Source Data [file 41467_2022_28500_MOESM4_ESM.zip › Source data/Fig1 A,B/ATG8(CP)-MG_c1.jpg]

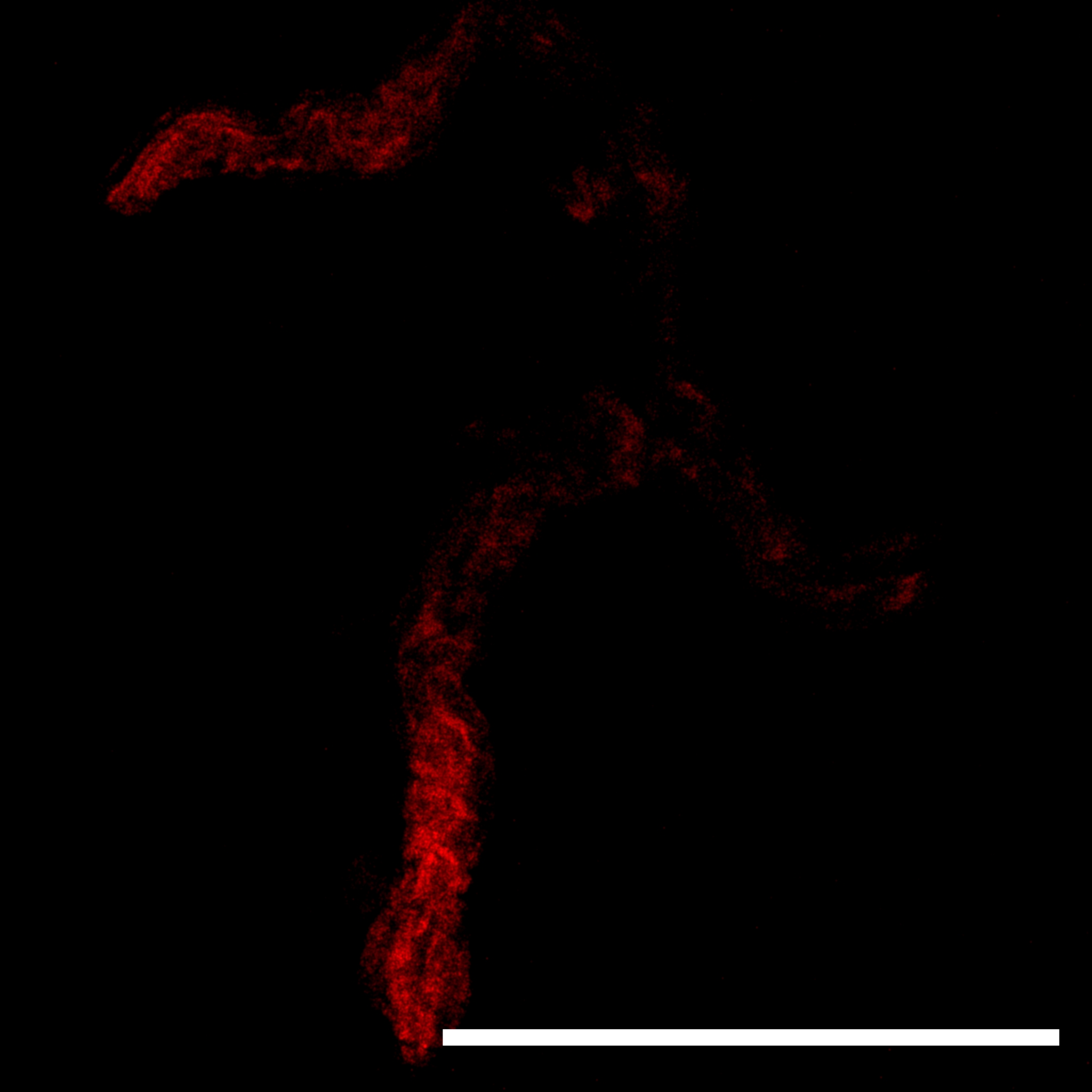

Supplement: Supplementary file 4 — Source Data [file 41467_2022_28500_MOESM4_ESM.zip › Source data/Fig1 A,B/ATG8(CP)-MG_c3.jpg]

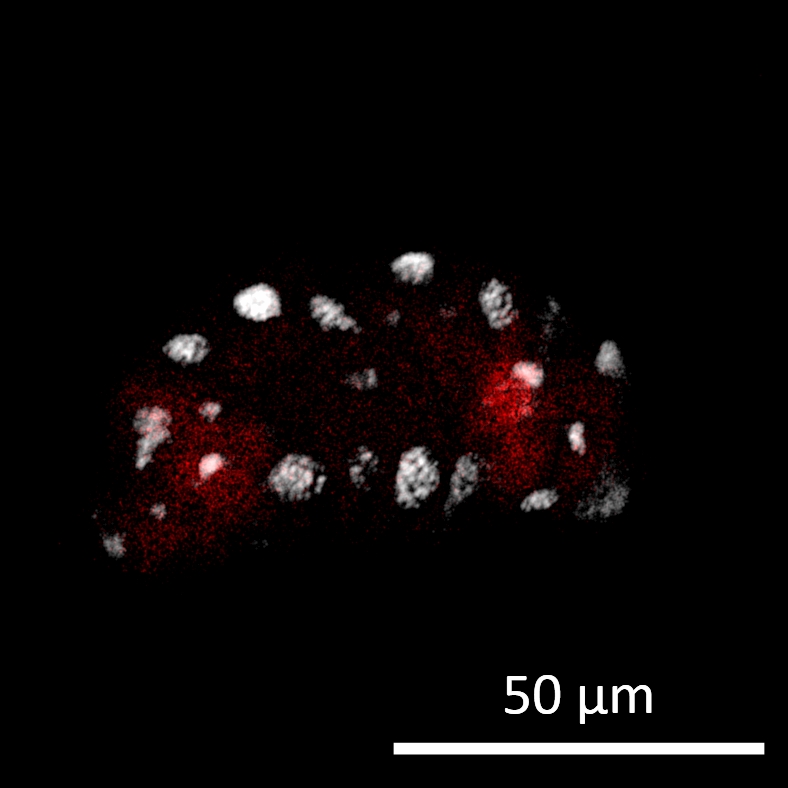

Supplement: Supplementary file 4 — Source Data [file 41467_2022_28500_MOESM4_ESM.zip › Source data/Fig1 A,B/ATG8(CP)-SG_c1+3.jpg]

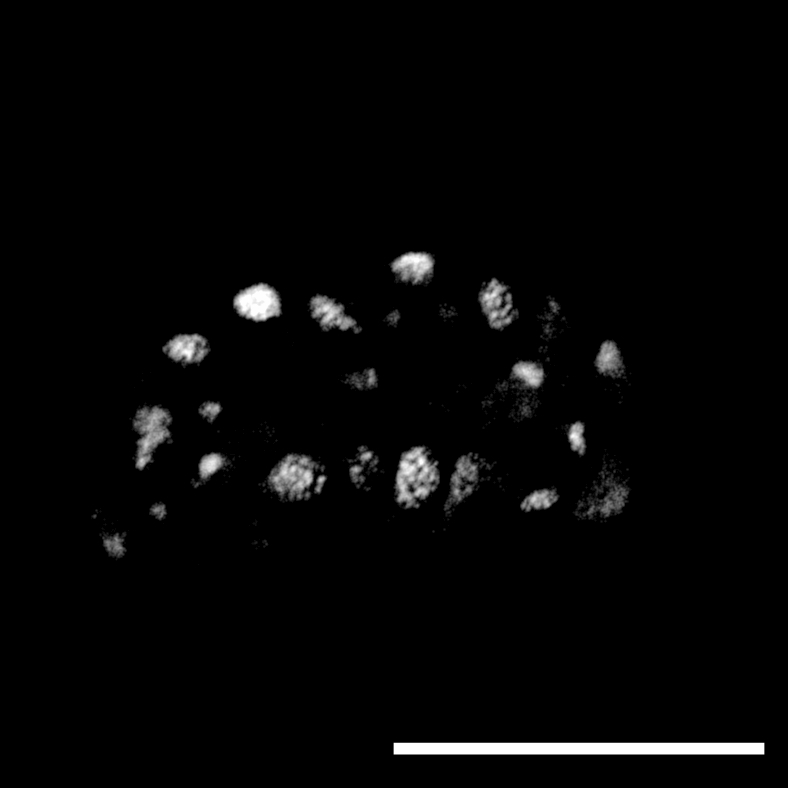

Supplement: Supplementary file 4 — Source Data [file 41467_2022_28500_MOESM4_ESM.zip › Source data/Fig1 A,B/ATG8(CP)-SG_c1.jpg]

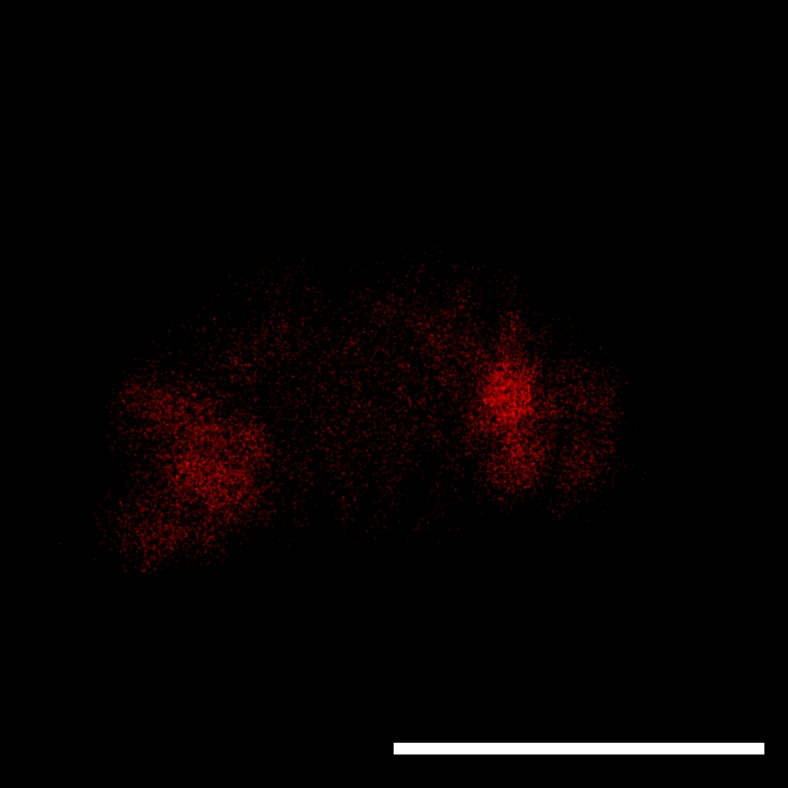

Supplement: Supplementary file 4 — Source Data [file 41467_2022_28500_MOESM4_ESM.zip › Source data/Fig1 A,B/ATG8(CP)-SG_c3.jpg]

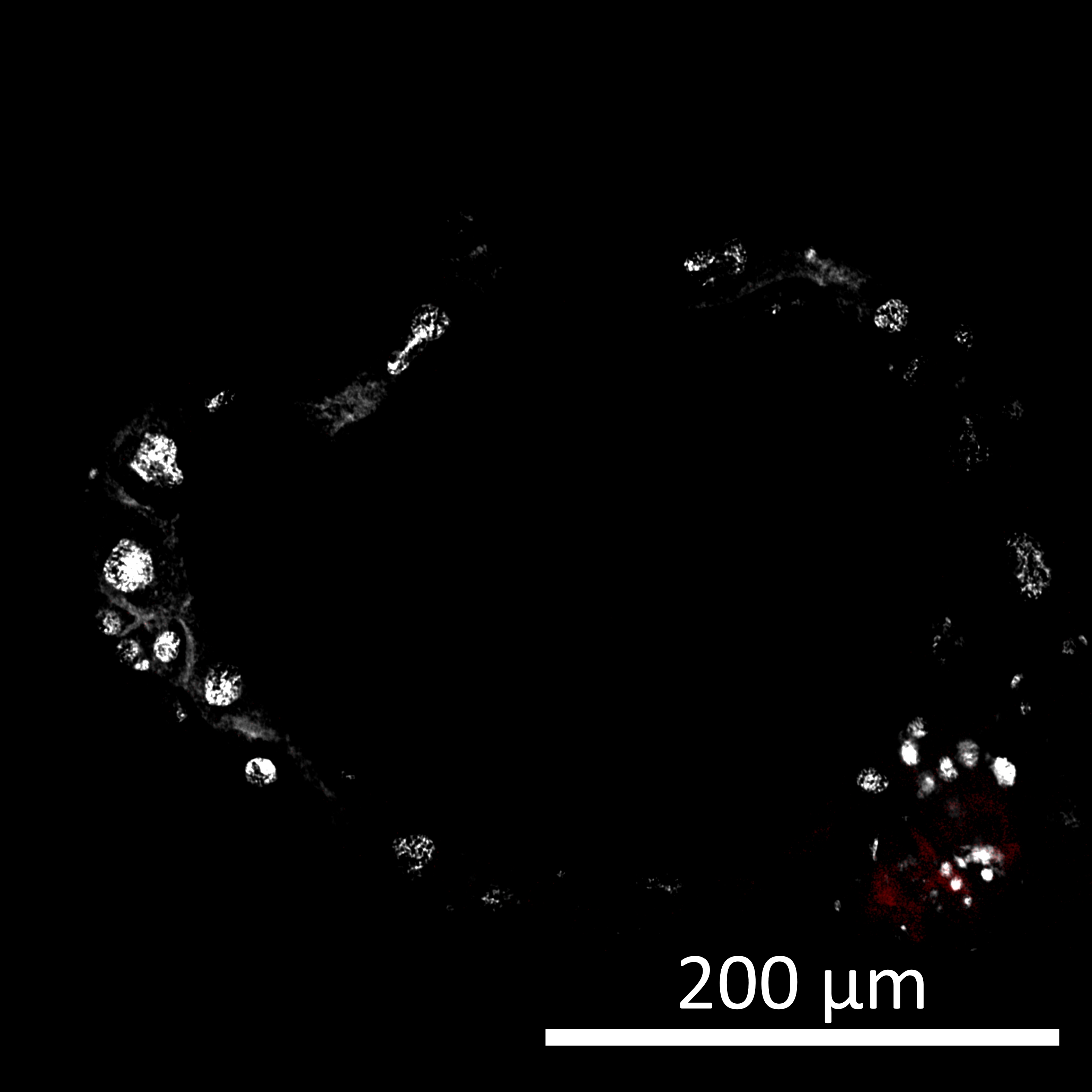

Supplement: Supplementary file 4 — Source Data [file 41467_2022_28500_MOESM4_ESM.zip › Source data/Fig1 A,B/ATG8(NV)-MG_c1+3.jpg]

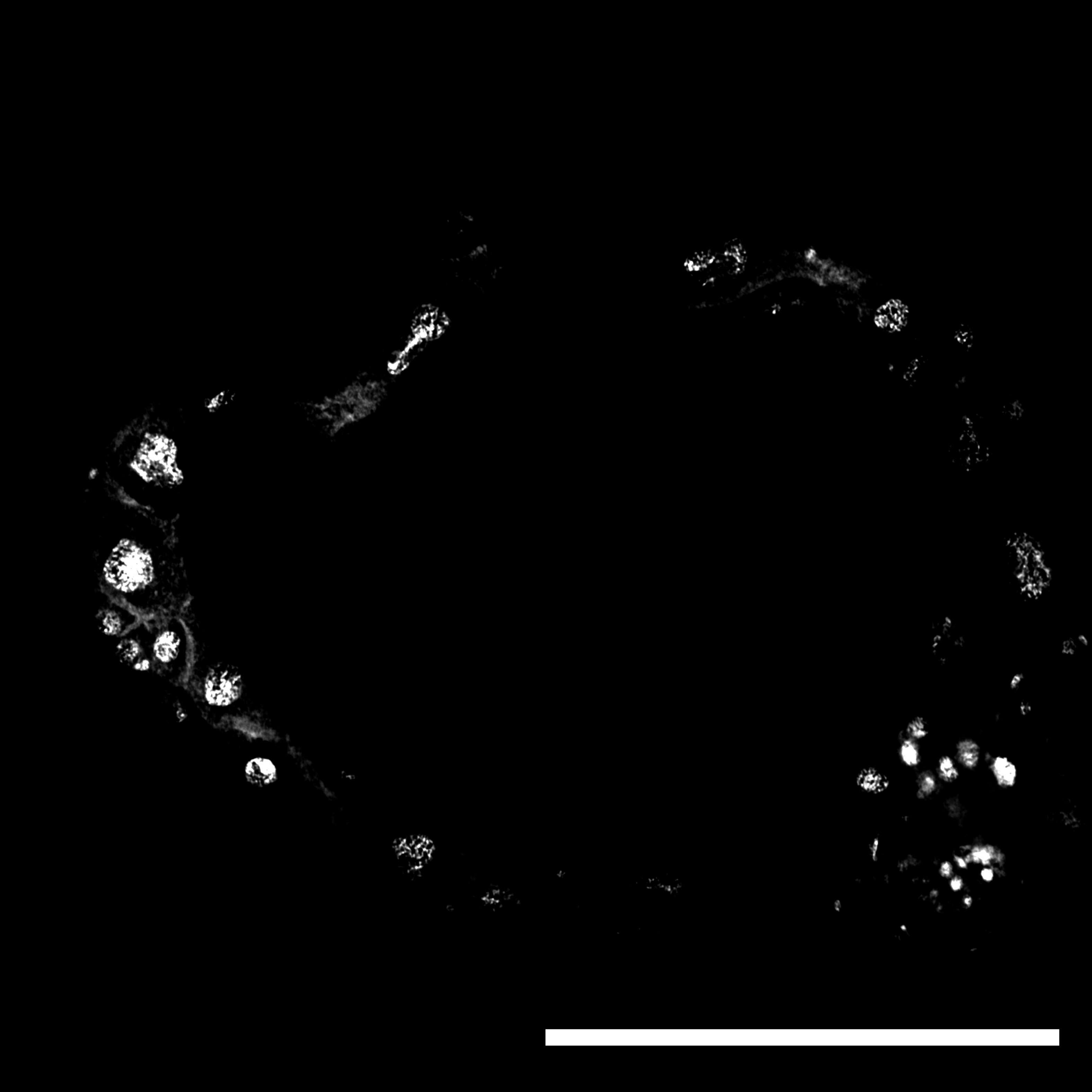

Supplement: Supplementary file 4 — Source Data [file 41467_2022_28500_MOESM4_ESM.zip › Source data/Fig1 A,B/ATG8(NV)-MG_c1.jpg]

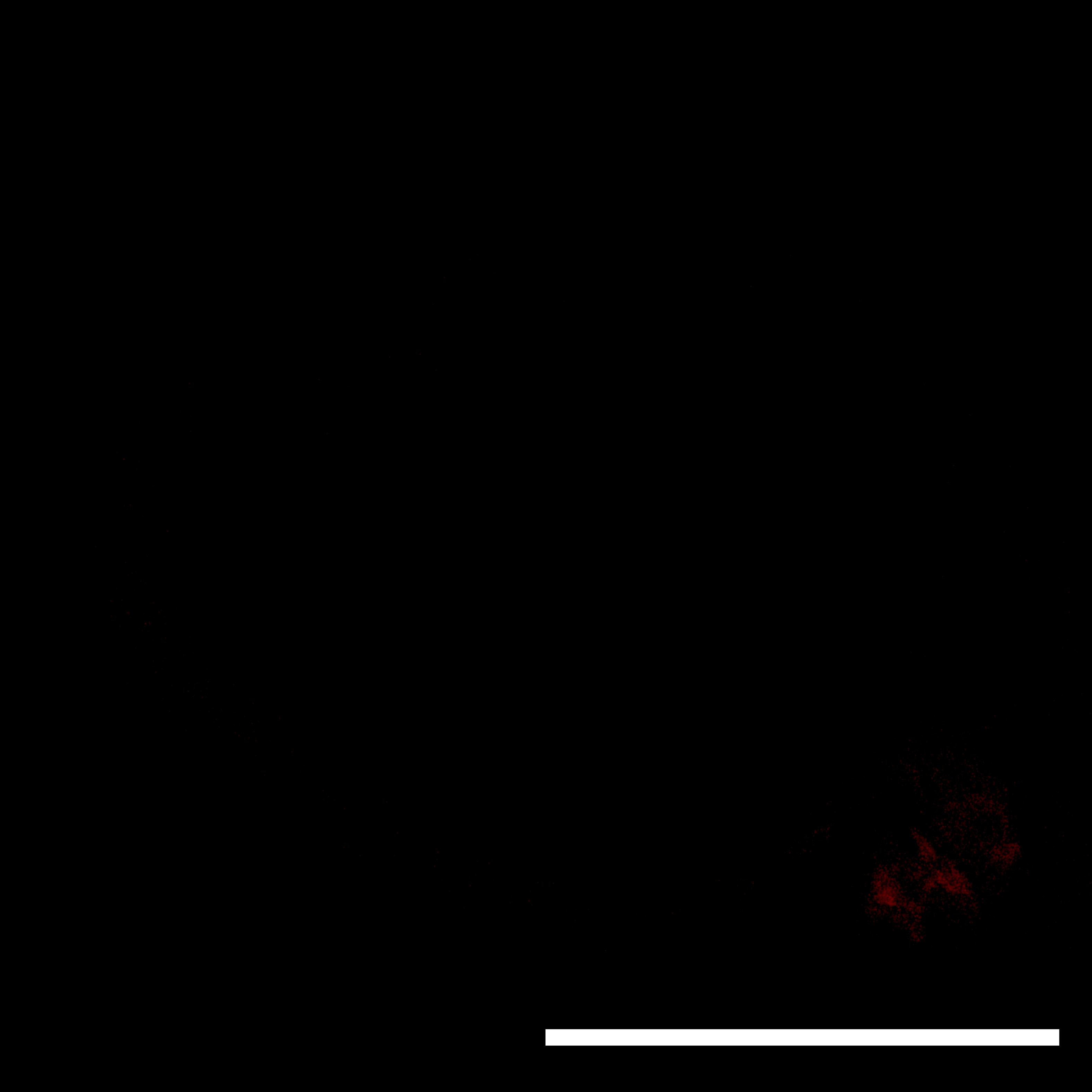

Supplement: Supplementary file 4 — Source Data [file 41467_2022_28500_MOESM4_ESM.zip › Source data/Fig1 A,B/ATG8(NV)-MG_c3.jpg]

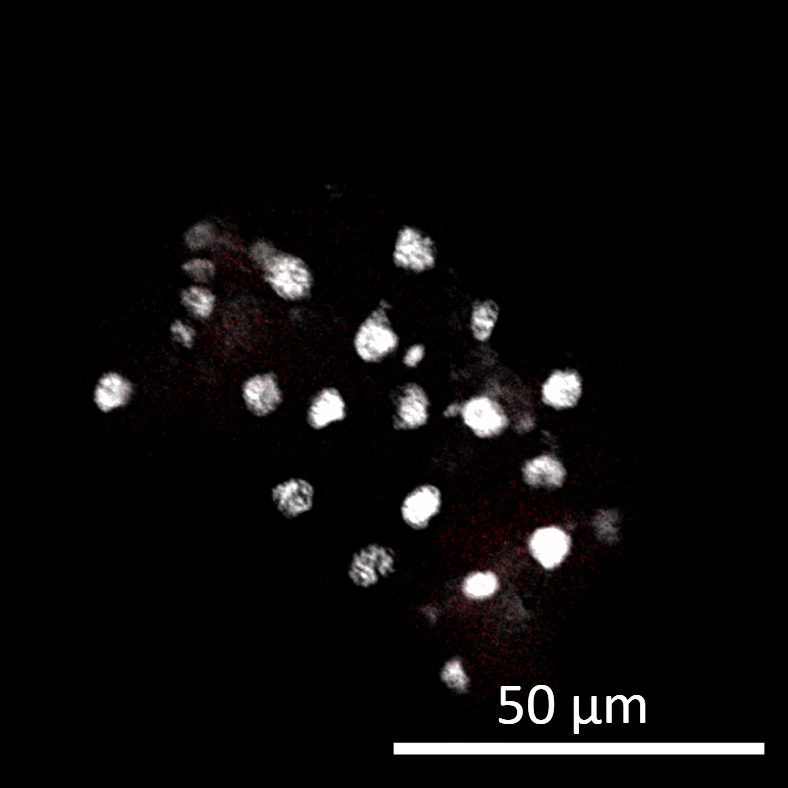

Supplement: Supplementary file 4 — Source Data [file 41467_2022_28500_MOESM4_ESM.zip › Source data/Fig1 A,B/ATG8(NV)-SG_c1+2+3.jpg]

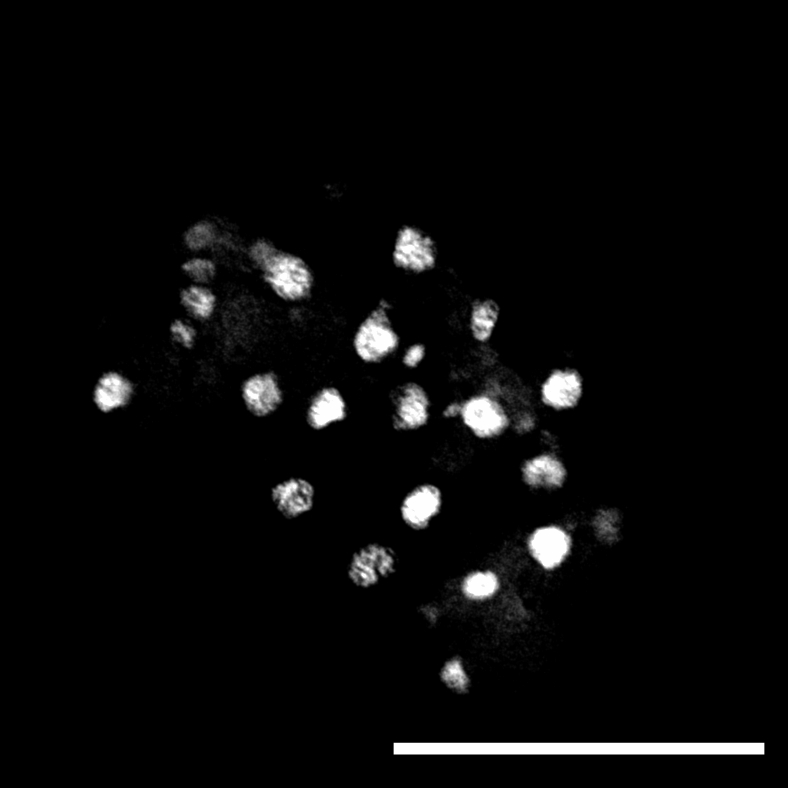

Supplement: Supplementary file 4 — Source Data [file 41467_2022_28500_MOESM4_ESM.zip › Source data/Fig1 A,B/ATG8(NV)-SG_c1.jpg]

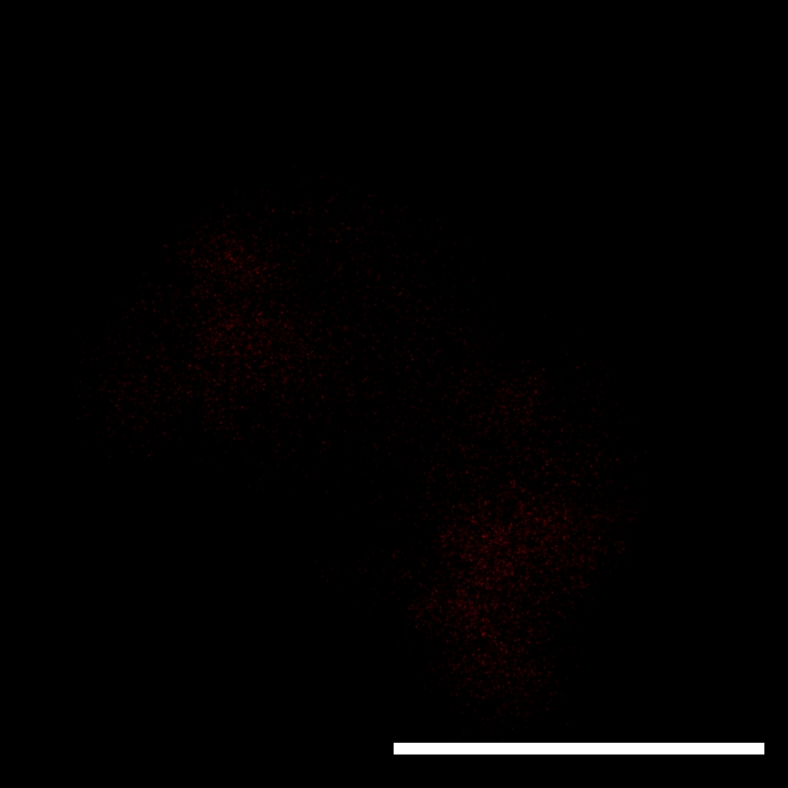

Supplement: Supplementary file 4 — Source Data [file 41467_2022_28500_MOESM4_ESM.zip › Source data/Fig1 A,B/ATG8(NV)-SG_c3.jpg]

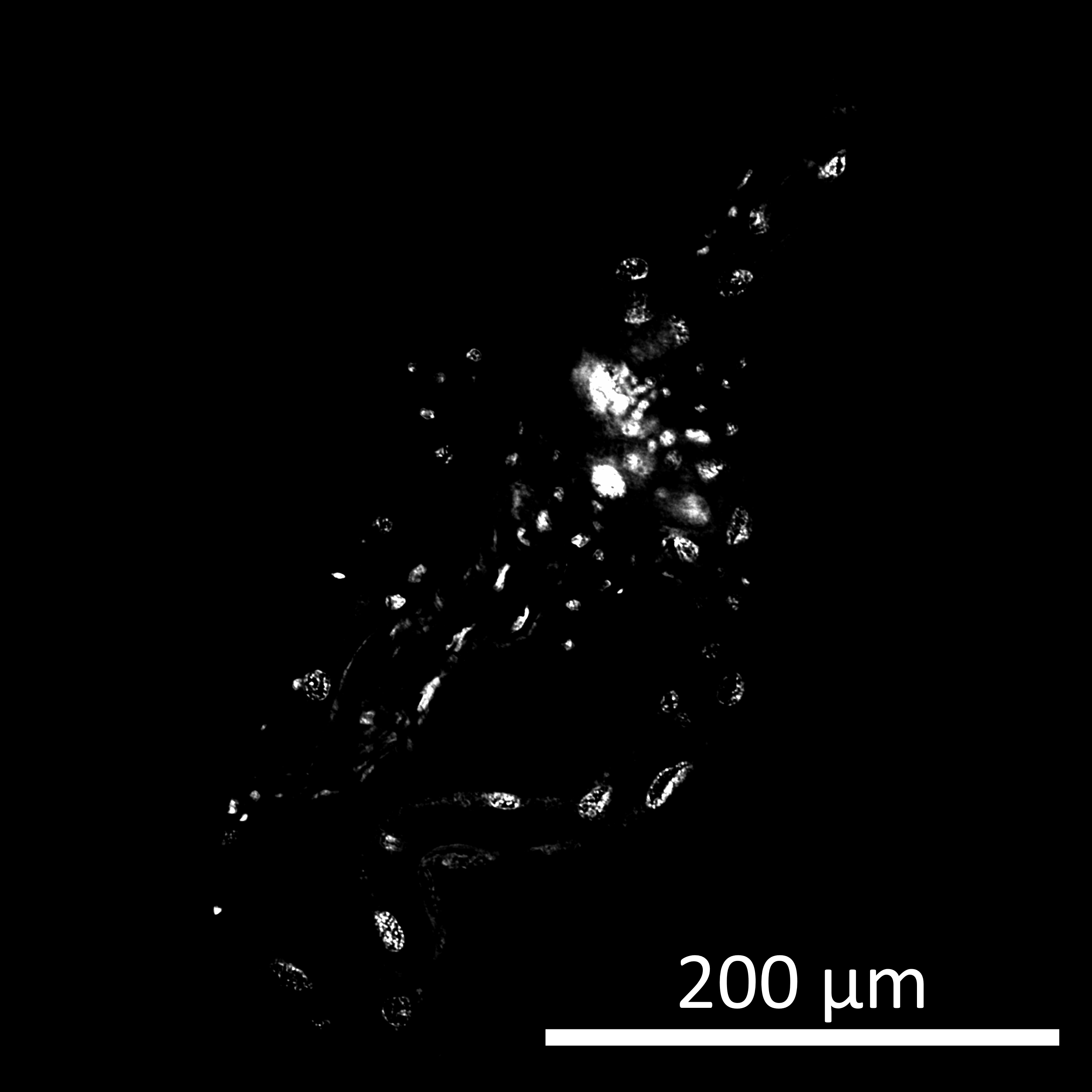

Supplement: Supplementary file 4 — Source Data [file 41467_2022_28500_MOESM4_ESM.zip › Source data/Fig1 A,B/MG-CTL_c1+2.jpg]

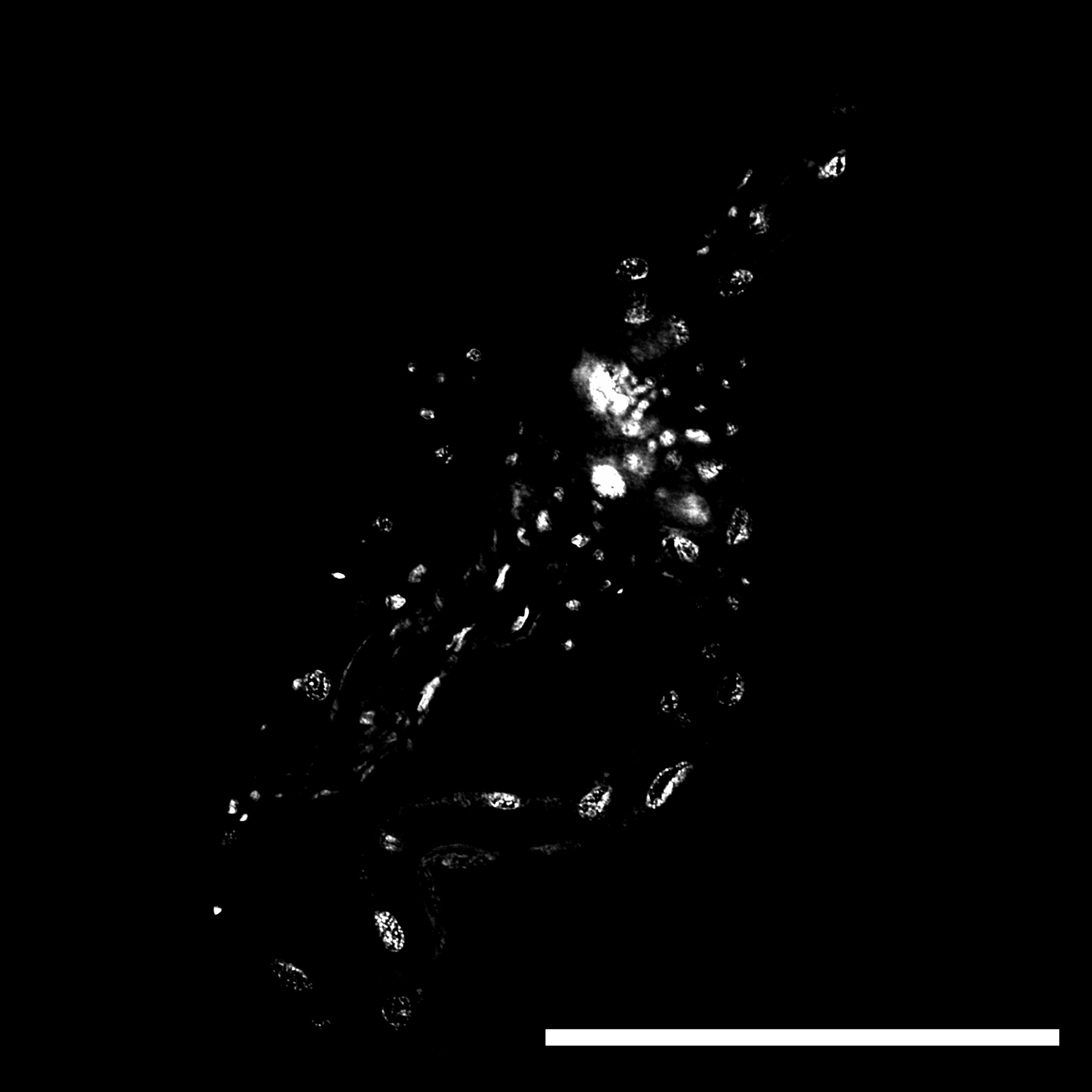

Supplement: Supplementary file 4 — Source Data [file 41467_2022_28500_MOESM4_ESM.zip › Source data/Fig1 A,B/MG-CTL_c1.jpg]

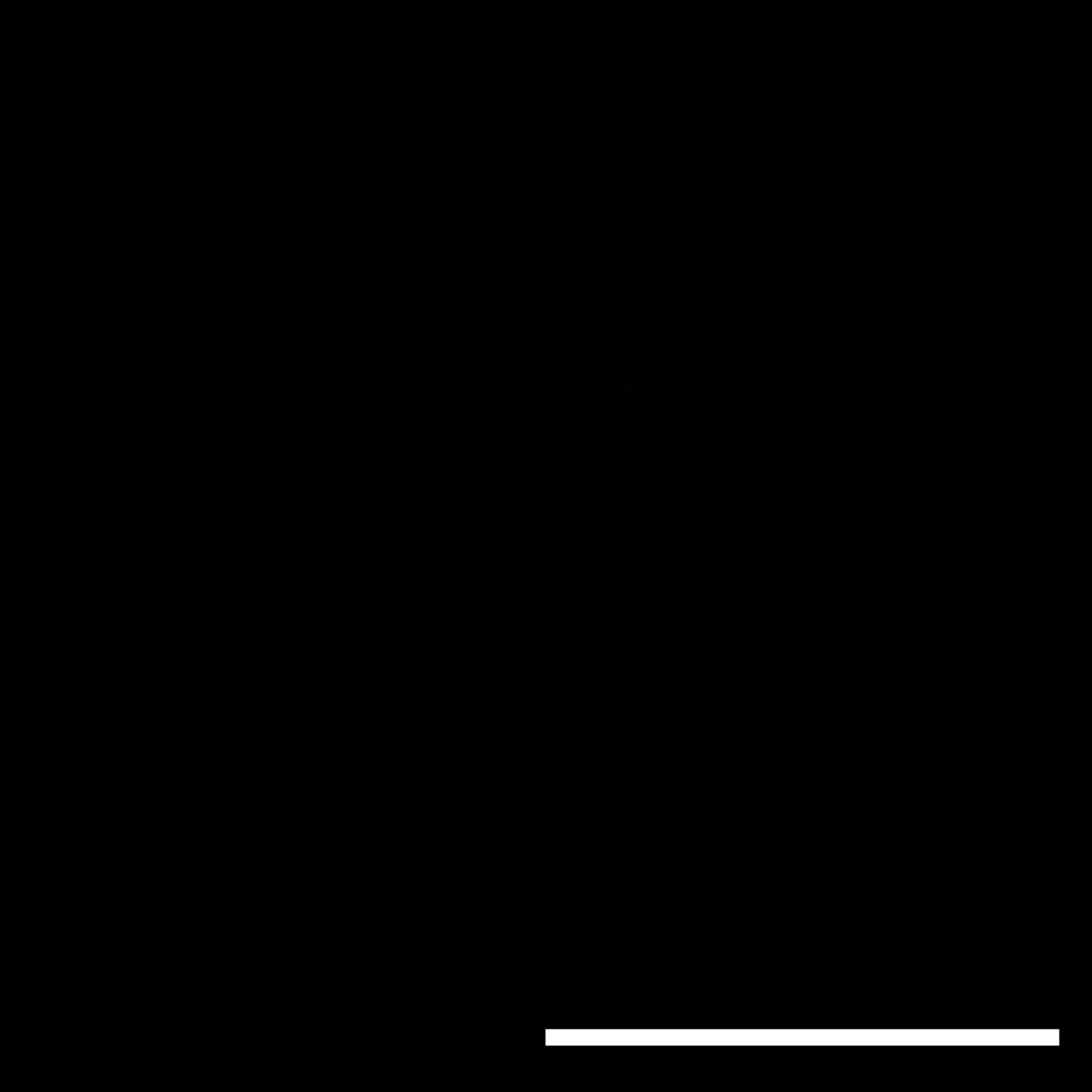

Supplement: Supplementary file 4 — Source Data [file 41467_2022_28500_MOESM4_ESM.zip › Source data/Fig1 A,B/MG-CTL_c2.jpg]

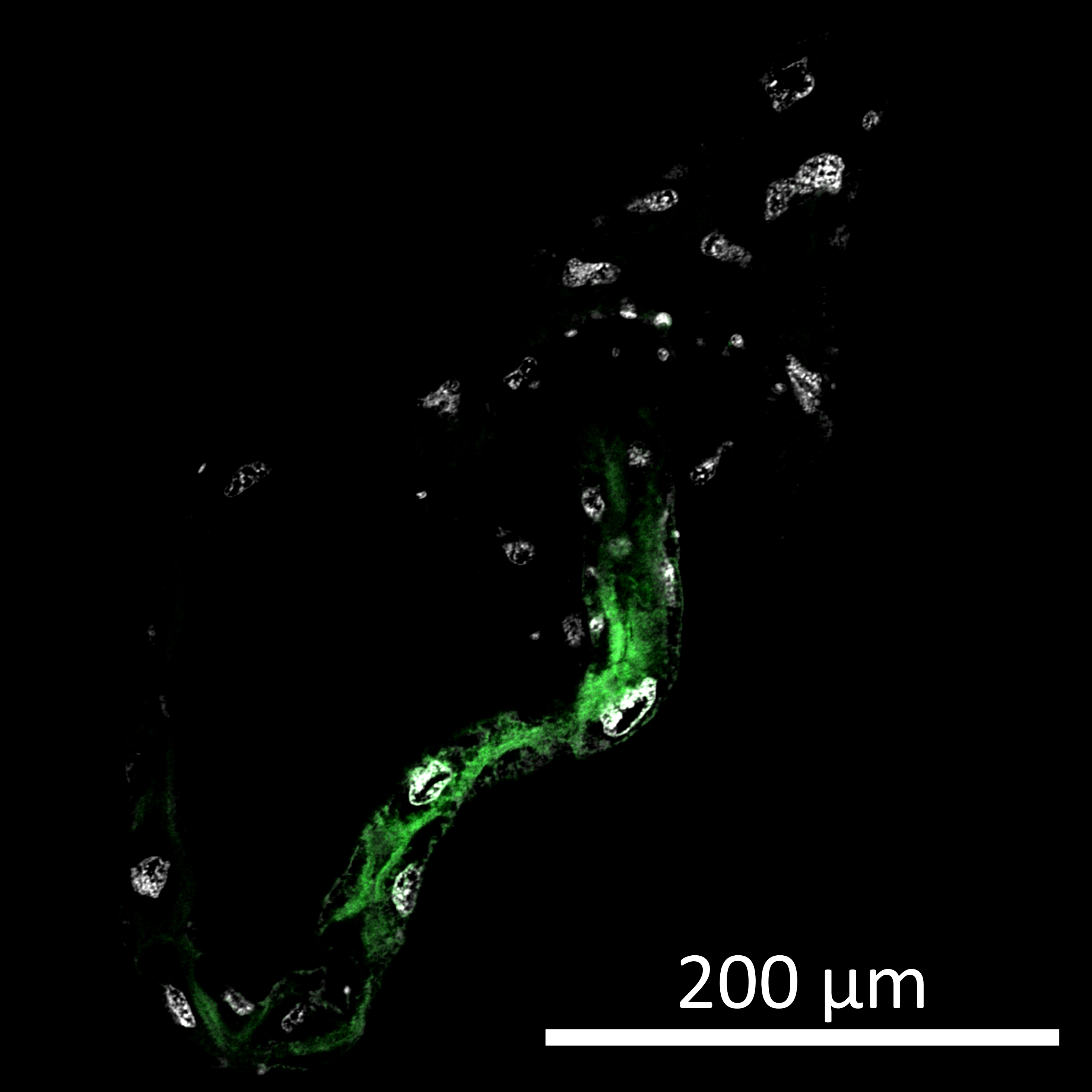

Supplement: Supplementary file 4 — Source Data [file 41467_2022_28500_MOESM4_ESM.zip › Source data/Fig1 A,B/MG-TYLCV_c1+2.jpg]

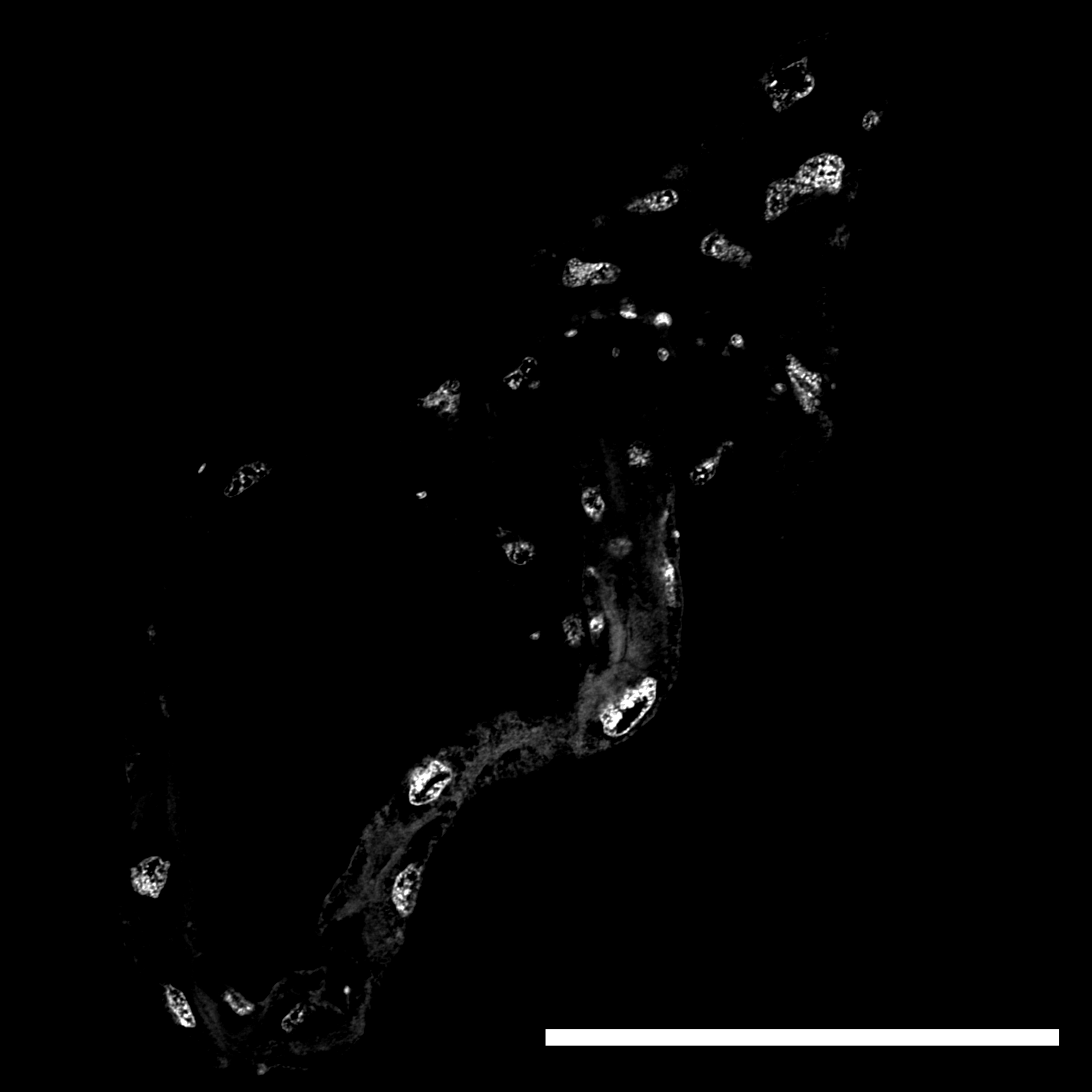

Supplement: Supplementary file 4 — Source Data [file 41467_2022_28500_MOESM4_ESM.zip › Source data/Fig1 A,B/MG-TYLCV_c1.jpg]

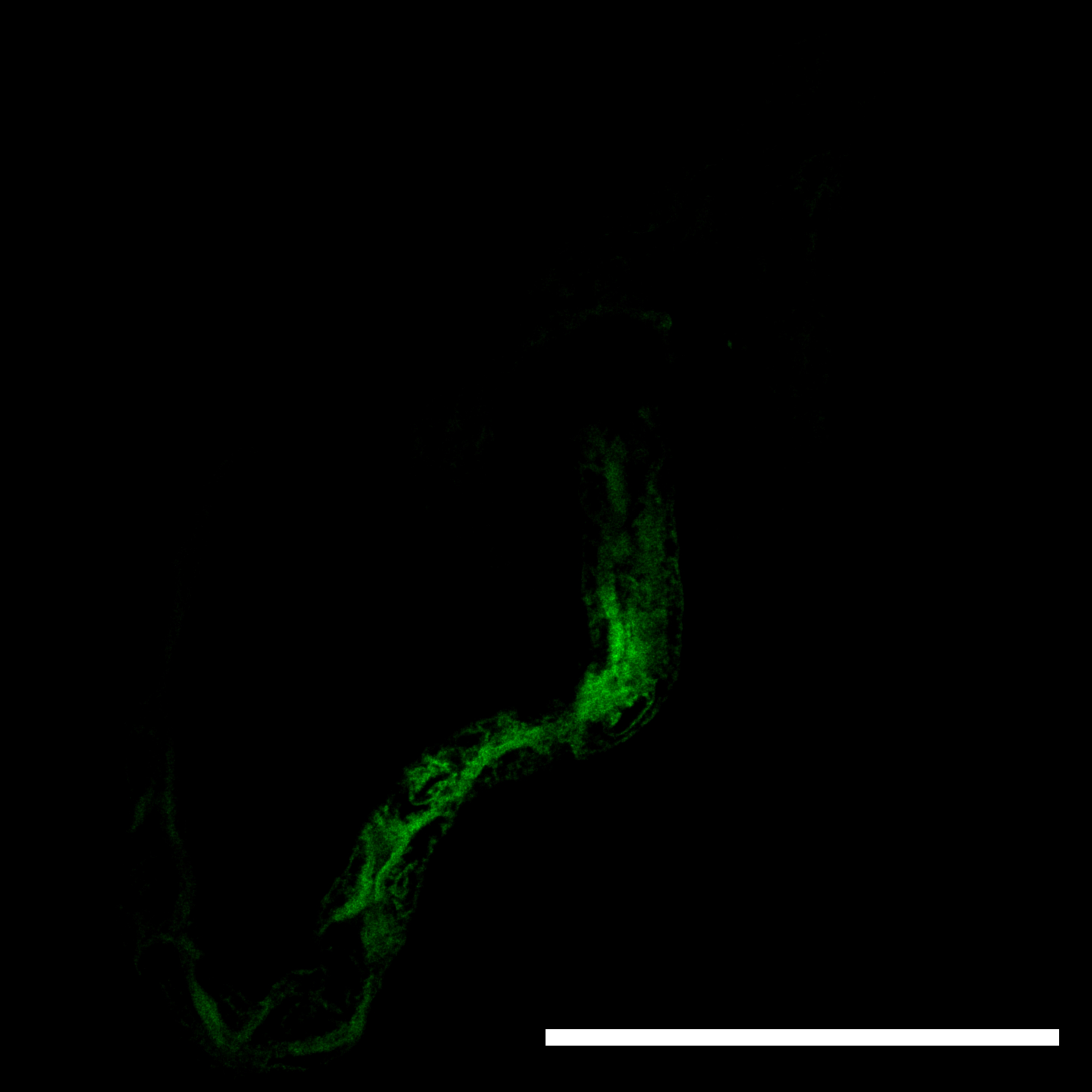

Supplement: Supplementary file 4 — Source Data [file 41467_2022_28500_MOESM4_ESM.zip › Source data/Fig1 A,B/MG-TYLCV_c2.jpg]

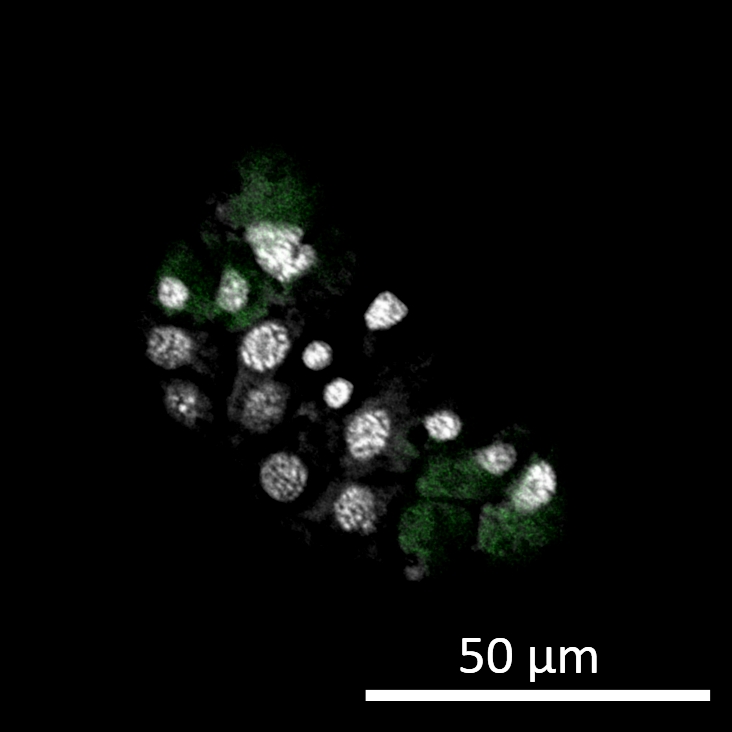

Supplement: Supplementary file 4 — Source Data [file 41467_2022_28500_MOESM4_ESM.zip › Source data/Fig1 A,B/SG-CTL_c1+2.jpg]

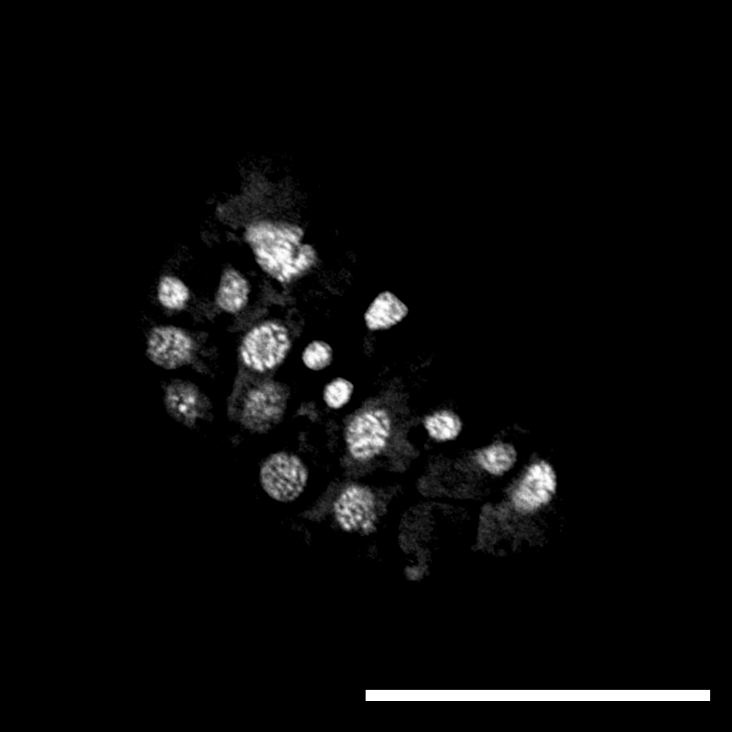

Supplement: Supplementary file 4 — Source Data [file 41467_2022_28500_MOESM4_ESM.zip › Source data/Fig1 A,B/SG-CTL_c1.jpg]

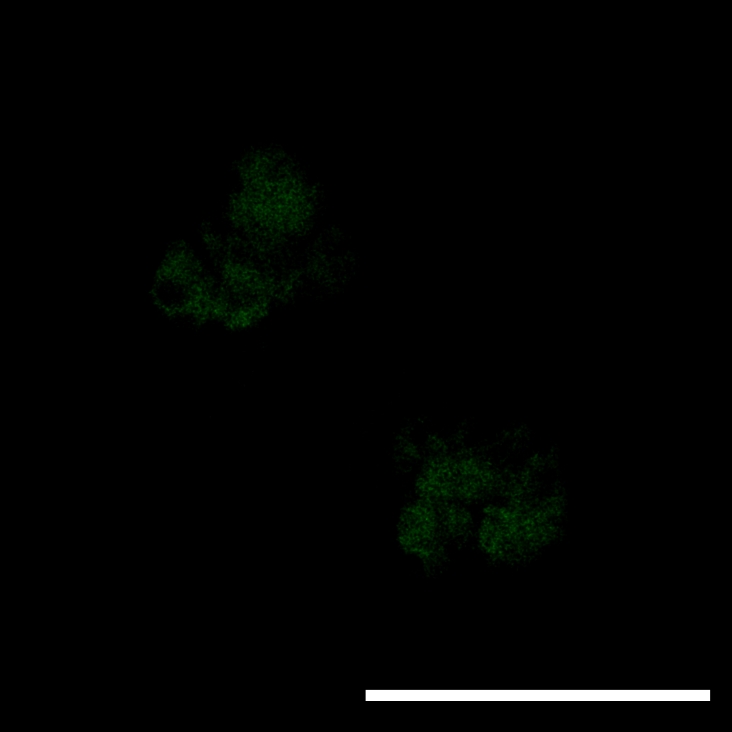

Supplement: Supplementary file 4 — Source Data [file 41467_2022_28500_MOESM4_ESM.zip › Source data/Fig1 A,B/SG-CTL_c2.jpg]

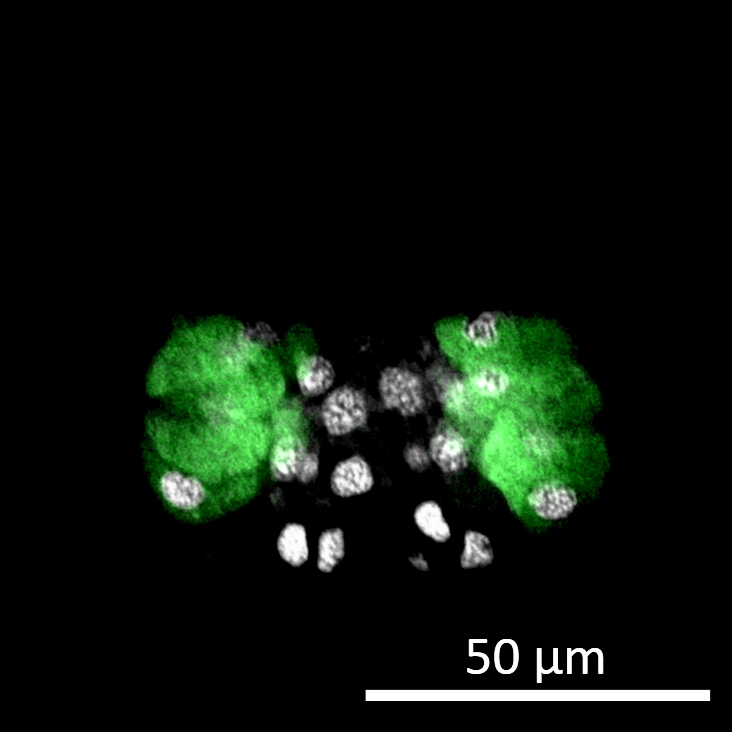

Supplement: Supplementary file 4 — Source Data [file 41467_2022_28500_MOESM4_ESM.zip › Source data/Fig1 A,B/SG-TYLCV_c1+2.jpg]

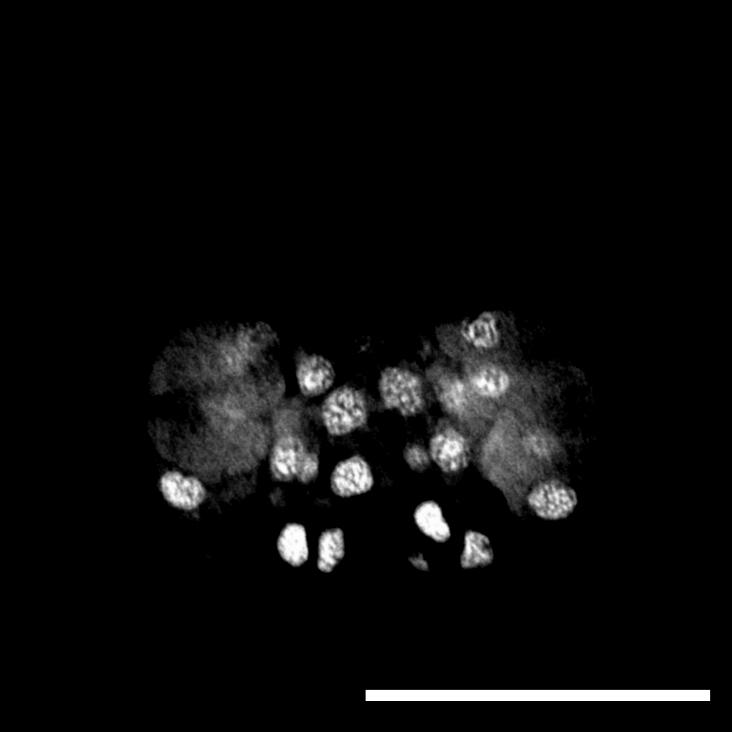

Supplement: Supplementary file 4 — Source Data [file 41467_2022_28500_MOESM4_ESM.zip › Source data/Fig1 A,B/SG-TYLCV_c1.jpg]

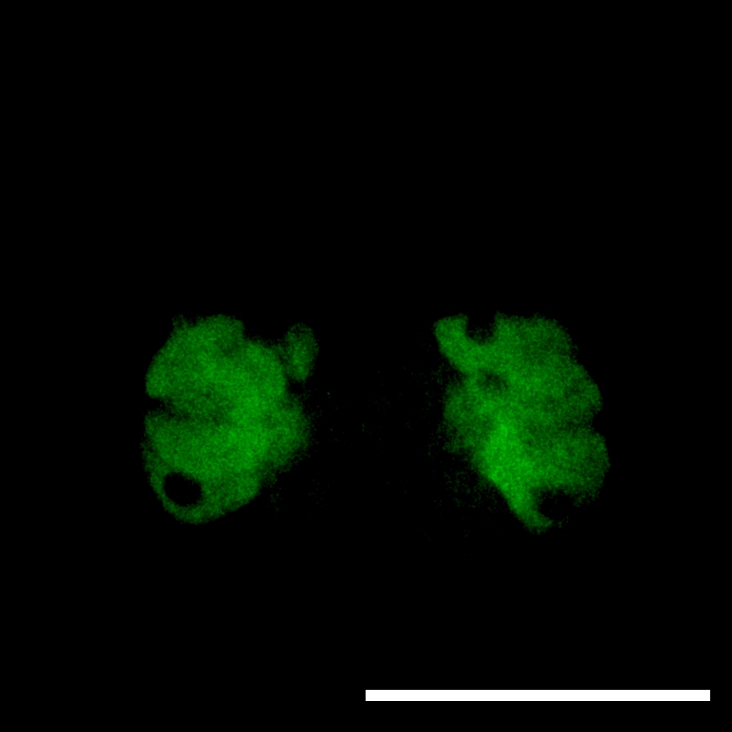

Supplement: Supplementary file 4 — Source Data [file 41467_2022_28500_MOESM4_ESM.zip › Source data/Fig1 A,B/SG-TYLCV_c2.jpg]

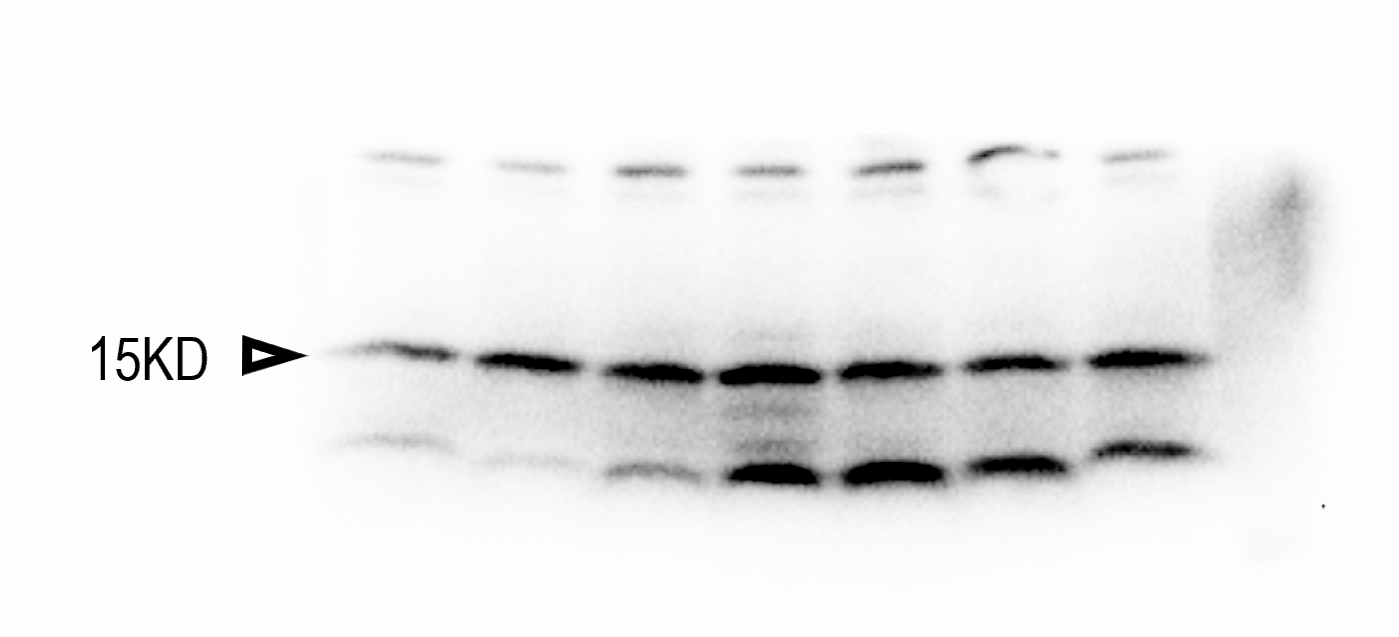

Supplement: Supplementary file 4 — Source Data [file 41467_2022_28500_MOESM4_ESM.zip › Source data/Fig1 E/ATG8.tif]

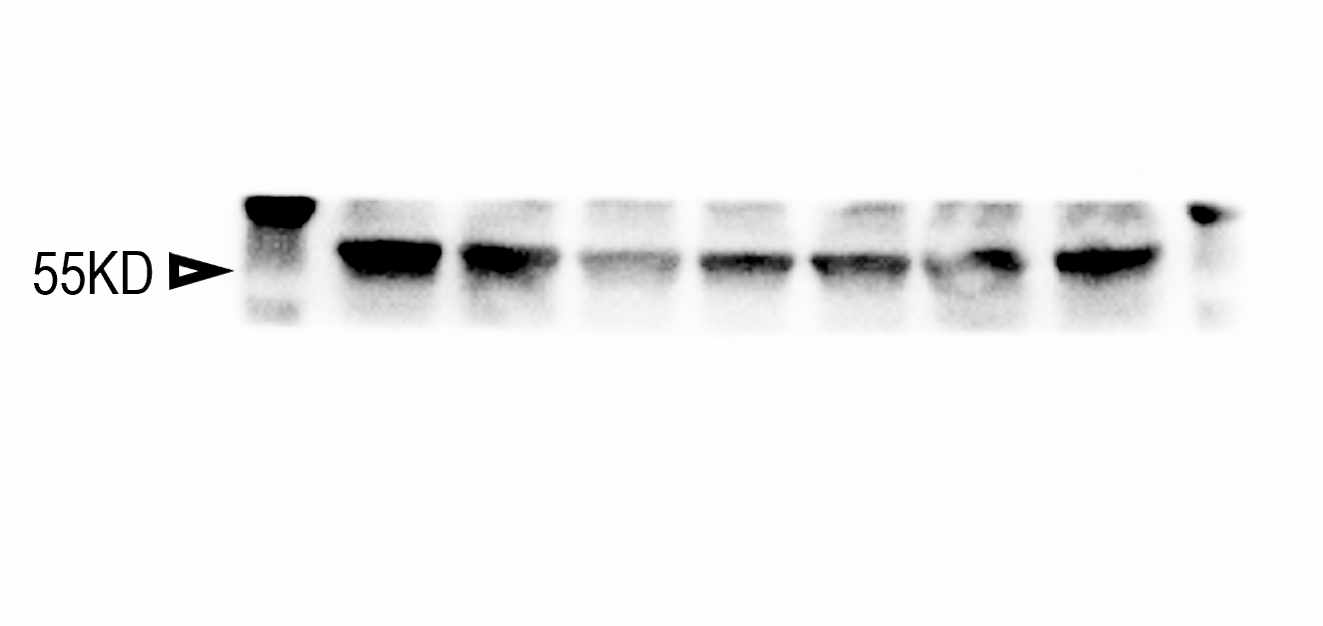

Supplement: Supplementary file 4 — Source Data [file 41467_2022_28500_MOESM4_ESM.zip › Source data/Fig1 E/Casp3.tif]

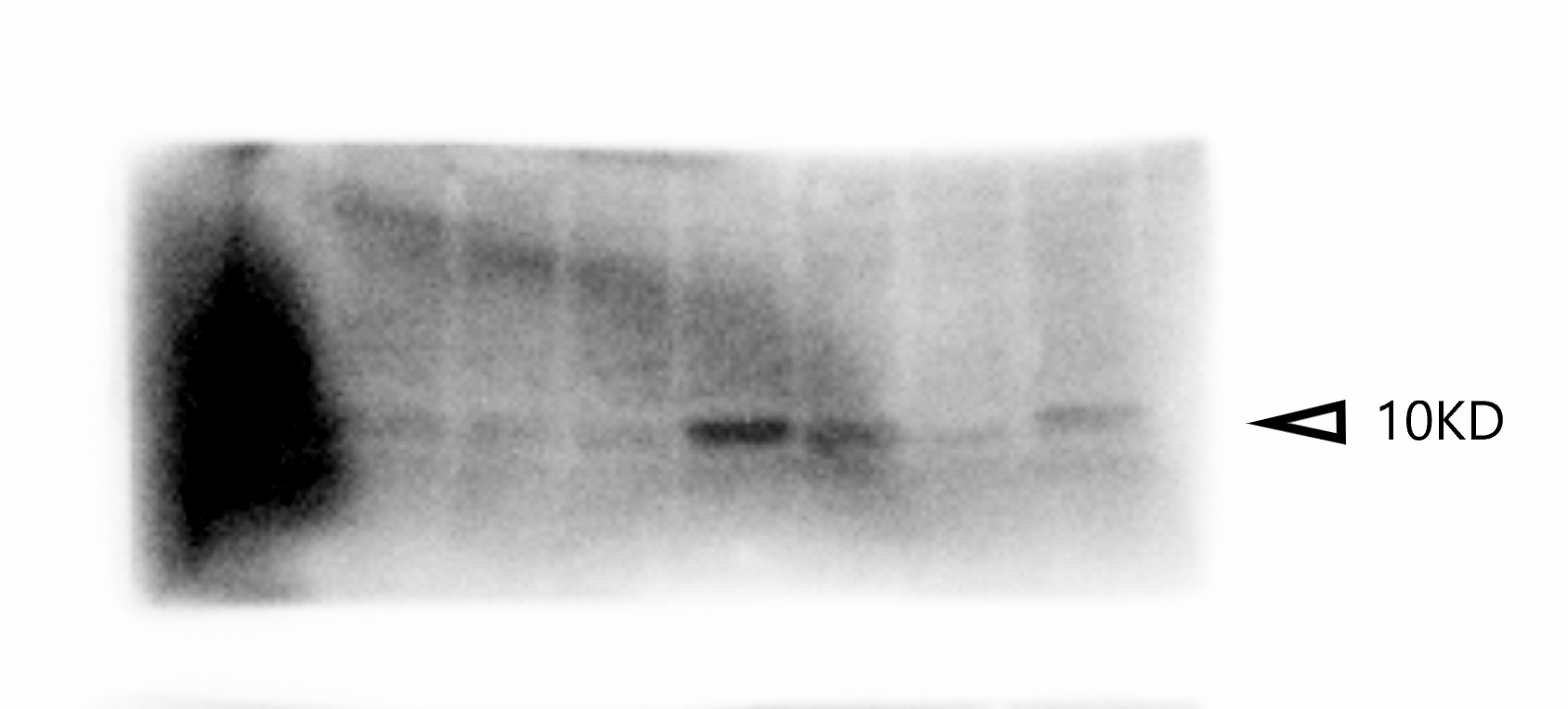

Supplement: Supplementary file 4 — Source Data [file 41467_2022_28500_MOESM4_ESM.zip › Source data/Fig1 E/cCasp3.tif]

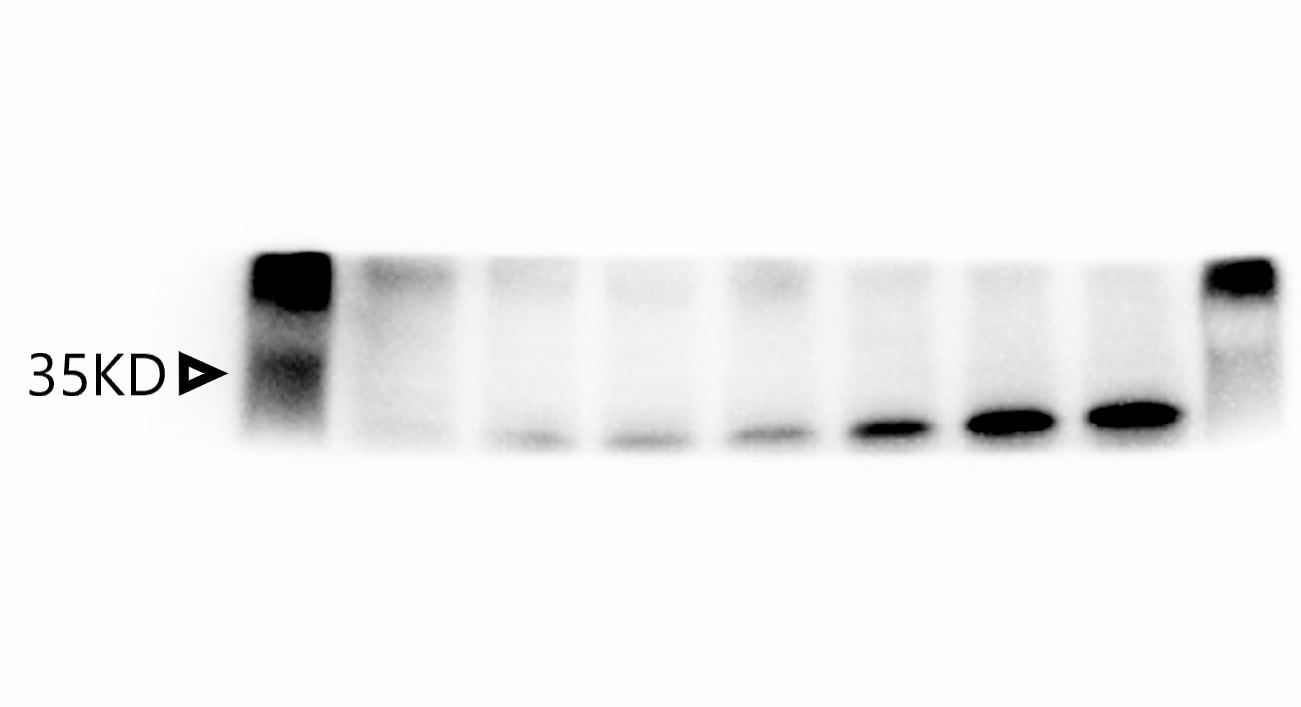

Supplement: Supplementary file 4 — Source Data [file 41467_2022_28500_MOESM4_ESM.zip › Source data/Fig1 E/CP.tif]

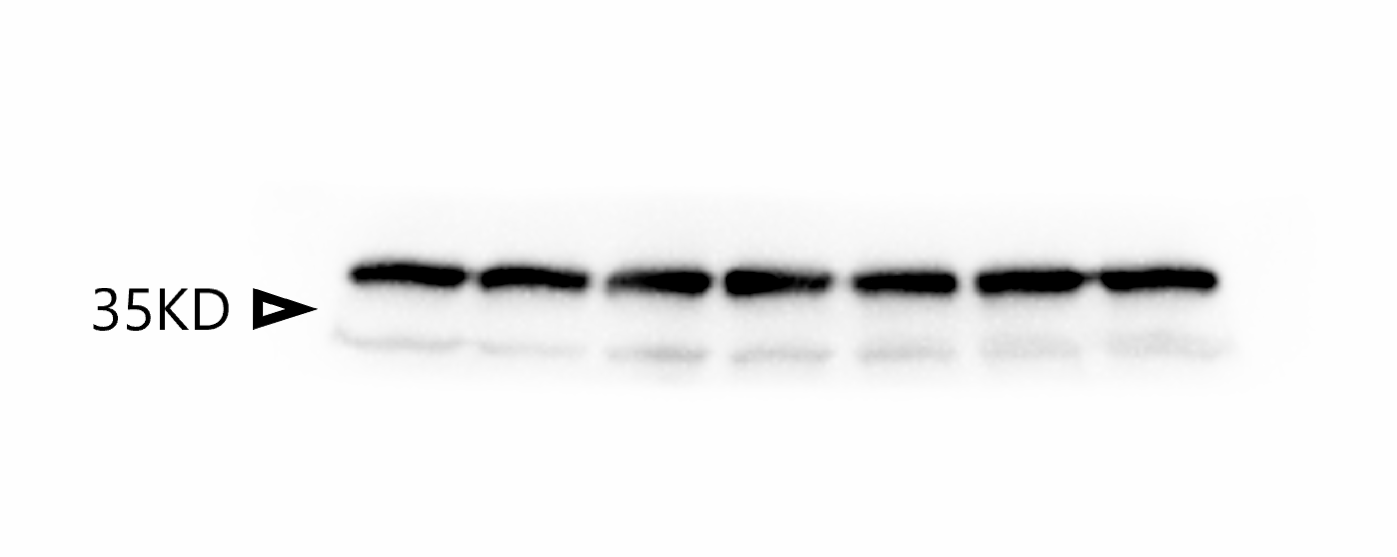

Supplement: Supplementary file 4 — Source Data [file 41467_2022_28500_MOESM4_ESM.zip › Source data/Fig1 E/GAPDH.tif]

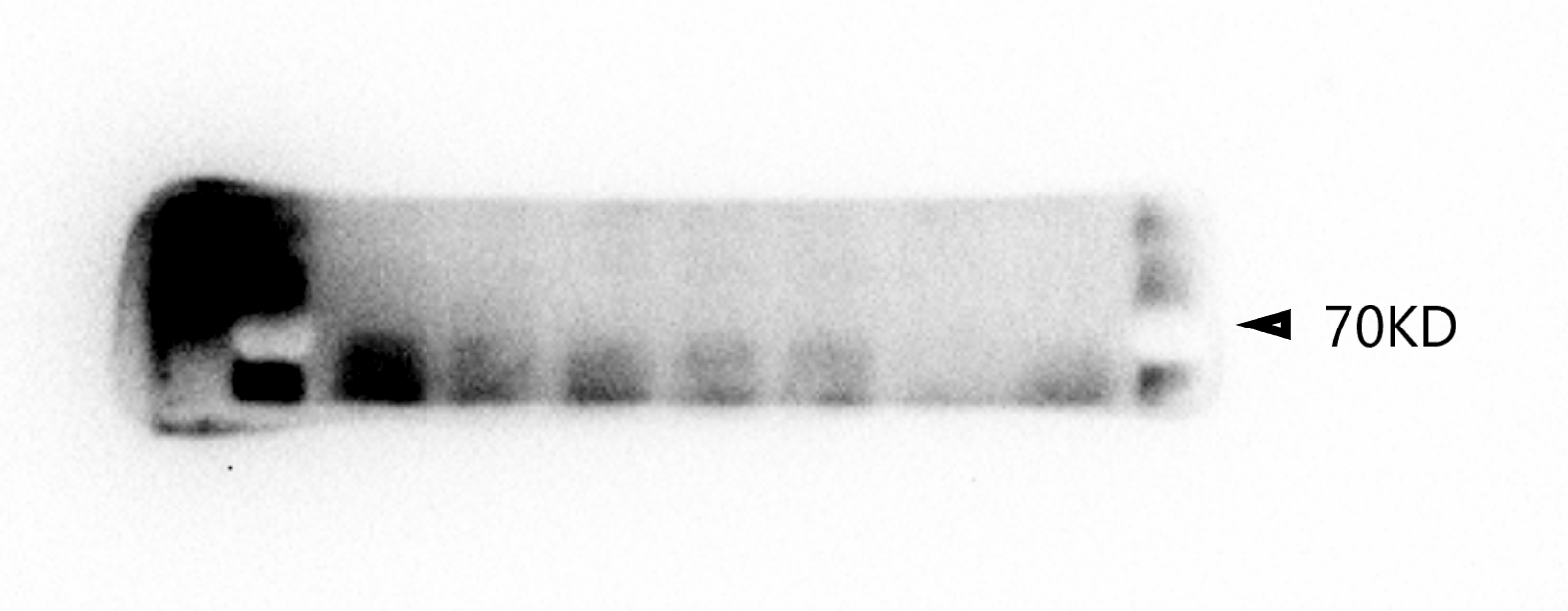

Supplement: Supplementary file 4 — Source Data [file 41467_2022_28500_MOESM4_ESM.zip › Source data/Fig1 E/SQSTM1.tif]

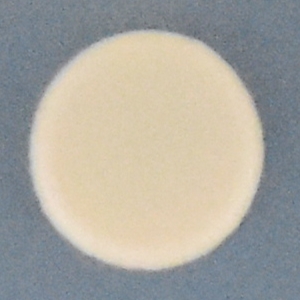

Supplement: Supplementary file 4 — Source Data [file 41467_2022_28500_MOESM4_ESM.zip › Source data/Fig2 A/DDONeg.jpg]

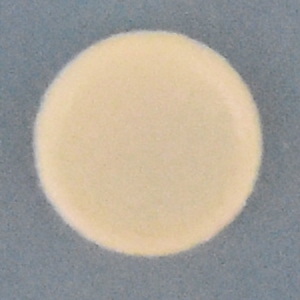

Supplement: Supplementary file 4 — Source Data [file 41467_2022_28500_MOESM4_ESM.zip › Source data/Fig2 A/DDOPEBP4.jpg]

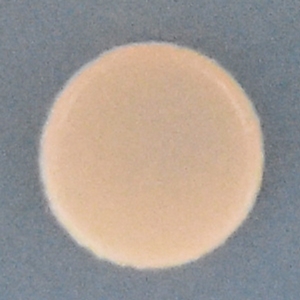

Supplement: Supplementary file 4 — Source Data [file 41467_2022_28500_MOESM4_ESM.zip › Source data/Fig2 A/DDOPos.jpg]

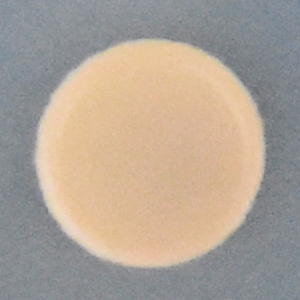

Supplement: Supplementary file 4 — Source Data [file 41467_2022_28500_MOESM4_ESM.zip › Source data/Fig2 A/DDOT7.jpg]

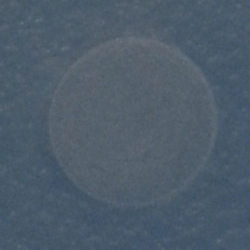

Supplement: Supplementary file 4 — Source Data [file 41467_2022_28500_MOESM4_ESM.zip › Source data/Fig2 A/QDONeg.jpg]

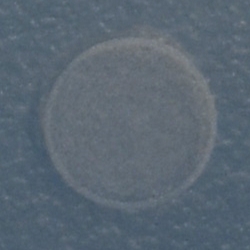

Supplement: Supplementary file 4 — Source Data [file 41467_2022_28500_MOESM4_ESM.zip › Source data/Fig2 A/QDOPEBP4.jpg]

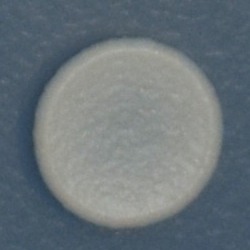

Supplement: Supplementary file 4 — Source Data [file 41467_2022_28500_MOESM4_ESM.zip › Source data/Fig2 A/QDOPos.jpg]

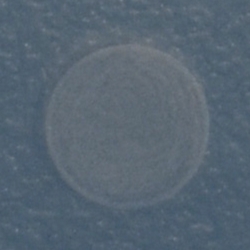

Supplement: Supplementary file 4 — Source Data [file 41467_2022_28500_MOESM4_ESM.zip › Source data/Fig2 A/QDOT7.jpg]

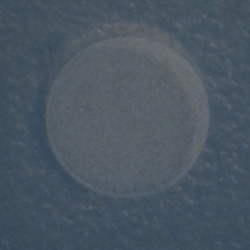

Supplement: Supplementary file 4 — Source Data [file 41467_2022_28500_MOESM4_ESM.zip › Source data/Fig2 A/TDONeg.jpg]

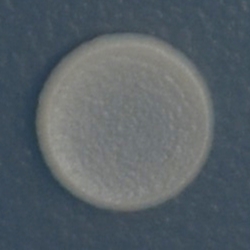

Supplement: Supplementary file 4 — Source Data [file 41467_2022_28500_MOESM4_ESM.zip › Source data/Fig2 A/TDOPEBP4.jpg]

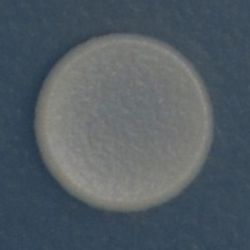

Supplement: Supplementary file 4 — Source Data [file 41467_2022_28500_MOESM4_ESM.zip › Source data/Fig2 A/TDOPos.jpg]

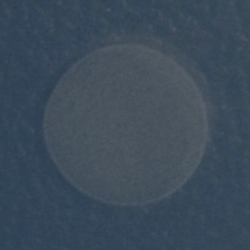

Supplement: Supplementary file 4 — Source Data [file 41467_2022_28500_MOESM4_ESM.zip › Source data/Fig2 A/TDOT7.jpg]

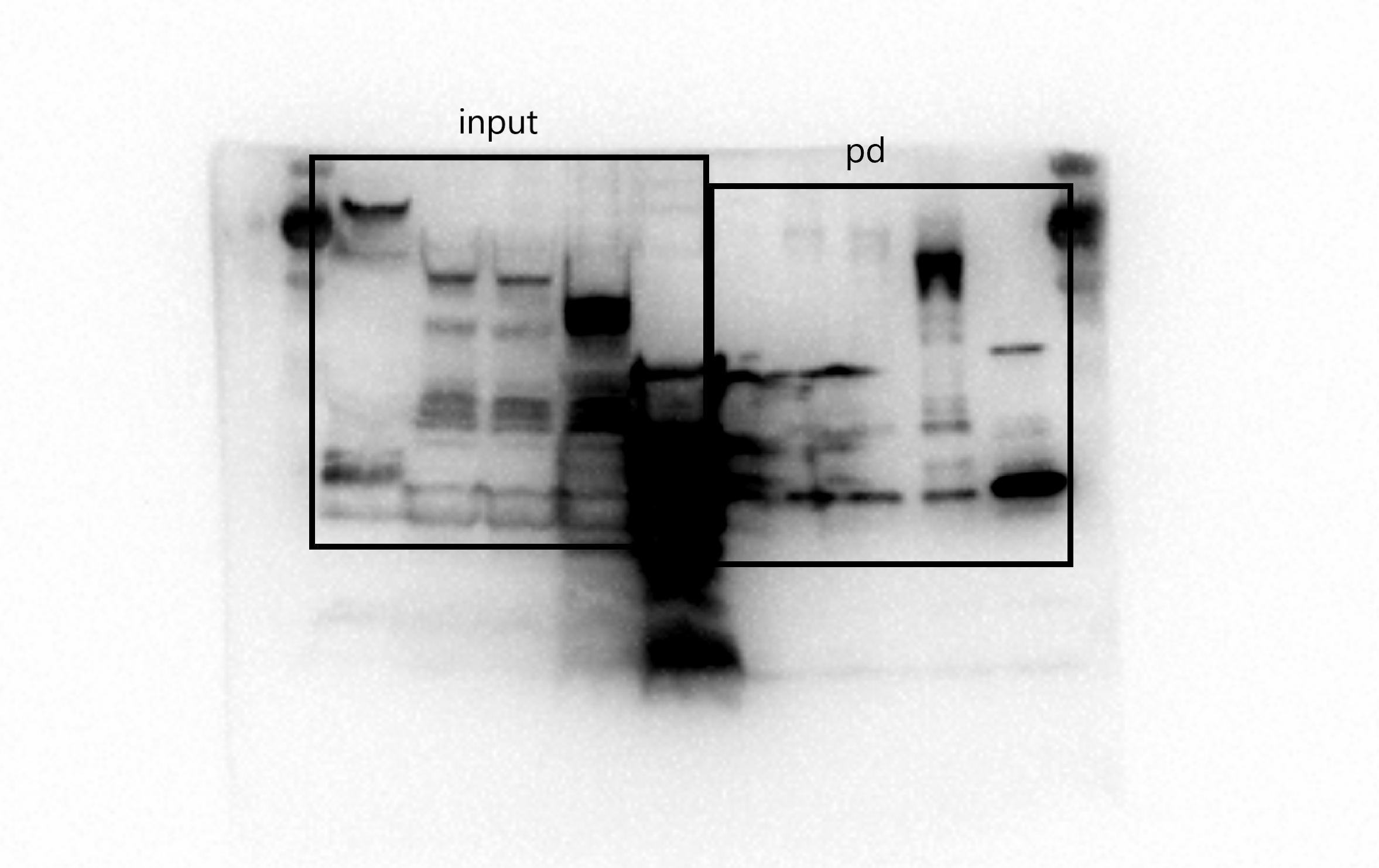

Supplement: Supplementary file 4 — Source Data [file 41467_2022_28500_MOESM4_ESM.zip › Source data/Fig2 B,C/anti-GST.tif]

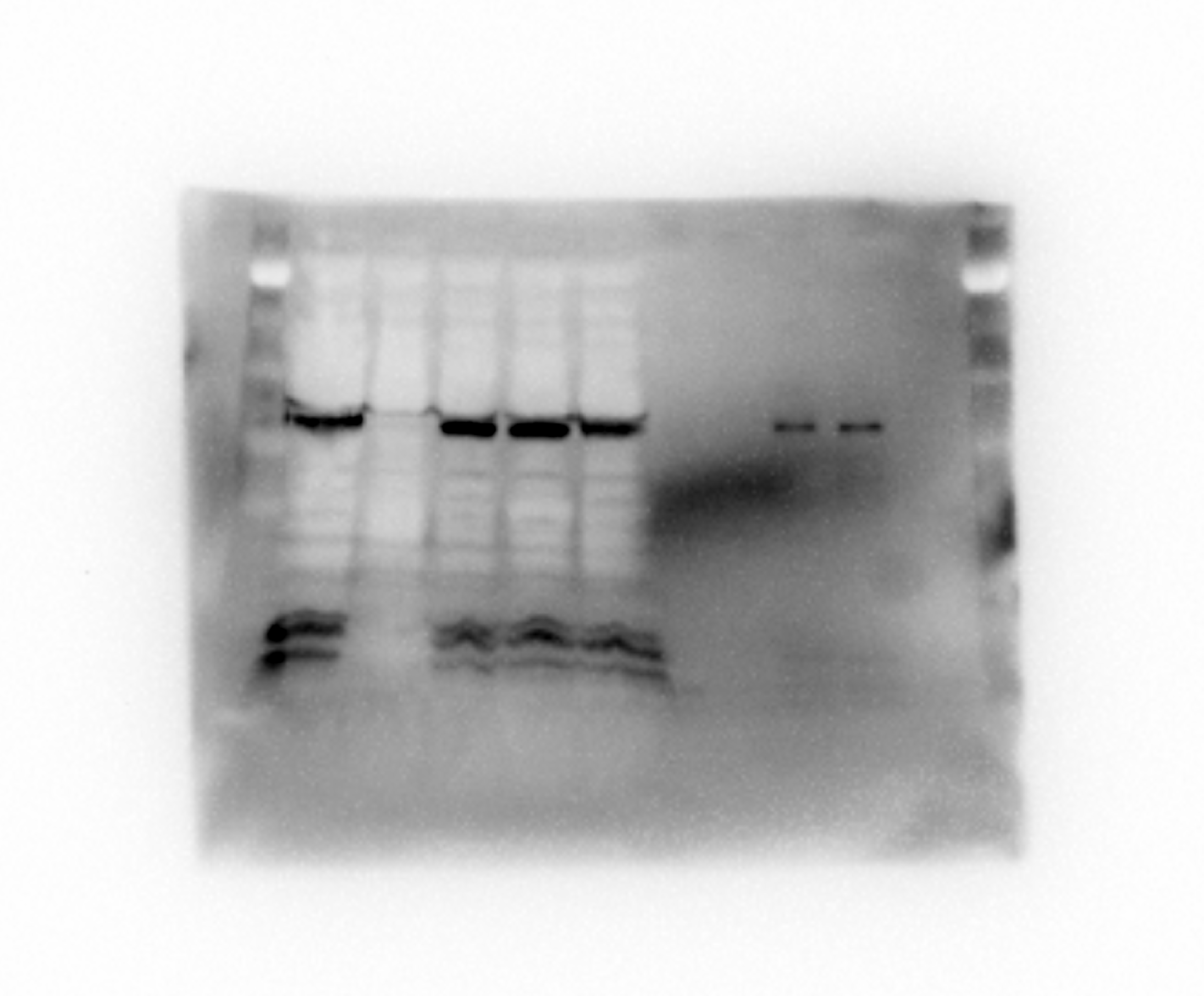

Supplement: Supplementary file 4 — Source Data [file 41467_2022_28500_MOESM4_ESM.zip › Source data/Fig2 B,C/anti-His.tif]

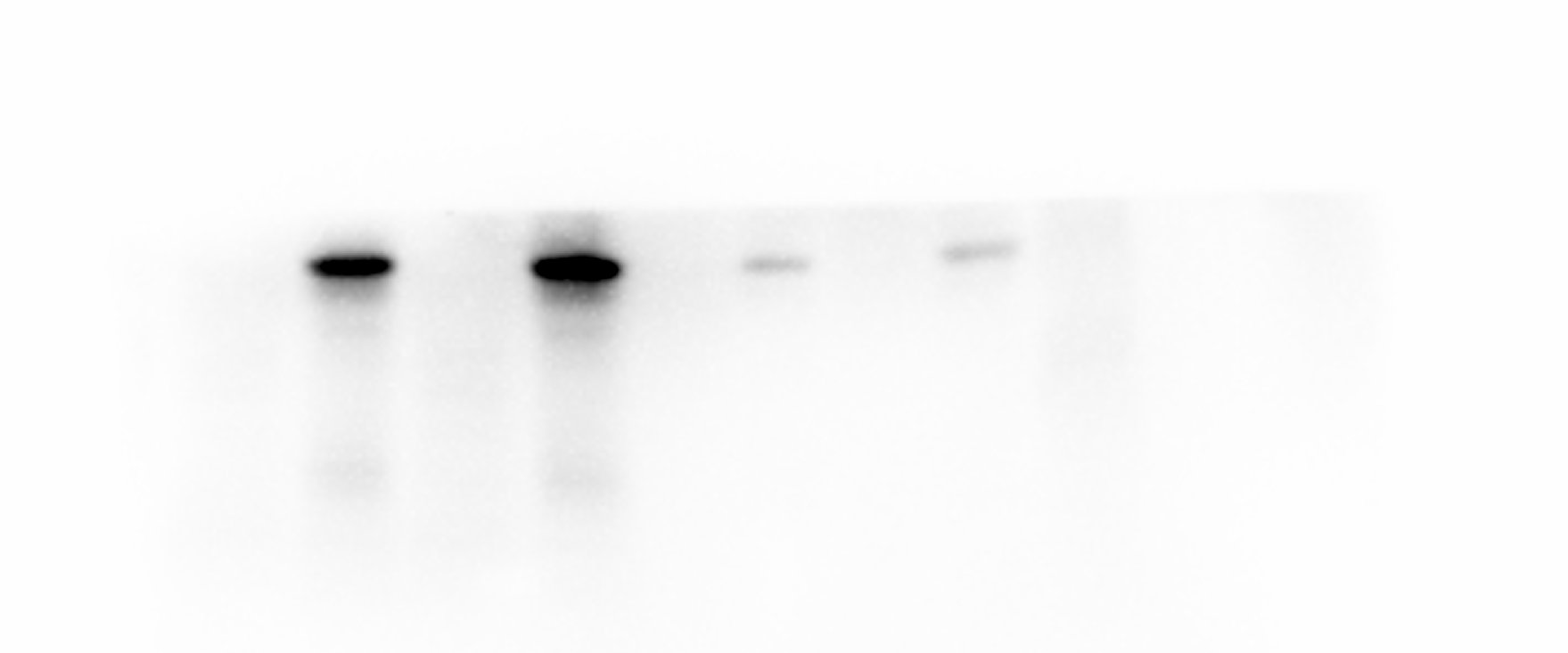

Supplement: Supplementary file 4 — Source Data [file 41467_2022_28500_MOESM4_ESM.zip › Source data/Fig2 B,C/CP.tif]

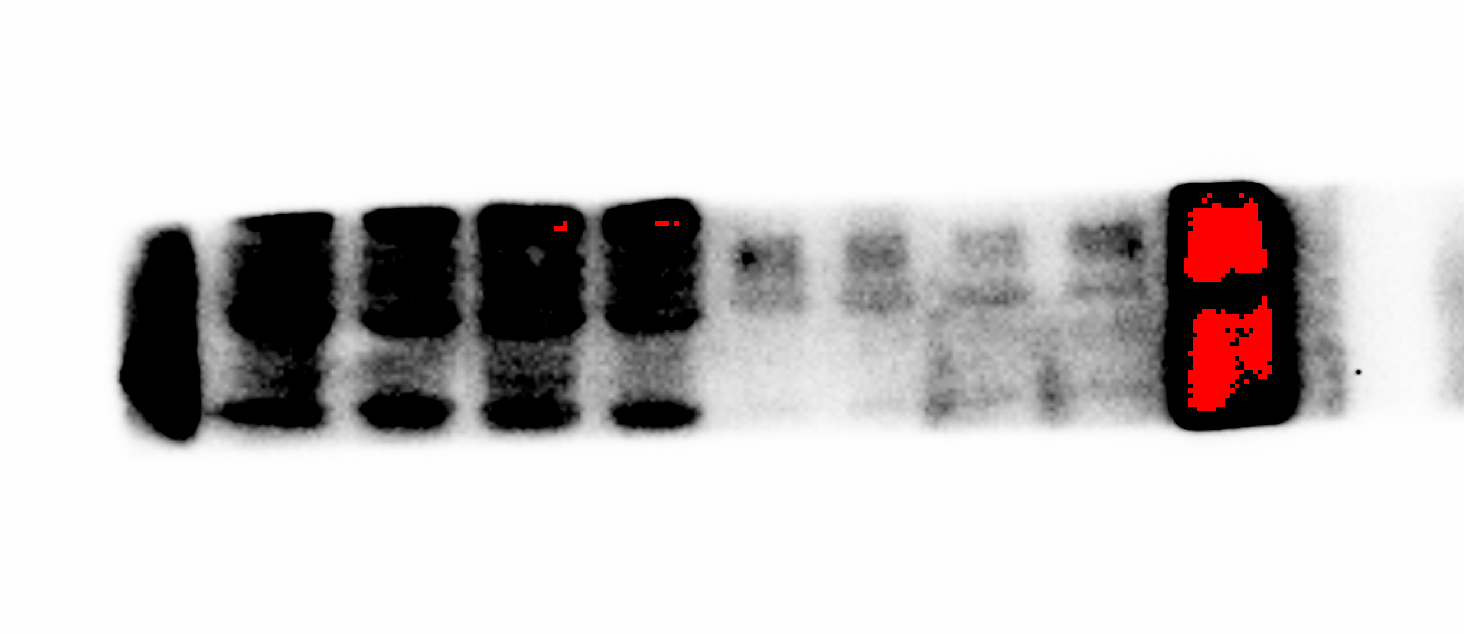

Supplement: Supplementary file 4 — Source Data [file 41467_2022_28500_MOESM4_ESM.zip › Source data/Fig2 B,C/PEBP4 (long exposure).tif]

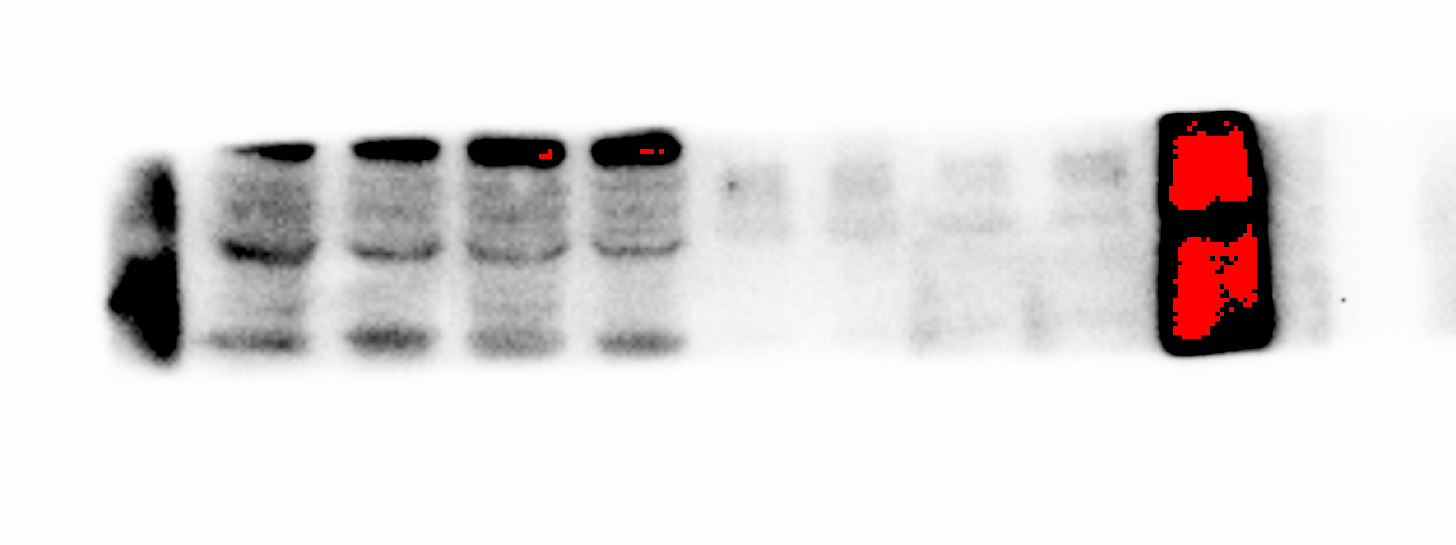

Supplement: Supplementary file 4 — Source Data [file 41467_2022_28500_MOESM4_ESM.zip › Source data/Fig2 B,C/PEBP4 (short exposure).tif]

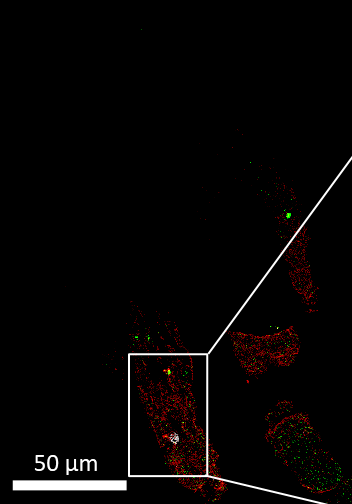

Supplement: Supplementary file 4 — Source Data [file 41467_2022_28500_MOESM4_ESM.zip › Source data/Fig2 D/CP-PEBP4-MG (2).tif]

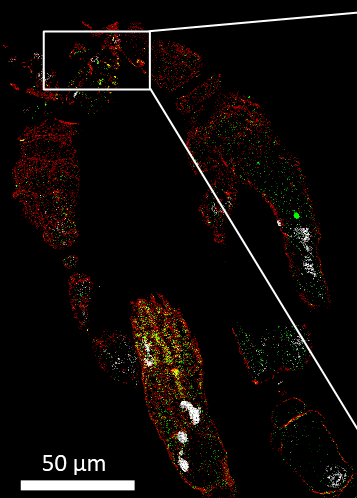

Supplement: Supplementary file 4 — Source Data [file 41467_2022_28500_MOESM4_ESM.zip › Source data/Fig2 D/CP-PEBP4-MG (3).tif]

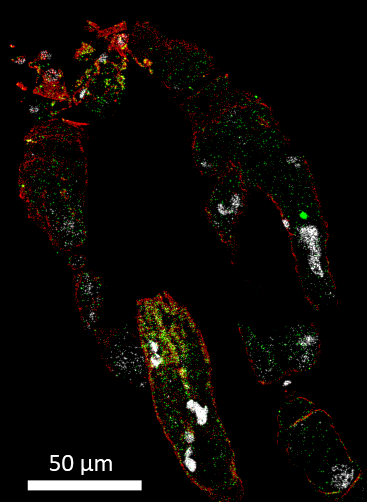

Supplement: Supplementary file 4 — Source Data [file 41467_2022_28500_MOESM4_ESM.zip › Source data/Fig2 D/CP-PEBP4-MG (4).tif]

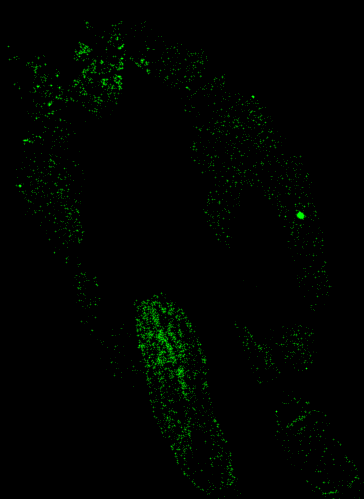

Supplement: Supplementary file 4 — Source Data [file 41467_2022_28500_MOESM4_ESM.zip › Source data/Fig2 D/CP-PEBP4-MG (5).tif]

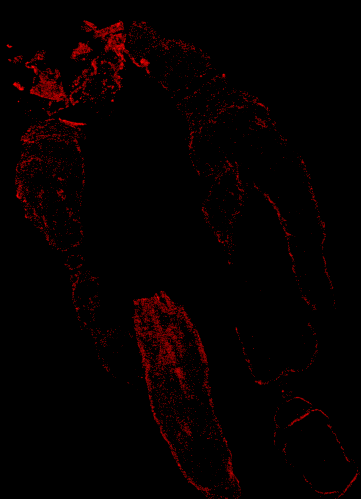

Supplement: Supplementary file 4 — Source Data [file 41467_2022_28500_MOESM4_ESM.zip › Source data/Fig2 D/CP-PEBP4-MG (6).tif]

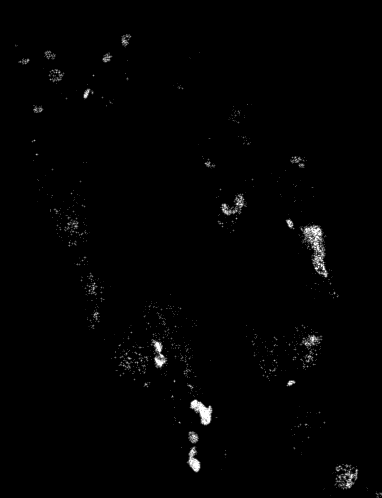

Supplement: Supplementary file 4 — Source Data [file 41467_2022_28500_MOESM4_ESM.zip › Source data/Fig2 D/CP-PEBP4-MG (7).tif]

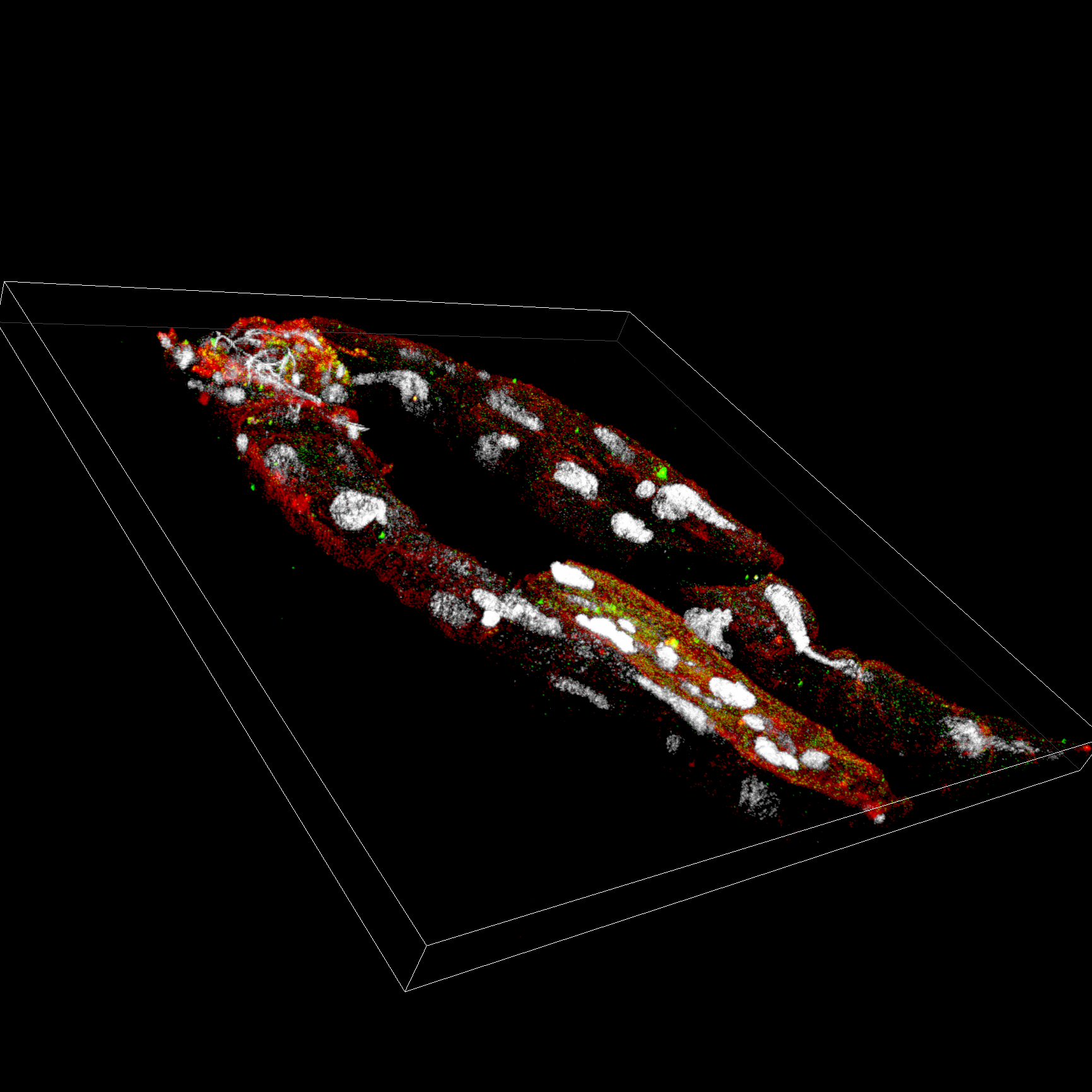

Supplement: Supplementary file 4 — Source Data [file 41467_2022_28500_MOESM4_ESM.zip › Source data/Fig2 D/CP-PEBP4-MG.png]

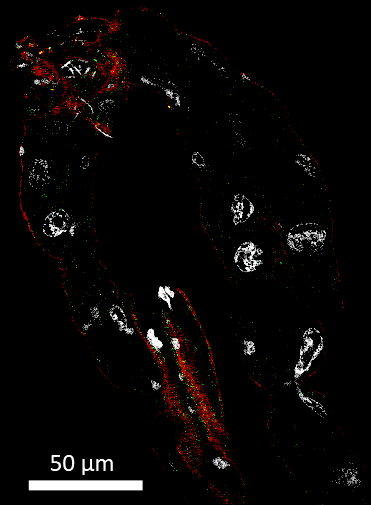

Supplement: Supplementary file 4 — Source Data [file 41467_2022_28500_MOESM4_ESM.zip › Source data/Fig2 D/CP-PEBP4-MG.tif]

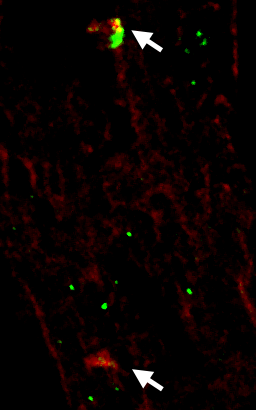

Supplement: Supplementary file 4 — Source Data [file 41467_2022_28500_MOESM4_ESM.zip › Source data/Fig2 D/CP-PEBP4-MG_c1+2 (2).tif]

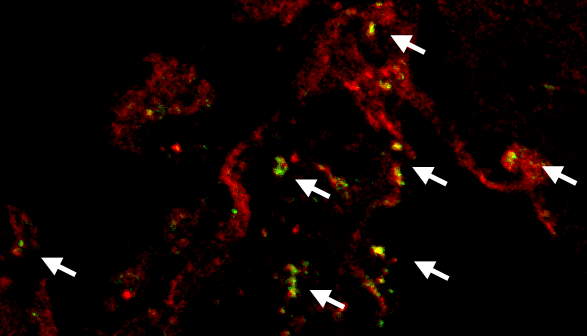

Supplement: Supplementary file 4 — Source Data [file 41467_2022_28500_MOESM4_ESM.zip › Source data/Fig2 D/CP-PEBP4-MG_c1+2+3+4 (2).tif]

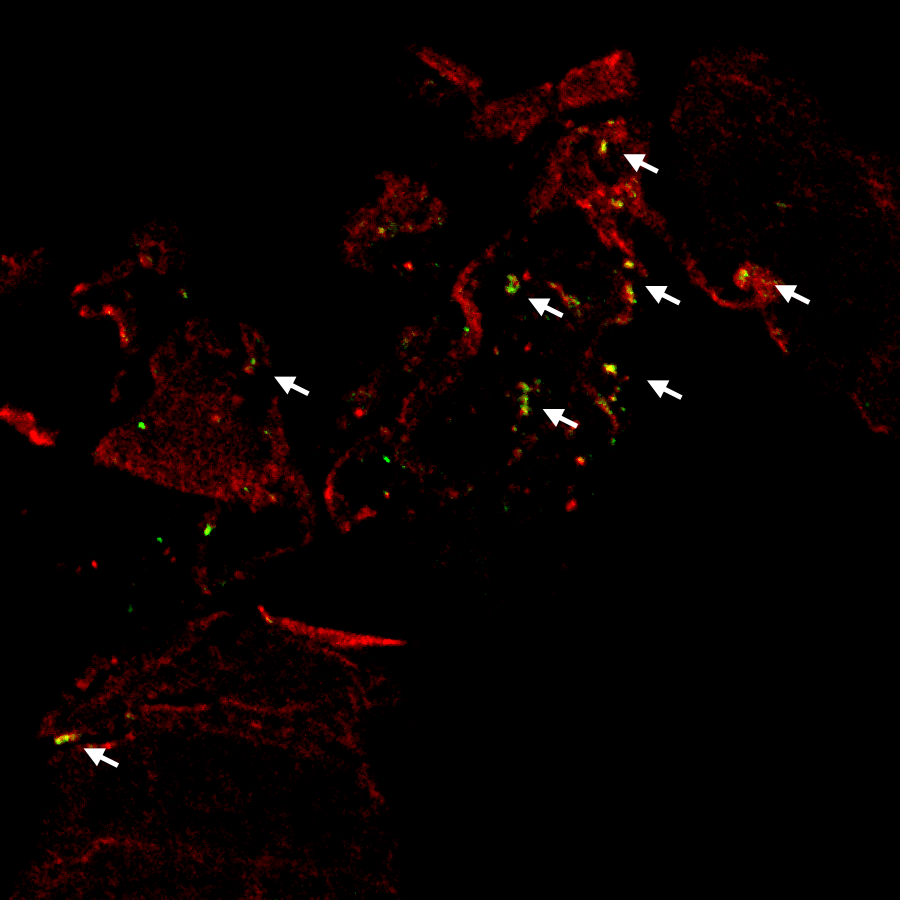

Supplement: Supplementary file 4 — Source Data [file 41467_2022_28500_MOESM4_ESM.zip › Source data/Fig2 D/CP-PEBP4-MG_c1+2+3+4.tif]

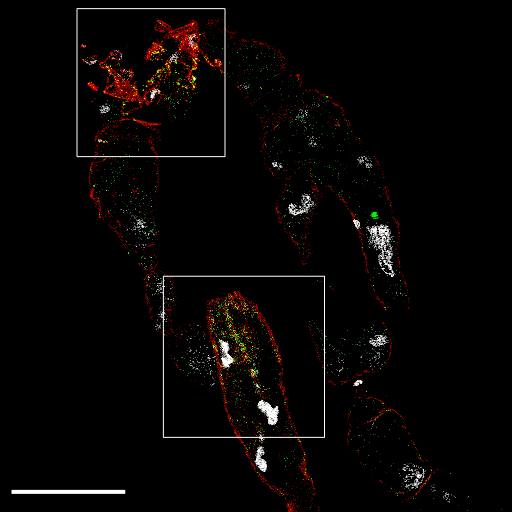

Supplement: Supplementary file 4 — Source Data [file 41467_2022_28500_MOESM4_ESM.zip › Source data/Fig2 D/CP-PEBP4-MG_c1+2+3.tif]

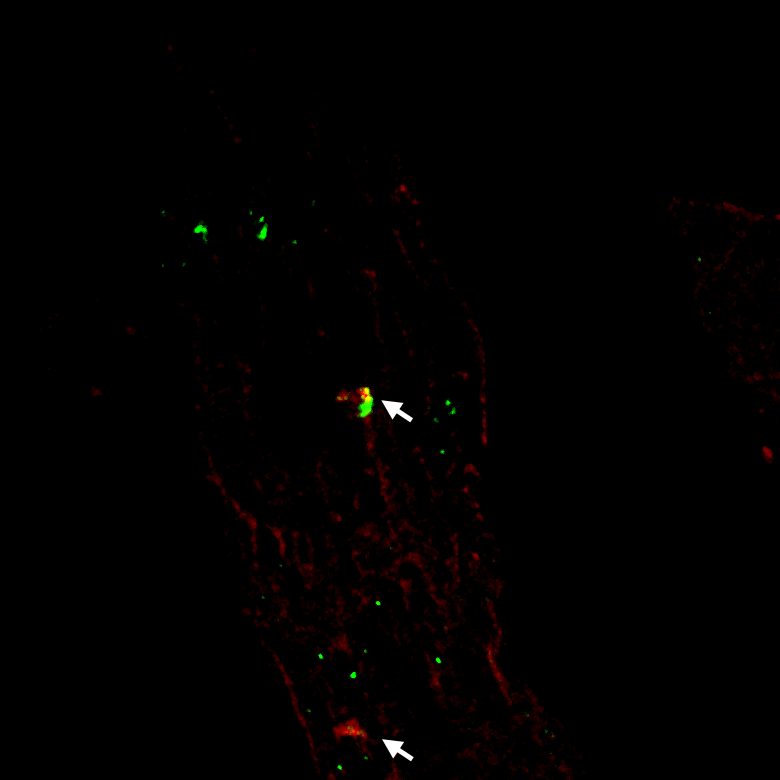

Supplement: Supplementary file 4 — Source Data [file 41467_2022_28500_MOESM4_ESM.zip › Source data/Fig2 D/CP-PEBP4-MG_c1+2.tif]

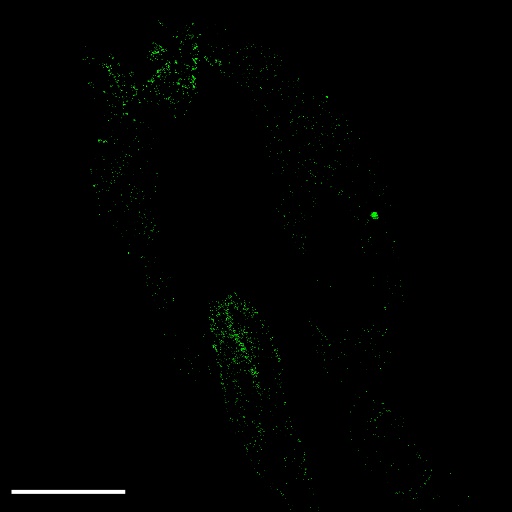

Supplement: Supplementary file 4 — Source Data [file 41467_2022_28500_MOESM4_ESM.zip › Source data/Fig2 D/CP-PEBP4-MG_c1.jpg]

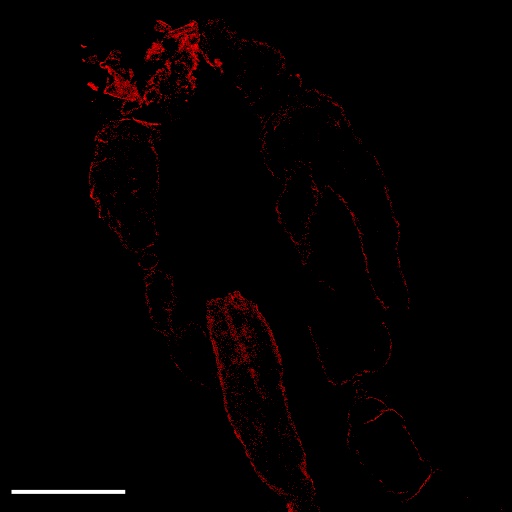

Supplement: Supplementary file 4 — Source Data [file 41467_2022_28500_MOESM4_ESM.zip › Source data/Fig2 D/CP-PEBP4-MG_c2.jpg]

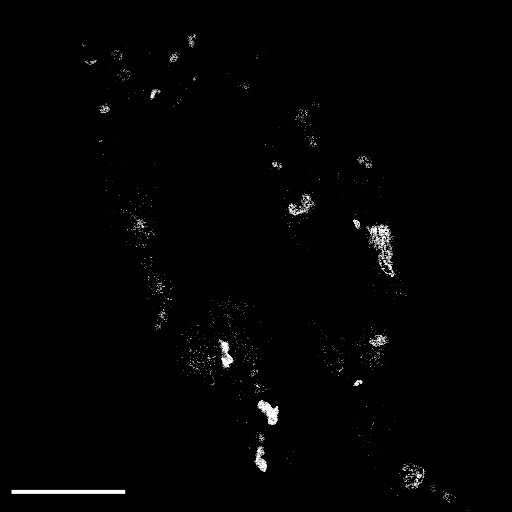

Supplement: Supplementary file 4 — Source Data [file 41467_2022_28500_MOESM4_ESM.zip › Source data/Fig2 D/CP-PEBP4-MG_c3.jpg]

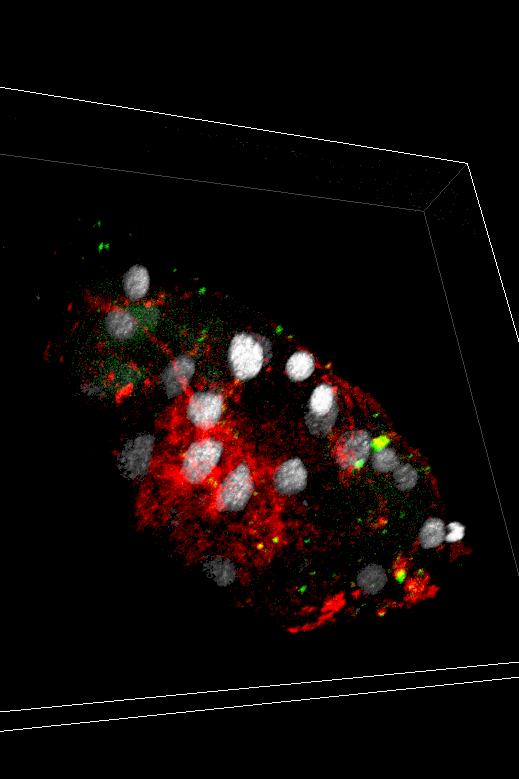

Supplement: Supplementary file 4 — Source Data [file 41467_2022_28500_MOESM4_ESM.zip › Source data/Fig2 D/V-SG-2-3 (Snapshot).tif]

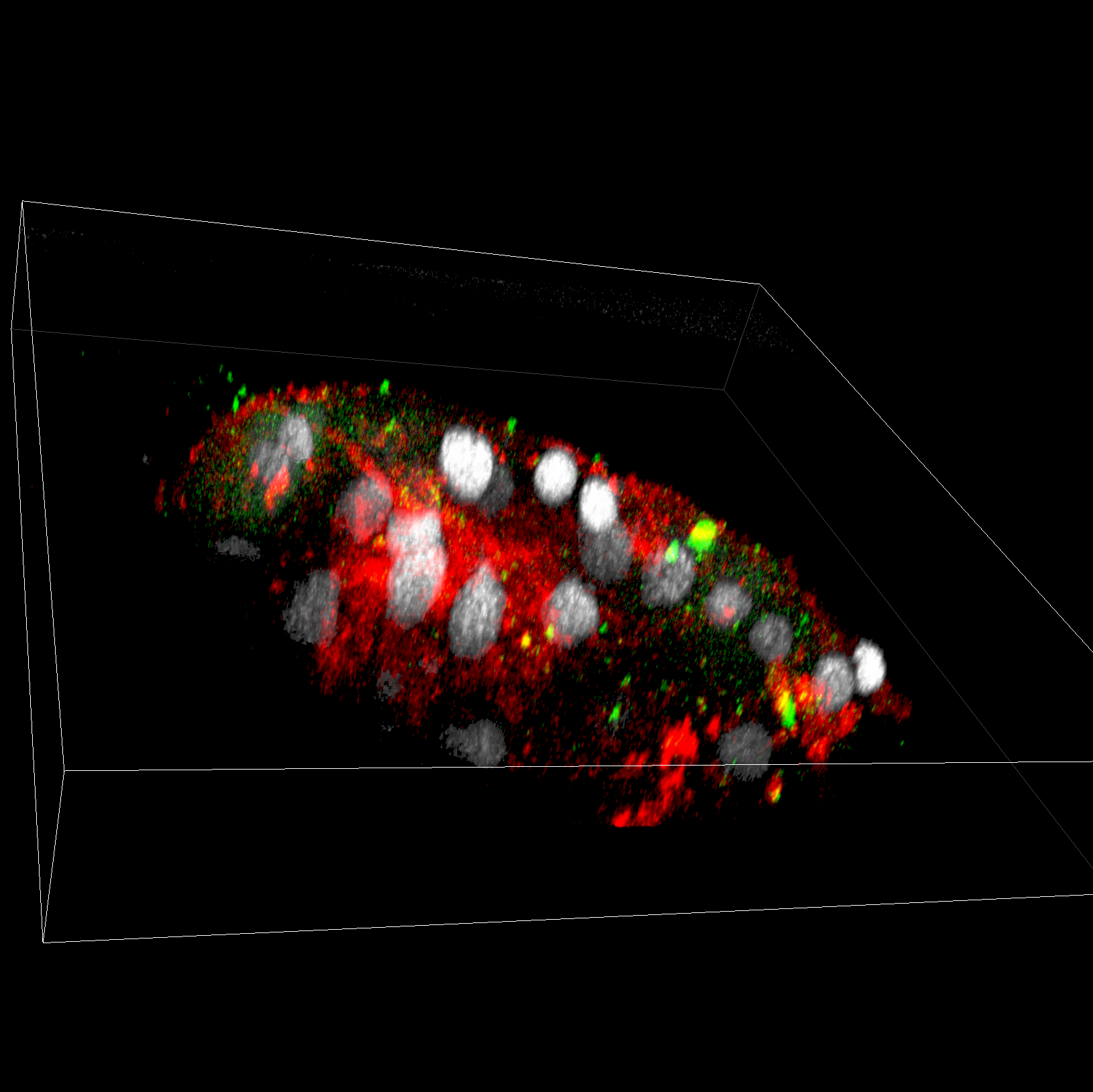

Supplement: Supplementary file 4 — Source Data [file 41467_2022_28500_MOESM4_ESM.zip › Source data/Fig2 D/V-SG-3D.png]

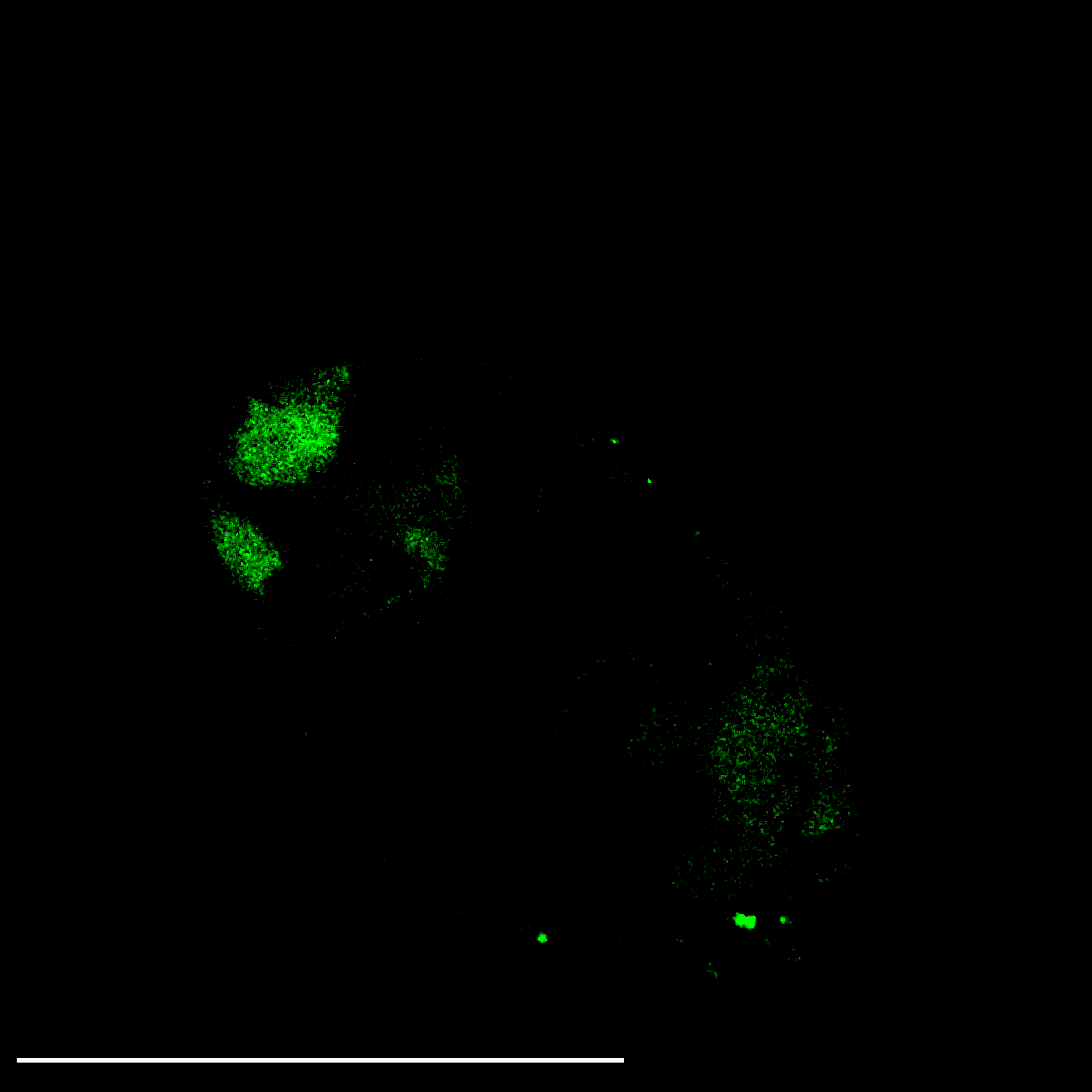

Supplement: Supplementary file 4 — Source Data [file 41467_2022_28500_MOESM4_ESM.zip › Source data/Fig2 D/V-SG_c1 (2).tif]

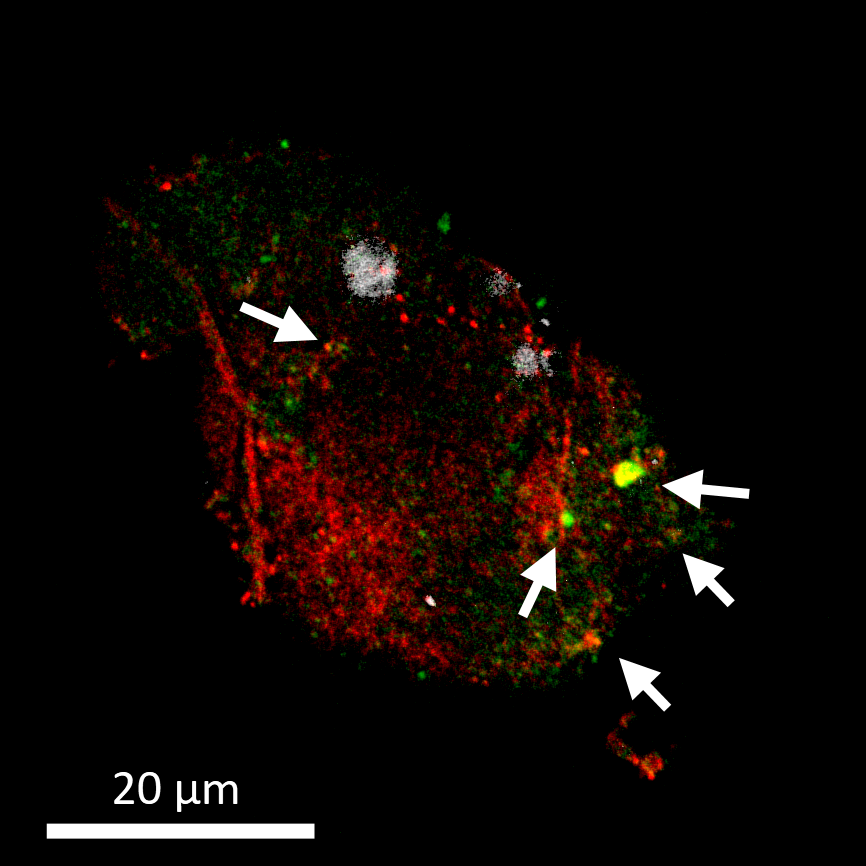

Supplement: Supplementary file 4 — Source Data [file 41467_2022_28500_MOESM4_ESM.zip › Source data/Fig2 D/V-SG_c1+2+3 (2).tif]

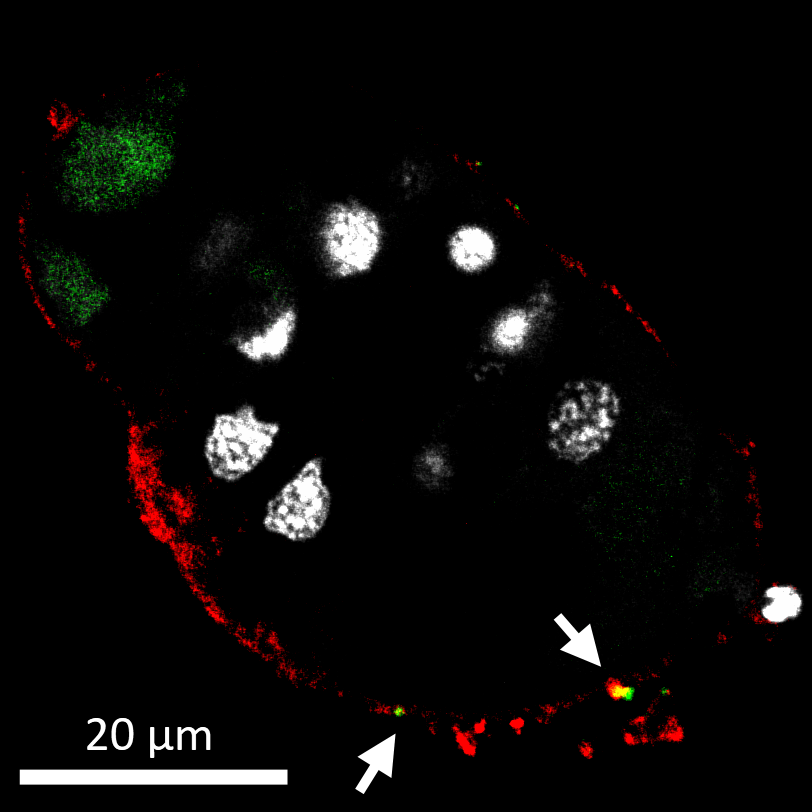

Supplement: Supplementary file 4 — Source Data [file 41467_2022_28500_MOESM4_ESM.zip › Source data/Fig2 D/V-SG_c1+2+3 (3).tif]

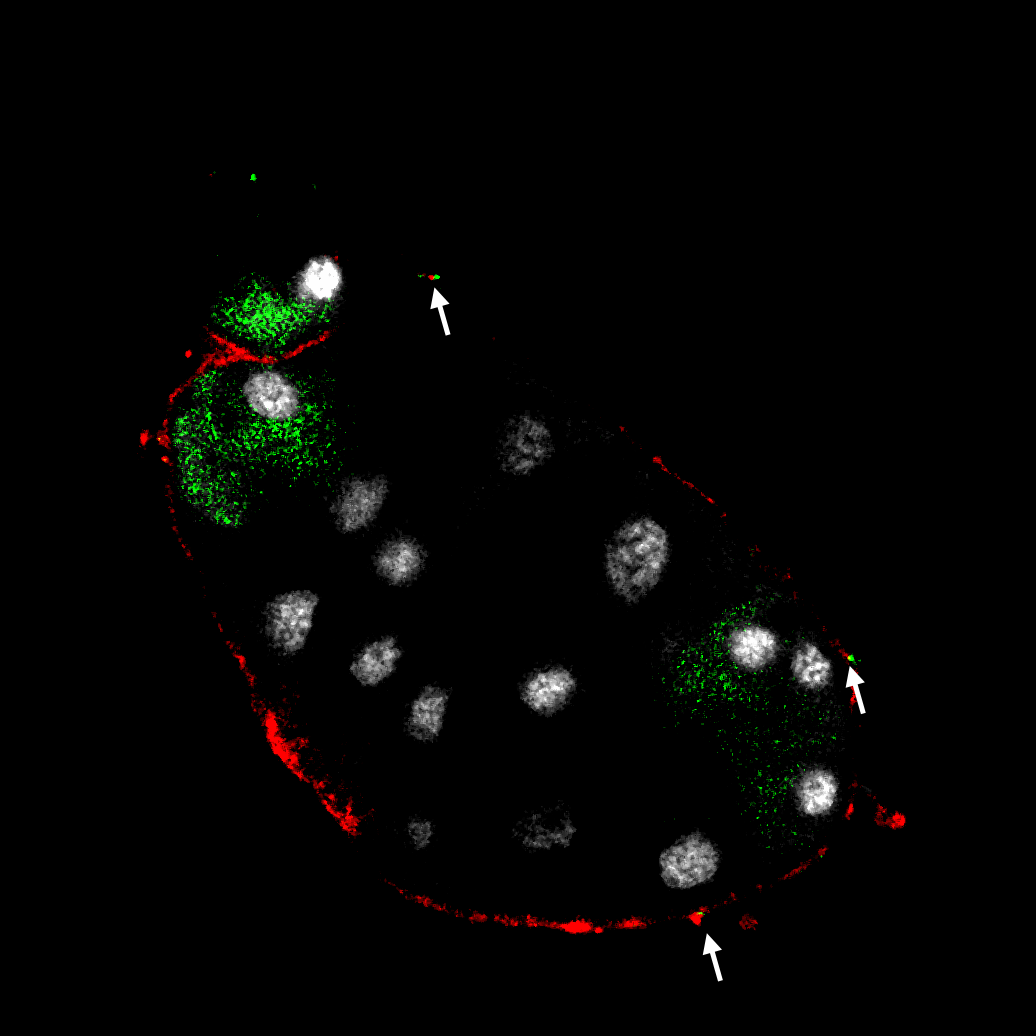

Supplement: Supplementary file 4 — Source Data [file 41467_2022_28500_MOESM4_ESM.zip › Source data/Fig2 D/V-SG_c1+2+3 (4).tif]

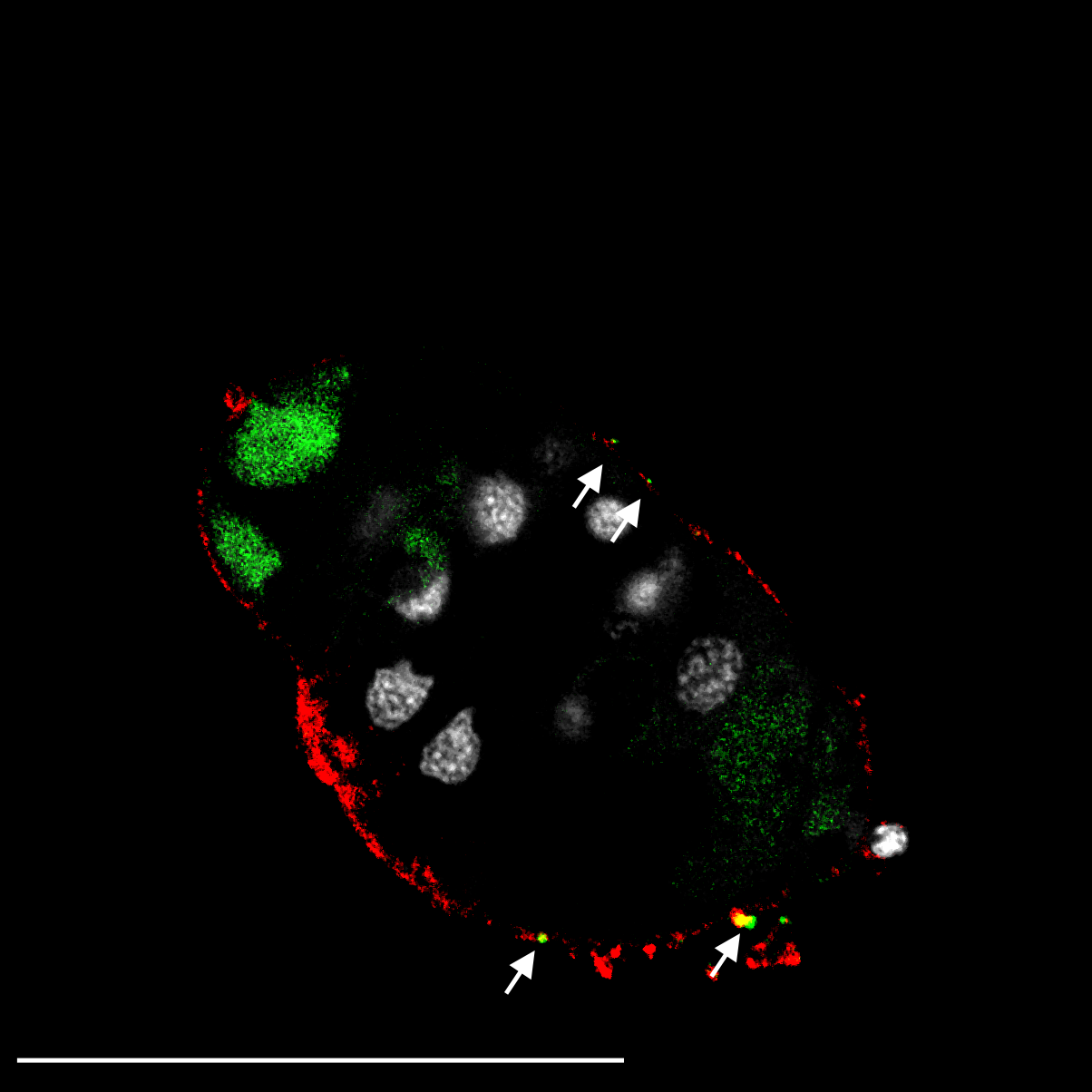

Supplement: Supplementary file 4 — Source Data [file 41467_2022_28500_MOESM4_ESM.zip › Source data/Fig2 D/V-SG_c1+2+3 (5).tif]

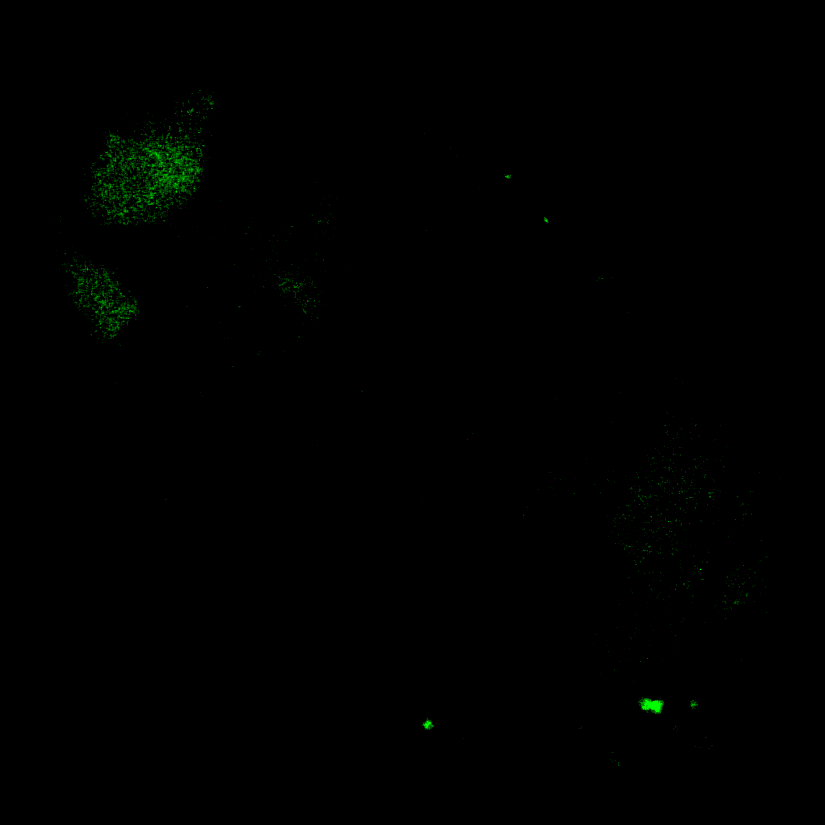

Supplement: Supplementary file 4 — Source Data [file 41467_2022_28500_MOESM4_ESM.zip › Source data/Fig2 D/V-SG_c1.tif]

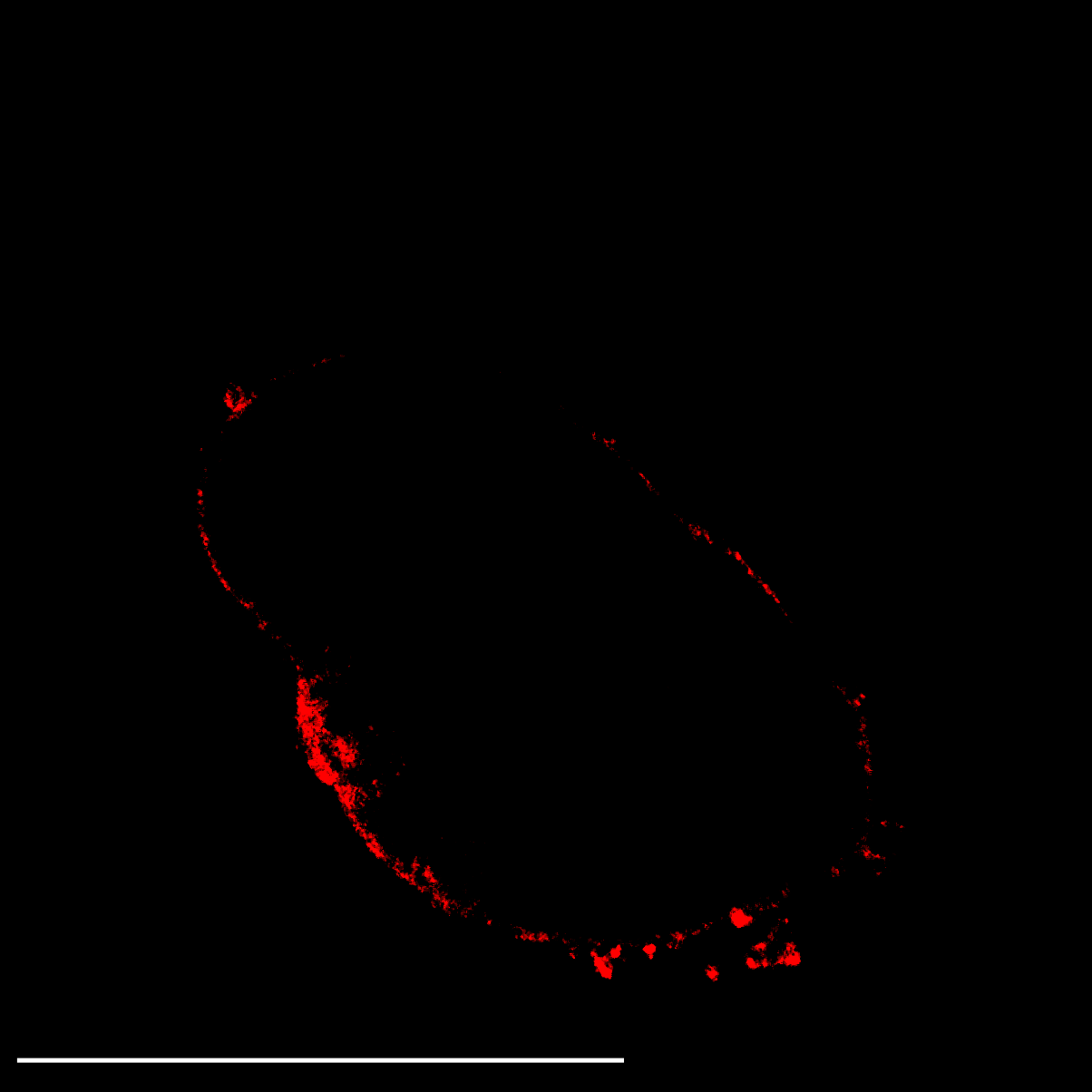

Supplement: Supplementary file 4 — Source Data [file 41467_2022_28500_MOESM4_ESM.zip › Source data/Fig2 D/V-SG_c2 (2).tif]

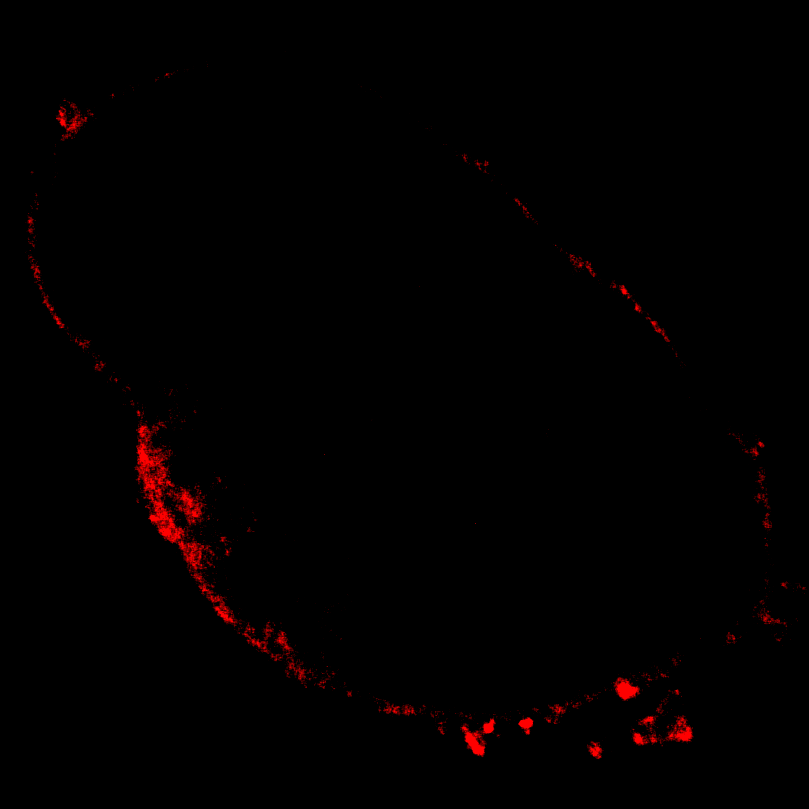

Supplement: Supplementary file 4 — Source Data [file 41467_2022_28500_MOESM4_ESM.zip › Source data/Fig2 D/V-SG_c2.tif]

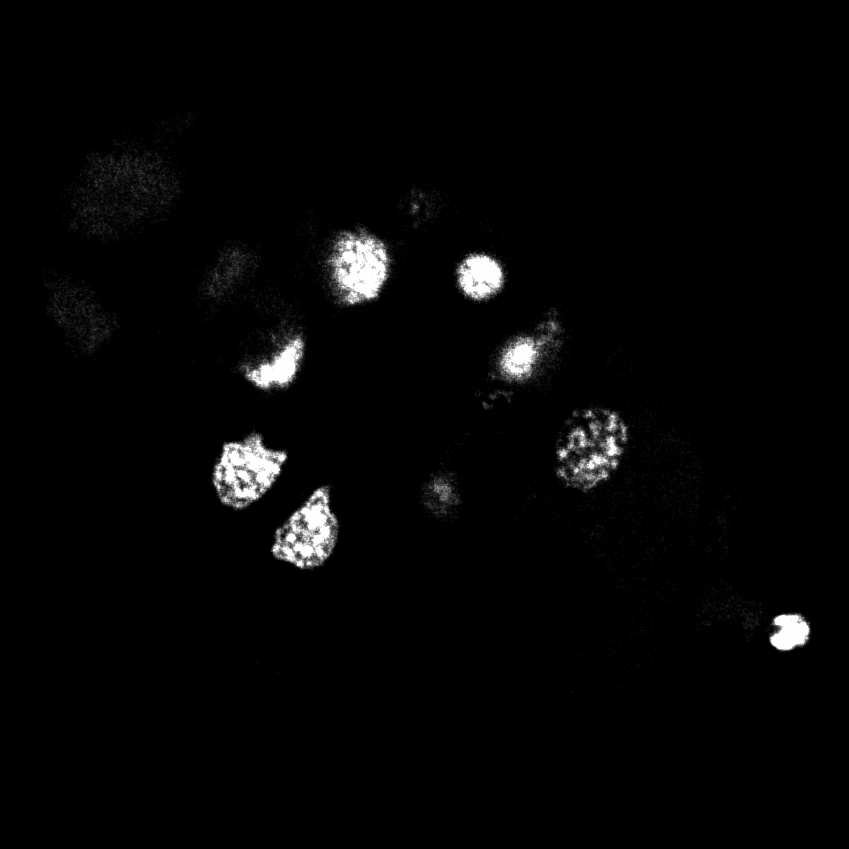

Supplement: Supplementary file 4 — Source Data [file 41467_2022_28500_MOESM4_ESM.zip › Source data/Fig2 D/V-SG_c3 (2).tif]

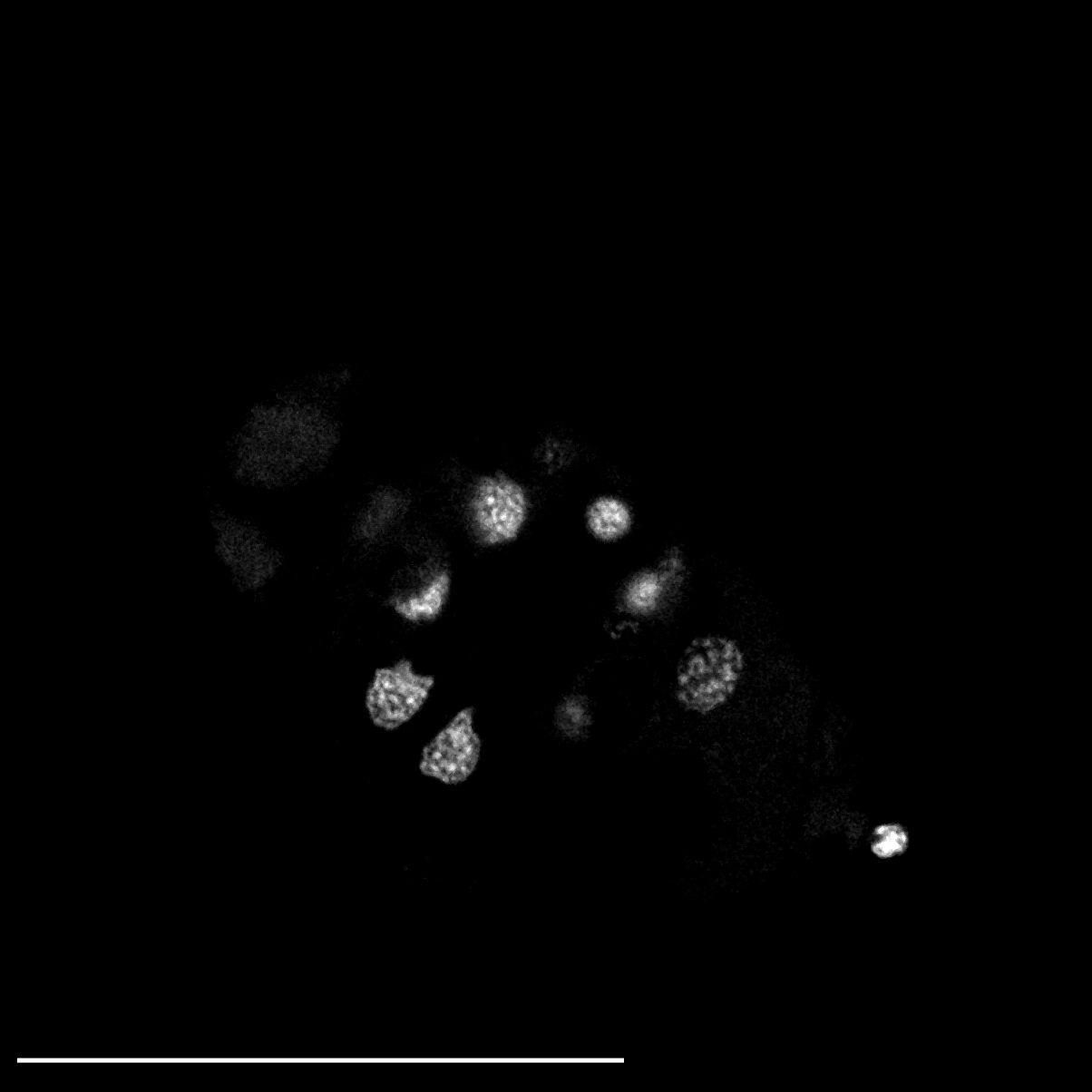

Supplement: Supplementary file 4 — Source Data [file 41467_2022_28500_MOESM4_ESM.zip › Source data/Fig2 D/V-SG_c3.tif]

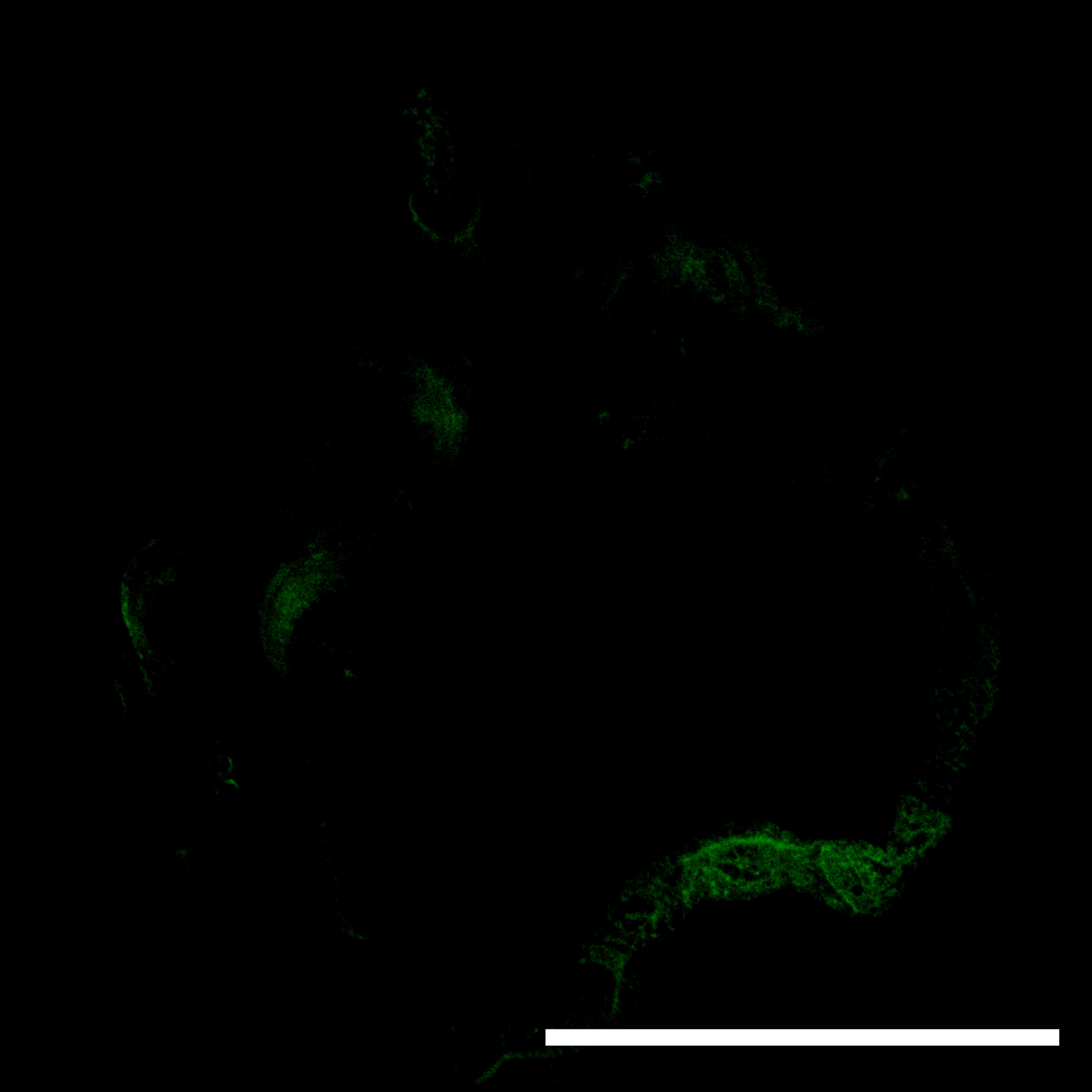

Supplement: Supplementary file 4 — Source Data [file 41467_2022_28500_MOESM4_ESM.zip › Source data/Fig3 D/MG-dsGFPr_c2.jpg]

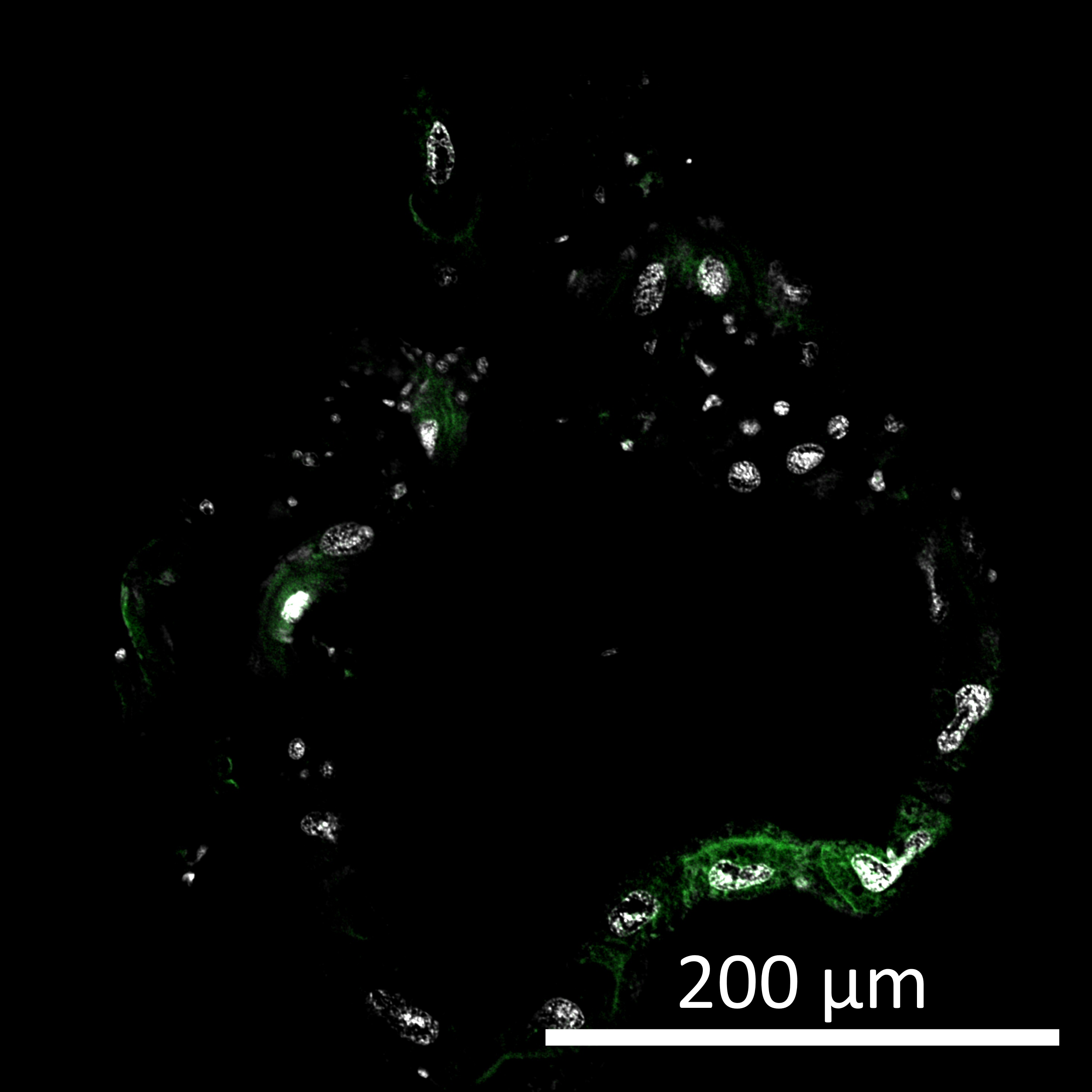

Supplement: Supplementary file 4 — Source Data [file 41467_2022_28500_MOESM4_ESM.zip › Source data/Fig3 D/MG-dsGFP_c1+2.jpg]

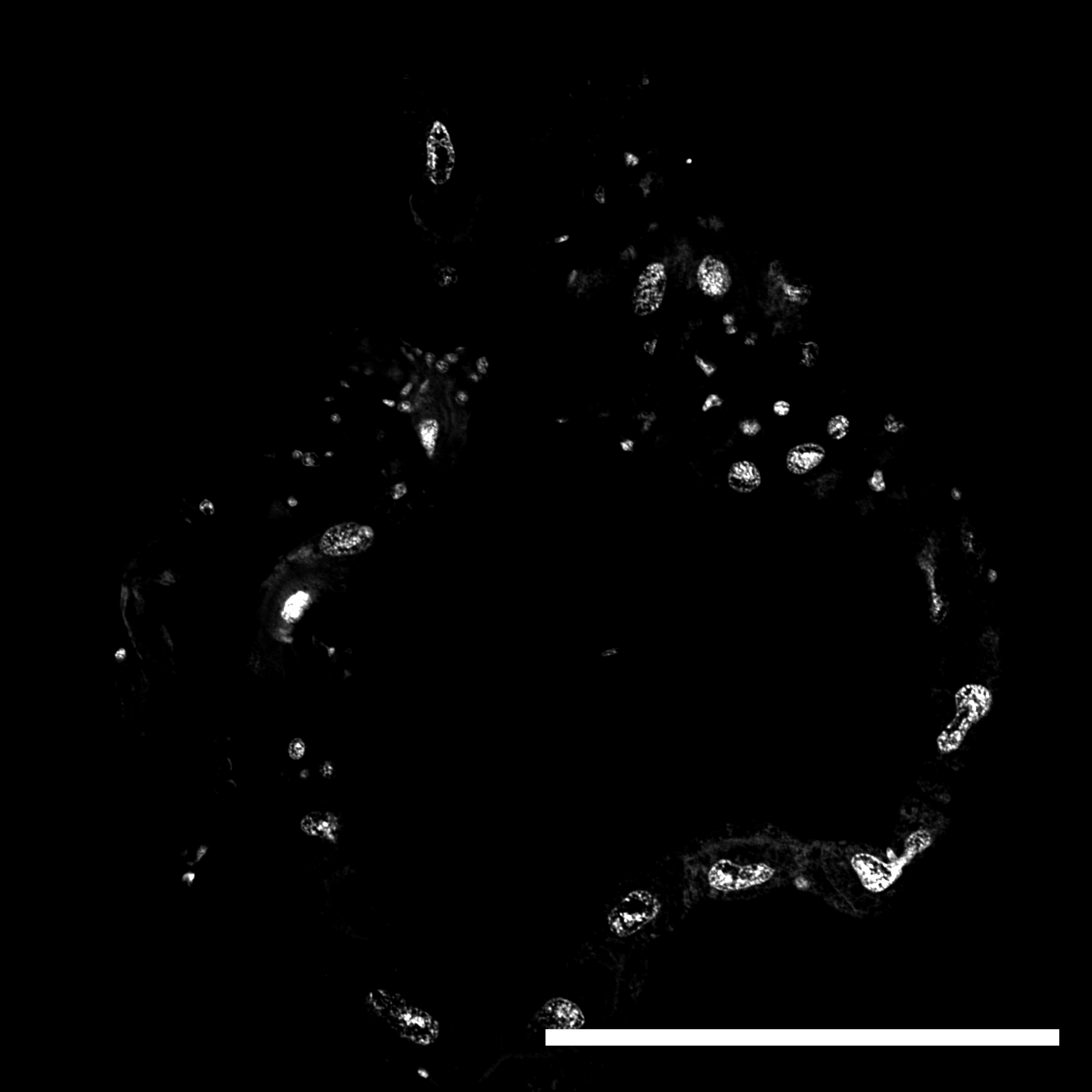

Supplement: Supplementary file 4 — Source Data [file 41467_2022_28500_MOESM4_ESM.zip › Source data/Fig3 D/MG-dsGFP_c1.jpg]

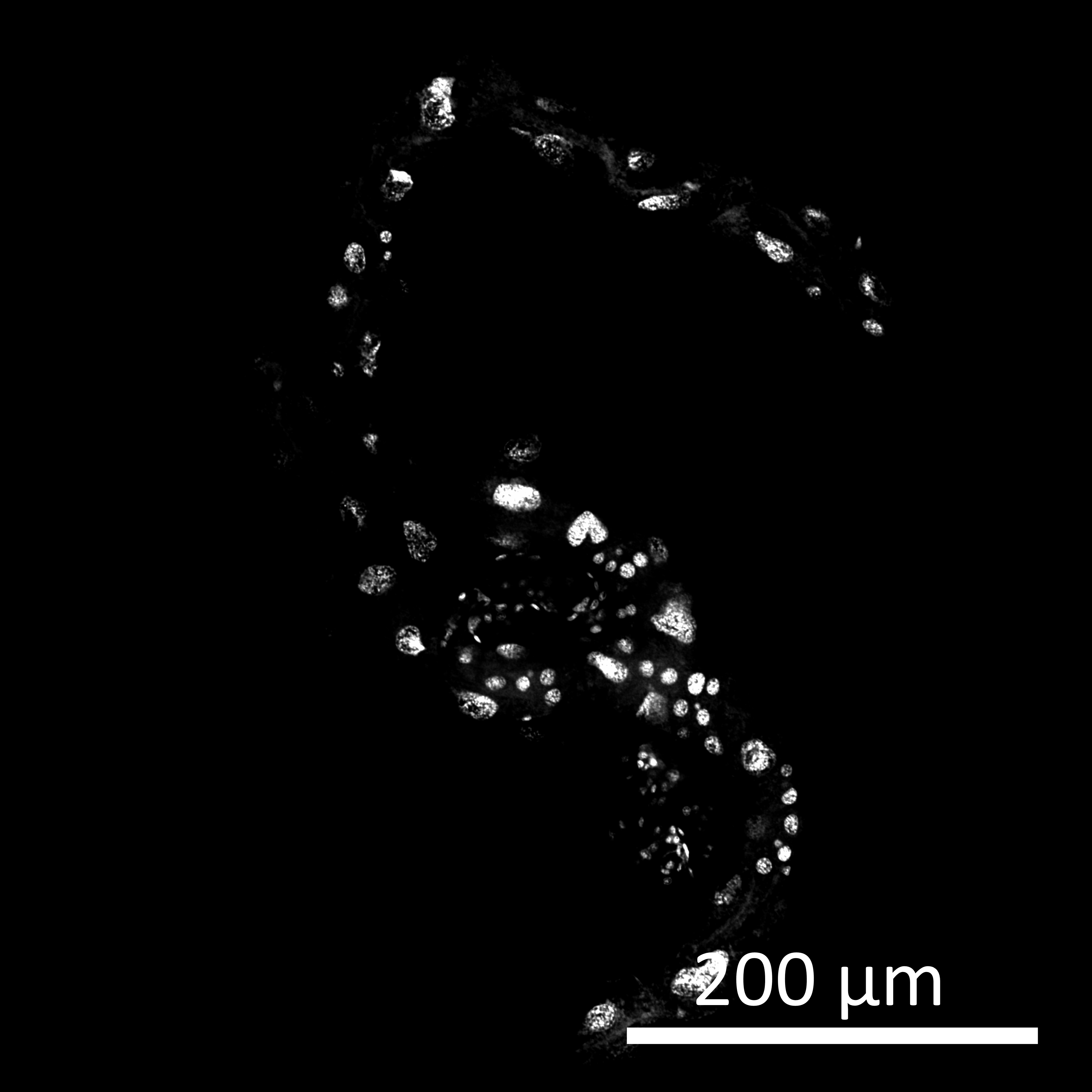

Supplement: Supplementary file 4 — Source Data [file 41467_2022_28500_MOESM4_ESM.zip › Source data/Fig3 D/MG-dsPEBP4_c1+2.jpg]

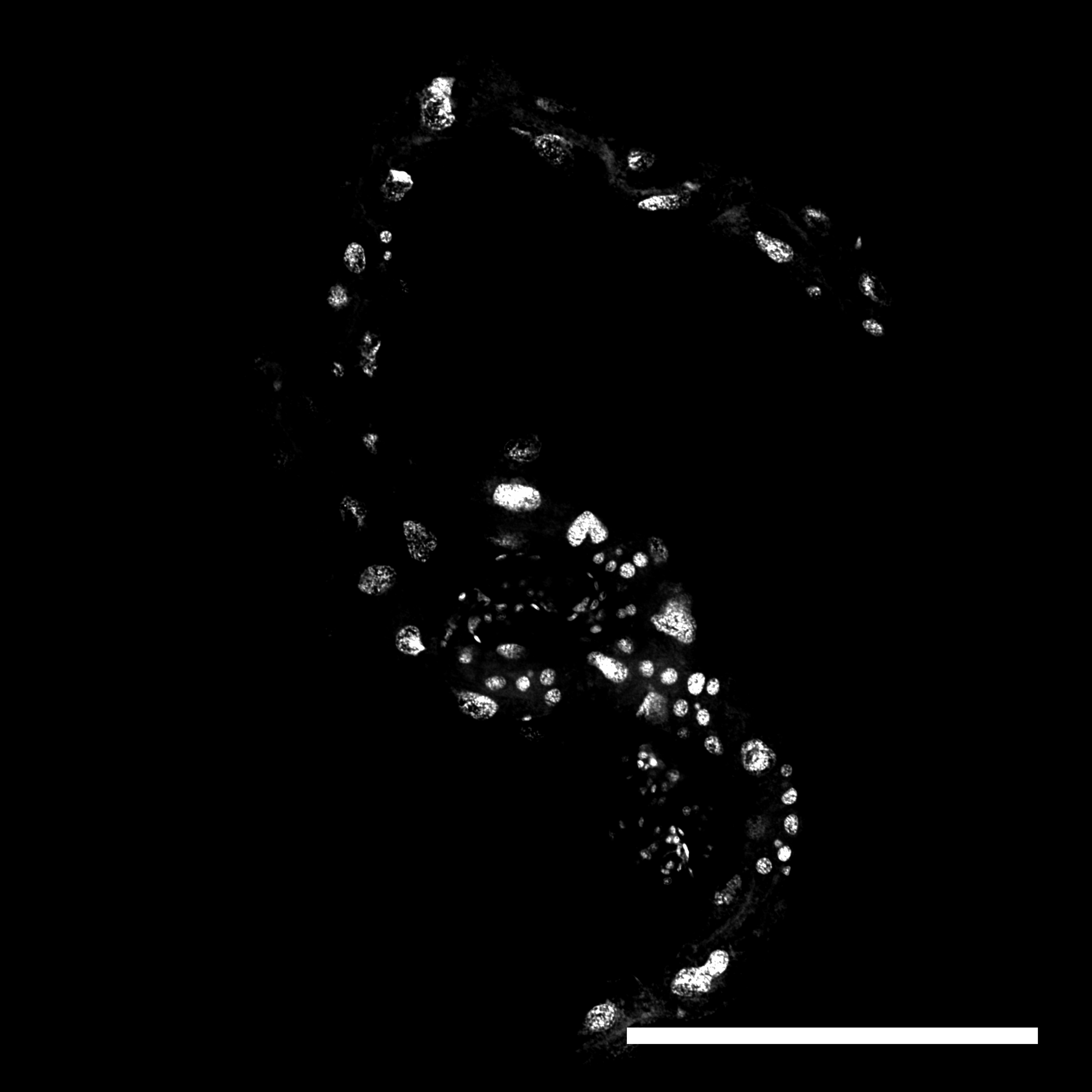

Supplement: Supplementary file 4 — Source Data [file 41467_2022_28500_MOESM4_ESM.zip › Source data/Fig3 D/MG-dsPEBP4_c1.jpg]

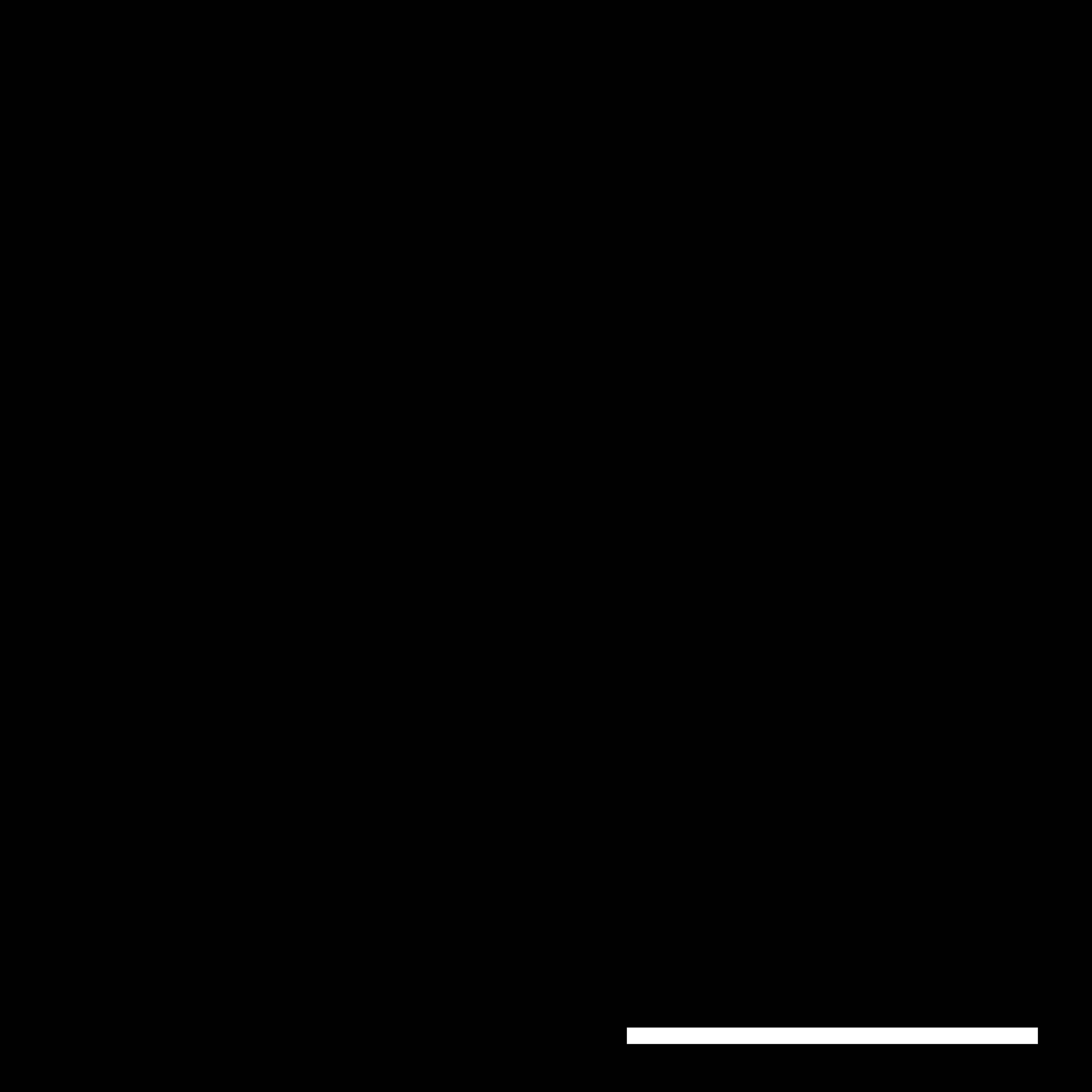

Supplement: Supplementary file 4 — Source Data [file 41467_2022_28500_MOESM4_ESM.zip › Source data/Fig3 D/MG-dsPEBP4_c2.jpg]

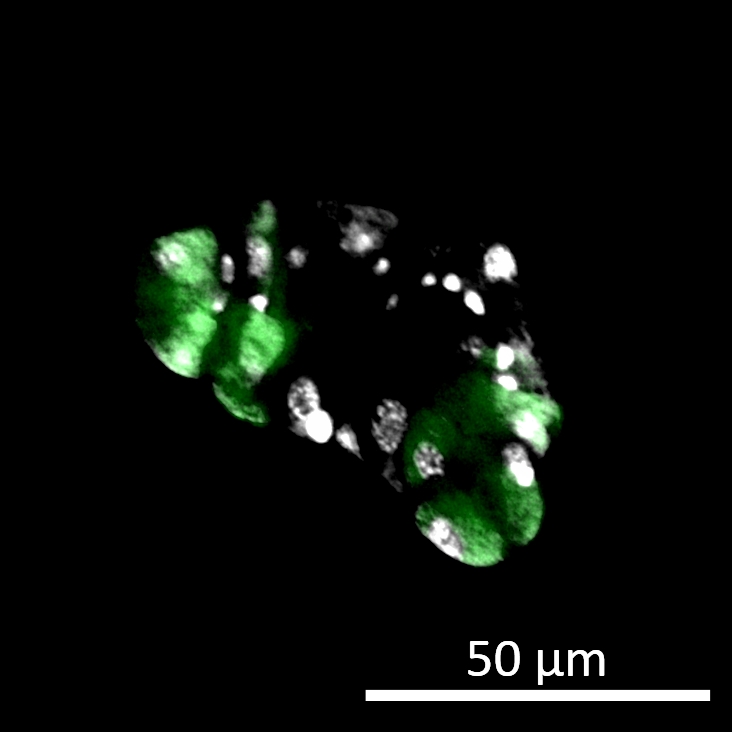

Supplement: Supplementary file 4 — Source Data [file 41467_2022_28500_MOESM4_ESM.zip › Source data/Fig3 D/SG-dsGFP_c1+2.jpg]

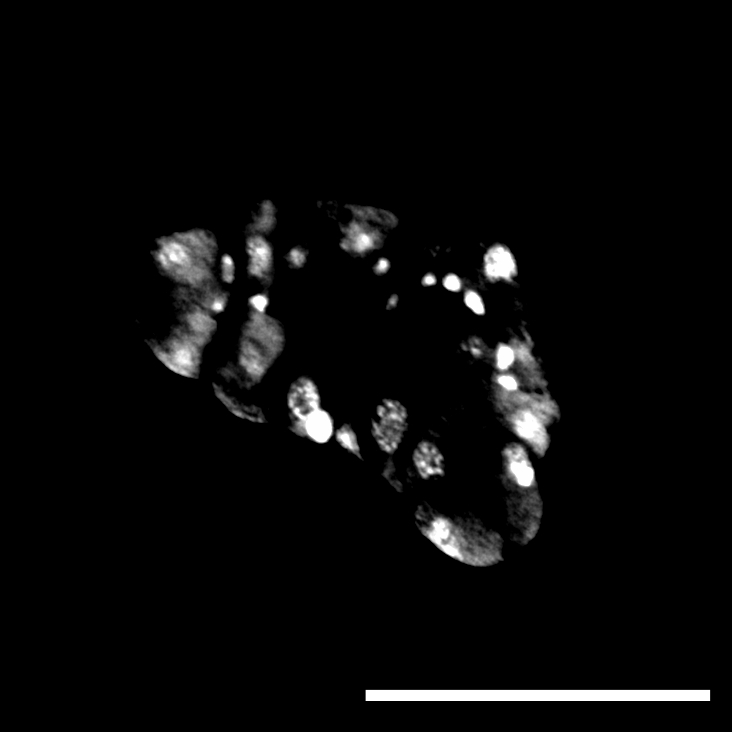

Supplement: Supplementary file 4 — Source Data [file 41467_2022_28500_MOESM4_ESM.zip › Source data/Fig3 D/SG-dsGFP_c1.jpg]

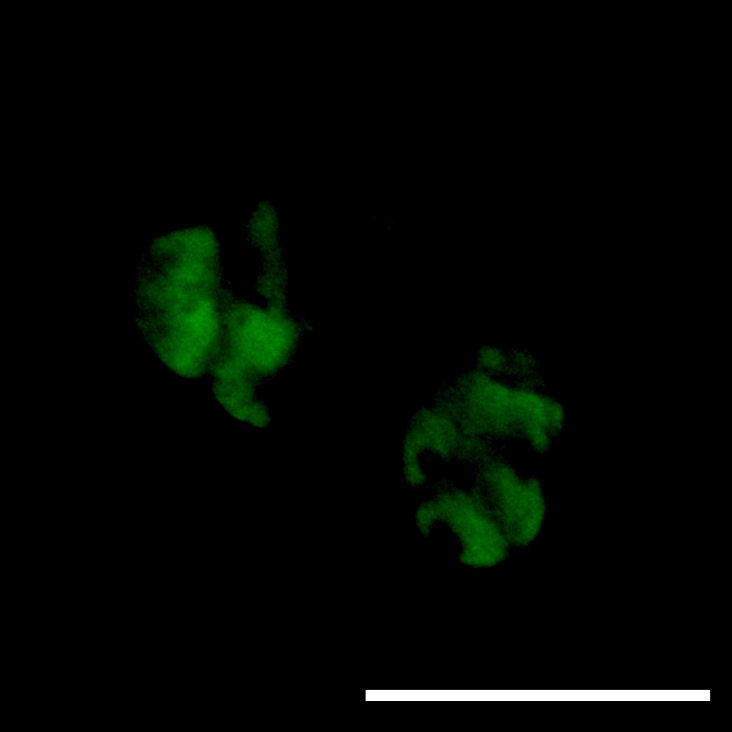

Supplement: Supplementary file 4 — Source Data [file 41467_2022_28500_MOESM4_ESM.zip › Source data/Fig3 D/SG-dsGFP_c2.jpg]

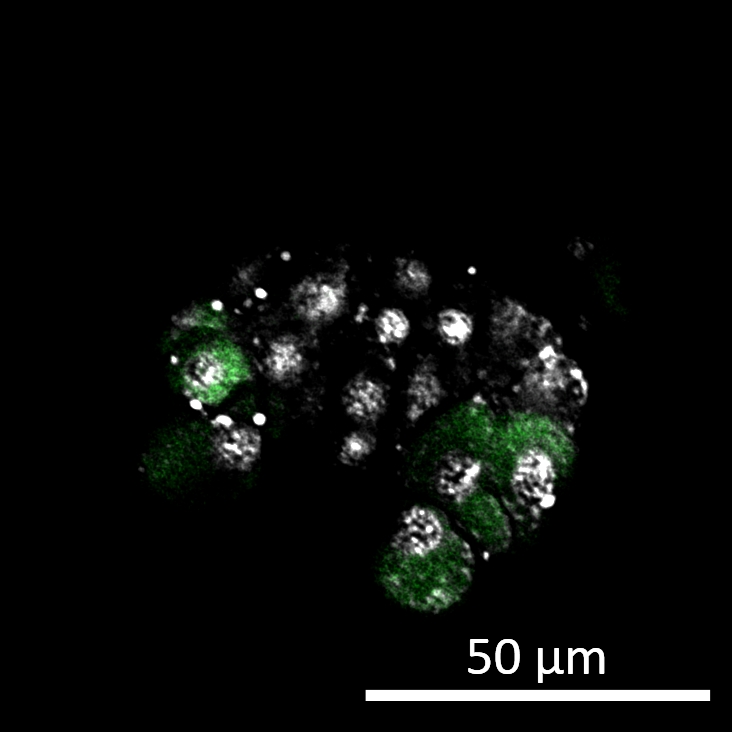

Supplement: Supplementary file 4 — Source Data [file 41467_2022_28500_MOESM4_ESM.zip › Source data/Fig3 D/SG-dsPEBP4_c1+2.jpg]

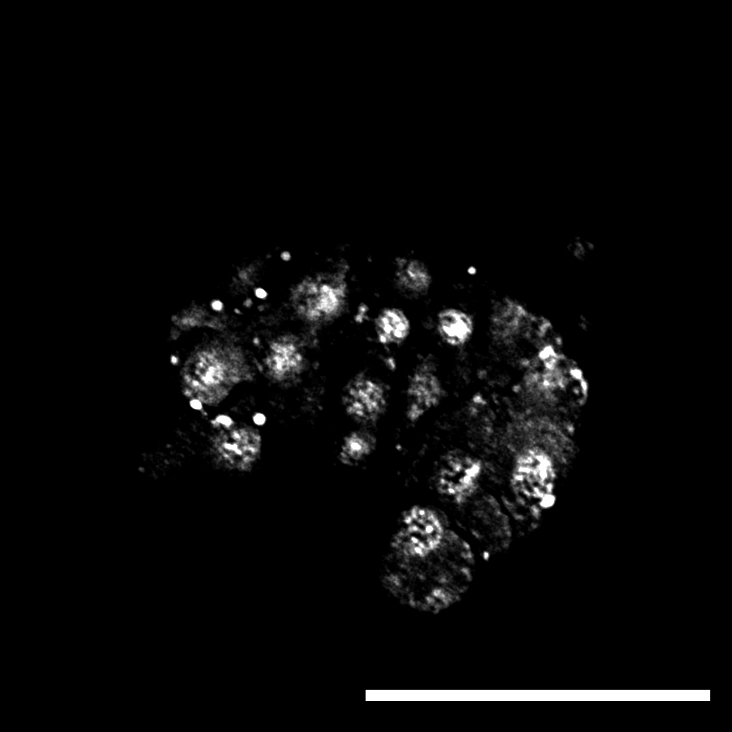

Supplement: Supplementary file 4 — Source Data [file 41467_2022_28500_MOESM4_ESM.zip › Source data/Fig3 D/SG-dsPEBP4_c1.jpg]

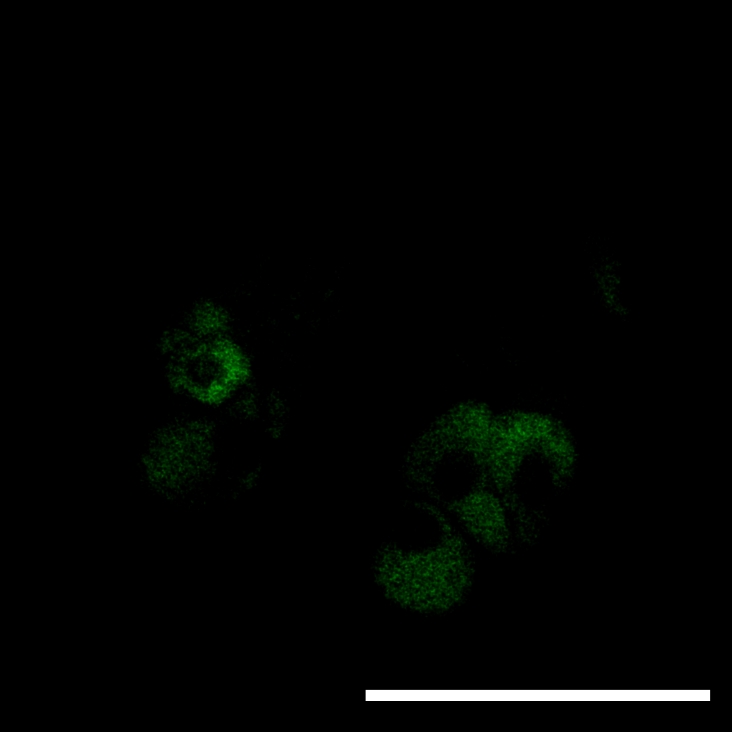

Supplement: Supplementary file 4 — Source Data [file 41467_2022_28500_MOESM4_ESM.zip › Source data/Fig3 D/SG-dsPEBP4_c2.jpg]

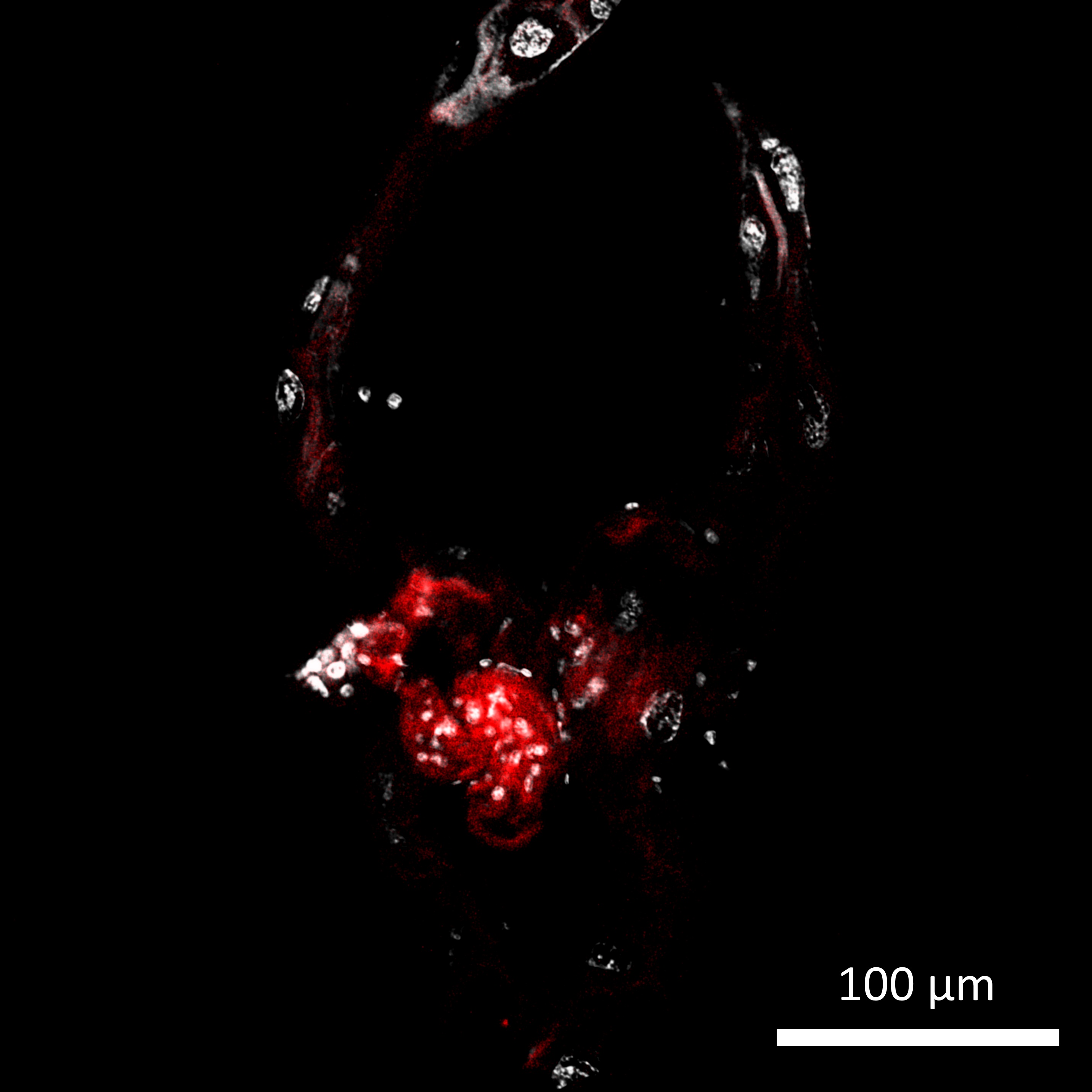

Supplement: Supplementary file 4 — Source Data [file 41467_2022_28500_MOESM4_ESM.zip › Source data/Fig3 E/ATG8-dsGFP(CP)-MG_c1+2.jpg]

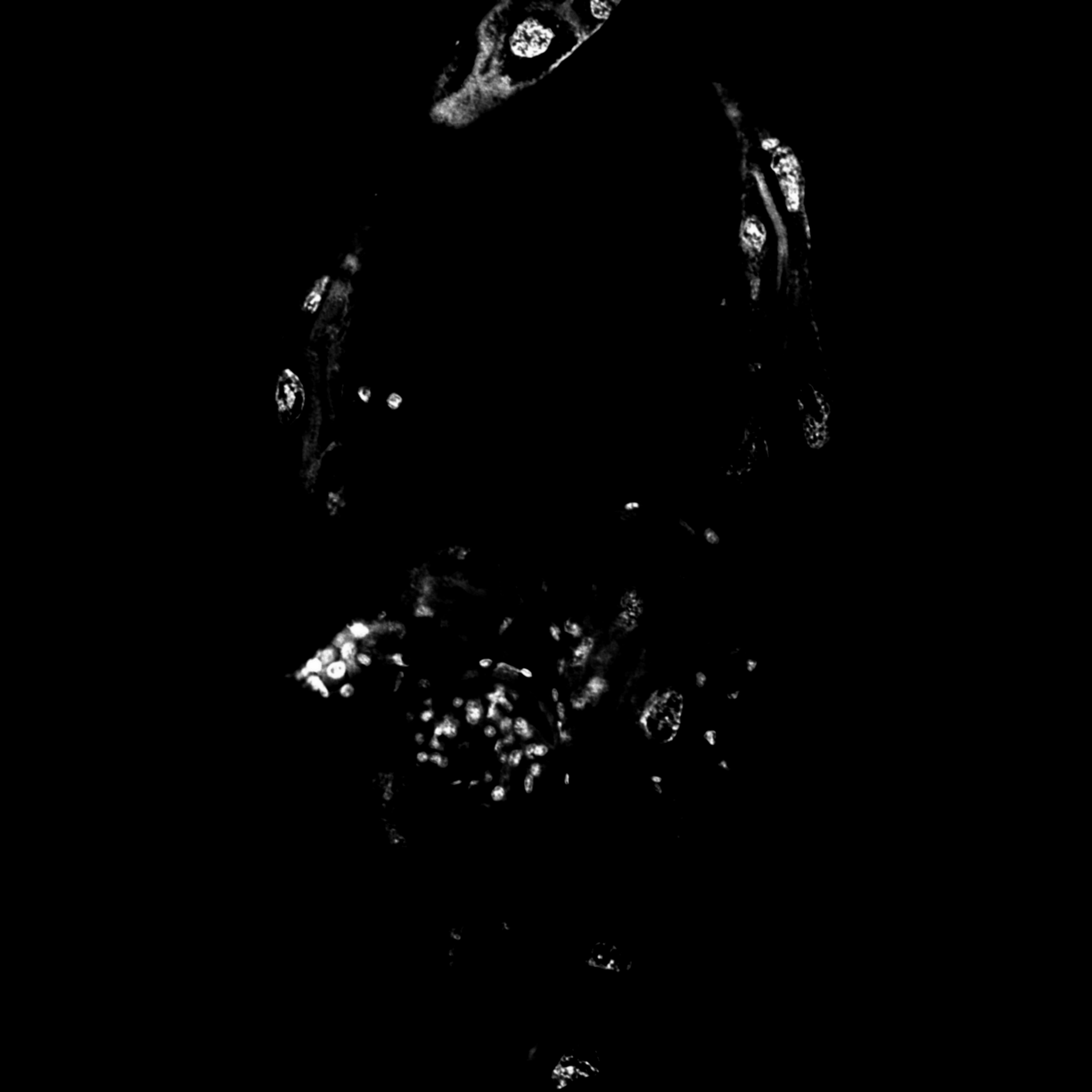

Supplement: Supplementary file 4 — Source Data [file 41467_2022_28500_MOESM4_ESM.zip › Source data/Fig3 E/ATG8-dsGFP(CP)-MG_c1.jpg]

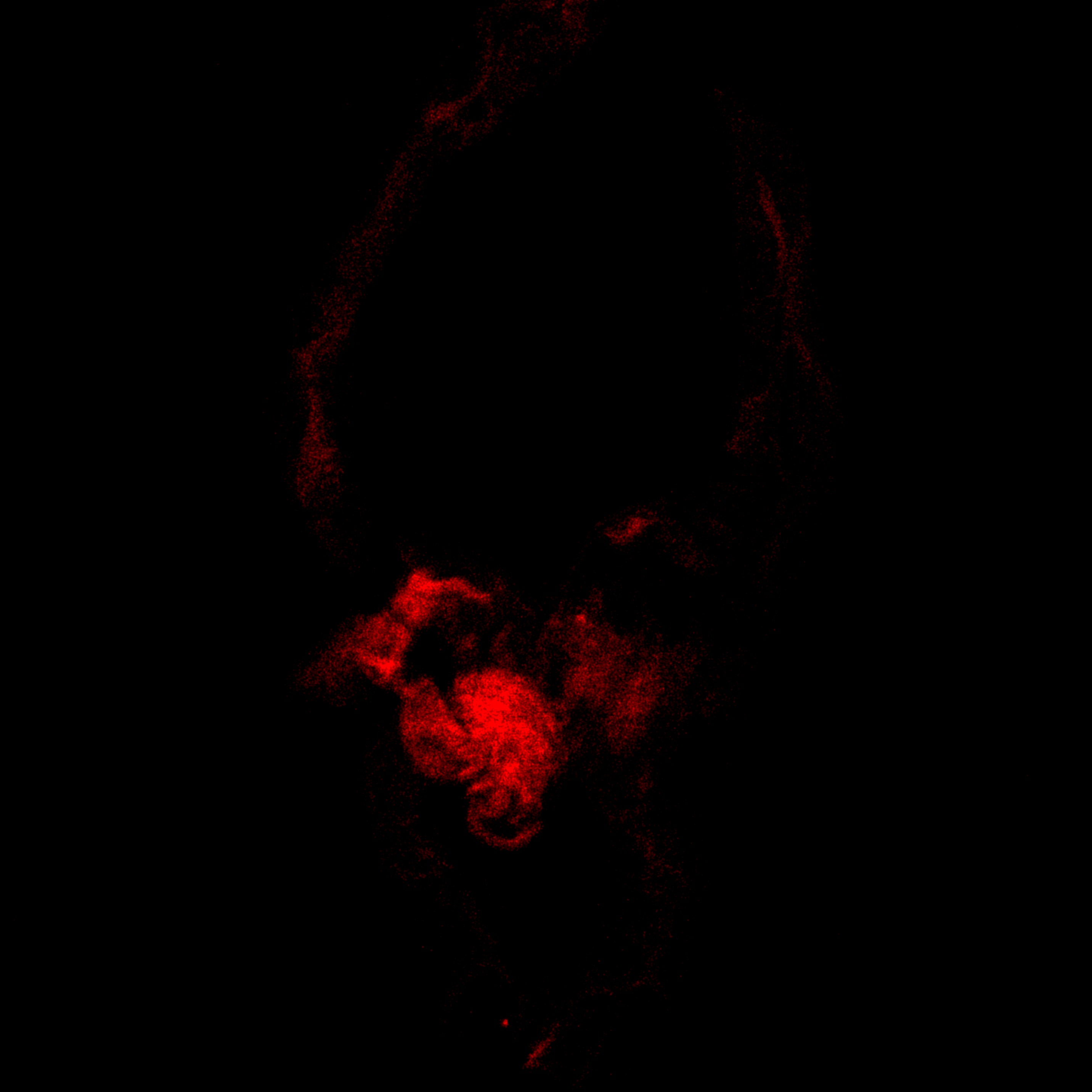

Supplement: Supplementary file 4 — Source Data [file 41467_2022_28500_MOESM4_ESM.zip › Source data/Fig3 E/ATG8-dsGFP(CP)-MG_c2.jpg]

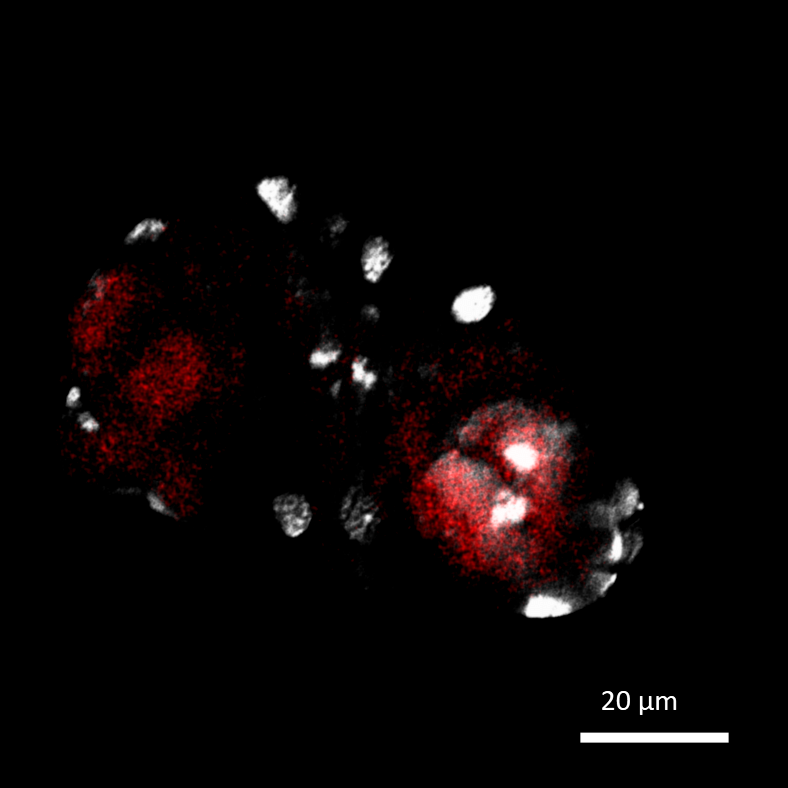

Supplement: Supplementary file 4 — Source Data [file 41467_2022_28500_MOESM4_ESM.zip › Source data/Fig3 E/ATG8-dsGFP(CP)-SG_c1+2.tif]

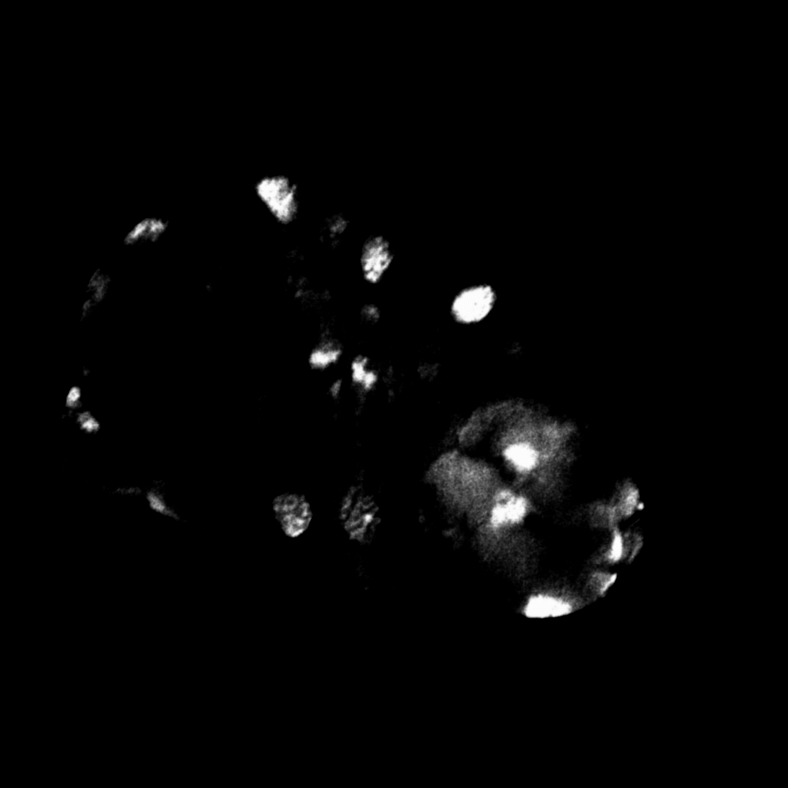

Supplement: Supplementary file 4 — Source Data [file 41467_2022_28500_MOESM4_ESM.zip › Source data/Fig3 E/ATG8-dsGFP(CP)-SG_c1.tif]

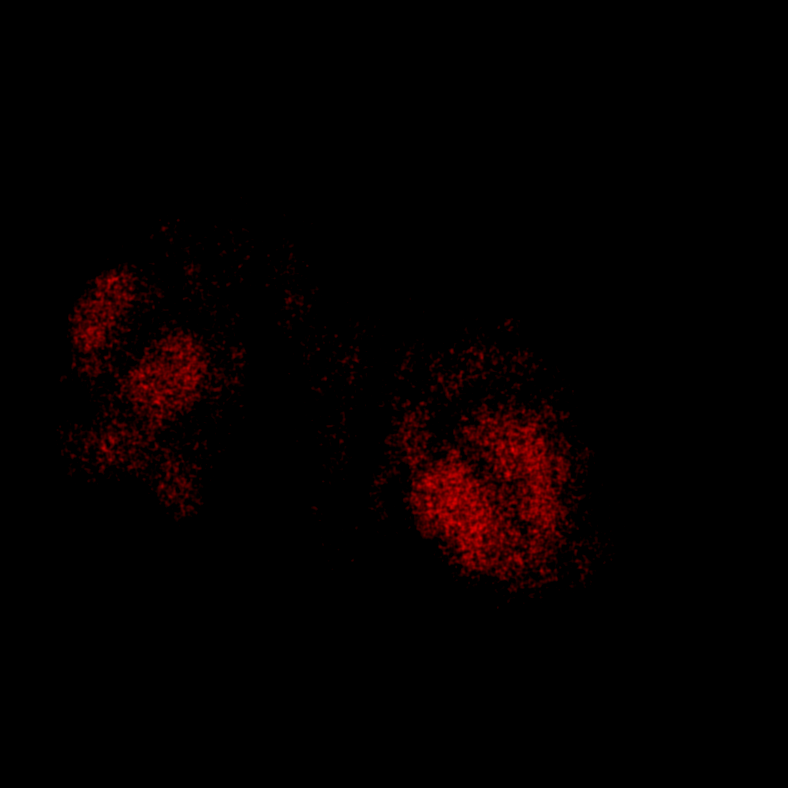

Supplement: Supplementary file 4 — Source Data [file 41467_2022_28500_MOESM4_ESM.zip › Source data/Fig3 E/ATG8-dsGFP(CP)-SG_c2.tif]

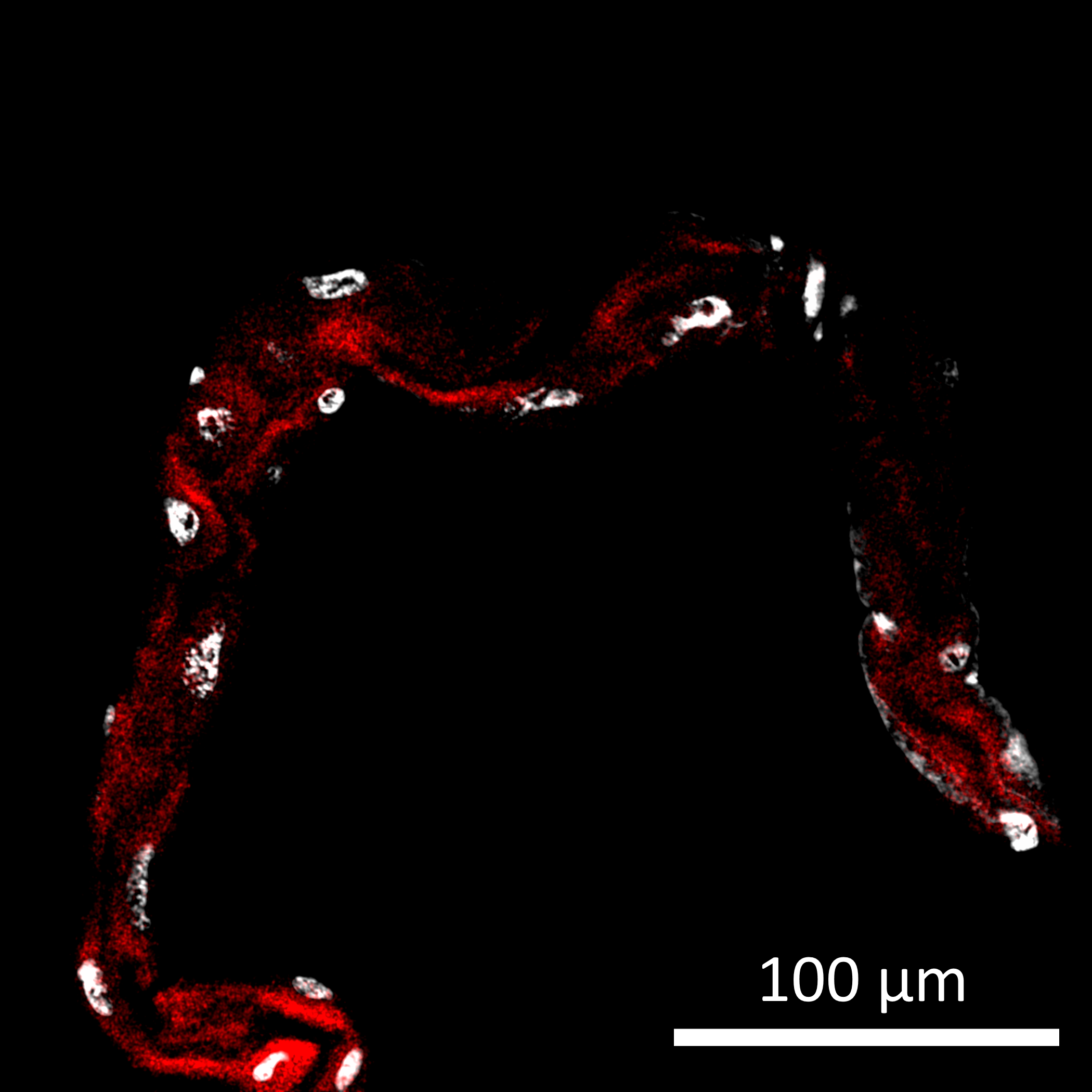

Supplement: Supplementary file 4 — Source Data [file 41467_2022_28500_MOESM4_ESM.zip › Source data/Fig3 E/ATG8-dsPEBP4(CP)-MG_c1+2.tif]

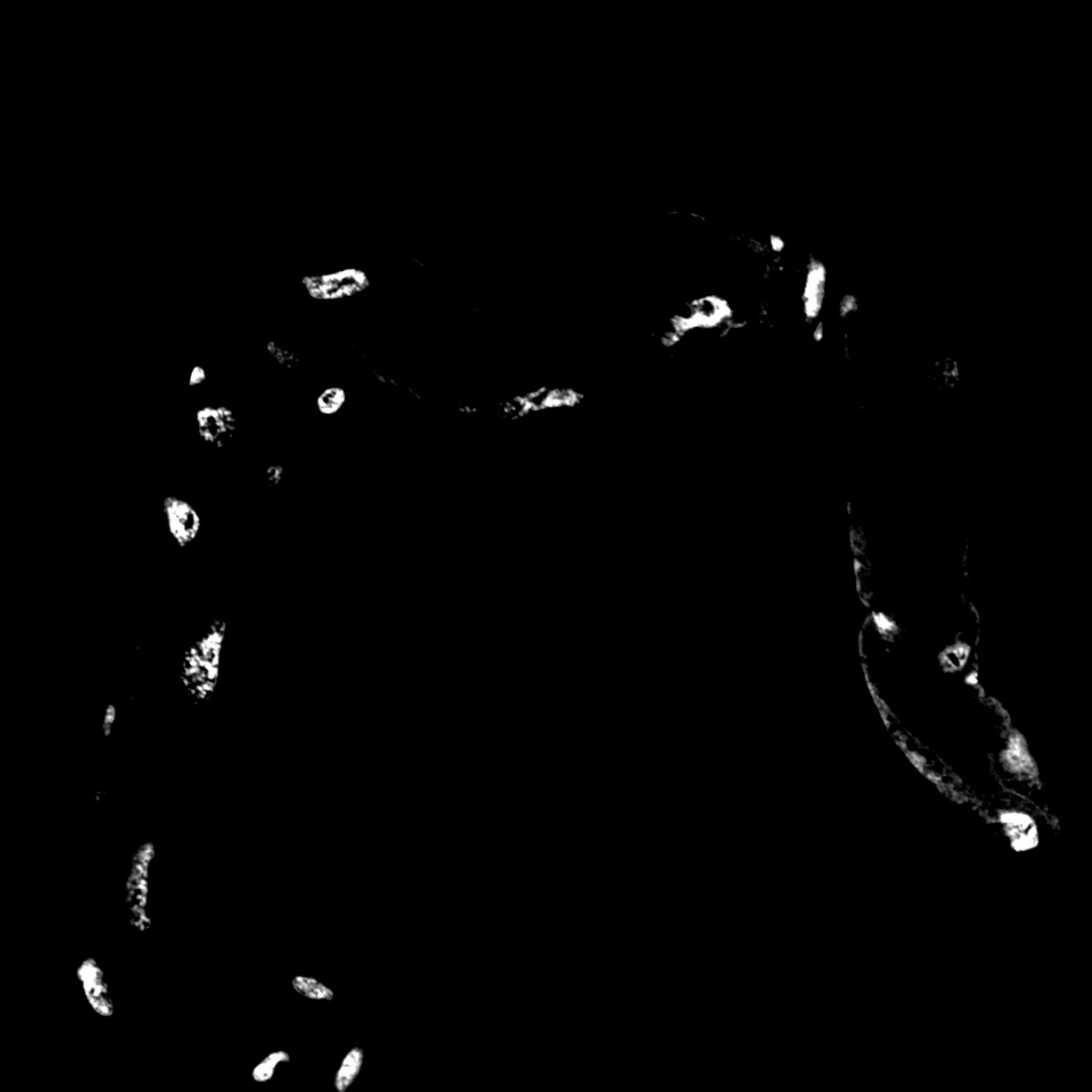

Supplement: Supplementary file 4 — Source Data [file 41467_2022_28500_MOESM4_ESM.zip › Source data/Fig3 E/ATG8-dsPEBP4(CP)-MG_c1.tif]

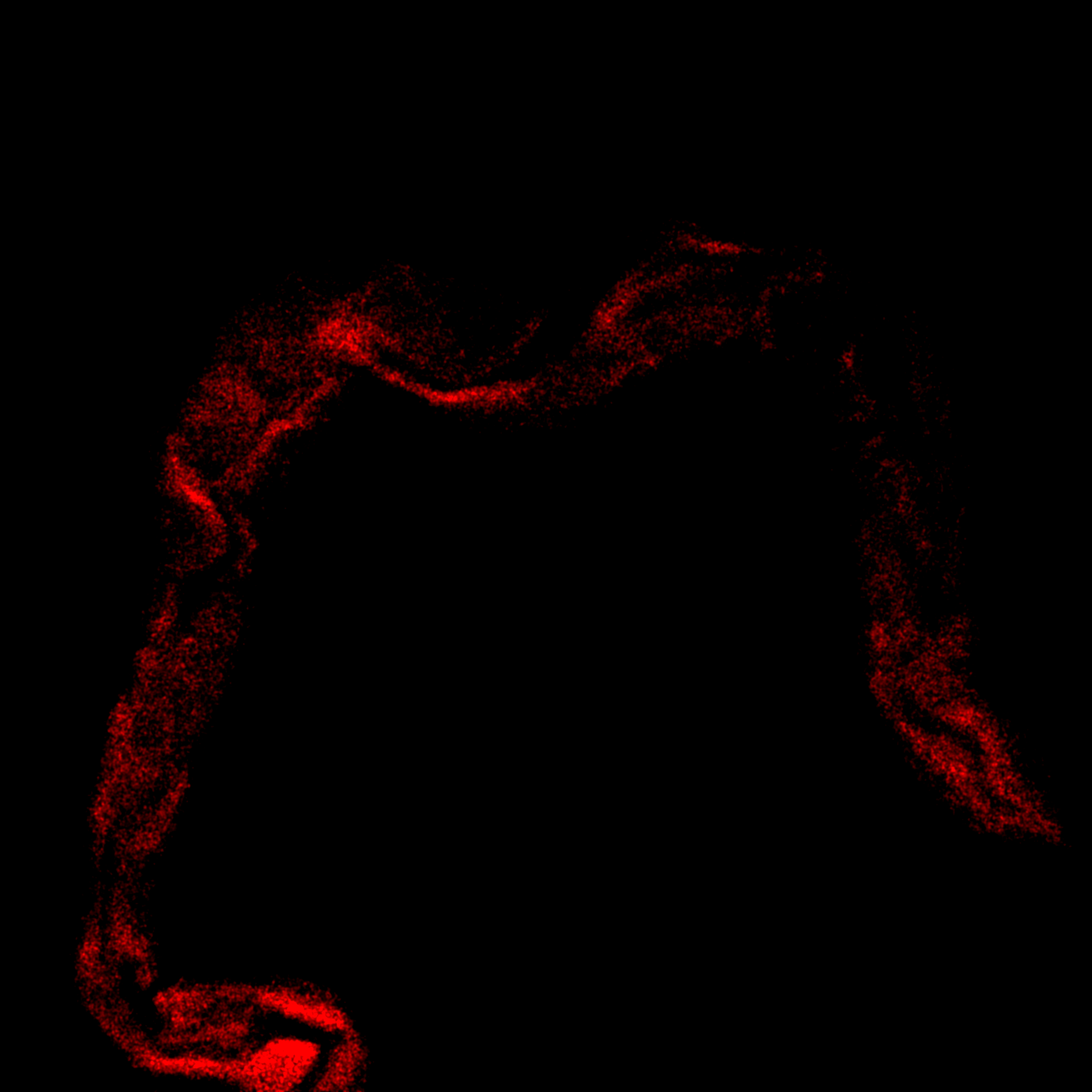

Supplement: Supplementary file 4 — Source Data [file 41467_2022_28500_MOESM4_ESM.zip › Source data/Fig3 E/ATG8-dsPEBP4(CP)-MG_c2.tif]

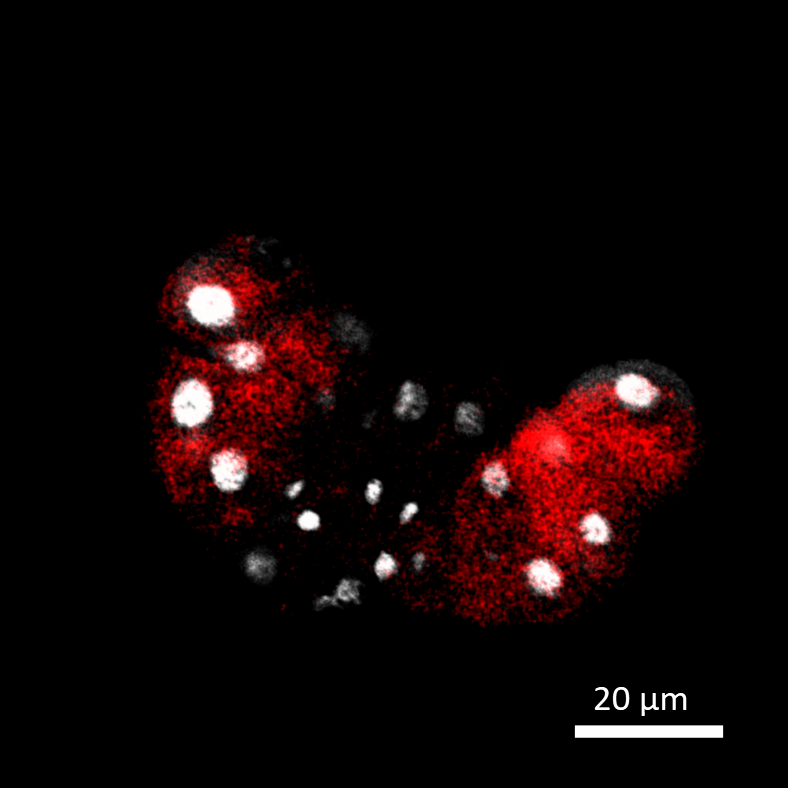

Supplement: Supplementary file 4 — Source Data [file 41467_2022_28500_MOESM4_ESM.zip › Source data/Fig3 E/ATG8-dsPEBP4(CP)-SG_c1+2.tif]

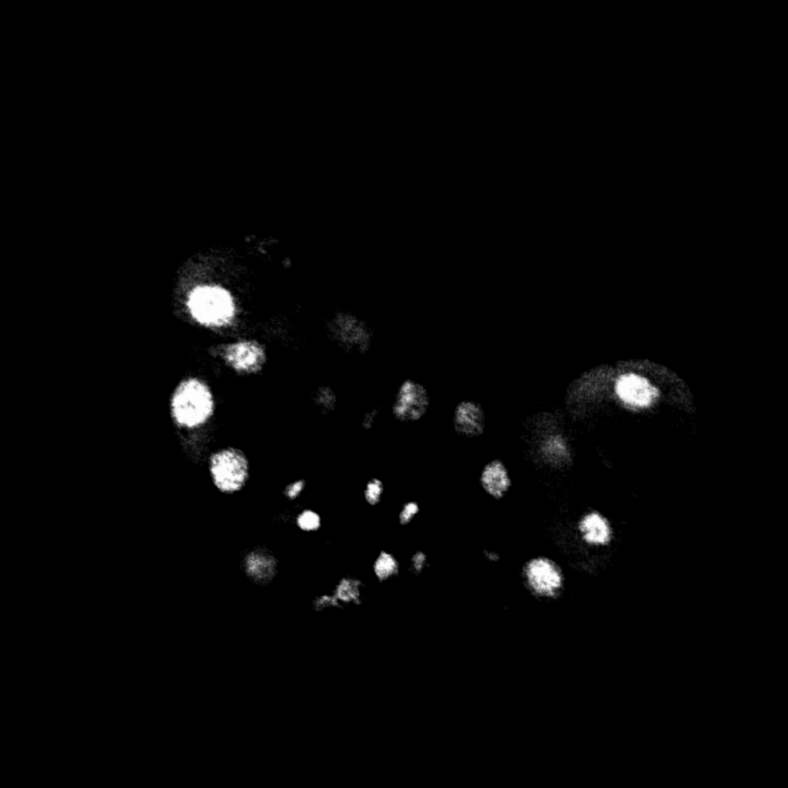

Supplement: Supplementary file 4 — Source Data [file 41467_2022_28500_MOESM4_ESM.zip › Source data/Fig3 E/ATG8-dsPEBP4(CP)-SG_c1.tif]

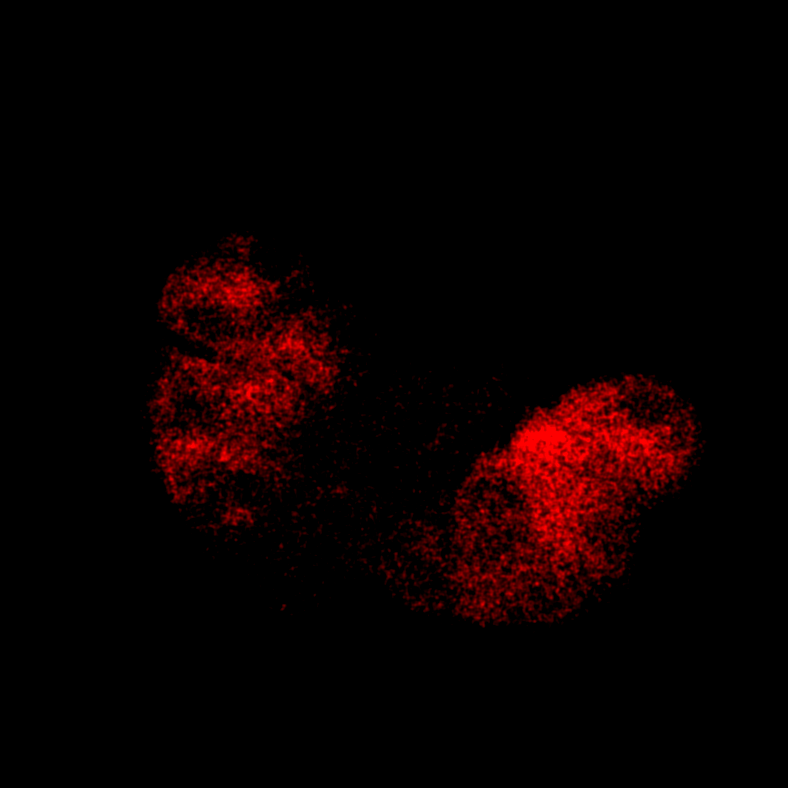

Supplement: Supplementary file 4 — Source Data [file 41467_2022_28500_MOESM4_ESM.zip › Source data/Fig3 E/ATG8-dsPEBP4(CP)-SG_c2.tif]

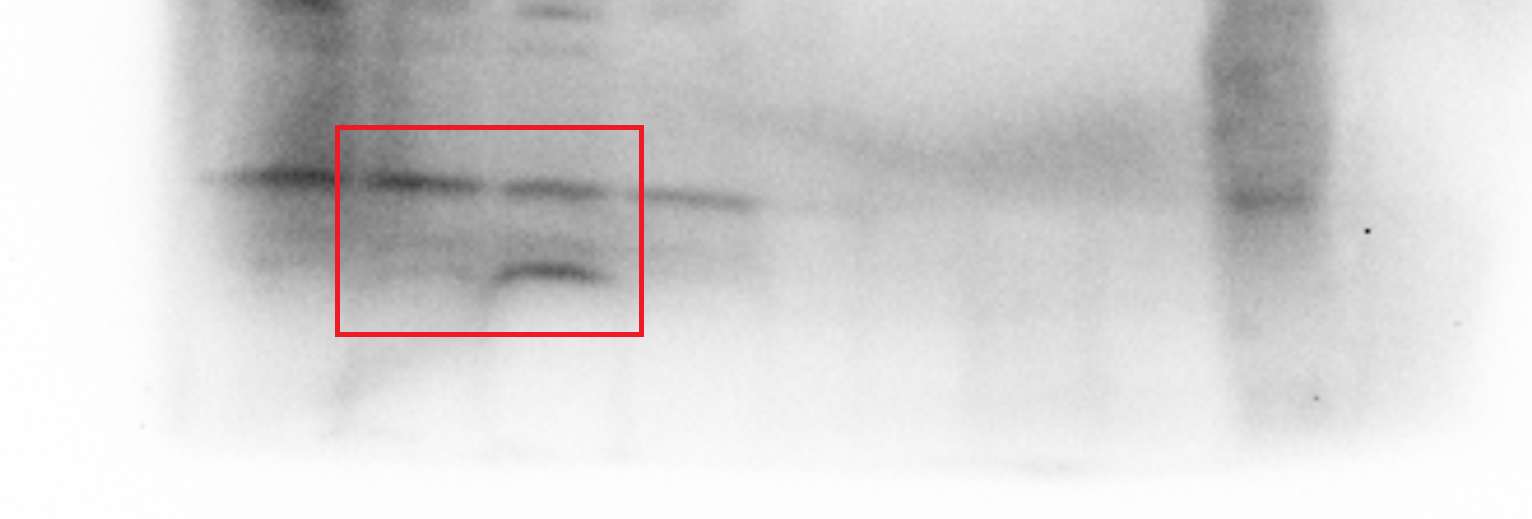

Supplement: Supplementary file 4 — Source Data [file 41467_2022_28500_MOESM4_ESM.zip › Source data/Fig3 F/ATG8.tif]
